# Compensating for over-production inhibition of the Hsmar1 transposon in *Escherichia coli* using a series of constitutive promoters

**DOI:** 10.1186/s13100-020-0200-5

**Published:** 2020-01-10

**Authors:** Michael Tellier, Ronald Chalmers

**Affiliations:** 1 School of Life Sciences, University of Nottingham, Queen’s Medical Centre, Nottingham, NG7 2UH UK; 2 Sir William Dunn School of Pathology, University of Oxford, Oxford, OX1 3RE UK

**Keywords:** Papillation assay, Hsmar1, Overproduction inhibition, SETMAR, Transposase, Transposable elements

## Abstract

**Background:**

Transposable elements (TEs) are a diverse group of self-mobilizing DNA elements. Transposition has been exploited as a powerful tool for molecular biology and genomics. However, transposition is sometimes limited because of auto-regulatory mechanisms that presumably allow them to cohabit within their hosts without causing excessive genomic damage. The papillation assay provides a powerful visual screen for hyperactive transposases. Transposition is revealed by the activation of a promoter-less *lacZ* gene when the transposon integrates into a non-essential gene on the host chromosome. Transposition events are detected as small blue speckles, or papillae, on the white background of the main *Escherichia coli* colony.

**Results:**

We analysed the parameters of the papillation assay including the strength of the transposase transcriptional and translational signals. To overcome certain limitations of inducible promoters, we constructed a set of vectors based on constitutive promoters of different strengths to widen the range of transposase expression. We characterized and validated our expression vectors with Hsmar1, a member of the *mariner* transposon family. The highest rate of transposition was observed with the weakest promoters. We then took advantage of our approach to investigate how the level of transposition responds to selected point mutations and the effect of joining the transposase monomers into a single-chain dimer.

**Conclusions:**

We generated a set of vectors to provide a wide range of transposase expression which will be useful for screening libraries of transposase mutants. The use of weak promoters should allow screening for truly hyperactive transposases rather than those that are simply resistant to auto-regulatory mechanisms, such as overproduction inhibition (OPI). We also found that mutations in the Hsmar1 dimer interface provide resistance to OPI in bacteria, which could be valuable for improving bacterial transposon mutagenesis techniques.

## Background

Transposable elements (TEs) are DNA sequences with the ability to move from one place to another in the genome. They are found in virtually all organisms and are particularly numerous in higher eukaryotes where they can represent a significant percentage of the genome [1–3]. Originally thought of as selfish elements that provide no advantage to the host, TEs have now been shown to be important drivers of genome evolution [4, 5]. Indeed, TEs can provide novel transcription factor binding sites, promoters, exons or poly(A) sites and can also be co-opted as microRNAs or long intergenic RNAs [6–8]. TEs are a diverse group of DNA sequences using a wide range of mechanisms to transpose within their hosts. One particular mechanism prevalent in eukaryotes, and used by the *mariner* family, is known as “cut-and-paste” transposition [9]. Over the past several years, our group and others have described the mechanisms regulating the transposition rate of different *mariner* transposons, such as Himar1, Hsmar1 or Mos1 [10–15]. In Hsmar1, a regulatory mechanism was first recognized because of the phenomenon of overproduction inhibition (OPI) [16]. The mechanism of OPI was eventually explained by the realization that double occupancy of the transposon ends with transposase dimers blocks assembly of the transpososome [12]. Thus, OPI curbs Hsmar1 transposition rate to avoid damaging the host genome by excessive transposition [12]. This mechanism will apply to any transposon in which a transposase multimer binds one transposon end and then recruits the second end as naked DNA.

OPI represents a limitation in the development of hyperactive transposases for biotechnological applications. Several approaches such as modifying the binding kinetics of the transposase to the inverted terminal repeat (ITR) or the monomer-dimer equilibrium can be used to overcome OPI. Indeed, we and others previously showed that most mutations in the conserved WVPHEL motif, in Himar1 and Hsmar1, result in hyperactive transposases but at the cost of producing non-productive DNA double-strand breaks and therefore DNA damage [17, 18].

**Fig. 1:**
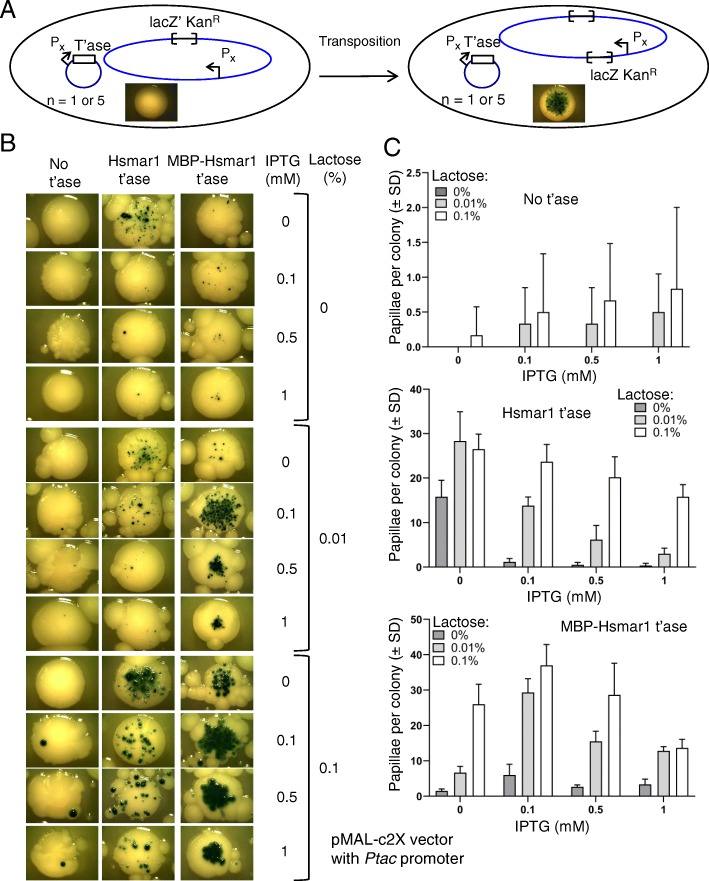
Characterization of the papillation assay using a strong inducible promoter. **a**. The Hsmar1 transposon (RC5096), which encodes a *lacZ* gene lacking transcription and translation signals and a kanamycin resistance marker (kanR), has been integrated in a non-transcribed region of a lac- *E. coli* strain. In absence of a vector encoding the transposase, the *lacZ* gene cannot be transposed in frame into an active open reading frame. The strain remains lac- and produces white colonies on plates containing X-gal. In presence of the transposase, the transposon can integrate in frame into the ORF of a transcribed gene, producing a lacZ fusion protein. The cell’s descendants will express lacZ and will appear as blue papillae on plates containing X-gal. Black arrow, promoter; open brackets, transposon ends; empty rectangle, transposase gene. For the mating-out assay, a chloramphenicol resistant derivative of the conjugative plasmid pOX38 is introduced into the reporter strain. Transposition of the kanR-marked transposon into the plasmid is detected by selecting transconjugants after mating with a recipient strain on chloramphenicol and kanamycin. **b**. An expression vector encoding no transposase (pMAL-c2X), Hsmar1 (pRC1721) or MBP-Hsmar1 (pRC880) transposase (t’ase) was transformed into the papillation strain and plated on different lactose and IPTG concentrations. Representative colonies of the papillation plates are shown. On some pictures, smaller colonies surrounding the main colony are visible. These satellite colonies appear only after several days of incubation when the ampicillin present on the plate has been degraded. They can be ignored because they do not contain any transposase expression plasmid. Part of this figure was previously published in [19] under the terms of the Creative Commons CC BY license. **c**. Quantification of the number of papillae per colony from single colonies. Average ± standard deviation of six representative colonies from the same biological replicate

To facilitate the isolation of suitable transposase mutants, the papillation assay was developed as an efficient screening procedure (Fig. 1a) [20, 21]. This assay is based on a *lacZ* gene, which lacks transcription and translation initiation signals, flanked by transposon ends. This reporter is integrated in a non-transcribed region of the genome of *Escherichia coli*. The transposase gene is provided *in trans* on a plasmid to simplify mutagenesis and library handling. For a *lacZ* gene fusion protein to arise, the transposon must insert in the correct orientation and reading frame, i.e. one in six insertions of the insertions into an active transcribed and translated protein-coding gene. When this happens within a colony growing on an X-gal indicator plate, it converts the cell and its descendants to a lac+ phenotype, which allows the outgrowth of blue microcolonies (papillae) on an otherwise white colony. The transposition rate is estimated by the rate of papillae appearance whereas the number of papillae per colony provides the level of transposition.

A limitation of the papillation assay is that it generally employs a transposase gene whose expression is under the control of an inducible promoter which cannot be finely regulated. We have constructed a set of vectors maintained at single copy or at ~ 13 copies per cell that carry various constitutive promoters in the absence or presence of a ribosome binding site (RBS). This set of vectors allows transposase expression across a wide range of expression levels facilitating the screening of hyperactive and/or OPI-resistant transposases. We used this set of vectors to compare an Hsmar1 transposase monomer to a single-chain dimer and to test for hyperactivity and OPI-resistance in several Hsmar1 transposase mutants. We found that one Hsmar1 mutant in the dimer interface, R141L, is resistant to OPI in *E. coli*.

## Results and discussion

### Characterization of the papillation assay using a strong inducible promoter

The papillation assay provides a visual assessment of the transposition rate, which is dependent on the concentration and activity of the transposase [12, 20]. We defined the level of transposition as the average number of papillae per colony after five days of incubation at 37 °C. In the previous papillation assay, the transposase was provided by the protein expression vector pMAL-c2x under the control of a Ptac promoter and was fused to the C-terminus of the maltose binding protein [18]. We first characterized the papillation assay using the Hsmar1 transposase cloned downstream of the inducible Ptac promoter and investigated the effect of different concentrations of IPTG and lactose on the transposition rate (Fig. 1b and c). The Ptac promoter can be induced by IPTG or by allolactose, which is metabolized from lactose by lacZ [22]. Therefore, lactose will not induce the Ptac promoter until a successful transposition event that leads to a lacZ+ cell occurred. Also, we investigated whether the presence of the MBP-tag affects the transposition rate (Fig. 1b and c). In the absence of transposase, the number of papillae per colony in all the conditions tested was between zero and three (Fig. 1b, no transposase column, and 1C). In presence of the transposase or MBP-transposase (middle and right columns, respectively), the number of papillae per colony varies with the concentration of IPTG and lactose.

Independently of the presence or absence of the MBP tag and the IPTG concentration, the number of papillae increases with the concentration of lactose (Fig. 1b and c). Lactose improves the sensitivity of the assay by allowing papillae to continue to grow when the other carbon sources are exhausted. One explanation could be the induction of the Ptac promoter by lactose. However, since the strain is lacZ- the lactose cannot be metabolized to allolactose, the inducer of the lac operator in the Ptac promoter [22]. Another explanation is that the lac+ cells form larger, more clearly visible, papillae because they are able to continue growing after the lac- cells have exhausted the carbon source in the LB agar. We confirm later (see below) that lactose does not influence the transposition rate but instead allows the late transposition events to become visible, explaining the positive correlation between the number of papillae and the lactose concentration seen here (Fig. 1b and c). We also note that at all lactose concentrations, the number of papillae was highest for the native transposase at 0 mM IPTG, while that for the MBP-fusion was highest at 0.1 mM IPTG (Fig. 1b and c). A more quantitative mating-out assay [20] confirmed the results from the papillation assay that the native transposase gave a higher transposition rate than the MBP-fusion in the absence of lactose and IPTG (Table 1).

**Table 1:** Transposition frequencies of MBP-tagged or untagged Hsmar1 transposase

Construct	Transposition frequency
Ptac MBP-Hsmar1 transposase	1.99 (±0.43) × 10^−6^
Ptac Hsmar1 transposase	2.58 (±0.02) × 10^− 5^

The bacterial mating-out assays have been performed with the RC5097 strain with the pRC880 or pRC1721 vectors and in absence of lactose and IPTG. Transposition frequencies are the average of three independent experiments ± standard error of the mean

Any further increase in the IPTG concentration results in a decrease of the transposition rate, consistent with the effects of OPI, which has been described for Hsmar1 in vitro, in *E. coli*, and in HeLa cells [12, 19]. Interestingly, the presence of the MBP tag affects the transposition rate of the transposase, potentially through its stabilization. We therefore decided to use untagged Hsmar1 transposase for the remaining experiments.

### SETMAR transposition activity was lost during the same period as Hsmar1 transposase domestication

**Fig. 2:**
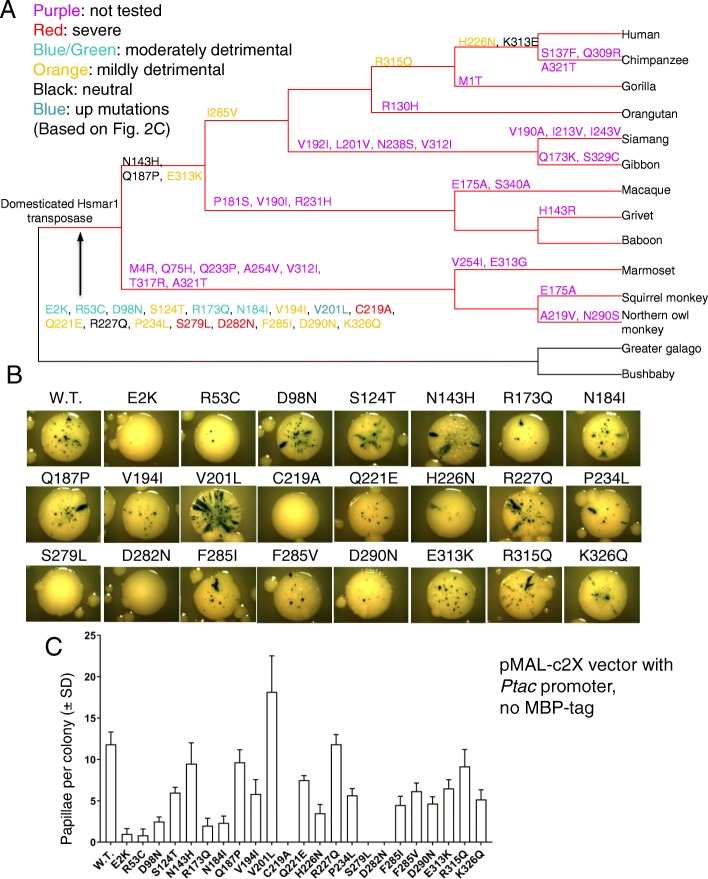
SETMAR transposition activity was lost during the same period as Hsmar1 transposase domestication. **a**. Phylogenetic tree of anthropoid primates which represents the emergence of mutations in the Hsmar1 domain of SETMAR. All the mutations present in the human SETMAR were tested by papillation assay to determine their effects on Hsmar1 transposition. The colour code used for the effect of the mutation on the number of papillae per colony is based on Fig. 2c. **b**. Representative colonies of pMAL-C2X expressing wild-type (pRC1721) or mutant Hsmar1 transposases (pRC1877–1899). The papillation assays were performed in presence of 0.01% lactose and no IPTG. **c**. Quantification of the number of papillae per colony from single colonies. Average ± standard deviation of six representative colonies from the same biological replicate

The Hsmar1 transposase was originally discovered in the human genome where an inactivated Hsmar1 transposase is fused to a SET domain to form the *SETMAR* gene [23–25]. The domesticated Hsmar1 transposase is inefficient at performing transposition because of the mutation of the DDD triad catalytic motif to DDN [24, 25]. In vitro, the domesticated Hsmar1 transposase (DDN mutant) was found to be largely defective for transposition [24]. In a papillation assay, no papillae were observed with the domesticated Hsmar1 transposase (SETMAR exon 3), which indicates that it is totally defective for transposition in vivo (Additional file 1: Figure S1). Relative to the reactivated Hsmar1 transposase, which we presume to be the ancestral sequence, the human SETMAR protein contains 21 amino acid changes. We took advantage of our papillation assay to investigate the effect on transposition of these 21 changes and two other mutations that occurred in the human lineage (F285I and E313K). These changes were made as single mutants in the reactivated “wild-type” transposase with the Ptac promoter and tested in our papillation assay using 0.1% lactose without IPTG (Fig. 2a-c) [25]. Most of the 23 mutations present in the human SETMAR are in the transposase catalytic domain and are common to all anthropoid primates containing SETMAR, indicating that these mutations likely occurred before or during the domestication event. In addition to D282N, two other mutations, C219A and S279 L, completely disrupt Hsmar1 transposition activity (Fig. 2b and c). Two other mutations located in the first helix-turn-helix (HTH) ITR recognition domain of the transposase DNA binding domain, E2K and R53C, also severely affect the transposition rate. The E2K mutation is located upstream of the first helix whereas the R53C is found in the third helix, based on the Mos1 paired-end complex (PEC) structure [26]. None of these two residues directly interact with DNA, at least in the PEC structure [26]. In addition, seven other mutations located mostly in the transposase catalytic domain mildly affect Hsmar1 transposition activity. Only one mutation, V201 L, increases Hsmar1 transposition rate whereas the remaining mutations were neutral.

This result supports an absence of conservation of Hsmar1 transposase activity during SETMAR evolution, in agreement with recent studies which did not observe an in vivo nuclease activity of SETMAR in DNA repair assays [27, 28]. Two of the DNA binding mutants, E2K and R53C, are deleterious to Hsmar1 transposition activity in a papillation assay. It will be interesting to determine whether this effect is mediated through a change in ITR binding efficiency, which could have modified SETMAR’s ability to bind ITRs in the genome and therefore its emerging functions in regulating gene expression [29].

### Papillation assay with a featureless DNA constitutive promoter

**Fig. 3:**
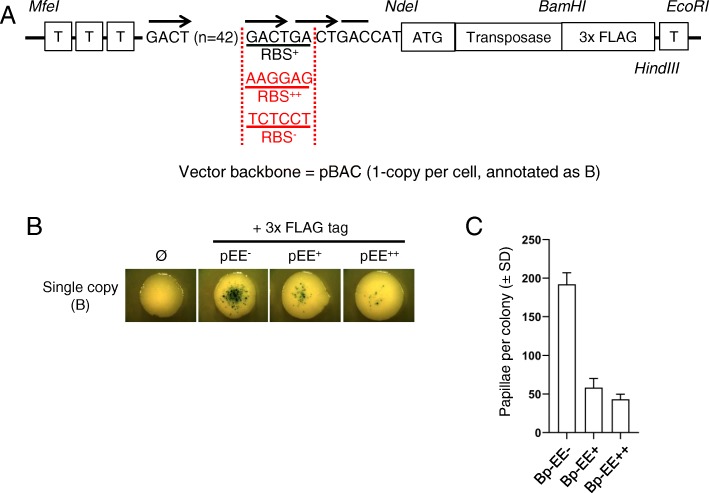
Papillation assay with a featureless DNA constitutive promoter. **a**. The Hsmar1 gene is fused to 3x FLAG-tag on its C-terminus and cloned downstream of pEE containing a ribosome binding site (RBS) based on the GACT repeat (RBS+), on an optimal RBS sequence (RBS++), or on an inactive RBS sequence (RBS-). The construct is located between terminator sequences (T) upstream and downstream to avoid read-through transcription. The plasmid backbone is a single-copy vector, pBACe3.6. **b**. Representative colonies of each single-copy vector expressing a wild-type FLAG-tagged Hsmar1 transposase under the control of pEE with three different RBSs (0 = no transposase/vector only control; pRC1821, 1833 and 1845, negative control: pRC1806). **c**. Quantification of the number of papillae per colony from single colonies. Average ± standard deviation of six representative colonies from the same biological replicate

We wondered if the expression level of the un-tagged transposase at 0 mM IPTG and 0.1% lactose (Fig. 1) represents the peak activity of the system or is the system already in OPI? To answer this question, we took advantage of a 44 GACT repeats sequence that represents an idealized segment of unbent, featureless DNA. It is known as the “even end” (EE) as it was first used to study the role of DNA bending in Tn10 transposition [30]. We reasoned that this would provide for a minimal level of transcription owing to its lack of TA and AT dinucleotides which feature in the − 10 region of sigma70 promoters (TATAAT, see flow cytometry GFP data below). Although the EE does not provide a − 10 region, it provides a G + A rich sequence that might act as a ribosome binding site, referred as RBS^+^ in this study (Fig. 3a, RBS^+^). We therefore abolished or optimized with a RBS from the bacteriophage T7 this putative RBS (Fig. 3a, RBS^−^ and RBS^++^, respectively) [31]. We find that transposition is the highest in the absence of a RBS (Fig. 3b and c), supporting the presence of a RBS activity with the Bp-EE+ vector.

The EE- promoter-UTR sequence is not necessarily the highest level of transposition attainable as EE+ and EE++ might already be in OPI because of the higher translation efficiency. We therefore explored transcriptional activity with a series of progressively degraded P_L_-λ promoters that had been selected from a mutant library for their lack of stochastic cell-to-cell variation [32].

### Characterization of the set of constitutive promoters

We synthesized a set of five constitutive promoters derived from the constitutive bacteriophage P_L_Tet-O1 promoter, ((OO, JJ, K, E, and P_L_Tet-O1 in [32]) (Table 2). The alignment of the promoters and the locations of important DNA sequences are shown in Additional file 1: Figure S2 [31, 33].

**Table 2:** List of constitutive promoters

Promoter name	Relative mRNA	Average promoter metric
pEE	n.d.	n. d.
p2 (OO)	0.003	0
p3 (JJ)	0.159	0.123911
p4 (K)	0.299	0.274103
p5 (E)	0.743	0.83686
p6 (P_L_Tet-O1)	1	0.868513

The promoter name inside the brackets, the relative mRNA and average promoter metric value are from [32]. n. d. not determined

**Fig. 4:**
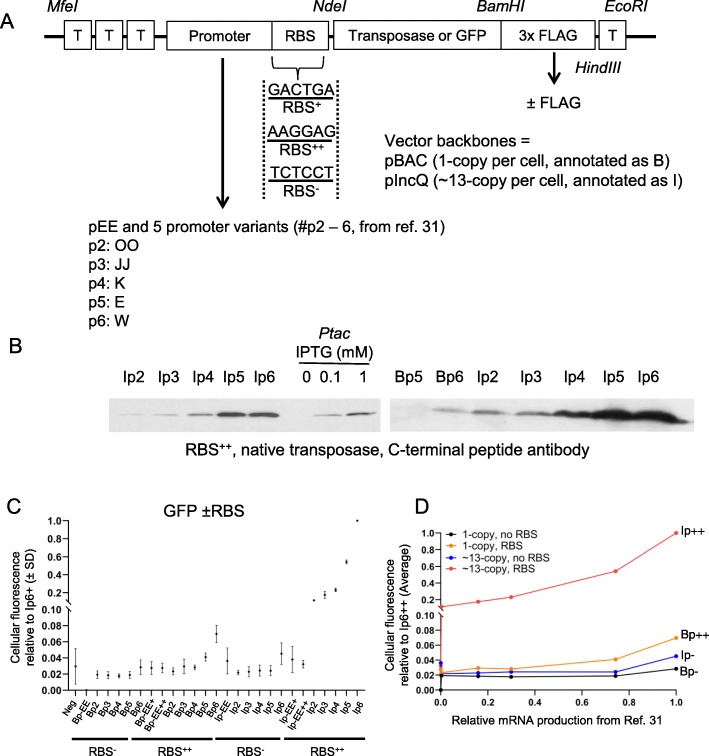
Characterization of the set of constitutive promoters. **a**. The *Hsmar1* gene is fused or not to 3x FLAG-tag on its C-terminus and cloned downstream of one of six different promoters (see text for more details) with an inactive or optimal RBS (defined in Fig. 2a). The construct is located between terminator sequences (T) upstream and downstream to avoid read-through transcription. To further control the number of copies, the plasmid backbone is a one-copy, pBACe3.6 (**b**), or a ~ 13-copy, pGMH491 (pIncQ, I), vector. **b**. Western blots using an antibody against the C-terminus of SETMAR, which corresponds to the domesticated Hsmar1, to compare the strongest promoters with an optimal RBS to the Ptac promoter induced with different concentration of IPTG. **c**. The promoter strength of each construct was determined by flow cytometry after cloning an *EGFP* gene in each vector (pRC1782–1807). The number EE to 6 corresponds to one of the six promoters. The single and ~ 13-copy vectors are annotated B or I, respectively. The vectors with an inactive or an optimal RBS are annotated – or ++, respectively. The fluorescence data were normalized to the strongest promoter, Ip6++. Average of the geometric mean ± standard deviation of two biological replicates, except for Bp-EE- where there is only replicate. Neg: negative control, Ip0 (empty vector). **d**. Plot of the relative mRNA production (as defined in [32]) versus the promoter strength determined by flow cytometry in Fig. 3c. The relative mRNA production of pEE was arbitrary defined as ten times less than p2

To increase the available range of expression levels, we also created by PCR a variant of each promoter where the RBS has been abolished (Fig. 4a). The expression construct is shown in Fig. 4a and is composed of the promoter and a RBS sequence, *NdeI* and *BamHI* restriction sites facilitate the cloning of a gene of interest, which can then be fused (RBS^−^ and RBS^++^) or not (RBS^++^ only) to a C-terminal 3x FLAG tag. The C-terminal tag was added to allow the study of proteins that do not have available antibodies. To avoid any read-through transcription, the construct is flanked by terminator sequences. The whole construct is delimited by *MfeI* and *EcoRI* restriction sites. The expression constructs were cloned either into a single-copy vector or a ~ 13-copy vector, pBACe3.6 (B) and pGHM491 (pIncQ, I), respectively [34, 35]. The following nomenclature will be used: Bp-EE to Bp6 represents the six promoters cloned into the single copy vector, Ip-EE to Ip6 corresponds to the six promoters cloned into the ~ 13-copy vector, the ‘-‘ and ‘++’ represents the abolished or the optimized RBS, respectively.

We first investigated the strongest non-FLAG-tagged expression vectors by performing western blots with an anti-Hsmar1 antibody (Fig. 4b). We also compared by western blotting these constructs with the Ptac inducible promoter previously used for papillation assay (Fig. 4b). Interestingly, two of our constructs (Ip5++ and Ip6++) produce a higher amount of Hsmar1 transposase than the Ptac promoter fully induced with 1 mM of IPTG.

We next quantified the strength of each expression vector by inserting an *EGFP* gene in each FLAG-tagged vector to investigate fluorescence levels by flow cytometry (Additional file 1: Figure S3). To determine the strength of the expression vectors, we normalized their geometric mean fluorescence value against the strongest vector, Ip6++ (Fig. 4c). Most of the single-copy expression vectors and the RBS- promoters produce an amount of EGFP fluorescence close to the background level. However, all of the ~ 13-copy expression vectors with a consensus RBS produce more fluorescence than their respective single-copy vectors. A comparison of the EGFP produced by the p5 and p6 promoters shows that the pIncQ expression vectors produced around 14 times more fluorescence than the pBAC expression vectors, indicating a potential copy number of 14 for pIncQ, which is in line with the literature [35]. Also, the vectors with a consensus RBS produce an amount of fluorescence correlating with the promoter strength originally determined by Alper and colleagues [32]. In contrast, all of the vectors without a RBS motif, except Ip6-, produce a fluorescence level close to the detection threshold (Fig. 4d). Similarly, the pEE promoter is also too weak to change the amount of fluorescence produced whether the RBS is present or absent.

### Characterization of the papillation assay with the wild-type Hsmar1 transposase

Prior to the determination of the rate of transposition of each expression vector with the papillation assay, we visually determined the best conditions for this assay using the Ip3++ expression vector, which has a strength similar to Ptac induced with 0.1 mM IPTG (Fig. 4b) and will therefore have a limited number of papillae per colony, and a range of lactose concentrations (Additional file 1: Fig. S4). Similarly to the Ptac promoter, we observed a correlation between the number of papillae per colony and the lactose concentration (Additional file 1: Figure S4A and B). We decided to work at 0.1% lactose since it represents the best trade-off between the number of papillae per colony and the size of the papillae for quantitation at high transposition rate. To confirm that it is a lactose-specific effect, we performed papillation assays with the Bp2++ expression vector and a 0.1% concentration of different sugars: no sugar, glucose, maltose, lactose, and maltose plus lactose (Additional file 1: Figure S5). Importantly, the activity of the constitutive expression vectors is independent of the absence or presence of sugars. A higher number of papillae was only observed when lactose was added, indicating that lactose allows the late transposition events to become visible as only lacZ+ cells are able to metabolize it.

**Fig. 5:**
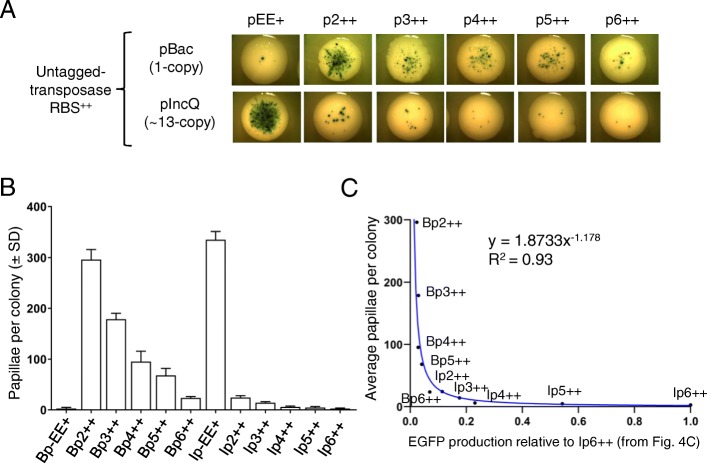
Characterization of the papillation assay with the wild-type untagged Hsmar1 transposase and optimal RBS. **a**. Representative colonies of each vector expressing a wild-type untagged Hsmar1 transposase (pRC1723–1728 and pRC1730–1735). **b**. Quantification of the number of papillae per colony from single colonies. Average ± standard deviation of six representative colonies from the same biological replicate. **c**. Plot of the EGFP production relative to Ip6++, determined in Fig. 4c, versus the average number of papillae per colony (as defined in Fig. 5b). As expected from overproduction inhibition (OPI), the promoter strength is inversely related to the level of transposition

The Hsmar1 transposition rate is inversely related to the transposase expression because of OPI [12, 19]. To determine whether we observe a similar relationship with our constitutive promoters, we first investigated the transposition rate supported by each RBS^++^ expression vector with the untagged wild-type transposase (Fig. 5a). As expected from the wide range of expression, we observed a 350-fold variation in the average number of papillae per colony (Fig. 5b). To better visualize the relationship between the expression vector strength and the level of transposition, as determined by the number of papillae per colony, we plotted the strength of the promoter as determined by our EGFP measurements against the number of papillae per colony (Fig. 5c). As previously documented in vitro, in *E. coli* and in HeLa cells, the wild-type Hsmar1 transposase expression is inversely related to the transposition rate for Bp++ and Ip++ vectors [12, 19].

**Fig. 6:**
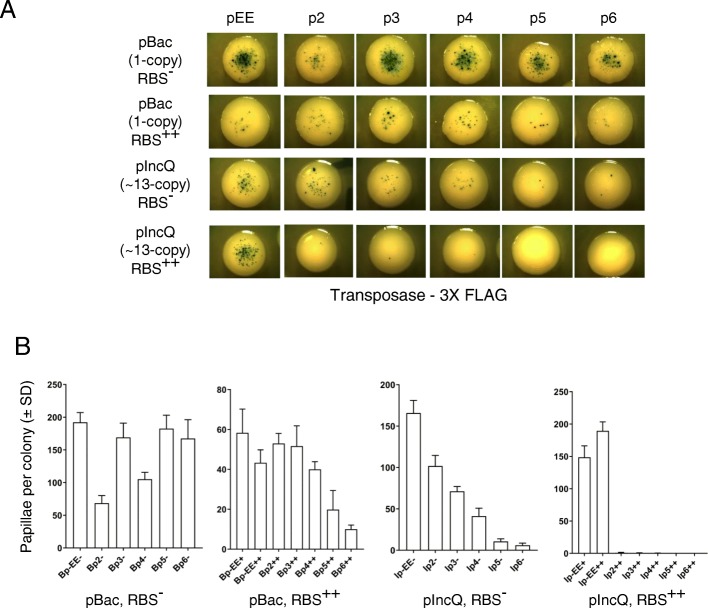
Characterization of the papillation assay with the wild-type FLAG-tagged Hsmar1 transposase and an optimal or inactive RBS. **a**. Representative colonies of each vector expressing a wild-type FLAG-tagged Hsmar1 transposase (pRC1821–1846). **b**. Quantification of the number of papillae per colony from single colonies. Average ± standard deviation of six representative colonies from the same biological replicate

There was a noticeable difference in the level of transposition between pBac and pIncQ vectors (Fig. 5c). To determine whether we could obtain a wider range of transposase expression, we tested the 3x FLAG tag expression vectors with or without a RBS (Fig. 6a). Quantitation of the level of transposition of each expression vector shows that the Bp++, Ip-, and Ip++ series follow an inverse relationship between transposase expression and transposition rate (Fig. 6b). However, the set of Bp- expression vectors is more difficult to interpret because of the single copy of the expression vector. This may be smoothed out in the Ip- series, which gave the most progressive response.

Similarly to the effect of the MBP-tag on transposition (Fig. 1), the presence of the 3x FLAG-tag also modifies the level of transposition (compare the RBS++ expression vectors from Figs. 5 and 6). However, it remains unclear how the presence of a tag affects the number of papillae per colony but it could be mediated by a change in transposase stability.

### Covalently linking two Hsmar1 monomers in a dimer affects the transposition rate

**Fig. 7:**
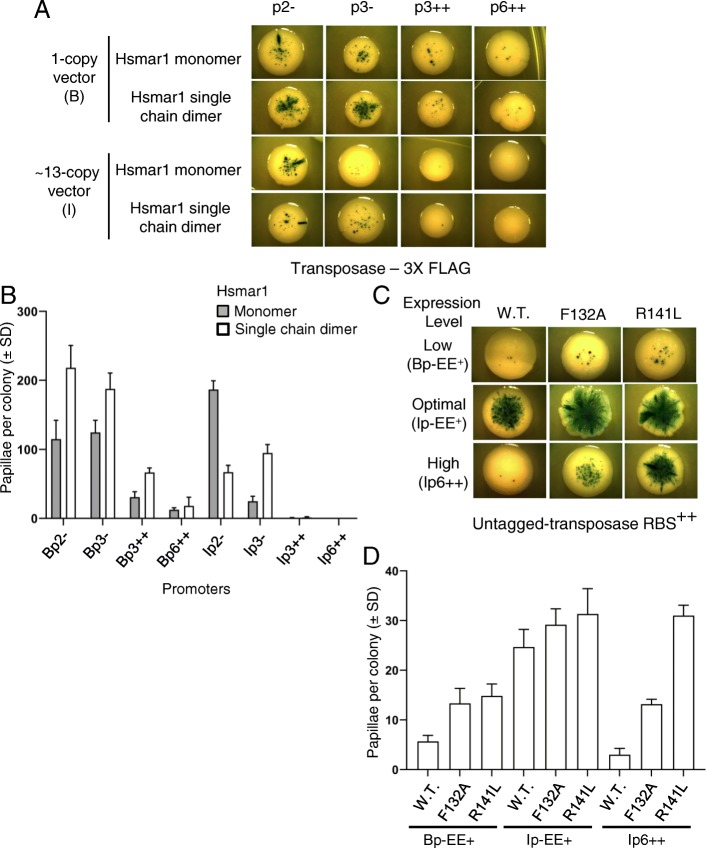
Covalently linking two Hsmar1 monomers in a dimer or mutating Hsmar1 dimer interface affect the transposition rate. **a**. Representative colonies of each expression vector expressing either Hsmar1 monomer (pRC1868–1871, 1873, 1875, and 1876) or Hsmar1 single chain dimer (pRC1858–1861, 1863, 1865, and 1866). **b**. Quantification of the number of papillae per colony from single colonies. Average ± standard deviation of six representative colonies from the same biological replicate. **c**. Different Hsmar1 mutants have been tested in low, optimal and high transposase expression level (Bp1+ (pRC1739 and 1740), Ip1+ (pRC1746 and 1747) and Ip6++ (pRC1752 and 1753), respectively). Representative colonies of each papillation plate is shown. **d**. Quantification of the number of papillae per colony from single colonies. Average ± standard deviation of six representative colonies from the same biological replicate

We recently described a novel Hsmar1 transposase construct where two monomers are covalently bound by a linker region [36]. We took advantage of our approach to test whether the transposition rate of a single chain dimer of Hsmar1 transposase differs from that of the monomer. At low expression levels, we expect a single chain dimer to transpose more efficiently than a monomer because of the physical link between the subunits, which favours dimerization and also requires only a single translation event. We cloned the monomeric and dimeric construct in a set of expression vectors spanning very low to high expression and performed a papillation assay (Fig. 7a). With the exception of Ip2-, we observe for the weakest expression vectors a higher number of papillae per colony for the single chain dimer, as shown by the quantitation of Bp2-, Bp3-, and Ip3- in Fig. 7b.

When compared to the results obtained with the Hsmar1 monomer, the single chain dimer transposition rate peaks at a different set of expression vectors, Bp2- and Bp3- for the covalent dimer and Ip2- for the monomer (Fig. 7b). This might indicate that Bp2- and Bp3- are weaker expression vectors than Ip2-. We do not observe any difference in the number of papillae per colony with stronger expression vectors such as Ip3++ and Ip6++ (Fig. 7a and b). This indicates that a single chain Hsmar1 dimer is as sensitive to OPI as the Hsmar1 monomer.

### Mutations in Hsmar1 dimer interface produce hyperactive mutants in bacteria

Transposable elements are useful for genetic screens and gene delivery applications [37]. However, OPI limits the transposition rate when the transposase concentration is too high [12]. One way to overcome OPI is to decrease the stability of the Hsmar1 dimer to shift the monomer-dimer equilibrium to the inactive monomeric form. We decided to take advantage of our approach to investigate two Hsmar1 transposases mutated in the dimer interface, one known mutant, F132A (F460 in SETMAR [38]), and R141L, which was identified in a screen for hyperactive transposases [9]. Both F132 and R141 are found in the dimer interface in the crystal structure of the Hsmar1 catalytic domain, which suggests that this subunit interface could be present in one of the transposition intermediates [9, 38]. Also, mutation of the F460 residue to lysine in SETMAR catalytic domain abolishes its dimerization in vitro [38]. We used three vectors expressing untagged Hsmar1 transposase at a low (Bp-EE+), optimal (Ip-EE+), and high (Ip6++) expression level (Fig. 7c and d). Interestingly, both F132A and R141L transposases are hyperactive at low level of expression when compared to WT. A higher number of papillae is also observed at high expression level for both mutants, with R141L showing a stronger resistance to OPI than F132A. To confirm the results, the transposition rates were also determined using the more quantitative mating-out assay [20] (Table 3). The results of the mating-out and transposition assays were similar with a higher transposition rate at optimal and high expression levels. Interestingly, Hsmar1 R141L transposition rate is not affected by the high transposase expression level produced by Ip6++, as the rate remains similar between Ip-EE+ and Ip6++ whereas we observe a 147-fold and a 17-fold decrease for the wild type transposase and for the F132A mutant, respectively.

**Table 3:** Transposition frequencies of two Hsmar1 transposase mutants expressed at optimal and high level

Construct	Transposition frequency	Mutant/W.T.
Ip-EE+ W.T.	4.73 (±1.02) × 10^−5^	
Ip-EE+ F132A	9.73 (±4.53) × 10^−4^	21
Ip-EE+ R141L	2.42 (±1.68) × 10^− 4^	5
Ip6++ W.T.	3.22 (±1.02) × 10^− 7^	
Ip6++ F132A	5.79 (±2.63) × 10^−5^	180
Ip6++ R141L	3.24 (±1.43) × 10^−4^	1006

The bacterial mating-out assays have been done with the RC5097 strain and the Ip-EE+ or Ip6++ vectors. Transposition frequencies are the average of three independent experiments ± standard error of the mean

## Conclusion

This study provides a set of expression vectors based on constitutive promoters to investigate the phenotypes of mutant transposase. It will be useful to distinguish between true hyperactive mutants and defective mutants that happen to be resistant to OPI. Compared to inducible promoters, our set of expression vectors provides a wide range of consistent transposase expression levels between individual cells. In addition to the characterization of the constitutive promoters, we also found one Hsmar1 mutation, R141L, which is OPI-resistant in *E. coli* and could therefore prove useful for improving bacterial transposon mutagenesis with *mariner* elements. Another approach in controlling the transposition rate is to use a single chain Hsmar1 dimer, which allows transposition to occur after a single translation event and would therefore permit the usage of a weak promoter with a weak RBS.

We believe our set of expression vectors will be useful or the study of other transposons and in the screening of libraries for finding hyperactive and/or OPI-resistant transposases. For transposons other than Hsmar1, the expression will have to be tuned to the system as different transposons will have different relationship between transposase concentration and transposition rate. A medium copy vector (pIncQ) with a medium promoter (p4) would be an ideal starting point. The expression can then be tuned by progressive degradation of the RBS.

## Methods

### Media and bacterial strains

Bacteria were grown in Luria-Bertani (LB) media at 37 °C. The following antibiotics were used at the indicated concentrations: ampicillin (Amp), 100 μg/ml), chloramphenicol (Cm), 25 μg/ml, and spectinomycin (Spec), 100 μg/ml. The following *E. coli* strains were used: RC5024 (identical to DH5α) [endA1 hsdR17 glnV44 thi-1 recA1 gyrA relA1 Δ (lacIZYA-argF)U169 deoR (φ80dlac Δ (lacZ)M15)], RC5094 [F- araD139 Δ (argF-lac)U169 rspL150 relA1 flbB5301 fruA25 deoC1 ptsF25 rpoS359::Tn*10*], RC5096 [F^−^ fhuA2 Δ (lacZ)r1 glnV44 e14-(McrA-) trp-31 his-1 rpsL104 xyl-7 mtl-2 metB1 Δ (mcrC-mrr)114::*IS10* argE::Hsmar1-lacZ’-kanR] and RC5097 (= RC5096 pOX38::miniTn10-CAT).

### Constitutive promoters

Alper et al. previously generated and characterized a set of constitutive promoters based on P_L_-λ ranging from strong down to very weak [32]. We selected the promoters 00, jj, K, E, and P_L_Tet-O1 (equivalent to p2, p3, p4, p5, and p6 in this study, Additional file 1: Figure S2) and generated pEE, a featureless tract of 44 GACT repeats which we chose to represent as an ideal promoter-less region (Table 4). Each promoter sequence is preceded by three terminator sequences and followed by a consensus ribosome binding site (RBS++, from [32]), a null RBS (RBS-), or a GACT RBS in the case of pEE (RBS+), a transposase gene, three FLAG-tag and a terminator sequence (Figs. 2a and 3a). The different RBS sequences were inserted by a PCR step.

**Table 4:** List and DNA sequences of constitutive promoters

Promoter name	Sequence	Relative mRNA	Average promoter metric
pEE	CTGACTGACTGACTGACTGACTGACTGACTGACTGACTGACTGACTGACTGACTGACTGACTGACTGACTGACTGACTGACTGACTGACTGACTGACTGACTGACTGACTGACTGACTGACTGACTGACTGACTGACTGACTGACTGACTGACTGACTGACTGACTGACTGACTGACTGACCATATG	n.d.	n.d.
p2 (00)	CAATTCCGACGTCTAAGGAAACCATTATCATGACATCAACCTATAAAAATAGGCGTATCACGAGGCCCTCTCGTCTCCACCTCAAGCTCCCTATCTAGTGATAGCGATTGACATCCCTATCAGTGACGGAGATATTGAGCACATCAGCAGGACGCACTGACCACTTTAAGAAGGAGATATACATATG	0.003	0
p3 (JJ)	CAATTCCGACGTCTAAGAAACCATTATTATCATGACATTAACCTATAAAAATAGGCGTATCACGAGGCCCTTTCGTCTTCACCTCGAGTCCCTATCAGTGATAGAGATTGACCTCCCTATCAGTGATAGAGATACTGAGCACATCAGCAGGACGCACTGACCACTTTAAGAAGGAGATATACATATG	0.159	0.123911
p4 (K)	CAATTCCGACGTCTAAGAAACCATTATTATCATGACATTAACCTATAAAAATAGGCGTATCACGAGGCCCTCTCGTCTTCACCTCGAGTCCCTATCAGTGATAGGGATTGACATCCCTATCAGTGATAGAGACACTGGGCACATCAGCAGGACGCACTGACCACTTTAAGAAGGAGATATACATATG	0.299	0.274103
p5 (E)	CAATTCCGACGCCTAAGAAACCATTATTATCATGACATTAGCCTATAAAAATAGGCGTACCACGAGGCCCTTTCGTCTTCACCTCGAGTCCCTATCAGTGATAGAGATTGACACCCCTATCAGTGATAGAGATACTGAGCACATCAGCAGGACGCACTGACCACTTTAAGAAGGAGATATACATATG	0.743	0.83686
p6 (P_L_Tet-O1)	CAATTCCGACGTCTAAGAAACCATTATTATCATGACATTAACCTATAAAAATAGGCGTATCACGAGGCCCTTTCGTCTTCACCTCGAGTCCCTATCAGTGATAGAGATTGACATCCCTATCAGTGATAGAGATACTGAGCACATCAGCAGGACGCACTGACCACTTTAAGAAGGAGATATACATATG	1	0.868513

Nomenclature (the letters indicated between brackets), sequence, relative mRNA and average promoter metrics of the constitutive promoters used in this study are from [32]. n.d.: not determined

### Plasmids

Expression plasmids were built by cloning the *EGFP* or *Hsmar1* gene in pBACe3.6, pGHM491, and pMAL-c2X (New England Biolabs) between *NdeI* and *BamHI* restriction endonuclease sites. A list of the plasmids used in this study can be found in Additional file 2: Table S1. The DNA sequences of the vectors based on pBACe3.6 and pMAL-c2X can be found in Additional file 3: Table S2. The DNA sequence of pGHM491 is unknown and therefore the DNA sequences of the vectors based on it are absent from Additional file 3: Table S2. Plasmids pRC880 and pRC1721 encode the wild-type transposase in pMAL-c2X in presence and absence of the MBP tag, respectively (Fig. 1). Plasmids pRC1782–1807 encode EGFP downstream of pEE to p6, with RBS-, RBS+, and RBS++, in pBACe3.6 and pGHM491 (Fig. 4). Plasmids pRC1723–1728 and pRC1730–1735 encode untagged Hsmar1 downstream of pEE to p6, with RBS+ and RBS++, in pBACe3.6 and pGHM491 (Figs. 4 and 5). Plasmids pRC1821–1846 encode FLAG-tagged Hsmar1 downstream of pEE to p6, with RBS-, RBS+, and RBS++, in pBACe3.6 and pGHM491 (Figs. 3 and 6). Plasmids pRC1877 to pRC1899 are derived from pMAL-c2X and encode the different Hsmar1 mutants with the mutations found in SETMAR (Fig. 2). Plasmids pRC1858–1861, 1863, 1865, 1866, 1868–1871, 1873, 1875, and 1876 encode the Hsmar1 monomer and Hsmar1 single chain dimer in Bp2-, Bp3-, Bp3++, Bp6++, Ip2-, Ip3++, and Ip6++ (Fig. 7). Plasmids pRC1739, 1740, 1746, 1747, 1752, and 1753 encode Hsmar1 F132A and R141L mutants cloned into Bp-EE+, Ip-EE+, and Ip6++ (Fig. 7).

### Flow cytometry

RC5096 cells expressing EGFP were grown overnight at 37 °C in LB medium supplemented with chloramphenicol or spectinomycin. The cultures were diluted in a 1:1000 ratio in fresh LB medium complemented with antibiotics and grown to mid-log phase (OD_600_ ~ 0.5). The cells were pelleted at 6000 g for 5 min, washed in 1X PBS twice, and resuspended in 500 μl of 1X PBS. Flow cytometry analysis was performed on 100,000 cells with a Beckman Coulter Astrios EQ. The FlowJo software was used to analyse the data (gating of the EGFP positive cells and acquisition of the geometric mean and the number of GFP positive cells) and to construct the overlayed plots. The number of GFP positive cells can be found in Additional file 4: Table S3.

### Western blotting

Cells containing a derivative of pMAL-c2x were grown in LB supplemented with 100 μg/ml of ampicillin at 37 °C until an OD_600_ of ~ 0.5 and were then induced with the required concentration of IPTG for 2 h at 37 °C. Cells containing pBACe3.6 or pGHM491 derivatives were grown in LB supplemented with respectively 100 μg/ml of spectinomycin or 50 μg/ml of chloramphenicol at 37 °C for the same amount of time as the induced cells. Promoters’ expression was analysed by pelleting ~ 1.5 × 10^9^ cells. The samples were resuspended in SDS sample buffer, boiled for 5 min, and loaded on 10% SDS-PAGE gels. Proteins were transferred to PVDF membrane, probed with an anti-SETMAR antibody raised against the amino acids 658–671, which correspond to the domesticated Hsmar1 (goat polyclonal, 1:500 dilution, ab3823, Abcam) followed by a horseradish peroxidase-conjugated anti-goat secondary antibody (rabbit polyclonal, 1:5000 dilution, ab6741, Abcam). Proteins were visualized by using the ECL system (Promega) and Fuji medical X-ray film (Fujufilm).

### Papillation assay

The papillation assay and the reporter strain RC5096 have been described previously (Fig. 1a) [18]. Briefly, transposase expression vectors were transformed into the RC5096 strain. It is a lac^−^ *E. coli* strain encoding a transposon containing a promoter-less *lacZ* and a kanamycin resistance gene flanked with Hsmar1 ends, which has been integrated in a silent genomic locus. In the absence of transposase, the strain produces white colonies on X-gal indicator plates. When the transposase is supplied *in trans*, the integration of a transposon into the correct reading frame of an active gene will produce a lacZ fusion protein. The descendants of this cell will become visible as blue papillae on X-gal indicator plates. RC5096 transformants were plated on LB-agar medium supplemented with different concentrations of lactose (or other sugars), 40 μg/ml of X-gal and either 50 μg/ml of chloramphenicol or 100 μg/ml of spectinomycin. Plates were incubated 5 days at 37 °C and photographed. The transposition rate is determined by the number of papillae per colony. Papillation assays were performed in biological duplicates.

### Mating-out assay

A chloramphenicol resistant derivative of the conjugative plasmid pOX38 has been introduced in the RC5096 papillation strains to create the donor strains RC5097. Briefly, RC5097 transformants and the recipient strain, RC5094, were grown overnight in LB supplemented with antibiotics at 37 °C. The next day, respectively one and three volumes of RC5097 and RC5094 were centrifuged for 5 min at 6000x g. Each pellet was resuspended in 3 ml of fresh LB, pooled together, and incubated in a shaking water bath for 3 h at 37 °C. After the mating, the transposition events were detected by plating 200 μl of each culture on LB-agar medium supplemented with tetracycline and kanamycin. The number of transconjugants was obtained by plating a 10^− 5^ fold dilution of each culture on LB-agar medium supplemented with tetracycline and chloramphenicol. The plates were incubated overnight at 37 °C and the transposition rate determined the next day by dividing the number of kanamycin-resistant colonies by the number of chloramphenicol resistant colonies.

## Supplementary information

**Additional file 1: Figure S1**. SETMAR transposase domain is totally defective for transposition in vivo. **Figure S2**. Multiple sequence alignment of PLTetO1 and p2 to p6. **Figure S3**. FACS profiles of the vectors used in this study. **Figure S4**. Effect of lactose on the modified papillation assay. **Figure S5**. Effect of different sugars on the papillation assay.

**Additional file 2:** **Table S1.** List of plasmids used in this study.

**Additional file 3: Table S2.** DNA sequences of the plasmids used in this study.

**Additional file 4: Table S3.** Number of GFP positive cells in the flow cytometry experiments.

## Data Availability

All the materials mentioned and used in this work will be made available upon request.

## Abbreviations

EE: “Even-end” promoter
ITR: Inverted terminal repeat
OPI: Overproduction inhibition
RBS: Ribosome binding site
TE: Transposable element

## References

[1] Lander ES, Linton LM, Birren B, Nusbaum C, Zody MC, Baldwin J (2001). Initial sequencing and analysis of the human genome. Nature..

[2] Feschotte C, Pritham EJ (2007). DNA transposons and the evolution of eukaryotic genomes. Annu Rev Genet.

[3] Aziz RK, Breitbart M, Edwards RA (2010). Transposases are the most abundant, most ubiquitous genes in nature. Nucleic Acids Res.

[4] Orgel LE, Crick FH (1980). Selfish DNA: the ultimate parasite. Nature..

[5] Chuong EB, Elde NC, Feschotte C (2017). Regulatory activities of transposable elements: from conflicts to benefits. Nat Rev Genet.

[6] Piriyapongsa J, Jordan IK (2007). A family of human microRNA genes from miniature inverted-repeat transposable elements. PLoS One.

[7] Kapusta A, Kronenberg Z, Lynch VJ, Zhuo X, Ramsay L, Bourque G (2013). Transposable elements are major contributors to the origin, diversification, and regulation of vertebrate long noncoding RNAs. PLoS Genet.

[8] Jangam D, Feschotte C, Betran E (2017). Transposable element domestication as an adaptation to evolutionary conflicts. Trends Genet.

[9] Tellier M, Bouuaert CC, Chalmers R (2015). Mariner and the ITm superfamily of transposons. Microbiol Spectr.

[10] Claeys Bouuaert C, Chalmers R (2010). Transposition of the human Hsmar1 transposon: rate-limiting steps and the importance of the flanking TA dinucleotide in second strand cleavage. Nucleic Acids Res.

[11] Claeys Bouuaert C, Walker N, Liu D, Chalmers R (2014). Crosstalk between transposase subunits during cleavage of the mariner transposon. Nucleic Acids Res.

[12] Claeys Bouuaert C, Lipkow K, Andrews SS, Liu D, Chalmers R (2013). The autoregulation of a eukaryotic DNA transposon. Elife..

[13] Dawson A, Finnegan DJ (2003). Excision of the Drosophila mariner transposon Mos1. Comparison with bacterial transposition and V(D) J recombination. Mol Cell.

[14] Auge-Gouillou C, Brillet B, Hamelin MH, Bigot Y (2005). Assembly of the mariner Mos1 synaptic complex. Mol Cell Biol.

[15] Richardson JM, Dawson A, O'Hagan N, Taylor P, Finnegan DJ, Walkinshaw MD (2006). Mechanism of Mos1 transposition: insights from structural analysis. EMBO J.

[16] Lohe AR, Hartl DL (1996). Autoregulation of mariner transposase activity by overproduction and dominant-negative complementation. Mol Biol Evol.

[17] Lampe DJ (2010). Bacterial genetic methods to explore the biology of mariner transposons. Genetica..

[18] Liu D, Chalmers R (2014). Hyperactive mariner transposons are created by mutations that disrupt allosterism and increase the rate of transposon end synapsis. Nucleic Acids Res.

[19] Blundell-Hunter G, Tellier M, Chalmers R (2018). Transposase subunit architecture and its relationship to genome size and the rate of transposition in prokaryotes and eukaryotes. Nucleic Acids Res.

[20] Huisman O, Kleckner N (1987). A new generalizable test for detection of mutations affecting Tn10 transposition. Genetics..

[21] Pajunen MI, Rasila TS, Happonen LJ, Lamberg A, Haapa-Paananen S, Kiljunen S (2010). Universal platform for quantitative analysis of DNA transposition. Mob DNA.

[22] Wheatley RW, Lo S, Jancewicz LJ, Dugdale ML, Huber RE (2013). Structural explanation for allolactose (lac operon inducer) synthesis by lacZ beta-galactosidase and the evolutionary relationship between allolactose synthesis and the lac repressor. J Biol Chem.

[23] Cordaux R, Udit S, Batzer MA, Feschotte C (2006). Birth of a chimeric primate gene by capture of the transposase gene from a mobile element. Proc Natl Acad Sci U S A.

[24] Liu D, Bischerour J, Siddique A, Buisine N, Bigot Y, Chalmers R (2007). The human SETMAR protein preserves most of the activities of the ancestral Hsmar1 transposase. Mol Cell Biol.

[25] Miskey C, Papp B, Mates L, Sinzelle L, Keller H, Izsvak Z (2007). The ancient mariner sails again: transposition of the human Hsmar1 element by a reconstructed transposase and activities of the SETMAR protein on transposon ends. Mol Cell Biol.

[26] Richardson JM, Colloms SD, Finnegan DJ, Walkinshaw MD (2009). Molecular architecture of the Mos1 paired-end complex: the structural basis of DNA transposition in a eukaryote. Cell..

[27] Mohapatra S, Yannone SM, Lee SH, Hromas RA, Akopiants K, Menon V (2013). Trimming of damaged 3′ overhangs of DNA double-strand breaks by the Metnase and Artemis endonucleases. DNA Repair (Amst).

[28] Tellier M, Chalmers R (2019). The roles of the human SETMAR (Metnase) protein in illegitimate DNA recombination and non-homologous end joining repair. DNA Repair (Amst).

[29] Tellier M, Chalmers R (2019). Human SETMAR is a DNA sequence-specific histone-methylase with a broad effect on the transcriptome. Nucleic Acids Res.

[30] Sewitz S, Crellin P, Chalmers R (2003). The positive and negative regulation of Tn10 transposition by IHF is mediated by structurally asymmetric transposon arms. Nucleic Acids Res.

[31] Olins PO, Rangwala SH (1989). A novel sequence element derived from bacteriophage T7 mRNA acts as an enhancer of translation of the lacZ gene in Escherichia coli. J Biol Chem.

[32] Alper H, Fischer C, Nevoigt E, Stephanopoulos G (2005). Tuning genetic control through promoter engineering. Proc Natl Acad Sci U S A.

[33] Lutz R, Bujard H (1997). Independent and tight regulation of transcriptional units in Escherichia coli via the LacR/O, the TetR/O and AraC/I1-I2 regulatory elements. Nucleic Acids Res.

[34] Frengen Eirik, Weichenhan Dieter, Zhao Baohui, Osoegawa Kazutoyo, van Geel Michel, de Jong Pieter J. (1999). A Modular, Positive Selection Bacterial Artificial Chromosome Vector with Multiple Cloning Sites. Genomics.

[35] Rawlings DE, Tietze E (2001). Comparative biology of IncQ and IncQ-like plasmids. Microbiol Mol Biol Rev.

[36] Claeys Bouuaert C, Chalmers R (2017). A single active site in the mariner transposase cleaves DNA strands of opposite polarity. Nucleic Acids Res.

[37] Mates L, Chuah MK, Belay E, Jerchow B, Manoj N, Acosta-Sanchez A (2009). Molecular evolution of a novel hyperactive sleeping beauty transposase enables robust stable gene transfer in vertebrates. Nat Genet.

[38] Goodwin KD, He H, Imasaki T, Lee SH, Georgiadis MM (2010). Crystal structure of the human Hsmar1-derived transposase domain in the DNA repair enzyme Metnase. Biochemistry..

