# Supplementary material for: Compensating for over-production inhibition of the Hsmar1 transposon in Escherichia coli using a series of constitutive promoters
Source: Mob DNA. 2020 Jan 10;11:5. doi: 10.1186/s13100-020-0200-5 (PMC6954556; doi:10.1186/s13100-020-0200-5)
Supplement: Supplementary file 1 — Additional file 1: Figure S1. SETMAR transposase domain is totally defective for transposition in vivo. Figure S2. Multiple sequence alignment of PLTetO1 and p2 to p6. Figure S3. FACS profiles of the vectors used in this study. Figure S4. Effect of lactose on the modified papillation assay. Figure S5. Effect of different sugars on the papillation assay. [file 13100_2020_200_MOESM1_ESM.pdf]

Empty vector

SETMAR Exon 3 (W.T.)

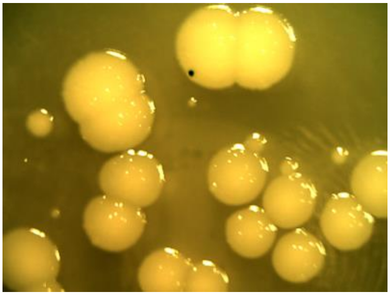

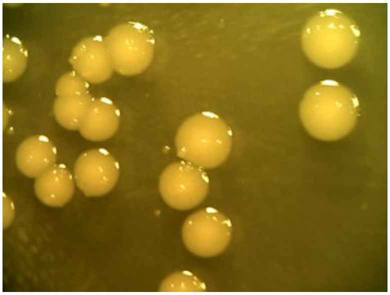

pMAL-c2X vector with
Ptac promoter,
No MBP-tag,
No IPTG, lactose 0.01%.

SF1. SETMAR transposase domain is totally defective for transposition *in vivo*.

Papillation assay performed with an empty vector control or wild-type SETMAR exon 3 (Hsmar1 transposase domain, pRC802 from (24)). Representative field of view of the papillation plates are shown.

Supplementary Figure 1

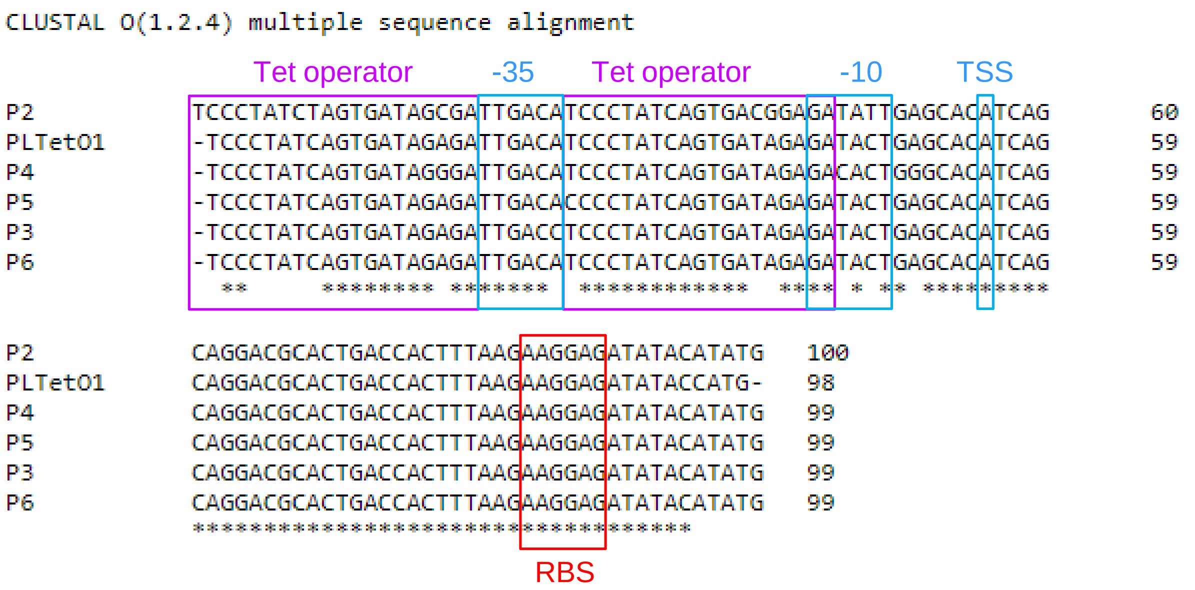

SF2. Multiple sequence alignment of PLTetO1 and p2 to p6.

The multiple alignment was performed with Clustal Omega. The locations of the Tet operators, -35, -10, TSS, and RBS are indicated by boxes and taken from ref. 30 and 32.

Cell sorter assay for amount of EGFP produced from the respective plasmids

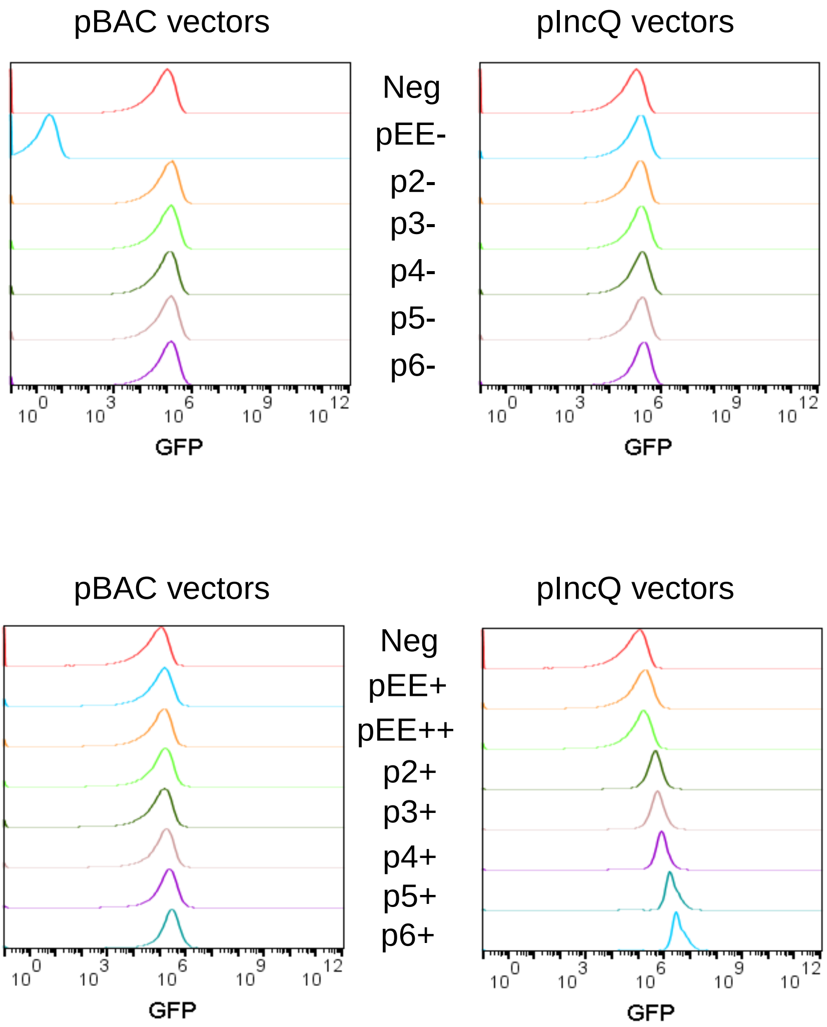

SF3. FACS profiles of the vectors used in this study

Example of FACS profile for each vector expressing the *eGFP* gene. Neg corresponds to Ip0, a negative control (empty vector).

A

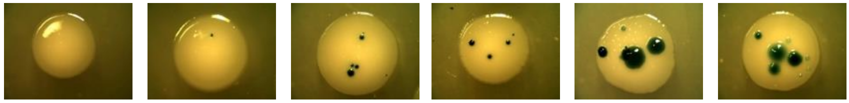

B

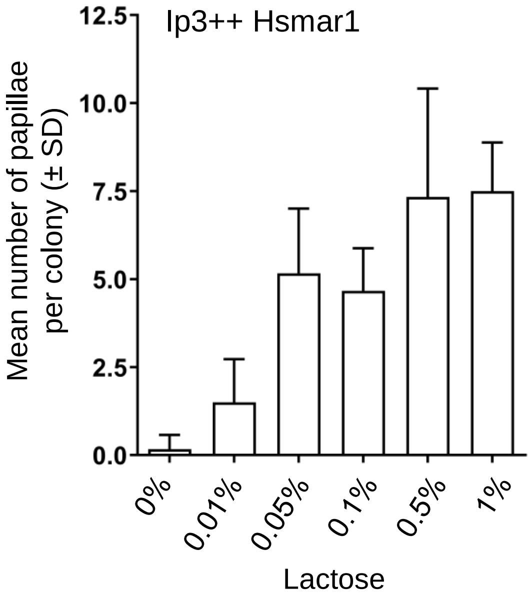

SF4. Effect of lactose on the modified papillation assay.

A/ Representative colony of the Ip3++ untagged Hsmar1 vector on different concentration of lactose.

B/ Quantification of the number of papillae per colony based on pictures of single colonies. Average ± standard deviation of six representative colonies from the same biological replicate.

Supplementary Figure 4

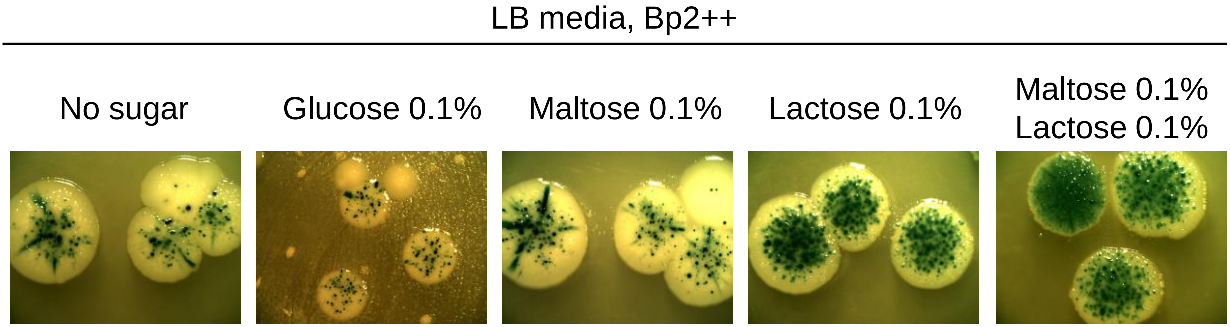

SF5. Effect of lactose and sucrose on the papillation assay.

The papillation assay was performed with Bp2++ untagged Hsmar1 vector and different sugars, glucose, maltose, lactose, and maltose and lactose. Representative field of view of the papillation plates are shown.

Supplementary Figure 5
