# Supplementary material for: Compensating for over-production inhibition of the Hsmar1 transposon in Escherichia coli using a series of constitutive promoters
Source: Mob DNA. 2020 Jan 10;11:5. doi: 10.1186/s13100-020-0200-5 (PMC6954556; doi:10.1186/s13100-020-0200-5)
Supplement: Supplementary file 2 — Additional file 2: Table S1. List of plasmids used in this study. [file 13100_2020_200_MOESM2_ESM.docx]

| Plasmid | Description | Marker |
| --- | --- | --- |
| pRC1782 | Flag-tagged EGFP with pEE with RBS++ in pBACe3.6 (BpEE++). | Cm^R^ |
| pRC1783 | Flag-tagged EGFP with p2 with RBS++ in pBACe3.6 (Bp2++). | Cm^R^ |
| pRC1784 | Flag-tagged EGFP with p3 with RBS++ in pBACe3.6 (Bp3++). | Cm^R^ |
| pRC1785 | Flag-tagged EGFP with p4 with RBS++ in pBACe3.6 (Bp4++). | Cm^R^ |
| pRC1786 | Flag-tagged EGFP with p5 with RBS++ in pBACe3.6 (Bp5++). | Cm^R^ |
| pRC1787 | Flag-tagged EGFP with p6 with RBS++ in pBACe3.6 (Bp6++). | Cm^R^ |
| pRC1788 | Flag-tagged EGFP with pEE with RBS++ in pGHM491 (IpEE++). | Spec^R^ |
| pRC1789 | Flag-tagged EGFP with p2 with RBS++ in pGHM491 (Ip2++). | Spec^R^ |
| pRC1790 | Flag-tagged EGFP with p3 with RBS++ in pGHM491 (Ip3++). | Spec^R^ |
| pRC1791 | Flag-tagged EGFP with p4 with RBS++ in pGHM491 (Ip4++). | Spec^R^ |
| pRC1792 | Flag-tagged EGFP with p5 with RBS++ in pGHM491 (Ip5++). | Spec^R^ |
| pRC1793 | Flag-tagged EGFP with p6 with RBS++ in pGHM491 (Ip6++). | Spec^R^ |
| pRC1794 | Flag-tagged EGFP with pEE without RBS in pBACe3.6 (BpEE-). | Cm^R^ |
| pRC1795 | Flag-tagged EGFP with p2 without RBS in pBACe3.6 (Bp2-). | Cm^R^ |
| pRC1796 | Flag-tagged EGFP with p3 without RBS in pBACe3.6 (Bp3-). | Cm^R^ |
| pRC1797 | Flag-tagged EGFP with p4 without RBS in pBACe3.6 (Bp4-). | Cm^R^ |
| pRC1798 | Flag-tagged EGFP with p5 without RBS in pBACe3.6 (Bp5-). | Cm^R^ |
| pRC1799 | Flag-tagged EGFP with p6 without RBS in pBACe3.6 (Bp6-). | Cm^R^ |
| pRC1800 | Flag-tagged EGFP with pEE without RBS in pGHM491 (IpEE-). | Spec^R^ |
| pRC1801 | Flag-tagged EGFP with p2 without RBS in pGHM491 (Ip2-). | Spec^R^ |
| pRC1802 | Flag-tagged EGFP with p3 without RBS in pGHM491 (Ip3-). | Spec^R^ |
| pRC1803 | Flag-tagged EGFP with p4 without RBS in pGHM491 (Ip4-). | Spec^R^ |
| pRC1804 | Flag-tagged EGFP with p5 without RBS in pGHM491 (Ip5-). | Spec^R^ |
| pRC1805 | Flag-tagged EGFP with p6 without RBS in pGHM491 (Ip6-). | Spec^R^ |
| pRC1806 | Flag-tagged EGFP with pEE with RBS+ in pBACe3.6 (BpEE+). | Cm^R^ |
| pRC1807 | Flag-tagged EGFP with pEE with RBS+ in pGHM491 (IpEE+). | Spec^R^ |
| pRC1723 | Untagged Hsmar1 with pEE with RBS+ in pBACe3.6 (BpEE+). | Cm^R^ |
| pRC1724 | Untagged Hsmar1 with p2 with RBS++ in pBACe3.6 (Bp2++). | Cm^R^ |
| pRC1725 | Untagged Hsmar1 with p3 with RBS++ in pBACe3.6 (Bp3++). | Cm^R^ |
| pRC1726 | Untagged Hsmar1 with p4 with RBS++ in pBACe3.6 (Bp4++). | Cm^R^ |
| pRC1727 | Untagged Hsmar1 with p5 with RBS++ in pBACe3.6 (Bp5++). | Cm^R^ |
| pRC1728 | Untagged Hsmar1 with p6 with RBS++ in pBACe3.6 (Bp6++). | Cm^R^ |
| pRC1730 | Untagged Hsmar1 with pEE with RBS+ in pGHM491 (IpEE+). | Spec^R^ |
| pRC1731 | Untagged Hsmar1 with p2 with RBS in pGHM491 (Ip2++). | Spec^R^ |
| pRC1732 | Untagged Hsmar1 with p3 with RBS in pGHM491 (Ip3++). | Spec^R^ |
| pRC1733 | Untagged Hsmar1 with p4 with RBS in pGHM491 (Ip4++). | Spec^R^ |
| pRC1734 | Untagged Hsmar1 with p5 with RBS in pGHM491 (Ip5++). | Spec^R^ |
| pRC1735 | Untagged Hsmar1 with p6 with RBS in pGHM491 (Ip6++). | Spec^R^ |
| pRC1821 | Flag-tagged Hsmar1 with pEE with RBS++ in pBACe3.6 (BpEE++). | Cm^R^ |
| pRC1822 | Flag-tagged Hsmar1 with p2 with RBS++ in pBACe3.6 (Bp2++). | Cm^R^ |
| pRC1823 | Flag-tagged Hsmar1 with p3 with RBS++ in pBACe3.6 (Bp3++). | Cm^R^ |
| pRC1824 | Flag-tagged Hsmar1 with p4 with RBS++ in pBACe3.6 (Bp4++). | Cm^R^ |
| pRC1825 | Flag-tagged Hsmar1 with p5 with RBS++ in pBACe3.6 (Bp5++). | Cm^R^ |
| pRC1826 | Flag-tagged Hsmar1 with p6 with RBS++ in pBACe3.6 (Bp6++). | Cm^R^ |
| pRC1827 | Flag-tagged Hsmar1 with pEE with RBS++ in pGHM491 (IpEE++). | Spec^R^ |
| pRC1828 | Flag-tagged Hsmar1 with p2 with RBS++ in pGHM491 (Ip2++). | Spec^R^ |
| pRC1829 | Flag-tagged Hsmar1 with p3 with RBS++ in pGHM491 (Ip3++). | Spec^R^ |
| pRC1830 | Flag-tagged Hsmar1 with p4 with RBS++ in pGHM491 (Ip4++). | Spec^R^ |
| pRC1831 | Flag-tagged Hsmar1 with p5 with RBS++ in pGHM491 (Ip5++). | Spec^R^ |
| pRC1832 | Flag-tagged Hsmar1 with p6 with RBS++ in pGHM491 (Ip6++). | Spec^R^ |
| pRC1833 | Flag-tagged Hsmar1 with pEE without RBS in pBACe3.6 (BpEE-). | Cm^R^ |
| pRC1834 | Flag-tagged Hsmar1 with p2 without RBS in pBACe3.6 (Bp2-). | Cm^R^ |
| pRC1835 | Flag-tagged Hsmar1 with p3 without RBS in pBACe3.6 (Bp3-). | Cm^R^ |
| pRC1836 | Flag-tagged Hsmar1 with p4 without RBS in pBACe3.6 (Bp4-). | Cm^R^ |
| pRC1837 | Flag-tagged Hsmar1 with p5 without RBS in pBACe3.6 (Bp5-). | Cm^R^ |
| pRC1838 | Flag-tagged Hsmar1 with p6 without RBS in pBACe3.6 (Bp6-). | Cm^R^ |
| pRC1839 | Flag-tagged Hsmar1 with pEE without RBS in pGHM491 (IpEE-). | Spec^R^ |
| pRC1840 | Flag-tagged Hsmar1 with p2 without RBS in pGHM491 (Ip2-). | Spec^R^ |
| pRC1841 | Flag-tagged Hsmar1 with p3 without RBS in pGHM491 (Ip3-). | Spec^R^ |
| pRC1842 | Flag-tagged Hsmar1 with p4 without RBS in pGHM491 (Ip4-). | Spec^R^ |
| pRC1843 | Flag-tagged Hsmar1 with p5 without RBS in pGHM491 (Ip5-). | Spec^R^ |
| pRC1844 | Flag-tagged Hsmar1 with p6 without RBS in pGHM491 (Ip6-). | Spec^R^ |
| pRC1845 | Flag-tagged Hsmar1 with pEE with RBS+ in pBACe3.6 (BpEE+). | Cm^R^ |
| pRC1846 | Flag-tagged Hsmar1 with pEE with RBS+ in pGHM491 (IpEE+). | Spec^R^ |
| pRC1858 | Flag-tagged Hsmar1 covalent dimer with p2 without RBS in pBACe3.6 (Bp2-). | Cm^R^ |
| pRC1859 | Flag-tagged Hsmar1 covalent dimer with p3 without RBS in pBACe3.6 (Bp3-). | Cm^R^ |
| pRC1860 | Flag-tagged Hsmar1 covalent dimer with p3 with RBS++ in pBACe3.6 (Bp3++). | Cm^R^ |
| pRC1861 | Flag-tagged Hsmar1 covalent dimer with p6 with RBS++ in pBACe3.6 (Bp6++). | Cm^R^ |
| pRC1863 | Flag-tagged Hsmar1 covalent dimer with p2 without RBS in pGHM491 (Ip2-). | Spec^R^ |
| pRC1865 | Flag-tagged Hsmar1 covalent dimer with p3 with RBS++ in pGHM491 (Ip3++). | Spec^R^ |
| pRC1866 | Flag-tagged Hsmar1 covalent dimer with p6 with RBS++ in pGHM491 (Ip6++). | Spec^R^ |
| pRC1868 | Flag-tagged Hsmar1 monomer with p2 without RBS in pBACe3.6 (Bp2-). | Cm^R^ |
| pRC1869 | Flag-tagged Hsmar1 monomer with p3 without RBS in pBACe3.6 (Bp3-). | Cm^R^ |
| pRC1870 | Flag-tagged Hsmar1 monomer with p3 with RBS++ in pBACe3.6 (Bp3++). | Cm^R^ |
| pRC1871 | Flag-tagged Hsmar1 monomer with p6 with RBS++ in pBACe3.6 (Bp6++). | Cm^R^ |
| pRC1873 | Flag-tagged Hsmar1 monomer with p2 without RBS in pGHM491 (Ip2-). | Spec^R^ |
| pRC1875 | Flag-tagged Hsmar1 monomer with p3 with RBS++ in pGHM491 (Ip3++). | Spec^R^ |
| pRC1876 | Flag-tagged Hsmar1 monomer with p6 with RBS++ in pGHM491 (Ip6++). | Spec^R^ |
| pRC1721 | Hsmar1 wild type in pMAL-C2X (without MBP-tag) | Amp^R^ |
| pRC1877 | Hsmar1 E2K in pMAL-C2X (without MBP-tag) | Amp^R^ |
| pRC1878 | Hsmar1 R53C in pMAL-C2X (without MBP-tag) | Amp^R^ |
| pRC1879 | Hsmar1 D98N in pMAL-C2X (without MBP-tag) | Amp^R^ |
| pRC1880 | Hsmar1 S124T in pMAL-C2X (without MBP-tag) | Amp^R^ |
| pRC1881 | Hsmar1 N143H in pMAL-C2X (without MBP-tag) | Amp^R^ |
| pRC1882 | Hsmar1 R173Q in pMAL-C2X (without MBP-tag) | Amp^R^ |
| pRC1883 | Hsmar1 N184I in pMAL-C2X (without MBP-tag) | Amp^R^ |
| pRC1884 | Hsmar1 Q187P in pMAL-C2X (without MBP-tag) | Amp^R^ |
| pRC1885 | Hsmar1 V194I in pMAL-C2X (without MBP-tag) | Amp^R^ |
| pRC1886 | Hsmar1 V201L in pMAL-C2X (without MBP-tag) | Amp^R^ |
| pRC1887 | Hsmar1 C219A in pMAL-C2X (without MBP-tag) | Amp^R^ |
| pRC1888 | Hsmar1 Q221E in pMAL-C2X (without MBP-tag) | Amp^R^ |
| pRC1889 | Hsmar1 H226N in pMAL-C2X (without MBP-tag) | Amp^R^ |
| pRC1890 | Hsmar1 R227Q in pMAL-C2X (without MBP-tag) | Amp^R^ |
| pRC1891 | Hsmar1 P234L in pMAL-C2X (without MBP-tag) | Amp^R^ |
| pRC1892 | Hsmar1 S279L in pMAL-C2X (without MBP-tag) | Amp^R^ |
| pRC1893 | Hsmar1 D282N in pMAL-C2X (without MBP-tag) | Amp^R^ |
| pRC1894 | Hsmar1 F285I in pMAL-C2X (without MBP-tag) | Amp^R^ |
| pRC1895 | Hsmar1 F285V in pMAL-C2X (without MBP-tag) | Amp^R^ |
| pRC1896 | Hsmar1 D290N in pMAL-C2X (without MBP-tag) | Amp^R^ |
| pRC1897 | Hsmar1 E313K in pMAL-C2X (without MBP-tag) | Amp^R^ |
| pRC1898 | Hsmar1 R315Q in pMAL-C2X (without MBP-tag) | Amp^R^ |
| pRC1899 | Hsmar1 K326Q in pMAL-C2X (without MBP-tag) | Amp^R^ |
| pRC1739 | Hsmar1 F132A with pEE with RBS+ in pBACe3.6 (Bp-EE+). | Cm^R^ |
| pRC1740 | Hsmar1 R141L with pEE with RBS+ in pBACe3.6 (Bp-EE+). | Cm^R^ |
| pRC1746 | Hsmar1 F132A with pEE with RBS+ in pGHM491 (Ip-EE+). | Spec^R^ |
| pRC1747 | Hsmar1 R141L with pEE with RBS+ in pGHM491 (Ip-EE+). | Spec^R^ |
| pRC1752 | Hsmar1 F132A with p6 with RBS++ in pGHM491 (Ip6++). | Spec^R^ |
| pRC1753 | Hsmar1 R141L with p6 with RBS++ in pGHM491 (Ip6++). | Spec^R^ |
