# Supplementary material for: Compensating for over-production inhibition of the Hsmar1 transposon in Escherichia coli using a series of constitutive promoters
Source: Mob DNA. 2020 Jan 10;11:5. doi: 10.1186/s13100-020-0200-5 (PMC6954556; doi:10.1186/s13100-020-0200-5)
Supplement: Supplementary file 3 — Additional file 3: Table S2. DNA sequences of the plasmids used in this study. [file 13100_2020_200_MOESM3_ESM.docx]

| Plasmid | Sequence |
| --- | --- |
| pRC880  (pMAL-c2X with MBP-Hsmar1) | CCGACACCATCGAATGGTGCAAAACCTTTCGCGGTATGGCATGATAGCGCCCGGAAGAGAGTCAATTCAGGGTGGTGAATGTGAAACCAGTAACGTTATACGATGTCGCAGAGTATGCCGGTGTCTCTTATCAGACCGTTTCCCGCGTGGTGAACCAGGCCAGCCACGTTTCTGCGAAAACGCGGGAAAAAGTGGAAGCGGCGATGGCGGAGCTGAATTACATTCCCAACCGCGTGGCACAACAACTGGCGGGCAAACAGTCGTTGCTGATTGGCGTTGCCACCTCCAGTCTGGCCCTGCACGCGCCGTCGCAAATTGTCGCGGCGATTAAATCTCGCGCCGATCAACTGGGTGCCAGCGTGGTGGTGTCGATGGTAGAACGAAGCGGCGTCGAAGCCTGTAAAGCGGCGGTGCACAATCTTCTCGCGCAACGCGTCAGTGGGCTGATCATTAACTATCCGCTGGATGACCAGGATGCCATTGCTGTGGAAGCTGCCTGCACTAATGTTCCGGCGTTATTTCTTGATGTCTCTGACCAGACACCCATCAACAGTATTATTTTCTCCCATGAAGACGGTACGCGACTGGGCGTGGAGCATCTGGTCGCATTGGGTCACCAGCAAATCGCGCTGTTAGCGGGCCCATTAAGTTCTGTCTCGGCGCGTCTGCGTCTGGCTGGCTGGCATAAATATCTCACTCGCAATCAAATTCAGCCGATAGCGGAACGGGAAGGCGACTGGAGTGCCATGTCCGGTTTTCAACAAACCATGCAAATGCTGAATGAGGGCATCGTTCCCACTGCGATGCTGGTTGCCAACGATCAGATGGCGCTGGGCGCAATGCGCGCCATTACCGAGTCCGGGCTGCGCGTTGGTGCGGATATCTCGGTAGTGGGATACGACGATACCGAAGACAGCTCATGTTATATCCCGCCGTTAACCACCATCAAACAGGATTTTCGCCTGCTGGGGCAAACCAGCGTGGACCGCTTGCTGCAACTCTCTCAGGGCCAGGCGGTGAAGGGCAATCAGCTGTTGCCCGTCTCACTGGTGAAAAGAAAAACCACCCTGGCGCCCAATACGCAAACCGCCTCTCCCCGCGCGTTGGCCGATTCATTAATGCAGCTGGCACGACAGGTTTCCCGACTGGAAAGCGGGCAGTGAGCGCAACGCAATTAATGTGAGTTAGCTCACTCATTAGGCACAATTCTCATGTTTGACAGCTTATCATCGACTGCACGGTGCACCAATGCTTCTGGCGTCAGGCAGCCATCGGAAGCTGTGGTATGGCTGTGCAGGTCGTAAATCACTGCATAATTCGTGTCGCTCAAGGCGCACTCCCGTTCTGGATAATGTTTTTTGCGCCGACATCATAACGGTTCTGGCAAATATTCTGAAATGAGCTGTTGACAATTAATCATCGGCTCGTATAATGTGTGGAATTGTGAGCGGATAACAATTTCACACAGGAAACAGCCAGTCCGTTTAGGTGTTTTCACGAGCACTTCACCAACAAGGACCATAGCATATGAAAATCGAAGAAGGTAAACTGGTAATCTGGATTAACGGCGATAAAGGCTATAACGGTCTCGCTGAAGTCGGTAAGAAATTCGAGAAAGATACCGGAATTAAAGTCACCGTTGAGCATCCGGATAAACTGGAAGAGAAATTCCCACAGGTTGCGGCAACTGGCGATGGCCCTGACATTATCTTCTGGGCACACGACCGCTTTGGTGGCTACGCTCAATCTGGCCTGTTGGCTGAAATCACCCCGGACAAAGCGTTCCAGGACAAGCTGTATCCGTTTACCTGGGATGCCGTACGTTACAACGGCAAGCTGATTGCTTACCCGATCGCTGTTGAAGCGTTATCGCTGATTTATAACAAAGATCTGCTGCCGAACCCGCCAAAAACCTGGGAAGAGATCCCGGCGCTGGATAAAGAACTGAAAGCGAAAGGTAAGAGCGCGCTGATGTTCAACCTGCAAGAACCGTACTTCACCTGGCCGCTGATTGCTGCTGACGGGGGTTATGCGTTCAAGTATGAAAACGGCAAGTACGACATTAAAGACGTGGGCGTGGATAACGCTGGCGCGAAAGCGGGTCTGACCTTCCTGGTTGACCTGATTAAAAACAAACACATGAATGCAGACACCGATTACTCCATCGCAGAAGCTGCCTTTAATAAAGGCGAAACAGCGATGACCATCAACGGCCCGTGGGCATGGTCCAACATCGACACCAGCAAAGTGAATTATGGTGTAACGGTACTGCCGACCTTCAAGGGTCAACCATCCAAACCGTTCGTTGGCGTGCTGAGCGCAGGTATTAACGCCGCCAGTCCGAACAAAGAGCTGGCAAAAGAGTTCCTCGAAAACTATCTGCTGACTGATGAAGGTCTGGAAGCGGTTAATAAAGACAAACCGCTGGGTGCCGTAGCGCTGAAGTCTTACGAGGAAGAGTTGGCGAAAGATCCACGTATTGCCGCCACTATGGAAAACGCCCAGAAAGGTGAAATCATGCCGAACATCCCGCAGATGTCCGCTTTCTGGTATGCCGTGCGTACTGCGGTGATCAACGCCGCCAGCGGTCGTCAGACTGTCGATGAAGCCCTGAAAGACGCGCAGACTAATTCGAGCTCGAACAACAACAACAATAACAATAACAACAACCTCGGGATCGAGGGAAGGATTTCAGaattcCATATGGAAATGATGCTCGATAAGAAACAGATTCGTGCGATCTTTCTCTTTGAGTTTAAAATGGGTCGCAAAGCGGCGGAGACGACGCGTAATATTAACAACGCGTTCGGTCCTGGCACCGCGAACGAGCGTACCGTGCAATGGTGGTTCAAAAAGTTTCGCAAAGGCGACGAATCTCTGGAGGACGAAGAGCGTTCTGGCCGCCCGTCCGAGGTTGACAACGACCAGCTGCGTGCAATCATCGAAGCTGATCCGCTGACTACCACCCGCGAAGTTGCTGAAGAACTGAATGTGGATCACTCTACTGTGGTTCGCCACCTGAAACAGATCGGTAAAGTAAAAAAACTGGACAAATGGGTTCCTCATGAACTGTCTGAAAACCAGAAAAACCGTCGTTTCGAAGTTAGCTCCTCTCTGATTCTGCGTAACAACAACGAACCGTTCCTGGATCGTATCGTAACCTGTGATGAGAAATGGATTCTGTATGATAACCGTCGCCGCTCTGCTCAGTGGCTGGATCGCGAAGAAGCTCCAAAACACTTCCCGAAACCGAATCTGCACCAGAAGAAAGTCATGGTAACCGTATGGTGGTCTGCCGCAGGTGTTATCCACTATTCCTTCCTGAACCCGGGCGAAACTATCACCAGCGAAAAATACTGCCAGCAGATTGACGAAATGCACCGTAAACTGCAGCGTCTGCAGCCAGCACTGGTGAATCGTAAAGGTCCGATCCTGCTGCATGATAACGCCCGTCCGCACGTTGCCCAACCGACCCTGCAGAAACTGAACGAACTGGGCTATGAAGTTCTGCCACACCCGCCGTACTCCCCGGATCTGTCCCCGACTGACTACCATTTCTTCAAGCATCTGGACAACTTCCTGCAGGGTAAACGTTTTCACAACCAACAGGACGCAGAAAACGCTTTCCAGGAGTTCGTCGAAAGCCGTTCCACTGACTTCTACGCGACCGGTATCAACAAGCTGATCAGCCGTTGGCAGAAATGCGTGGACTGTAACGGCAGCTACTTCGATTAAGGATCCTCTAGAGTCGACCTGCAGGCAAGCTTGGCACTGGCCGTCGTTTTACAACGTCGTGACTGGGAAAACCCTGGCGTTACCCAACTTAATCGCCTTGCAGCACATCCCCCTTTCGCCAGCTGGCGTAATAGCGAAGAGGCCCGCACCGATCGCCCTTCCCAACAGTTGCGCAGCCTGAATGGCGAATGGCAGCTTGGCTGTTTTGGCGGATGAGATAAGATTTTCAGCCTGATACAGATTAAATCAGAACGCAGAAGCGGTCTGATAAAACAGAATTTGCCTGGCGGCAGTAGCGCGGTGGTCCCACCTGACCCCATGCCGAACTCAGAAGTGAAACGCCGTAGCGCCGATGGTAGTGTGGGGTCTCCCCATGCGAGAGTAGGGAACTGCCAGGCATCAAATAAAACGAAAGGCTCAGTCGAAAGACTGGGCCTTTCGTTTTATCTGTTGTTTGTCGGTGAACGCTCTCCTGAGTAGGACAAATCCGCCGGGAGCGGATTTGAACGTTGCGAAGCAACGGCCCGGAGGGTGGCGGGCAGGACGCCCGCCATAAACTGCCAGGCATCAAATTAAGCAGAAGGCCATCCTGACGGATGGCCTTTTTGCGTTTCTACAAACTCTTTTTGTTTATTTTTCTAAATACATTCAAATATGTATCCGCTCATGAGACAATAACCCTGATAAATGCTTCAATAATATTGAAAAAGGAAGAGTATGAGTATTCAACATTTCCGTGTCGCCCTTATTCCCTTTTTTGCGGCATTTTGCCTTCCTGTTTTTGCTCACCCAGAAACGCTGGTGAAAGTAAAAGATGCTGAAGATCAGTTGGGTGCACGAGTGGGTTACATCGAACTGGATCTCAACAGCGGTAAGATCCTTGAGAGTTTTCGCCCCGAAGAACGTTCTCCAATGATGAGCACTTTTAAAGTTCTGCTATGTGGCGCGGTATTATCCCGTGTTGACGCCGGGCAAGAGCAACTCGGTCGCCGCATACACTATTCTCAGAATGACTTGGTTGAGTACTCACCAGTCACAGAAAAGCATCTTACGGATGGCATGACAGTAAGAGAATTATGCAGTGCTGCCATAACCATGAGTGATAACACTGCGGCCAACTTACTTCTGACAACGATCGGAGGACCGAAGGAGCTAACCGCTTTTTTGCACAACATGGGGGATCATGTAACTCGCCTTGATCGTTGGGAACCGGAGCTGAATGAAGCCATACCAAACGACGAGCGTGACACCACGATGCCTGTAGCAATGGCAACAACGTTGCGCAAACTATTAACTGGCGAACTACTTACTCTAGCTTCCCGGCAACAATTAATAGACTGGATGGAGGCGGATAAAGTTGCAGGACCACTTCTGCGCTCGGCCCTTCCGGCTGGCTGGTTTATTGCTGATAAATCTGGAGCCGGTGAGCGTGGGTCTCGCGGTATCATTGCAGCACTGGGGCCAGATGGTAAGCCCTCCCGTATCGTAGTTATCTACACGACGGGGAGTCAGGCAACTATGGATGAACGAAATAGACAGATCGCTGAGATAGGTGCCTCACTGATTAAGCATTGGTAACTGTCAGACCAAGTTTACTCATATATACTTTAGATTGATTTACCCCGGTTGATAATCAGAAAAGCCCCAAAAACAGGAAGATTGTATAAGCAAATATTTAAATTGTAAACGTTAATATTTTGTTAAAATTCGCGTTAAATTTTTGTTAAATCAGCTCATTTTTTAACCAATAGGCCGAAATCGGCAAAATCCCTTATAAATCAAAAGAATAGACCGAGATAGGGTTGAGTGTTGTTCCAGTTTGGAACAAGAGTCCACTATTAAAGAACGTGGACTCCAACGTCAAAGGGCGAAAAACCGTCTATCAGGGCGATGGCCCACTACGTGAACCATCACCCAAATCAAGTTTTTTGGGGTCGAGGTGCCGTAAAGCACTAAATCGGAACCCTAAAGGGAGCCCCCGATTTAGAGCTTGACGGGGAAAGCCGGCGAACGTGGCGAGAAAGGAAGGGAAGAAAGCGAAAGGAGCGGGCGCTAGGGCGCTGGCAAGTGTAGCGGTCACGCTGCGCGTAACCACCACACCCGCCGCGCTTAATGCGCCGCTACAGGGCGCGTAAAAGGATCTAGGTGAAGATCCTTTTTGATAATCTCATGACCAAAATCCCTTAACGTGAGTTTTCGTTCCACTGAGCGTCAGACCCCGTAGAAAAGATCAAAGGATCTTCTTGAGATCCTTTTTTTCTGCGCGTAATCTGCTGCTTGCAAACAAAAAAACCACCGCTACCAGCGGTGGTTTGTTTGCCGGATCAAGAGCTACCAACTCTTTTTCCGAAGGTAACTGGCTTCAGCAGAGCGCAGATACCAAATACTGTCCTTCTAGTGTAGCCGTAGTTAGGCCACCACTTCAAGAACTCTGTAGCACCGCCTACATACCTCGCTCTGCTAATCCTGTTACCAGTGGCTGCTGCCAGTGGCGATAAGTCGTGTCTTACCGGGTTGGACTCAAGACGATAGTTACCGGATAAGGCGCAGCGGTCGGGCTGAACGGGGGGTTCGTGCACACAGCCCAGCTTGGAGCGAACGACCTACACCGAACTGAGATACCTACAGCGTGAGCTATGAGAAAGCGCCACGCTTCCCGAAGGGAGAAAGGCGGACAGGTATCCGGTAAGCGGCAGGGTCGGAACAGGAGAGCGCACGAGGGAGCTTCCAGGGGGAAACGCCTGGTATCTTTATAGTCCTGTCGGGTTTCGCCACCTCTGACTTGAGCGTCGATTTTTGTGATGCTCGTCAGGGGGGCGGAGCCTATGGAAAAACGCCAGCAACGCGGCCTTTTTACGGTTCCTGGCCTTTTGCTGGCCTTTTGCTCACATGTTCTTTCCTGCGTTATCCCCTGATTCTGTGGATAACCGTATTACCGCCTTTGAGTGAGCTGATACCGCTCGCCGCAGCCGAACGACCGAGCGCAGCGAGTCAGTGAGCGAGGAAGCGGAAGAGCGCCTGATGCGGTATTTTCTCCTTACGCATCTGTGCGGTATTTCACACCGCATATATGGTGCACTCTCAGTACAATCTGCTCTGATGCCGCATAGTTAAGCCAGTATACACTCCGCTATCGCTACGTGACTGGGTCATGGCTGCGCCCCGACACCCGCCAACACCCGCTGACGCGCCCTGACGGGCTTGTCTGCTCCCGGCATCCGCTTACAGACAAGCTGTGACCGTCTCCGGGAGCTGCATGTGTCAGAGGTTTTCACCGTCATCACCGAAACGCGCGAGGCAGCTGCGGTAAAGCTCATCAGCGTGGTCGTGCAGCGATTCACAGATGTCTGCCTGTTCATCCGCGTCCAGCTCGTTGAGTTTCTCCAGAAGCGTTAATGTCTGGCTTCTGATAAAGCGGGCCATGTTAAGGGCGGTTTTTTCCTGTTTGGTCACTTGATGCCTCCGTGTAAGGGGGAATTTCTGTTCATGGGGGTAATGATACCGATGAAACGAGAGAGGATGCTCACGATACGGGTTACTGATGATGAACATGCCCGGTTACTGGAACGTTGTGAGGGTAAACAACTGGCGGTATGGATGCGGCGGGACCAGAGAAAAATCACTCAGGGTCAATGCCAGCGCTTCGTTAATACAGATGTAGGTGTTCCACAGGGTAGCCAGCAGCATCCTGCGATGCAGATCCGGAACATAATGGTGCAGGGCGCTGACTTCCGCGTTTCCAGACTTTACGAAACACGGAAACCGAAGACCATTCATGTTGTTGCTCAGGTCGCAGACGTTTTGCAGCAGCAGTCGCTTCACGTTCGCTCGCGTATCGGTGATTCATTCTGCTAACCAGTAAGGCAACCCCGCCAGCCTAGCCGGGTCCTCAACGACAGGAGCACGATCATGCGCACCCGTGGCCAGGACCCAACGCTGCCCGAAATT |
| pRC1721  (pMAL-c2x with native Hsmar1, no MBP-tag) | CCGACACCATCGAATGGTGCAAAACCTTTCGCGGTATGGCATGATAGCGCCCGGAAGAGAGTCAATTCAGGGTGGTGAATGTGAAACCAGTAACGTTATACGATGTCGCAGAGTATGCCGGTGTCTCTTATCAGACCGTTTCCCGCGTGGTGAACCAGGCCAGCCACGTTTCTGCGAAAACGCGGGAAAAAGTGGAAGCGGCGATGGCGGAGCTGAATTACATTCCCAACCGCGTGGCACAACAACTGGCGGGCAAACAGTCGTTGCTGATTGGCGTTGCCACCTCCAGTCTGGCCCTGCACGCGCCGTCGCAAATTGTCGCGGCGATTAAATCTCGCGCCGATCAACTGGGTGCCAGCGTGGTGGTGTCGATGGTAGAACGAAGCGGCGTCGAAGCCTGTAAAGCGGCGGTGCACAATCTTCTCGCGCAACGCGTCAGTGGGCTGATCATTAACTATCCGCTGGATGACCAGGATGCCATTGCTGTGGAAGCTGCCTGCACTAATGTTCCGGCGTTATTTCTTGATGTCTCTGACCAGACACCCATCAACAGTATTATTTTCTCCCATGAAGACGGTACGCGACTGGGCGTGGAGCATCTGGTCGCATTGGGTCACCAGCAAATCGCGCTGTTAGCGGGCCCATTAAGTTCTGTCTCGGCGCGTCTGCGTCTGGCTGGCTGGCATAAATATCTCACTCGCAATCAAATTCAGCCGATAGCGGAACGGGAAGGCGACTGGAGTGCCATGTCCGGTTTTCAACAAACCATGCAAATGCTGAATGAGGGCATCGTTCCCACTGCGATGCTGGTTGCCAACGATCAGATGGCGCTGGGCGCAATGCGCGCCATTACCGAGTCCGGGCTGCGCGTTGGTGCGGATATCTCGGTAGTGGGATACGACGATACCGAAGACAGCTCATGTTATATCCCGCCGTTAACCACCATCAAACAGGATTTTCGCCTGCTGGGGCAAACCAGCGTGGACCGCTTGCTGCAACTCTCTCAGGGCCAGGCGGTGAAGGGCAATCAGCTGTTGCCCGTCTCACTGGTGAAAAGAAAAACCACCCTGGCGCCCAATACGCAAACCGCCTCTCCCCGCGCGTTGGCCGATTCATTAATGCAGCTGGCACGACAGGTTTCCCGACTGGAAAGCGGGCAGTGAGCGCAACGCAATTAATGTGAGTTAGCTCACTCATTAGGCACAATTCTCATGTTTGACAGCTTATCATCGACTGCACGGTGCACCAATGCTTCTGGCGTCAGGCAGCCATCGGAAGCTGTGGTATGGCTGTGCAGGTCGTAAATCACTGCATAATTCGTGTCGCTCAAGGCGCACTCCCGTTCTGGATAATGTTTTTTGCGCCGACATCATAACGGTTCTGGCAAATATTCTGAAATGAGCTGTTGACAATTAATCATCGGCTCGTATAATGTGTGGAATTGTGAGCGGATAACAATTTCACACAGGAAACAGCCAGTCCGTTTAGGTGTTTTCACGAGCACTTCACCAACAAGGACCATAGCATATGGAAATGATGCTCGATAAGAAACAGATTCGTGCGATCTTTCTCTTTGAGTTTAAAATGGGTCGCAAAGCGGCGGAGACGACGCGTAATATTAACAACGCGTTCGGTCCTGGCACCGCGAACGAGCGTACCGTGCAATGGTGGTTCAAAAAGTTTCGCAAAGGCGACGAATCTCTGGAGGACGAAGAGCGTTCTGGCCGCCCGTCCGAGGTTGACAACGACCAGCTGCGTGCAATCATCGAAGCTGATCCGCTGACTACCACCCGCGAAGTTGCTGAAGAACTGAATGTGGATCACTCTACTGTGGTTCGCCACCTGAAACAGATCGGTAAAGTAAAAAAACTGGACAAATGGGTTCCTCATGAACTGTCTGAAAACCAGAAAAACCGTCGTTTCGAAGTTAGCTCCTCTCTGATTCTGCGTAACAACAACGAACCGTTCCTGGATCGTATCGTAACCTGTGATGAGAAATGGATTCTGTATGATAACCGTCGCCGCTCTGCTCAGTGGCTGGATCGCGAAGAAGCTCCAAAACACTTCCCGAAACCGAATCTGCACCAGAAGAAAGTCATGGTAACCGTATGGTGGTCTGCCGCAGGTGTTATCCACTATTCCTTCCTGAACCCGGGCGAAACTATCACCAGCGAAAAATACTGCCAGCAGATTGACGAAATGCACCGTAAACTGCAGCGTCTGCAGCCAGCACTGGTGAATCGTAAAGGTCCGATCCTGCTGCATGATAACGCCCGTCCGCACGTTGCCCAACCGACCCTGCAGAAACTGAACGAACTGGGCTATGAAGTTCTGCCACACCCGCCGTACTCCCCGGATCTGTCCCCGACTGACTACCATTTCTTCAAGCATCTGGACAACTTCCTGCAGGGTAAACGTTTTCACAACCAACAGGACGCAGAAAACGCTTTCCAGGAGTTCGTCGAAAGCCGTTCCACTGACTTCTACGCGACCGGTATCAACAAGCTGATCAGCCGTTGGCAGAAATGCGTGGACTGTAACGGCAGCTACTTCGATTAAGGATCCTCTAGAGTCGACCTGCAGGCAAGCTTGGCACTGGCCGTCGTTTTACAACGTCGTGACTGGGAAAACCCTGGCGTTACCCAACTTAATCGCCTTGCAGCACATCCCCCTTTCGCCAGCTGGCGTAATAGCGAAGAGGCCCGCACCGATCGCCCTTCCCAACAGTTGCGCAGCCTGAATGGCGAATGGCAGCTTGGCTGTTTTGGCGGATGAGATAAGATTTTCAGCCTGATACAGATTAAATCAGAACGCAGAAGCGGTCTGATAAAACAGAATTTGCCTGGCGGCAGTAGCGCGGTGGTCCCACCTGACCCCATGCCGAACTCAGAAGTGAAACGCCGTAGCGCCGATGGTAGTGTGGGGTCTCCCCATGCGAGAGTAGGGAACTGCCAGGCATCAAATAAAACGAAAGGCTCAGTCGAAAGACTGGGCCTTTCGTTTTATCTGTTGTTTGTCGGTGAACGCTCTCCTGAGTAGGACAAATCCGCCGGGAGCGGATTTGAACGTTGCGAAGCAACGGCCCGGAGGGTGGCGGGCAGGACGCCCGCCATAAACTGCCAGGCATCAAATTAAGCAGAAGGCCATCCTGACGGATGGCCTTTTTGCGTTTCTACAAACTCTTTTTGTTTATTTTTCTAAATACATTCAAATATGTATCCGCTCATGAGACAATAACCCTGATAAATGCTTCAATAATATTGAAAAAGGAAGAGTATGAGTATTCAACATTTCCGTGTCGCCCTTATTCCCTTTTTTGCGGCATTTTGCCTTCCTGTTTTTGCTCACCCAGAAACGCTGGTGAAAGTAAAAGATGCTGAAGATCAGTTGGGTGCACGAGTGGGTTACATCGAACTGGATCTCAACAGCGGTAAGATCCTTGAGAGTTTTCGCCCCGAAGAACGTTCTCCAATGATGAGCACTTTTAAAGTTCTGCTATGTGGCGCGGTATTATCCCGTGTTGACGCCGGGCAAGAGCAACTCGGTCGCCGCATACACTATTCTCAGAATGACTTGGTTGAGTACTCACCAGTCACAGAAAAGCATCTTACGGATGGCATGACAGTAAGAGAATTATGCAGTGCTGCCATAACCATGAGTGATAACACTGCGGCCAACTTACTTCTGACAACGATCGGAGGACCGAAGGAGCTAACCGCTTTTTTGCACAACATGGGGGATCATGTAACTCGCCTTGATCGTTGGGAACCGGAGCTGAATGAAGCCATACCAAACGACGAGCGTGACACCACGATGCCTGTAGCAATGGCAACAACGTTGCGCAAACTATTAACTGGCGAACTACTTACTCTAGCTTCCCGGCAACAATTAATAGACTGGATGGAGGCGGATAAAGTTGCAGGACCACTTCTGCGCTCGGCCCTTCCGGCTGGCTGGTTTATTGCTGATAAATCTGGAGCCGGTGAGCGTGGGTCTCGCGGTATCATTGCAGCACTGGGGCCAGATGGTAAGCCCTCCCGTATCGTAGTTATCTACACGACGGGGAGTCAGGCAACTATGGATGAACGAAATAGACAGATCGCTGAGATAGGTGCCTCACTGATTAAGCATTGGTAACTGTCAGACCAAGTTTACTCATATATACTTTAGATTGATTTACCCCGGTTGATAATCAGAAAAGCCCCAAAAACAGGAAGATTGTATAAGCAAATATTTAAATTGTAAACGTTAATATTTTGTTAAAATTCGCGTTAAATTTTTGTTAAATCAGCTCATTTTTTAACCAATAGGCCGAAATCGGCAAAATCCCTTATAAATCAAAAGAATAGACCGAGATAGGGTTGAGTGTTGTTCCAGTTTGGAACAAGAGTCCACTATTAAAGAACGTGGACTCCAACGTCAAAGGGCGAAAAACCGTCTATCAGGGCGATGGCCCACTACGTGAACCATCACCCAAATCAAGTTTTTTGGGGTCGAGGTGCCGTAAAGCACTAAATCGGAACCCTAAAGGGAGCCCCCGATTTAGAGCTTGACGGGGAAAGCCGGCGAACGTGGCGAGAAAGGAAGGGAAGAAAGCGAAAGGAGCGGGCGCTAGGGCGCTGGCAAGTGTAGCGGTCACGCTGCGCGTAACCACCACACCCGCCGCGCTTAATGCGCCGCTACAGGGCGCGTAAAAGGATCTAGGTGAAGATCCTTTTTGATAATCTCATGACCAAAATCCCTTAACGTGAGTTTTCGTTCCACTGAGCGTCAGACCCCGTAGAAAAGATCAAAGGATCTTCTTGAGATCCTTTTTTTCTGCGCGTAATCTGCTGCTTGCAAACAAAAAAACCACCGCTACCAGCGGTGGTTTGTTTGCCGGATCAAGAGCTACCAACTCTTTTTCCGAAGGTAACTGGCTTCAGCAGAGCGCAGATACCAAATACTGTCCTTCTAGTGTAGCCGTAGTTAGGCCACCACTTCAAGAACTCTGTAGCACCGCCTACATACCTCGCTCTGCTAATCCTGTTACCAGTGGCTGCTGCCAGTGGCGATAAGTCGTGTCTTACCGGGTTGGACTCAAGACGATAGTTACCGGATAAGGCGCAGCGGTCGGGCTGAACGGGGGGTTCGTGCACACAGCCCAGCTTGGAGCGAACGACCTACACCGAACTGAGATACCTACAGCGTGAGCTATGAGAAAGCGCCACGCTTCCCGAAGGGAGAAAGGCGGACAGGTATCCGGTAAGCGGCAGGGTCGGAACAGGAGAGCGCACGAGGGAGCTTCCAGGGGGAAACGCCTGGTATCTTTATAGTCCTGTCGGGTTTCGCCACCTCTGACTTGAGCGTCGATTTTTGTGATGCTCGTCAGGGGGGCGGAGCCTATGGAAAAACGCCAGCAACGCGGCCTTTTTACGGTTCCTGGCCTTTTGCTGGCCTTTTGCTCACATGTTCTTTCCTGCGTTATCCCCTGATTCTGTGGATAACCGTATTACCGCCTTTGAGTGAGCTGATACCGCTCGCCGCAGCCGAACGACCGAGCGCAGCGAGTCAGTGAGCGAGGAAGCGGAAGAGCGCCTGATGCGGTATTTTCTCCTTACGCATCTGTGCGGTATTTCACACCGCATATATGGTGCACTCTCAGTACAATCTGCTCTGATGCCGCATAGTTAAGCCAGTATACACTCCGCTATCGCTACGTGACTGGGTCATGGCTGCGCCCCGACACCCGCCAACACCCGCTGACGCGCCCTGACGGGCTTGTCTGCTCCCGGCATCCGCTTACAGACAAGCTGTGACCGTCTCCGGGAGCTGCATGTGTCAGAGGTTTTCACCGTCATCACCGAAACGCGCGAGGCAGCTGCGGTAAAGCTCATCAGCGTGGTCGTGCAGCGATTCACAGATGTCTGCCTGTTCATCCGCGTCCAGCTCGTTGAGTTTCTCCAGAAGCGTTAATGTCTGGCTTCTGATAAAGCGGGCCATGTTAAGGGCGGTTTTTTCCTGTTTGGTCACTTGATGCCTCCGTGTAAGGGGGAATTTCTGTTCATGGGGGTAATGATACCGATGAAACGAGAGAGGATGCTCACGATACGGGTTACTGATGATGAACATGCCCGGTTACTGGAACGTTGTGAGGGTAAACAACTGGCGGTATGGATGCGGCGGGACCAGAGAAAAATCACTCAGGGTCAATGCCAGCGCTTCGTTAATACAGATGTAGGTGTTCCACAGGGTAGCCAGCAGCATCCTGCGATGCAGATCCGGAACATAATGGTGCAGGGCGCTGACTTCCGCGTTTCCAGACTTTACGAAACACGGAAACCGAAGACCATTCATGTTGTTGCTCAGGTCGCAGACGTTTTGCAGCAGCAGTCGCTTCACGTTCGCTCGCGTATCGGTGATTCATTCTGCTAACCAGTAAGGCAACCCCGCCAGCCTAGCCGGGTCCTCAACGACAGGAGCACGATCATGCGCACCCGTGGCCAGGACCCAACGCTGCCCGAAATT |
| pRC1782 (Bp-EE++, EGFP Flag) | gatccgcggaattgTTAATAACAGGGGACGTGGTAATCCGTCCCCTTTTTATTTCTGACTGAGTTAATAACAGGCCTGCTTCGGCAGGCCTTTTTATTTCTGACTGAGTTCTTCTCAGGCCTGCTGGTAATCGCAGGCCTTTTTATTTTCTAGgCTGACTGACTGACTGACTGACTGACTGACTGACTGACTGACTGACTGACTGACTGACTGACTGACTGACTGACTGACTGACTGACTGACTGACTGACTGACTaaggagCTGACCATATGGTGAGCAAGGGCGAGGAGCTGTTCACCGGGGTGGTGCCCATCCTGGTCGAGCTGGACGGCGACGTAAACGGCCACAAGTTCAGCGTGCGCGGCGAGGGCGAGGGCGATGCCACCAACGGCAAGCTGACCCTGAAGTTCATCTGCACCACCGGCAAGCTGCCCGTGCCCTGGCCCACCCTCGTGACCACCCTGACCTACGGCGTGCAGTGCTTCAGCCGCTACCCCGACCACATGAAGCAGCACGACTTCTTCAAGTCCGCCATGCCCGAAGGCTACGTCCAGGAGCGCACCATCTCCTTCAAGGACGACGGCACCTACAAGACCCGCGCCGAGGTGAAGTTCGAGGGCGACACCCTGGTGAACCGCATCGAGCTGAAGGGCATCGACTTCAAGGAGGACGGCAACATCCTGGGGCACAAGCTGGAGTACAACTACAACAGCCACAACGTCTATATCACCGCTGACAAGCAGAAGAACGGCATCAAGGCCAACTTCAAGATCCGCCACAACATCGAGGACGGCAGCGTGCAGCTCGCCGACCACTACCAGCAGAACACCCCCATCGGCGACGGCCCCGTGCTGCTGCCCGACAACCACTACCTGAGCACCCAGTCCGCCCTGAGCAAAGACCCCAACGAGAAGCGCGATCACATGGTCCTGCTGGAGTTCGTGACCGCCGCCGGGATCACTCTCGGCATGGACGAGCTGTACAAGTAAGGATCCgactacaaggacgacgatgacaagGAAgattataaagatgatgacgataaaAGAgactacaaggacgacgatgacaagTAAAAGCTTGTTCTTCTCAGGCCTGCTGGTAATCGCAGGCCTTTTTATTTGAATTCcgcggatccttctatagtgtcacctaaatgtcgacggccaggcggccgccaggcctacccactagtcaattcgggaggatcgaaacggcagatcgcaaaaaacagtacatacagaaggagacatgaacatgaacatcaaaaaaattgtaaaacaagccacagttctgacttttacgactgcacttctggcaggaggagcgactcaagccttcgcgaaagaaaataaccaaaaagcatacaaagaaacgtacggcgtctctcatattacacgccatgatatgctgcagatccctaaacagcagcaaaacgaaaaataccaagtgcctcaattcgatcaatcaacgattaaaaatattgagtctgcaaaaggacttgatgtgtgggacagctggccgctgcaaaacgctgacggaacagtagctgaatacaacggctatcacgttgtgtttgctcttgcgggaagcccgaaagacgctgatgacacatcaatctacatgttttatcaaaaggtcggcgacaactcaatcgacagctggaaaaacgcgggccgtgtctttaaagacagcgataagttcgacgccaacgatccgatcctgaaagatcagacgcaagaatggtccggttctgcaacctttacatctgacggaaaaatccgtttattctacactgactattccggtaaacattacggcaaacaaagcctgacaacagcgcaggtaaatgtgtcaaaatctgatgacacactcaaaatcaacggagtggaagatcacaaaacgatttttgacggagacggaaaaacatatcagaacgttcagcagtttatcgatgaaggcaattatacatccggcgacaaccatacgctgagagaccctcactacgttgaagacaaaggccataaataccttgtattcgaagccaacacgggaacagaaaacggataccaaggcgaagaatctttatttaacaaagcgtactacggcggcggcacgaacttcttccgtaaagaaagccagaagcttcagcagagcgctaaaaaacgcgatgctgagttagcgaacggcgccctcggtatcatagagttaaataatgattacacattgaaaaaagtaatgaagccgctgatcacttcaaacacggtaactgatgaaatcgagcgcgcgaatgttttcaaaatgaacggcaaatggtacttgttcactgattcacgcggttcaaaaatgacgatcgatggtattaactcaaacgatatttacatgcttggttatgtatcaaactctttaaccggcccttacaagccgctgaacaaaacagggcttgtgctgcaaatgggtcttgatccaaacgatgtgacattcacttactctcacttcgcagtgccgcaagccaaaggcaacaatgtggttatcacaagctacatgacaaacagaggcttcttcgaggataaaaaggcaacatttgcgccaagcttcttaatgaacatcaaaggcaataaaacatccgttgtcaaaaacagcatcctggagcaaggacagctgacagtcaactaataacagcaaaaagaaaatgccgatacttcattggcattttcttttatttctcaacaagatggtgaattgactagtgggtagatccacaggacgggtgtggtcgccatgatcgcgtagtcgatagtggctccaagtagcgaagcgagcaggactgggcggcggccaaagcggtcggacagtgctccgagaacgggtgcgcatagaaattgcatcaacgcatatagcgctagcagcacgccatagtgactggcgatgctgtcggaatggacgatatcccgcaagaggcccggcagtaccggcataaccaagcctatgcctacagcatccagggtgacggtgccgaggatgacgatgagcgcattgttagatttcatacacggtgcctgactgcgttagcaatttaactgtgataaactaccgcattaaagcttatcgatgataagctgtcaaacatgagaattgatccggaacccttaatataacttcgtataatgtatgctatacgaagttattaggtccctcgactatagggtcaccgtcgacagcgacacacttgcatcggatgcagcccggttaacgtgccggcacggcctgggtaaccaggtattttgtccacataaccgtgcgcaaaatgttgtggataagcaggacacagcagcaatccacagcaggcatacaaccgcacaccgaggttactccgttctacaggttacgacgacatgtcaatacttgcccttgacaggcattgatggaatcgtagtctcacgctgatagtctgatcgacaatacaagtgggaccgtggtcccagaccgataatcagaccgacaacacgagtgggatcgtggtcccagactaataatcagaccgacgatacgagtgggaccgtggtcccagactaataatcagaccgacgatacgagtgggaccgtggttccagactaataatcagaccgacgatacgagtgggaccgtggtcccagactaataatcagaccgacgatacgagtgggaccatggtcccagactaataatcagaccgacgatacgagtgggaccgtggtcccagtctgattatcagaccgacgatacgagtgggaccgtggtcccagactaataatcagaccgacgatacgagtgggaccgtggtcccagactaataatcagaccgacgatacgagtgggaccgtggtcccagtctgattatcagaccgacgatacaagtggaacagtgggcccagagagaatattcaggccagttatgctttctggcctgtaacaaaggacattaagtaaagacagataaacgtagactaaaacgtggtcgcatcagggtgctggcttttcaagttccttaagaatggcctcaattttctctatacactcagttggaacacgagacctgtccaggttaagcaccattttatcgcccttatacaatactgtcgctccaggagcaaactgatgtcgtgagcttaaactagttcttgatgcagatgacgttttaagcacagaagttaaaagagtgataacttcttcagcttcaaatatcaccccagcttttttctgctcatgaaggttagatgcctgctgcttaagtaattcctctttatctgtaaaggctttttgaagtgcatcacctgaccgggcagatagttcaccggggtgagaaaaaagagcaacaactgatttaggcaatttggcggtgttgatacagcgggtaataatcttacgtgaaatattttccgcatcagccagcgcagaaatatttccagcaaattcattctgcaatcggcttgcataacgctgaccacgttcataagcacttgttgggcgataatcgttacccaatctggataatgcagccatctgctcatcatccagctcgccaaccagaacacgataatcactttcggtaagtgcagcagctttacgacggcgactcccatcggcaatttctatgacaccagatactcttcgaccgaacgccggtgtctgttgaccagtcagtagaaaagaagggatgagatcatccagtgcgtcctcagtaagcagctcctggtcacgttcattacctgaccatacccgagaggtcttctcaacactatcaccccggagcacttcaagagtaaacttcacatcccgaccacatacaggcaaagtaatggcattaccgcgagccattactcctacgcgcgcaattaacgaatccaccatcggggcagctggtgtcgataacgaagtatcttcaaccggttgagtattgagcgtatgttttggaataacaggcgcacgcttcattatctaatctcccagcgtggtttaatcagacgatcgaaaatttcattgcagacaggttcccaaatagaaagagcatttctccaggcaccagttgaagagcgttgatcaatggcctgttcaaaaacagttctcatccggatctgacctttaccaacttcatccgtttcacgtacaacattttttagaaccatgcttccccaggcatcccgaatttgctcctccatccacggggactgagagccattactattgctgtatttggtaagcaaaatacgtacatcaggctcgaaccctttaagatcaacgttcttgagcagatcacgaagcatatcgaaaaactgcagtgcggaggtgtagtcaaacaactcagcaggcgtgggaacaatcagcacatcagcagcacatacgacattaatcgtgccgatacccaggttaggcgcgctgtcaataactatgacatcatagtcatgagcaacagtttcaatggccagtcggagcatcaggtgtggatcggtgggcagtttaccttcatcaaatttgcccattaactcagtttcaatacggtgcagagccagacaggaaggaataatgtcaagccccggccagcaagtgggctttattgcataagtgacatcgtccttttccccaagatagaaaggcaggagagtgtcttctgcatgaatatgaagatctggtacccatccgtgatacattgaggctgttccctgggggtcgttaccttccacgagcaaaacacgtagccccttcagagccagatcctgagcaagatgaacagaaactgaggttttgtaaacgccacctttatgggcagcaaccccgatcaccggtggaaatacgtcttcagcacgtcgcaatcgcgtaccaaacacatcacgcatatgattaatttgttcaattgtataaccaacacgttgctcaacccgtcctcgaatttccatatccgggtgcggtagtcgccctgctttctcggcatctctgatagcctgagaagaaaccccaactaaatccgctgcttcacctattctccagcgccgggttattttcctcgcttccgggctgtcatcattaaactgtgcaatggcgatagccttcgtcatttcatgaccagcgtttatgcactggttaagtgtttccatgagtttcattctgaacatcctttaatcattgctttgcgtttttttattaaatcttgcaatttactgcaaagcaacaacaaaatcgcaaagtcatcaaaaaaccgcaaagttgtttaaaataagagcaacactacaaaaggagataagaagagcacatacctcagtcacttattatcactagcgctcgccgcagccgtgtaaccgagcatagcgagcgaactggcgaggaagcaaagaagaactgttctgtcagatagctcttacgctcagcgcaagaagaaatatccaccgtgggaaaaactccaggtagaggtacacacgcggatagccaattcagagtaataaactgtgataatcaaccctcatcaatgatgacgaactaacccccgatatcaggtcacatgacgaagggaaagagaaggaaatcaactgtgacaaactgccctcaaatttggcttccttaaaaattacagttcaaaaagtatgagaaaatccatgcaggctgaaggaaacagcaaaactgtgacaaattaccctcagtaggtcagaacaaatgtgacgaaccaccctcaaatctgtgacagataaccctcagactatcctgtcgtcatggaagtgatatcgcggaaggaaaatacgatatgagtcgtctggcggcctttctttttctcaatgtatgagaggcgcattggagttctgctgttgatctcattaacacagacctgcaggaagcggcggcggaagtcaggcatacgctggtaactttgaggcagctggtaacgctctatgatccagtcgattttcagagagacgatgcctgagccatccggcttacgatactgacacagggattcgtataaacgcatggcatacggattggtgatttcttttgtttcactaagccgaaactgcgtaaaccggttctgtaacccgataaagaagggaatgagatatgggttgatatgtacactgtaaagccctctggatggactgtgcgcacgtttgataaaccaaggaaaagattcatagcctttttcatcgccggcatcctcttcagggcgataaaaaaccacttccttccccgcgaaactcttcaatgcctgccgtatatccttactggcttccgcagaggtcaatccgaatatttcagcatatttagcaacatggatctcgcagataccgtcatgttcctgtagggtgccatcagattttctgatctggtcaacgaacagatacagcatacgtttttgatcccgggagagactatatgccgcctcagtgaggtcgtttgactggacgattcgcgggctatttttacgtttcttgtgattgataaccgctgtttccgccatgacagatccatgtgaagtgtgacaagtttttagattgtcacactaaataaaaaagagtcaataagcagggataactttgtgaaaaaacagcttcttctgagggcaatttgtcacagggttaagggcaatttgtcacagACAGGACTGTCATTTGAGGGTGATTTGTCACACTGAAAGGGCAATTTGTCACAACACCTTCTCTAGAACCAGCATGGATAAAGGCCTACAAGGCGCTCTAAAAAAGAAGATCTAAAAACTATAAAAAAAATAATTATAAAAATATCCCCGTGGATAAGTGGATAACCCCAAGGGAAGTTTTTTCAGGCATCGTGTGTAAGCAGAATATATAAGTGCTGTTCCCTGGTGCTTCCTCGCTCACTCGAGGGCTTCGCCCTGTCGCTCAACTGCGGCGAGCACTACTGGCTGTAAaaggacagaccacatcatggttctgtgttcattaggttgttctgtccattgctgacataatccgctccacttcaacgtaacaccgcacgaagatttctattgttcctgaaggcatattcaaatcgttttcgttaccgcttgcaggcatcatgacagaacactacttcctataaacgctacacaggctcctgagattaataatgcggatctctacgataatgggagattttcccgactgtttcgttcgcttctcagtggataacagccagcttctctgtttaacagacaaaaacagcatatccactcagttccacatttccatataaaggccaaggcatttattctcaggataattgtttcagcatcgcaaccgcatcagactccggcatcgcaaactgcacccggtgccgggcagccacatccagcgcaaaaaccttcgtgtagacttccgttgaactgatggacttatgtcccatcaggctttgcagaactttcagcggtataccggcatacagcatgtgcatcgcataggaatggcggaacgtatgtggtgtgaccggaacagagaacgtcacaccgtcagcagcagcggcggcaaccgcctccccaatccaggtcctgaccgttctgtccgtcacttcccagatccgcgctttctctgtccttcctgtgcgacggttacgccgctccatgagcttatcgcgaataaatacctgtgacggaagatcacttcgcagaataaataaatcctggtgtccctgttgataccgggaagccctgggccaacttttggcgaaaatgagacgttgatcggcacgtaagaggttccaactttcaccataatgaaataagatcactaccgggcgtattttttgagttatcgagattttcaggagctaaggaagctaaaatggagaaaaaaatcactggatataccaccgttgatatatcccaatggcatcgtaaagaacattttgaggcatttcagtcagttgctcaatgtacctataaccagaccgttcagctggatattacggcctttttaaagaccgtaaagaaaaataagcacaagttttatccggcctttattcacattcttgcccgcctgatgaatgctcatccggagttccgtatggcaatgaaagacggtgagctggtgatatgggatagtgttcacccttgttacaccgttttccatgagcaaactgaaacgttttcatcgctctggagtgaataccacgacgatttccggcagtttctacacatatattcgcaagatgtggcgtgttacggtgaaaacctggcctatttccctaaagggtttattgagaatatgtttttcgtctcagccaatccctgggtgagtttcaccagttttgatttaaacgtggccaatatggacaacttcttcgcccccgttttcaccatgggcaaatattatacgcaaggcgacaaggtgctgatgccgctggcgattcaggttcatcatgccgtttgtgatggcttccatgtcggcagaatgcttaatgaattacaacagtactgcgatgagtggcagggcggggcgtaatttttttaaggcagttattggtgcccttaaacgcctggttgctacgcctgaataagtgataataagcggatgaatggcagaaattcgatgataagctgtcaaacatgagaattggtcgacggcgcgccaaagcttgcatgcctgcagccgcgtaacctggcaaaatcggttacggttgagtaataaatggatgccctgcgtaagcggggcacatttcattacctctttctccgcacccgacatagataataacttcgtatagtatacattatacgaagttatctagtagacttaattaaggatcgatccggcgcgccaatagtcatgccccgcgcccaccggaaggagctgactgggttgaaggctctcaagggcatcggtcgagcttgacattgtaggactatattgctctaataaatttgcggccgctaatacgactcactatagggagag |
| pRC1783 (Bp2++ EGFP Flag) | gatccgcggaattgTGGTTAATAACAGGGGACGTGGTAATCCGTCCCCTTTTTATTTCTGACTGAGTTAATAACAGGCCTGCTTCGGCAGGCCTTTTTATTTCTGACTGAGTTCTTCTCAGGCCTGCTGGTAATCGCAGGCCTTTTTATTTTCTAGaCAATTCCGACGTCTAAGGAAACCATTATCATGACATCAACCTATAAAAATAGGCGTATCACGAGGCCCTCTCGTCTCCACCTCAAGCTCCCTATCTAGTGATAGCGATTGACATCCCTATCAGTGACGGAGATATTGAGCACATCAGCAGGACGCACTGACCACTTTAAGaaggagATATACATATGGTGAGCAAGGGCGAGGAGCTGTTCACCGGGGTGGTGCCCATCCTGGTCGAGCTGGACGGCGACGTAAACGGCCACAAGTTCAGCGTGCGCGGCGAGGGCGAGGGCGATGCCACCAACGGCAAGCTGACCCTGAAGTTCATCTGCACCACCGGCAAGCTGCCCGTGCCCTGGCCCACCCTCGTGACCACCCTGACCTACGGCGTGCAGTGCTTCAGCCGCTACCCCGACCACATGAAGCAGCACGACTTCTTCAAGTCCGCCATGCCCGAAGGCTACGTCCAGGAGCGCACCATCTCCTTCAAGGACGACGGCACCTACAAGACCCGCGCCGAGGTGAAGTTCGAGGGCGACACCCTGGTGAACCGCATCGAGCTGAAGGGCATCGACTTCAAGGAGGACGGCAACATCCTGGGGCACAAGCTGGAGTACAACTACAACAGCCACAACGTCTATATCACCGCTGACAAGCAGAAGAACGGCATCAAGGCCAACTTCAAGATCCGCCACAACATCGAGGACGGCAGCGTGCAGCTCGCCGACCACTACCAGCAGAACACCCCCATCGGCGACGGCCCCGTGCTGCTGCCCGACAACCACTACCTGAGCACCCAGTCCGCCCTGAGCAAAGACCCCAACGAGAAGCGCGATCACATGGTCCTGCTGGAGTTCGTGACCGCCGCCGGGATCACTCTCGGCATGGACGAGCTGTACAAGTAAGGATCCgactacaaggacgacgatgacaagGAAgattataaagatgatgacgataaaAGAgactacaaggacgacgatgacaagTAAAAGCTTGTTCTTCTCAGGCCTGCTGGTAATCGCAGGCCTTTTTATTTGAATTCcgcggatccttctatagtgtcacctaaatgtcgacggccaggcggccgccaggcctacccactagtcaattcgggaggatcgaaacggcagatcgcaaaaaacagtacatacagaaggagacatgaacatgaacatcaaaaaaattgtaaaacaagccacagttctgacttttacgactgcacttctggcaggaggagcgactcaagccttcgcgaaagaaaataaccaaaaagcatacaaagaaacgtacggcgtctctcatattacacgccatgatatgctgcagatccctaaacagcagcaaaacgaaaaataccaagtgcctcaattcgatcaatcaacgattaaaaatattgagtctgcaaaaggacttgatgtgtgggacagctggccgctgcaaaacgctgacggaacagtagctgaatacaacggctatcacgttgtgtttgctcttgcgggaagcccgaaagacgctgatgacacatcaatctacatgttttatcaaaaggtcggcgacaactcaatcgacagctggaaaaacgcgggccgtgtctttaaagacagcgataagttcgacgccaacgatccgatcctgaaagatcagacgcaagaatggtccggttctgcaacctttacatctgacggaaaaatccgtttattctacactgactattccggtaaacattacggcaaacaaagcctgacaacagcgcaggtaaatgtgtcaaaatctgatgacacactcaaaatcaacggagtggaagatcacaaaacgatttttgacggagacggaaaaacatatcagaacgttcagcagtttatcgatgaaggcaattatacatccggcgacaaccatacgctgagagaccctcactacgttgaagacaaaggccataaataccttgtattcgaagccaacacgggaacagaaaacggataccaaggcgaagaatctttatttaacaaagcgtactacggcggcggcacgaacttcttccgtaaagaaagccagaagcttcagcagagcgctaaaaaacgcgatgctgagttagcgaacggcgccctcggtatcatagagttaaataatgattacacattgaaaaaagtaatgaagccgctgatcacttcaaacacggtaactgatgaaatcgagcgcgcgaatgttttcaaaatgaacggcaaatggtacttgttcactgattcacgcggttcaaaaatgacgatcgatggtattaactcaaacgatatttacatgcttggttatgtatcaaactctttaaccggcccttacaagccgctgaacaaaacagggcttgtgctgcaaatgggtcttgatccaaacgatgtgacattcacttactctcacttcgcagtgccgcaagccaaaggcaacaatgtggttatcacaagctacatgacaaacagaggcttcttcgaggataaaaaggcaacatttgcgccaagcttcttaatgaacatcaaaggcaataaaacatccgttgtcaaaaacagcatcctggagcaaggacagctgacagtcaactaataacagcaaaaagaaaatgccgatacttcattggcattttcttttatttctcaacaagatggtgaattgactagtgggtagatccacaggacgggtgtggtcgccatgatcgcgtagtcgatagtggctccaagtagcgaagcgagcaggactgggcggcggccaaagcggtcggacagtgctccgagaacgggtgcgcatagaaattgcatcaacgcatatagcgctagcagcacgccatagtgactggcgatgctgtcggaatggacgatatcccgcaagaggcccggcagtaccggcataaccaagcctatgcctacagcatccagggtgacggtgccgaggatgacgatgagcgcattgttagatttcatacacggtgcctgactgcgttagcaatttaactgtgataaactaccgcattaaagcttatcgatgataagctgtcaaacatgagaattgatccggaacccttaatataacttcgtataatgtatgctatacgaagttattaggtccctcgactatagggtcaccgtcgacagcgacacacttgcatcggatgcagcccggttaacgtgccggcacggcctgggtaaccaggtattttgtccacataaccgtgcgcaaaatgttgtggataagcaggacacagcagcaatccacagcaggcatacaaccgcacaccgaggttactccgttctacaggttacgacgacatgtcaatacttgcccttgacaggcattgatggaatcgtagtctcacgctgatagtctgatcgacaatacaagtgggaccgtggtcccagaccgataatcagaccgacaacacgagtgggatcgtggtcccagactaataatcagaccgacgatacgagtgggaccgtggtcccagactaataatcagaccgacgatacgagtgggaccgtggttccagactaataatcagaccgacgatacgagtgggaccgtggtcccagactaataatcagaccgacgatacgagtgggaccatggtcccagactaataatcagaccgacgatacgagtgggaccgtggtcccagtctgattatcagaccgacgatacgagtgggaccgtggtcccagactaataatcagaccgacgatacgagtgggaccgtggtcccagactaataatcagaccgacgatacgagtgggaccgtggtcccagtctgattatcagaccgacgatacaagtggaacagtgggcccagagagaatattcaggccagttatgctttctggcctgtaacaaaggacattaagtaaagacagataaacgtagactaaaacgtggtcgcatcagggtgctggcttttcaagttccttaagaatggcctcaattttctctatacactcagttggaacacgagacctgtccaggttaagcaccattttatcgcccttatacaatactgtcgctccaggagcaaactgatgtcgtgagcttaaactagttcttgatgcagatgacgttttaagcacagaagttaaaagagtgataacttcttcagcttcaaatatcaccccagcttttttctgctcatgaaggttagatgcctgctgcttaagtaattcctctttatctgtaaaggctttttgaagtgcatcacctgaccgggcagatagttcaccggggtgagaaaaaagagcaacaactgatttaggcaatttggcggtgttgatacagcgggtaataatcttacgtgaaatattttccgcatcagccagcgcagaaatatttccagcaaattcattctgcaatcggcttgcataacgctgaccacgttcataagcacttgttgggcgataatcgttacccaatctggataatgcagccatctgctcatcatccagctcgccaaccagaacacgataatcactttcggtaagtgcagcagctttacgacggcgactcccatcggcaatttctatgacaccagatactcttcgaccgaacgccggtgtctgttgaccagtcagtagaaaagaagggatgagatcatccagtgcgtcctcagtaagcagctcctggtcacgttcattacctgaccatacccgagaggtcttctcaacactatcaccccggagcacttcaagagtaaacttcacatcccgaccacatacaggcaaagtaatggcattaccgcgagccattactcctacgcgcgcaattaacgaatccaccatcggggcagctggtgtcgataacgaagtatcttcaaccggttgagtattgagcgtatgttttggaataacaggcgcacgcttcattatctaatctcccagcgtggtttaatcagacgatcgaaaatttcattgcagacaggttcccaaatagaaagagcatttctccaggcaccagttgaagagcgttgatcaatggcctgttcaaaaacagttctcatccggatctgacctttaccaacttcatccgtttcacgtacaacattttttagaaccatgcttccccaggcatcccgaatttgctcctccatccacggggactgagagccattactattgctgtatttggtaagcaaaatacgtacatcaggctcgaaccctttaagatcaacgttcttgagcagatcacgaagcatatcgaaaaactgcagtgcggaggtgtagtcaaacaactcagcaggcgtgggaacaatcagcacatcagcagcacatacgacattaatcgtgccgatacccaggttaggcgcgctgtcaataactatgacatcatagtcatgagcaacagtttcaatggccagtcggagcatcaggtgtggatcggtgggcagtttaccttcatcaaatttgcccattaactcagtttcaatacggtgcagagccagacaggaaggaataatgtcaagccccggccagcaagtgggctttattgcataagtgacatcgtccttttccccaagatagaaaggcaggagagtgtcttctgcatgaatatgaagatctggtacccatccgtgatacattgaggctgttccctgggggtcgttaccttccacgagcaaaacacgtagccccttcagagccagatcctgagcaagatgaacagaaactgaggttttgtaaacgccacctttatgggcagcaaccccgatcaccggtggaaatacgtcttcagcacgtcgcaatcgcgtaccaaacacatcacgcatatgattaatttgttcaattgtataaccaacacgttgctcaacccgtcctcgaatttccatatccgggtgcggtagtcgccctgctttctcggcatctctgatagcctgagaagaaaccccaactaaatccgctgcttcacctattctccagcgccgggttattttcctcgcttccgggctgtcatcattaaactgtgcaatggcgatagccttcgtcatttcatgaccagcgtttatgcactggttaagtgtttccatgagtttcattctgaacatcctttaatcattgctttgcgtttttttattaaatcttgcaatttactgcaaagcaacaacaaaatcgcaaagtcatcaaaaaaccgcaaagttgtttaaaataagagcaacactacaaaaggagataagaagagcacatacctcagtcacttattatcactagcgctcgccgcagccgtgtaaccgagcatagcgagcgaactggcgaggaagcaaagaagaactgttctgtcagatagctcttacgctcagcgcaagaagaaatatccaccgtgggaaaaactccaggtagaggtacacacgcggatagccaattcagagtaataaactgtgataatcaaccctcatcaatgatgacgaactaacccccgatatcaggtcacatgacgaagggaaagagaaggaaatcaactgtgacaaactgccctcaaatttggcttccttaaaaattacagttcaaaaagtatgagaaaatccatgcaggctgaaggaaacagcaaaactgtgacaaattaccctcagtaggtcagaacaaatgtgacgaaccaccctcaaatctgtgacagataaccctcagactatcctgtcgtcatggaagtgatatcgcggaaggaaaatacgatatgagtcgtctggcggcctttctttttctcaatgtatgagaggcgcattggagttctgctgttgatctcattaacacagacctgcaggaagcggcggcggaagtcaggcatacgctggtaactttgaggcagctggtaacgctctatgatccagtcgattttcagagagacgatgcctgagccatccggcttacgatactgacacagggattcgtataaacgcatggcatacggattggtgatttcttttgtttcactaagccgaaactgcgtaaaccggttctgtaacccgataaagaagggaatgagatatgggttgatatgtacactgtaaagccctctggatggactgtgcgcacgtttgataaaccaaggaaaagattcatagcctttttcatcgccggcatcctcttcagggcgataaaaaaccacttccttccccgcgaaactcttcaatgcctgccgtatatccttactggcttccgcagaggtcaatccgaatatttcagcatatttagcaacatggatctcgcagataccgtcatgttcctgtagggtgccatcagattttctgatctggtcaacgaacagatacagcatacgtttttgatcccgggagagactatatgccgcctcagtgaggtcgtttgactggacgattcgcgggctatttttacgtttcttgtgattgataaccgctgtttccgccatgacagatccatgtgaagtgtgacaagtttttagattgtcacactaaataaaaaagagtcaataagcagggataactttgtgaaaaaacagcttcttctgagggcaatttgtcacagggttaagggcaatttgtcacagACAGGACTGTCATTTGAGGGTGATTTGTCACACTGAAAGGGCAATTTGTCACAACACCTTCTCTAGAACCAGCATGGATAAAGGCCTACAAGGCGCTCTAAAAAAGAAGATCTAAAAACTATAAAAAAAATAATTATAAAAATATCCCCGTGGATAAGTGGATAACCCCAAGGGAAGTTTTTTCAGGCATCGTGTGTAAGCAGAATATATAAGTGCTGTTCCCTGGTGCTTCCTCGCTCACTCGAGGGCTTCGCCCTGTCGCTCAACTGCGGCGAGCACTACTGGCTGTAAaaggacagaccacatcatggttctgtgttcattaggttgttctgtccattgctgacataatccgctccacttcaacgtaacaccgcacgaagatttctattgttcctgaaggcatattcaaatcgttttcgttaccgcttgcaggcatcatgacagaacactacttcctataaacgctacacaggctcctgagattaataatgcggatctctacgataatgggagattttcccgactgtttcgttcgcttctcagtggataacagccagcttctctgtttaacagacaaaaacagcatatccactcagttccacatttccatataaaggccaaggcatttattctcaggataattgtttcagcatcgcaaccgcatcagactccggcatcgcaaactgcacccggtgccgggcagccacatccagcgcaaaaaccttcgtgtagacttccgttgaactgatggacttatgtcccatcaggctttgcagaactttcagcggtataccggcatacagcatgtgcatcgcataggaatggcggaacgtatgtggtgtgaccggaacagagaacgtcacaccgtcagcagcagcggcggcaaccgcctccccaatccaggtcctgaccgttctgtccgtcacttcccagatccgcgctttctctgtccttcctgtgcgacggttacgccgctccatgagcttatcgcgaataaatacctgtgacggaagatcacttcgcagaataaataaatcctggtgtccctgttgataccgggaagccctgggccaacttttggcgaaaatgagacgttgatcggcacgtaagaggttccaactttcaccataatgaaataagatcactaccgggcgtattttttgagttatcgagattttcaggagctaaggaagctaaaatggagaaaaaaatcactggatataccaccgttgatatatcccaatggcatcgtaaagaacattttgaggcatttcagtcagttgctcaatgtacctataaccagaccgttcagctggatattacggcctttttaaagaccgtaaagaaaaataagcacaagttttatccggcctttattcacattcttgcccgcctgatgaatgctcatccggagttccgtatggcaatgaaagacggtgagctggtgatatgggatagtgttcacccttgttacaccgttttccatgagcaaactgaaacgttttcatcgctctggagtgaataccacgacgatttccggcagtttctacacatatattcgcaagatgtggcgtgttacggtgaaaacctggcctatttccctaaagggtttattgagaatatgtttttcgtctcagccaatccctgggtgagtttcaccagttttgatttaaacgtggccaatatggacaacttcttcgcccccgttttcaccatgggcaaatattatacgcaaggcgacaaggtgctgatgccgctggcgattcaggttcatcatgccgtttgtgatggcttccatgtcggcagaatgcttaatgaattacaacagtactgcgatgagtggcagggcggggcgtaatttttttaaggcagttattggtgcccttaaacgcctggttgctacgcctgaataagtgataataagcggatgaatggcagaaattcgatgataagctgtcaaacatgagaattggtcgacggcgcgccaaagcttgcatgcctgcagccgcgtaacctggcaaaatcggttacggttgagtaataaatggatgccctgcgtaagcggggcacatttcattacctctttctccgcacccgacatagataataacttcgtatagtatacattatacgaagttatctagtagacttaattaaggatcgatccggcgcgccaatagtcatgccccgcgcccaccggaaggagctgactgggttgaaggctctcaagggcatcggtcgagcttgacattgtaggactatattgctctaataaatttgcggccgctaatacgactcactatagggagag |
| pRC1784  (Bp3++ EGFP Flag) | gatccgcggaattgTGGTTAATAACAGGGGACGTGGTAATCCGTCCCCTTTTTATTTCTGACTGAGTTAATAACAGGCCTGCTTCGGCAGGCCTTTTTATTTCTGACTGAGTTCTTCTCAGGCCTGCTGGTAATCGCAGGCCTTTTTATTTTCTAGaCAATTCCGACGTCTAAGAAACCATTATTATCATGACATTAACCTATAAAAATAGGCGTATCACGAGGCCCTTTCGTCTTCACCTCGAGTCCCTATCAGTGATAGAGATTGACCTCCCTATCAGTGATAGAGATACTGAGCACATCAGCAGGACGCACTGACCACTTTAAGAAGGAGATATACATATGGTGAGCAAGGGCGAGGAGCTGTTCACCGGGGTGGTGCCCATCCTGGTCGAGCTGGACGGCGACGTAAACGGCCACAAGTTCAGCGTGCGCGGCGAGGGCGAGGGCGATGCCACCAACGGCAAGCTGACCCTGAAGTTCATCTGCACCACCGGCAAGCTGCCCGTGCCCTGGCCCACCCTCGTGACCACCCTGACCTACGGCGTGCAGTGCTTCAGCCGCTACCCCGACCACATGAAGCAGCACGACTTCTTCAAGTCCGCCATGCCCGAAGGCTACGTCCAGGAGCGCACCATCTCCTTCAAGGACGACGGCACCTACAAGACCCGCGCCGAGGTGAAGTTCGAGGGCGACACCCTGGTGAACCGCATCGAGCTGAAGGGCATCGACTTCAAGGAGGACGGCAACATCCTGGGGCACAAGCTGGAGTACAACTACAACAGCCACAACGTCTATATCACCGCTGACAAGCAGAAGAACGGCATCAAGGCCAACTTCAAGATCCGCCACAACATCGAGGACGGCAGCGTGCAGCTCGCCGACCACTACCAGCAGAACACCCCCATCGGCGACGGCCCCGTGCTGCTGCCCGACAACCACTACCTGAGCACCCAGTCCGCCCTGAGCAAAGACCCCAACGAGAAGCGCGATCACATGGTCCTGCTGGAGTTCGTGACCGCCGCCGGGATCACTCTCGGCATGGACGAGCTGTACAAGTAAGGATCCgactacaaggacgacgatgacaagGAAgattataaagatgatgacgataaaAGAgactacaaggacgacgatgacaagTAAAAGCTTGTTCTTCTCAGGCCTGCTGGTAATCGCAGGCCTTTTTATTTGAATTCcgcggatccttctatagtgtcacctaaatgtcgacggccaggcggccgccaggcctacccactagtcaattcgggaggatcgaaacggcagatcgcaaaaaacagtacatacagaaggagacatgaacatgaacatcaaaaaaattgtaaaacaagccacagttctgacttttacgactgcacttctggcaggaggagcgactcaagccttcgcgaaagaaaataaccaaaaagcatacaaagaaacgtacggcgtctctcatattacacgccatgatatgctgcagatccctaaacagcagcaaaacgaaaaataccaagtgcctcaattcgatcaatcaacgattaaaaatattgagtctgcaaaaggacttgatgtgtgggacagctggccgctgcaaaacgctgacggaacagtagctgaatacaacggctatcacgttgtgtttgctcttgcgggaagcccgaaagacgctgatgacacatcaatctacatgttttatcaaaaggtcggcgacaactcaatcgacagctggaaaaacgcgggccgtgtctttaaagacagcgataagttcgacgccaacgatccgatcctgaaagatcagacgcaagaatggtccggttctgcaacctttacatctgacggaaaaatccgtttattctacactgactattccggtaaacattacggcaaacaaagcctgacaacagcgcaggtaaatgtgtcaaaatctgatgacacactcaaaatcaacggagtggaagatcacaaaacgatttttgacggagacggaaaaacatatcagaacgttcagcagtttatcgatgaaggcaattatacatccggcgacaaccatacgctgagagaccctcactacgttgaagacaaaggccataaataccttgtattcgaagccaacacgggaacagaaaacggataccaaggcgaagaatctttatttaacaaagcgtactacggcggcggcacgaacttcttccgtaaagaaagccagaagcttcagcagagcgctaaaaaacgcgatgctgagttagcgaacggcgccctcggtatcatagagttaaataatgattacacattgaaaaaagtaatgaagccgctgatcacttcaaacacggtaactgatgaaatcgagcgcgcgaatgttttcaaaatgaacggcaaatggtacttgttcactgattcacgcggttcaaaaatgacgatcgatggtattaactcaaacgatatttacatgcttggttatgtatcaaactctttaaccggcccttacaagccgctgaacaaaacagggcttgtgctgcaaatgggtcttgatccaaacgatgtgacattcacttactctcacttcgcagtgccgcaagccaaaggcaacaatgtggttatcacaagctacatgacaaacagaggcttcttcgaggataaaaaggcaacatttgcgccaagcttcttaatgaacatcaaaggcaataaaacatccgttgtcaaaaacagcatcctggagcaaggacagctgacagtcaactaataacagcaaaaagaaaatgccgatacttcattggcattttcttttatttctcaacaagatggtgaattgactagtgggtagatccacaggacgggtgtggtcgccatgatcgcgtagtcgatagtggctccaagtagcgaagcgagcaggactgggcggcggccaaagcggtcggacagtgctccgagaacgggtgcgcatagaaattgcatcaacgcatatagcgctagcagcacgccatagtgactggcgatgctgtcggaatggacgatatcccgcaagaggcccggcagtaccggcataaccaagcctatgcctacagcatccagggtgacggtgccgaggatgacgatgagcgcattgttagatttcatacacggtgcctgactgcgttagcaatttaactgtgataaactaccgcattaaagcttatcgatgataagctgtcaaacatgagaattgatccggaacccttaatataacttcgtataatgtatgctatacgaagttattaggtccctcgactatagggtcaccgtcgacagcgacacacttgcatcggatgcagcccggttaacgtgccggcacggcctgggtaaccaggtattttgtccacataaccgtgcgcaaaatgttgtggataagcaggacacagcagcaatccacagcaggcatacaaccgcacaccgaggttactccgttctacaggttacgacgacatgtcaatacttgcccttgacaggcattgatggaatcgtagtctcacgctgatagtctgatcgacaatacaagtgggaccgtggtcccagaccgataatcagaccgacaacacgagtgggatcgtggtcccagactaataatcagaccgacgatacgagtgggaccgtggtcccagactaataatcagaccgacgatacgagtgggaccgtggttccagactaataatcagaccgacgatacgagtgggaccgtggtcccagactaataatcagaccgacgatacgagtgggaccatggtcccagactaataatcagaccgacgatacgagtgggaccgtggtcccagtctgattatcagaccgacgatacgagtgggaccgtggtcccagactaataatcagaccgacgatacgagtgggaccgtggtcccagactaataatcagaccgacgatacgagtgggaccgtggtcccagtctgattatcagaccgacgatacaagtggaacagtgggcccagagagaatattcaggccagttatgctttctggcctgtaacaaaggacattaagtaaagacagataaacgtagactaaaacgtggtcgcatcagggtgctggcttttcaagttccttaagaatggcctcaattttctctatacactcagttggaacacgagacctgtccaggttaagcaccattttatcgcccttatacaatactgtcgctccaggagcaaactgatgtcgtgagcttaaactagttcttgatgcagatgacgttttaagcacagaagttaaaagagtgataacttcttcagcttcaaatatcaccccagcttttttctgctcatgaaggttagatgcctgctgcttaagtaattcctctttatctgtaaaggctttttgaagtgcatcacctgaccgggcagatagttcaccggggtgagaaaaaagagcaacaactgatttaggcaatttggcggtgttgatacagcgggtaataatcttacgtgaaatattttccgcatcagccagcgcagaaatatttccagcaaattcattctgcaatcggcttgcataacgctgaccacgttcataagcacttgttgggcgataatcgttacccaatctggataatgcagccatctgctcatcatccagctcgccaaccagaacacgataatcactttcggtaagtgcagcagctttacgacggcgactcccatcggcaatttctatgacaccagatactcttcgaccgaacgccggtgtctgttgaccagtcagtagaaaagaagggatgagatcatccagtgcgtcctcagtaagcagctcctggtcacgttcattacctgaccatacccgagaggtcttctcaacactatcaccccggagcacttcaagagtaaacttcacatcccgaccacatacaggcaaagtaatggcattaccgcgagccattactcctacgcgcgcaattaacgaatccaccatcggggcagctggtgtcgataacgaagtatcttcaaccggttgagtattgagcgtatgttttggaataacaggcgcacgcttcattatctaatctcccagcgtggtttaatcagacgatcgaaaatttcattgcagacaggttcccaaatagaaagagcatttctccaggcaccagttgaagagcgttgatcaatggcctgttcaaaaacagttctcatccggatctgacctttaccaacttcatccgtttcacgtacaacattttttagaaccatgcttccccaggcatcccgaatttgctcctccatccacggggactgagagccattactattgctgtatttggtaagcaaaatacgtacatcaggctcgaaccctttaagatcaacgttcttgagcagatcacgaagcatatcgaaaaactgcagtgcggaggtgtagtcaaacaactcagcaggcgtgggaacaatcagcacatcagcagcacatacgacattaatcgtgccgatacccaggttaggcgcgctgtcaataactatgacatcatagtcatgagcaacagtttcaatggccagtcggagcatcaggtgtggatcggtgggcagtttaccttcatcaaatttgcccattaactcagtttcaatacggtgcagagccagacaggaaggaataatgtcaagccccggccagcaagtgggctttattgcataagtgacatcgtccttttccccaagatagaaaggcaggagagtgtcttctgcatgaatatgaagatctggtacccatccgtgatacattgaggctgttccctgggggtcgttaccttccacgagcaaaacacgtagccccttcagagccagatcctgagcaagatgaacagaaactgaggttttgtaaacgccacctttatgggcagcaaccccgatcaccggtggaaatacgtcttcagcacgtcgcaatcgcgtaccaaacacatcacgcatatgattaatttgttcaattgtataaccaacacgttgctcaacccgtcctcgaatttccatatccgggtgcggtagtcgccctgctttctcggcatctctgatagcctgagaagaaaccccaactaaatccgctgcttcacctattctccagcgccgggttattttcctcgcttccgggctgtcatcattaaactgtgcaatggcgatagccttcgtcatttcatgaccagcgtttatgcactggttaagtgtttccatgagtttcattctgaacatcctttaatcattgctttgcgtttttttattaaatcttgcaatttactgcaaagcaacaacaaaatcgcaaagtcatcaaaaaaccgcaaagttgtttaaaataagagcaacactacaaaaggagataagaagagcacatacctcagtcacttattatcactagcgctcgccgcagccgtgtaaccgagcatagcgagcgaactggcgaggaagcaaagaagaactgttctgtcagatagctcttacgctcagcgcaagaagaaatatccaccgtgggaaaaactccaggtagaggtacacacgcggatagccaattcagagtaataaactgtgataatcaaccctcatcaatgatgacgaactaacccccgatatcaggtcacatgacgaagggaaagagaaggaaatcaactgtgacaaactgccctcaaatttggcttccttaaaaattacagttcaaaaagtatgagaaaatccatgcaggctgaaggaaacagcaaaactgtgacaaattaccctcagtaggtcagaacaaatgtgacgaaccaccctcaaatctgtgacagataaccctcagactatcctgtcgtcatggaagtgatatcgcggaaggaaaatacgatatgagtcgtctggcggcctttctttttctcaatgtatgagaggcgcattggagttctgctgttgatctcattaacacagacctgcaggaagcggcggcggaagtcaggcatacgctggtaactttgaggcagctggtaacgctctatgatccagtcgattttcagagagacgatgcctgagccatccggcttacgatactgacacagggattcgtataaacgcatggcatacggattggtgatttcttttgtttcactaagccgaaactgcgtaaaccggttctgtaacccgataaagaagggaatgagatatgggttgatatgtacactgtaaagccctctggatggactgtgcgcacgtttgataaaccaaggaaaagattcatagcctttttcatcgccggcatcctcttcagggcgataaaaaaccacttccttccccgcgaaactcttcaatgcctgccgtatatccttactggcttccgcagaggtcaatccgaatatttcagcatatttagcaacatggatctcgcagataccgtcatgttcctgtagggtgccatcagattttctgatctggtcaacgaacagatacagcatacgtttttgatcccgggagagactatatgccgcctcagtgaggtcgtttgactggacgattcgcgggctatttttacgtttcttgtgattgataaccgctgtttccgccatgacagatccatgtgaagtgtgacaagtttttagattgtcacactaaataaaaaagagtcaataagcagggataactttgtgaaaaaacagcttcttctgagggcaatttgtcacagggttaagggcaatttgtcacagACAGGACTGTCATTTGAGGGTGATTTGTCACACTGAAAGGGCAATTTGTCACAACACCTTCTCTAGAACCAGCATGGATAAAGGCCTACAAGGCGCTCTAAAAAAGAAGATCTAAAAACTATAAAAAAAATAATTATAAAAATATCCCCGTGGATAAGTGGATAACCCCAAGGGAAGTTTTTTCAGGCATCGTGTGTAAGCAGAATATATAAGTGCTGTTCCCTGGTGCTTCCTCGCTCACTCGAGGGCTTCGCCCTGTCGCTCAACTGCGGCGAGCACTACTGGCTGTAAaaggacagaccacatcatggttctgtgttcattaggttgttctgtccattgctgacataatccgctccacttcaacgtaacaccgcacgaagatttctattgttcctgaaggcatattcaaatcgttttcgttaccgcttgcaggcatcatgacagaacactacttcctataaacgctacacaggctcctgagattaataatgcggatctctacgataatgggagattttcccgactgtttcgttcgcttctcagtggataacagccagcttctctgtttaacagacaaaaacagcatatccactcagttccacatttccatataaaggccaaggcatttattctcaggataattgtttcagcatcgcaaccgcatcagactccggcatcgcaaactgcacccggtgccgggcagccacatccagcgcaaaaaccttcgtgtagacttccgttgaactgatggacttatgtcccatcaggctttgcagaactttcagcggtataccggcatacagcatgtgcatcgcataggaatggcggaacgtatgtggtgtgaccggaacagagaacgtcacaccgtcagcagcagcggcggcaaccgcctccccaatccaggtcctgaccgttctgtccgtcacttcccagatccgcgctttctctgtccttcctgtgcgacggttacgccgctccatgagcttatcgcgaataaatacctgtgacggaagatcacttcgcagaataaataaatcctggtgtccctgttgataccgggaagccctgggccaacttttggcgaaaatgagacgttgatcggcacgtaagaggttccaactttcaccataatgaaataagatcactaccgggcgtattttttgagttatcgagattttcaggagctaaggaagctaaaatggagaaaaaaatcactggatataccaccgttgatatatcccaatggcatcgtaaagaacattttgaggcatttcagtcagttgctcaatgtacctataaccagaccgttcagctggatattacggcctttttaaagaccgtaaagaaaaataagcacaagttttatccggcctttattcacattcttgcccgcctgatgaatgctcatccggagttccgtatggcaatgaaagacggtgagctggtgatatgggatagtgttcacccttgttacaccgttttccatgagcaaactgaaacgttttcatcgctctggagtgaataccacgacgatttccggcagtttctacacatatattcgcaagatgtggcgtgttacggtgaaaacctggcctatttccctaaagggtttattgagaatatgtttttcgtctcagccaatccctgggtgagtttcaccagttttgatttaaacgtggccaatatggacaacttcttcgcccccgttttcaccatgggcaaatattatacgcaaggcgacaaggtgctgatgccgctggcgattcaggttcatcatgccgtttgtgatggcttccatgtcggcagaatgcttaatgaattacaacagtactgcgatgagtggcagggcggggcgtaatttttttaaggcagttattggtgcccttaaacgcctggttgctacgcctgaataagtgataataagcggatgaatggcagaaattcgatgataagctgtcaaacatgagaattggtcgacggcgcgccaaagcttgcatgcctgcagccgcgtaacctggcaaaatcggttacggttgagtaataaatggatgccctgcgtaagcggggcacatttcattacctctttctccgcacccgacatagataataacttcgtatagtatacattatacgaagttatctagtagacttaattaaggatcgatccggcgcgccaatagtcatgccccgcgcccaccggaaggagctgactgggttgaaggctctcaagggcatcggtcgagcttgacattgtaggactatattgctctaataaatttgcggccgctaatacgactcactatagggagag |
| pRC1785  (Bp4++ EGFP Flag) | gatccgcggaattgTGGTTAATAACAGGGGACGTGGTAATCCGTCCCCTTTTTATTTCTGACTGAGTTAATAACAGGCCTGCTTCGGCAGGCCTTTTTATTTCTGACTGAGTTCTTCTCAGGCCTGCTGGTAATCGCAGGCCTTTTTATTTTCTAGgCAATTCCGACGTCTAAGAAACCATTATTATCATGACATTAACCTATAAAAATAGGCGTATCACGAGGCCCTCTCGTCTTCACCTCGAGTCCCTATCAGTGATAGGGATTGACATCCCTATCAGTGATAGAGACACTGGGCACATCAGCAGGACGCACTGACCACTTTAAGAAGGAGATATACATATGGTGAGCAAGGGCGAGGAGCTGTTCACCGGGGTGGTGCCCATCCTGGTCGAGCTGGACGGCGACGTAAACGGCCACAAGTTCAGCGTGCGCGGCGAGGGCGAGGGCGATGCCACCAACGGCAAGCTGACCCTGAAGTTCATCTGCACCACCGGCAAGCTGCCCGTGCCCTGGCCCACCCTCGTGACCACCCTGACCTACGGCGTGCAGTGCTTCAGCCGCTACCCCGACCACATGAAGCAGCACGACTTCTTCAAGTCCGCCATGCCCGAAGGCTACGTCCAGGAGCGCACCATCTCCTTCAAGGACGACGGCACCTACAAGACCCGCGCCGAGGTGAAGTTCGAGGGCGACACCCTGGTGAACCGCATCGAGCTGAAGGGCATCGACTTCAAGGAGGACGGCAACATCCTGGGGCACAAGCTGGAGTACAACTACAACAGCCACAACGTCTATATCACCGCTGACAAGCAGAAGAACGGCATCAAGGCCAACTTCAAGATCCGCCACAACATCGAGGACGGCAGCGTGCAGCTCGCCGACCACTACCAGCAGAACACCCCCATCGGCGACGGCCCCGTGCTGCTGCCCGACAACCACTACCTGAGCACCCAGTCCGCCCTGAGCAAAGACCCCAACGAGAAGCGCGATCACATGGTCCTGCTGGAGTTCGTGACCGCCGCCGGGATCACTCTCGGCATGGACGAGCTGTACAAGTAAGGATCCgactacaaggacgacgatgacaagGAAgattataaagatgatgacgataaaAGAgactacaaggacgacgatgacaagTAAAAGCTTGTTCTTCTCAGGCCTGCTGGTAATCGCAGGCCTTTTTATTTGAATTCcgcggatccttctatagtgtcacctaaatgtcgacggccaggcggccgccaggcctacccactagtcaattcgggaggatcgaaacggcagatcgcaaaaaacagtacatacagaaggagacatgaacatgaacatcaaaaaaattgtaaaacaagccacagttctgacttttacgactgcacttctggcaggaggagcgactcaagccttcgcgaaagaaaataaccaaaaagcatacaaagaaacgtacggcgtctctcatattacacgccatgatatgctgcagatccctaaacagcagcaaaacgaaaaataccaagtgcctcaattcgatcaatcaacgattaaaaatattgagtctgcaaaaggacttgatgtgtgggacagctggccgctgcaaaacgctgacggaacagtagctgaatacaacggctatcacgttgtgtttgctcttgcgggaagcccgaaagacgctgatgacacatcaatctacatgttttatcaaaaggtcggcgacaactcaatcgacagctggaaaaacgcgggccgtgtctttaaagacagcgataagttcgacgccaacgatccgatcctgaaagatcagacgcaagaatggtccggttctgcaacctttacatctgacggaaaaatccgtttattctacactgactattccggtaaacattacggcaaacaaagcctgacaacagcgcaggtaaatgtgtcaaaatctgatgacacactcaaaatcaacggagtggaagatcacaaaacgatttttgacggagacggaaaaacatatcagaacgttcagcagtttatcgatgaaggcaattatacatccggcgacaaccatacgctgagagaccctcactacgttgaagacaaaggccataaataccttgtattcgaagccaacacgggaacagaaaacggataccaaggcgaagaatctttatttaacaaagcgtactacggcggcggcacgaacttcttccgtaaagaaagccagaagcttcagcagagcgctaaaaaacgcgatgctgagttagcgaacggcgccctcggtatcatagagttaaataatgattacacattgaaaaaagtaatgaagccgctgatcacttcaaacacggtaactgatgaaatcgagcgcgcgaatgttttcaaaatgaacggcaaatggtacttgttcactgattcacgcggttcaaaaatgacgatcgatggtattaactcaaacgatatttacatgcttggttatgtatcaaactctttaaccggcccttacaagccgctgaacaaaacagggcttgtgctgcaaatgggtcttgatccaaacgatgtgacattcacttactctcacttcgcagtgccgcaagccaaaggcaacaatgtggttatcacaagctacatgacaaacagaggcttcttcgaggataaaaaggcaacatttgcgccaagcttcttaatgaacatcaaaggcaataaaacatccgttgtcaaaaacagcatcctggagcaaggacagctgacagtcaactaataacagcaaaaagaaaatgccgatacttcattggcattttcttttatttctcaacaagatggtgaattgactagtgggtagatccacaggacgggtgtggtcgccatgatcgcgtagtcgatagtggctccaagtagcgaagcgagcaggactgggcggcggccaaagcggtcggacagtgctccgagaacgggtgcgcatagaaattgcatcaacgcatatagcgctagcagcacgccatagtgactggcgatgctgtcggaatggacgatatcccgcaagaggcccggcagtaccggcataaccaagcctatgcctacagcatccagggtgacggtgccgaggatgacgatgagcgcattgttagatttcatacacggtgcctgactgcgttagcaatttaactgtgataaactaccgcattaaagcttatcgatgataagctgtcaaacatgagaattgatccggaacccttaatataacttcgtataatgtatgctatacgaagttattaggtccctcgactatagggtcaccgtcgacagcgacacacttgcatcggatgcagcccggttaacgtgccggcacggcctgggtaaccaggtattttgtccacataaccgtgcgcaaaatgttgtggataagcaggacacagcagcaatccacagcaggcatacaaccgcacaccgaggttactccgttctacaggttacgacgacatgtcaatacttgcccttgacaggcattgatggaatcgtagtctcacgctgatagtctgatcgacaatacaagtgggaccgtggtcccagaccgataatcagaccgacaacacgagtgggatcgtggtcccagactaataatcagaccgacgatacgagtgggaccgtggtcccagactaataatcagaccgacgatacgagtgggaccgtggttccagactaataatcagaccgacgatacgagtgggaccgtggtcccagactaataatcagaccgacgatacgagtgggaccatggtcccagactaataatcagaccgacgatacgagtgggaccgtggtcccagtctgattatcagaccgacgatacgagtgggaccgtggtcccagactaataatcagaccgacgatacgagtgggaccgtggtcccagactaataatcagaccgacgatacgagtgggaccgtggtcccagtctgattatcagaccgacgatacaagtggaacagtgggcccagagagaatattcaggccagttatgctttctggcctgtaacaaaggacattaagtaaagacagataaacgtagactaaaacgtggtcgcatcagggtgctggcttttcaagttccttaagaatggcctcaattttctctatacactcagttggaacacgagacctgtccaggttaagcaccattttatcgcccttatacaatactgtcgctccaggagcaaactgatgtcgtgagcttaaactagttcttgatgcagatgacgttttaagcacagaagttaaaagagtgataacttcttcagcttcaaatatcaccccagcttttttctgctcatgaaggttagatgcctgctgcttaagtaattcctctttatctgtaaaggctttttgaagtgcatcacctgaccgggcagatagttcaccggggtgagaaaaaagagcaacaactgatttaggcaatttggcggtgttgatacagcgggtaataatcttacgtgaaatattttccgcatcagccagcgcagaaatatttccagcaaattcattctgcaatcggcttgcataacgctgaccacgttcataagcacttgttgggcgataatcgttacccaatctggataatgcagccatctgctcatcatccagctcgccaaccagaacacgataatcactttcggtaagtgcagcagctttacgacggcgactcccatcggcaatttctatgacaccagatactcttcgaccgaacgccggtgtctgttgaccagtcagtagaaaagaagggatgagatcatccagtgcgtcctcagtaagcagctcctggtcacgttcattacctgaccatacccgagaggtcttctcaacactatcaccccggagcacttcaagagtaaacttcacatcccgaccacatacaggcaaagtaatggcattaccgcgagccattactcctacgcgcgcaattaacgaatccaccatcggggcagctggtgtcgataacgaagtatcttcaaccggttgagtattgagcgtatgttttggaataacaggcgcacgcttcattatctaatctcccagcgtggtttaatcagacgatcgaaaatttcattgcagacaggttcccaaatagaaagagcatttctccaggcaccagttgaagagcgttgatcaatggcctgttcaaaaacagttctcatccggatctgacctttaccaacttcatccgtttcacgtacaacattttttagaaccatgcttccccaggcatcccgaatttgctcctccatccacggggactgagagccattactattgctgtatttggtaagcaaaatacgtacatcaggctcgaaccctttaagatcaacgttcttgagcagatcacgaagcatatcgaaaaactgcagtgcggaggtgtagtcaaacaactcagcaggcgtgggaacaatcagcacatcagcagcacatacgacattaatcgtgccgatacccaggttaggcgcgctgtcaataactatgacatcatagtcatgagcaacagtttcaatggccagtcggagcatcaggtgtggatcggtgggcagtttaccttcatcaaatttgcccattaactcagtttcaatacggtgcagagccagacaggaaggaataatgtcaagccccggccagcaagtgggctttattgcataagtgacatcgtccttttccccaagatagaaaggcaggagagtgtcttctgcatgaatatgaagatctggtacccatccgtgatacattgaggctgttccctgggggtcgttaccttccacgagcaaaacacgtagccccttcagagccagatcctgagcaagatgaacagaaactgaggttttgtaaacgccacctttatgggcagcaaccccgatcaccggtggaaatacgtcttcagcacgtcgcaatcgcgtaccaaacacatcacgcatatgattaatttgttcaattgtataaccaacacgttgctcaacccgtcctcgaatttccatatccgggtgcggtagtcgccctgctttctcggcatctctgatagcctgagaagaaaccccaactaaatccgctgcttcacctattctccagcgccgggttattttcctcgcttccgggctgtcatcattaaactgtgcaatggcgatagccttcgtcatttcatgaccagcgtttatgcactggttaagtgtttccatgagtttcattctgaacatcctttaatcattgctttgcgtttttttattaaatcttgcaatttactgcaaagcaacaacaaaatcgcaaagtcatcaaaaaaccgcaaagttgtttaaaataagagcaacactacaaaaggagataagaagagcacatacctcagtcacttattatcactagcgctcgccgcagccgtgtaaccgagcatagcgagcgaactggcgaggaagcaaagaagaactgttctgtcagatagctcttacgctcagcgcaagaagaaatatccaccgtgggaaaaactccaggtagaggtacacacgcggatagccaattcagagtaataaactgtgataatcaaccctcatcaatgatgacgaactaacccccgatatcaggtcacatgacgaagggaaagagaaggaaatcaactgtgacaaactgccctcaaatttggcttccttaaaaattacagttcaaaaagtatgagaaaatccatgcaggctgaaggaaacagcaaaactgtgacaaattaccctcagtaggtcagaacaaatgtgacgaaccaccctcaaatctgtgacagataaccctcagactatcctgtcgtcatggaagtgatatcgcggaaggaaaatacgatatgagtcgtctggcggcctttctttttctcaatgtatgagaggcgcattggagttctgctgttgatctcattaacacagacctgcaggaagcggcggcggaagtcaggcatacgctggtaactttgaggcagctggtaacgctctatgatccagtcgattttcagagagacgatgcctgagccatccggcttacgatactgacacagggattcgtataaacgcatggcatacggattggtgatttcttttgtttcactaagccgaaactgcgtaaaccggttctgtaacccgataaagaagggaatgagatatgggttgatatgtacactgtaaagccctctggatggactgtgcgcacgtttgataaaccaaggaaaagattcatagcctttttcatcgccggcatcctcttcagggcgataaaaaaccacttccttccccgcgaaactcttcaatgcctgccgtatatccttactggcttccgcagaggtcaatccgaatatttcagcatatttagcaacatggatctcgcagataccgtcatgttcctgtagggtgccatcagattttctgatctggtcaacgaacagatacagcatacgtttttgatcccgggagagactatatgccgcctcagtgaggtcgtttgactggacgattcgcgggctatttttacgtttcttgtgattgataaccgctgtttccgccatgacagatccatgtgaagtgtgacaagtttttagattgtcacactaaataaaaaagagtcaataagcagggataactttgtgaaaaaacagcttcttctgagggcaatttgtcacagggttaagggcaatttgtcacagACAGGACTGTCATTTGAGGGTGATTTGTCACACTGAAAGGGCAATTTGTCACAACACCTTCTCTAGAACCAGCATGGATAAAGGCCTACAAGGCGCTCTAAAAAAGAAGATCTAAAAACTATAAAAAAAATAATTATAAAAATATCCCCGTGGATAAGTGGATAACCCCAAGGGAAGTTTTTTCAGGCATCGTGTGTAAGCAGAATATATAAGTGCTGTTCCCTGGTGCTTCCTCGCTCACTCGAGGGCTTCGCCCTGTCGCTCAACTGCGGCGAGCACTACTGGCTGTAAaaggacagaccacatcatggttctgtgttcattaggttgttctgtccattgctgacataatccgctccacttcaacgtaacaccgcacgaagatttctattgttcctgaaggcatattcaaatcgttttcgttaccgcttgcaggcatcatgacagaacactacttcctataaacgctacacaggctcctgagattaataatgcggatctctacgataatgggagattttcccgactgtttcgttcgcttctcagtggataacagccagcttctctgtttaacagacaaaaacagcatatccactcagttccacatttccatataaaggccaaggcatttattctcaggataattgtttcagcatcgcaaccgcatcagactccggcatcgcaaactgcacccggtgccgggcagccacatccagcgcaaaaaccttcgtgtagacttccgttgaactgatggacttatgtcccatcaggctttgcagaactttcagcggtataccggcatacagcatgtgcatcgcataggaatggcggaacgtatgtggtgtgaccggaacagagaacgtcacaccgtcagcagcagcggcggcaaccgcctccccaatccaggtcctgaccgttctgtccgtcacttcccagatccgcgctttctctgtccttcctgtgcgacggttacgccgctccatgagcttatcgcgaataaatacctgtgacggaagatcacttcgcagaataaataaatcctggtgtccctgttgataccgggaagccctgggccaacttttggcgaaaatgagacgttgatcggcacgtaagaggttccaactttcaccataatgaaataagatcactaccgggcgtattttttgagttatcgagattttcaggagctaaggaagctaaaatggagaaaaaaatcactggatataccaccgttgatatatcccaatggcatcgtaaagaacattttgaggcatttcagtcagttgctcaatgtacctataaccagaccgttcagctggatattacggcctttttaaagaccgtaaagaaaaataagcacaagttttatccggcctttattcacattcttgcccgcctgatgaatgctcatccggagttccgtatggcaatgaaagacggtgagctggtgatatgggatagtgttcacccttgttacaccgttttccatgagcaaactgaaacgttttcatcgctctggagtgaataccacgacgatttccggcagtttctacacatatattcgcaagatgtggcgtgttacggtgaaaacctggcctatttccctaaagggtttattgagaatatgtttttcgtctcagccaatccctgggtgagtttcaccagttttgatttaaacgtggccaatatggacaacttcttcgcccccgttttcaccatgggcaaatattatacgcaaggcgacaaggtgctgatgccgctggcgattcaggttcatcatgccgtttgtgatggcttccatgtcggcagaatgcttaatgaattacaacagtactgcgatgagtggcagggcggggcgtaatttttttaaggcagttattggtgcccttaaacgcctggttgctacgcctgaataagtgataataagcggatgaatggcagaaattcgatgataagctgtcaaacatgagaattggtcgacggcgcgccaaagcttgcatgcctgcagccgcgtaacctggcaaaatcggttacggttgagtaataaatggatgccctgcgtaagcggggcacatttcattacctctttctccgcacccgacatagataataacttcgtatagtatacattatacgaagttatctagtagacttaattaaggatcgatccggcgcgccaatagtcatgccccgcgcccaccggaaggagctgactgggttgaaggctctcaagggcatcggtcgagcttgacattgtaggactatattgctctaataaatttgcggccgctaatacgactcactatagggagag |
| pRC1786  (Bp5++ EGFP Flag) | gatccgcggaattgGTTAATAACAGGGGACGTGGTAATCCGTCCCCTTTTTATTTCTGACTGAGTTAATAACAGGCCTGCTTCGGCAGGCCTTTTTATTTCTGACTGAGTTCTTCTCAGGCCTGCTGGTAATCGCAGGCCTTTTTATTTTCTAGcCAATTCCGACGCCTAAGAAACCATTATTATCATGACATTAGCCTATAAAAATAGGCGTACCACGAGGCCCTTTCGTCTTCACCTCGAGTCCCTATCAGTGATAGAGATTGACACCCCTATCAGTGATAGAGATACTGAGCACATCAGCAGGACGCACTGACCACTTTAAGAAGGAGATATACATATGGTGAGCAAGGGCGAGGAGCTGTTCACCGGGGTGGTGCCCATCCTGGTCGAGCTGGACGGCGACGTAAACGGCCACAAGTTCAGCGTGCGCGGCGAGGGCGAGGGCGATGCCACCAACGGCAAGCTGACCCTGAAGTTCATCTGCACCACCGGCAAGCTGCCCGTGCCCTGGCCCACCCTCGTGACCACCCTGACCTACGGCGTGCAGTGCTTCAGCCGCTACCCCGACCACATGAAGCAGCACGACTTCTTCAAGTCCGCCATGCCCGAAGGCTACGTCCAGGAGCGCACCATCTCCTTCAAGGACGACGGCACCTACAAGACCCGCGCCGAGGTGAAGTTCGAGGGCGACACCCTGGTGAACCGCATCGAGCTGAAGGGCATCGACTTCAAGGAGGACGGCAACATCCTGGGGCACAAGCTGGAGTACAACTACAACAGCCACAACGTCTATATCACCGCTGACAAGCAGAAGAACGGCATCAAGGCCAACTTCAAGATCCGCCACAACATCGAGGACGGCAGCGTGCAGCTCGCCGACCACTACCAGCAGAACACCCCCATCGGCGACGGCCCCGTGCTGCTGCCCGACAACCACTACCTGAGCACCCAGTCCGCCCTGAGCAAAGACCCCAACGAGAAGCGCGATCACATGGTCCTGCTGGAGTTCGTGACCGCCGCCGGGATCACTCTCGGCATGGACGAGCTGTACAAGTAAGGATCCgactacaaggacgacgatgacaagGAAgattataaagatgatgacgataaaAGAgactacaaggacgacgatgacaagTAAAAGCTTGTTCTTCTCAGGCCTGCTGGTAATCGCAGGCCTTTTTATTTGAATTCcgcggatccttctatagtgtcacctaaatgtcgacggccaggcggccgccaggcctacccactagtcaattcgggaggatcgaaacggcagatcgcaaaaaacagtacatacagaaggagacatgaacatgaacatcaaaaaaattgtaaaacaagccacagttctgacttttacgactgcacttctggcaggaggagcgactcaagccttcgcgaaagaaaataaccaaaaagcatacaaagaaacgtacggcgtctctcatattacacgccatgatatgctgcagatccctaaacagcagcaaaacgaaaaataccaagtgcctcaattcgatcaatcaacgattaaaaatattgagtctgcaaaaggacttgatgtgtgggacagctggccgctgcaaaacgctgacggaacagtagctgaatacaacggctatcacgttgtgtttgctcttgcgggaagcccgaaagacgctgatgacacatcaatctacatgttttatcaaaaggtcggcgacaactcaatcgacagctggaaaaacgcgggccgtgtctttaaagacagcgataagttcgacgccaacgatccgatcctgaaagatcagacgcaagaatggtccggttctgcaacctttacatctgacggaaaaatccgtttattctacactgactattccggtaaacattacggcaaacaaagcctgacaacagcgcaggtaaatgtgtcaaaatctgatgacacactcaaaatcaacggagtggaagatcacaaaacgatttttgacggagacggaaaaacatatcagaacgttcagcagtttatcgatgaaggcaattatacatccggcgacaaccatacgctgagagaccctcactacgttgaagacaaaggccataaataccttgtattcgaagccaacacgggaacagaaaacggataccaaggcgaagaatctttatttaacaaagcgtactacggcggcggcacgaacttcttccgtaaagaaagccagaagcttcagcagagcgctaaaaaacgcgatgctgagttagcgaacggcgccctcggtatcatagagttaaataatgattacacattgaaaaaagtaatgaagccgctgatcacttcaaacacggtaactgatgaaatcgagcgcgcgaatgttttcaaaatgaacggcaaatggtacttgttcactgattcacgcggttcaaaaatgacgatcgatggtattaactcaaacgatatttacatgcttggttatgtatcaaactctttaaccggcccttacaagccgctgaacaaaacagggcttgtgctgcaaatgggtcttgatccaaacgatgtgacattcacttactctcacttcgcagtgccgcaagccaaaggcaacaatgtggttatcacaagctacatgacaaacagaggcttcttcgaggataaaaaggcaacatttgcgccaagcttcttaatgaacatcaaaggcaataaaacatccgttgtcaaaaacagcatcctggagcaaggacagctgacagtcaactaataacagcaaaaagaaaatgccgatacttcattggcattttcttttatttctcaacaagatggtgaattgactagtgggtagatccacaggacgggtgtggtcgccatgatcgcgtagtcgatagtggctccaagtagcgaagcgagcaggactgggcggcggccaaagcggtcggacagtgctccgagaacgggtgcgcatagaaattgcatcaacgcatatagcgctagcagcacgccatagtgactggcgatgctgtcggaatggacgatatcccgcaagaggcccggcagtaccggcataaccaagcctatgcctacagcatccagggtgacggtgccgaggatgacgatgagcgcattgttagatttcatacacggtgcctgactgcgttagcaatttaactgtgataaactaccgcattaaagcttatcgatgataagctgtcaaacatgagaattgatccggaacccttaatataacttcgtataatgtatgctatacgaagttattaggtccctcgactatagggtcaccgtcgacagcgacacacttgcatcggatgcagcccggttaacgtgccggcacggcctgggtaaccaggtattttgtccacataaccgtgcgcaaaatgttgtggataagcaggacacagcagcaatccacagcaggcatacaaccgcacaccgaggttactccgttctacaggttacgacgacatgtcaatacttgcccttgacaggcattgatggaatcgtagtctcacgctgatagtctgatcgacaatacaagtgggaccgtggtcccagaccgataatcagaccgacaacacgagtgggatcgtggtcccagactaataatcagaccgacgatacgagtgggaccgtggtcccagactaataatcagaccgacgatacgagtgggaccgtggttccagactaataatcagaccgacgatacgagtgggaccgtggtcccagactaataatcagaccgacgatacgagtgggaccatggtcccagactaataatcagaccgacgatacgagtgggaccgtggtcccagtctgattatcagaccgacgatacgagtgggaccgtggtcccagactaataatcagaccgacgatacgagtgggaccgtggtcccagactaataatcagaccgacgatacgagtgggaccgtggtcccagtctgattatcagaccgacgatacaagtggaacagtgggcccagagagaatattcaggccagttatgctttctggcctgtaacaaaggacattaagtaaagacagataaacgtagactaaaacgtggtcgcatcagggtgctggcttttcaagttccttaagaatggcctcaattttctctatacactcagttggaacacgagacctgtccaggttaagcaccattttatcgcccttatacaatactgtcgctccaggagcaaactgatgtcgtgagcttaaactagttcttgatgcagatgacgttttaagcacagaagttaaaagagtgataacttcttcagcttcaaatatcaccccagcttttttctgctcatgaaggttagatgcctgctgcttaagtaattcctctttatctgtaaaggctttttgaagtgcatcacctgaccgggcagatagttcaccggggtgagaaaaaagagcaacaactgatttaggcaatttggcggtgttgatacagcgggtaataatcttacgtgaaatattttccgcatcagccagcgcagaaatatttccagcaaattcattctgcaatcggcttgcataacgctgaccacgttcataagcacttgttgggcgataatcgttacccaatctggataatgcagccatctgctcatcatccagctcgccaaccagaacacgataatcactttcggtaagtgcagcagctttacgacggcgactcccatcggcaatttctatgacaccagatactcttcgaccgaacgccggtgtctgttgaccagtcagtagaaaagaagggatgagatcatccagtgcgtcctcagtaagcagctcctggtcacgttcattacctgaccatacccgagaggtcttctcaacactatcaccccggagcacttcaagagtaaacttcacatcccgaccacatacaggcaaagtaatggcattaccgcgagccattactcctacgcgcgcaattaacgaatccaccatcggggcagctggtgtcgataacgaagtatcttcaaccggttgagtattgagcgtatgttttggaataacaggcgcacgcttcattatctaatctcccagcgtggtttaatcagacgatcgaaaatttcattgcagacaggttcccaaatagaaagagcatttctccaggcaccagttgaagagcgttgatcaatggcctgttcaaaaacagttctcatccggatctgacctttaccaacttcatccgtttcacgtacaacattttttagaaccatgcttccccaggcatcccgaatttgctcctccatccacggggactgagagccattactattgctgtatttggtaagcaaaatacgtacatcaggctcgaaccctttaagatcaacgttcttgagcagatcacgaagcatatcgaaaaactgcagtgcggaggtgtagtcaaacaactcagcaggcgtgggaacaatcagcacatcagcagcacatacgacattaatcgtgccgatacccaggttaggcgcgctgtcaataactatgacatcatagtcatgagcaacagtttcaatggccagtcggagcatcaggtgtggatcggtgggcagtttaccttcatcaaatttgcccattaactcagtttcaatacggtgcagagccagacaggaaggaataatgtcaagccccggccagcaagtgggctttattgcataagtgacatcgtccttttccccaagatagaaaggcaggagagtgtcttctgcatgaatatgaagatctggtacccatccgtgatacattgaggctgttccctgggggtcgttaccttccacgagcaaaacacgtagccccttcagagccagatcctgagcaagatgaacagaaactgaggttttgtaaacgccacctttatgggcagcaaccccgatcaccggtggaaatacgtcttcagcacgtcgcaatcgcgtaccaaacacatcacgcatatgattaatttgttcaattgtataaccaacacgttgctcaacccgtcctcgaatttccatatccgggtgcggtagtcgccctgctttctcggcatctctgatagcctgagaagaaaccccaactaaatccgctgcttcacctattctccagcgccgggttattttcctcgcttccgggctgtcatcattaaactgtgcaatggcgatagccttcgtcatttcatgaccagcgtttatgcactggttaagtgtttccatgagtttcattctgaacatcctttaatcattgctttgcgtttttttattaaatcttgcaatttactgcaaagcaacaacaaaatcgcaaagtcatcaaaaaaccgcaaagttgtttaaaataagagcaacactacaaaaggagataagaagagcacatacctcagtcacttattatcactagcgctcgccgcagccgtgtaaccgagcatagcgagcgaactggcgaggaagcaaagaagaactgttctgtcagatagctcttacgctcagcgcaagaagaaatatccaccgtgggaaaaactccaggtagaggtacacacgcggatagccaattcagagtaataaactgtgataatcaaccctcatcaatgatgacgaactaacccccgatatcaggtcacatgacgaagggaaagagaaggaaatcaactgtgacaaactgccctcaaatttggcttccttaaaaattacagttcaaaaagtatgagaaaatccatgcaggctgaaggaaacagcaaaactgtgacaaattaccctcagtaggtcagaacaaatgtgacgaaccaccctcaaatctgtgacagataaccctcagactatcctgtcgtcatggaagtgatatcgcggaaggaaaatacgatatgagtcgtctggcggcctttctttttctcaatgtatgagaggcgcattggagttctgctgttgatctcattaacacagacctgcaggaagcggcggcggaagtcaggcatacgctggtaactttgaggcagctggtaacgctctatgatccagtcgattttcagagagacgatgcctgagccatccggcttacgatactgacacagggattcgtataaacgcatggcatacggattggtgatttcttttgtttcactaagccgaaactgcgtaaaccggttctgtaacccgataaagaagggaatgagatatgggttgatatgtacactgtaaagccctctggatggactgtgcgcacgtttgataaaccaaggaaaagattcatagcctttttcatcgccggcatcctcttcagggcgataaaaaaccacttccttccccgcgaaactcttcaatgcctgccgtatatccttactggcttccgcagaggtcaatccgaatatttcagcatatttagcaacatggatctcgcagataccgtcatgttcctgtagggtgccatcagattttctgatctggtcaacgaacagatacagcatacgtttttgatcccgggagagactatatgccgcctcagtgaggtcgtttgactggacgattcgcgggctatttttacgtttcttgtgattgataaccgctgtttccgccatgacagatccatgtgaagtgtgacaagtttttagattgtcacactaaataaaaaagagtcaataagcagggataactttgtgaaaaaacagcttcttctgagggcaatttgtcacagggttaagggcaatttgtcacagACAGGACTGTCATTTGAGGGTGATTTGTCACACTGAAAGGGCAATTTGTCACAACACCTTCTCTAGAACCAGCATGGATAAAGGCCTACAAGGCGCTCTAAAAAAGAAGATCTAAAAACTATAAAAAAAATAATTATAAAAATATCCCCGTGGATAAGTGGATAACCCCAAGGGAAGTTTTTTCAGGCATCGTGTGTAAGCAGAATATATAAGTGCTGTTCCCTGGTGCTTCCTCGCTCACTCGAGGGCTTCGCCCTGTCGCTCAACTGCGGCGAGCACTACTGGCTGTAAaaggacagaccacatcatggttctgtgttcattaggttgttctgtccattgctgacataatccgctccacttcaacgtaacaccgcacgaagatttctattgttcctgaaggcatattcaaatcgttttcgttaccgcttgcaggcatcatgacagaacactacttcctataaacgctacacaggctcctgagattaataatgcggatctctacgataatgggagattttcccgactgtttcgttcgcttctcagtggataacagccagcttctctgtttaacagacaaaaacagcatatccactcagttccacatttccatataaaggccaaggcatttattctcaggataattgtttcagcatcgcaaccgcatcagactccggcatcgcaaactgcacccggtgccgggcagccacatccagcgcaaaaaccttcgtgtagacttccgttgaactgatggacttatgtcccatcaggctttgcagaactttcagcggtataccggcatacagcatgtgcatcgcataggaatggcggaacgtatgtggtgtgaccggaacagagaacgtcacaccgtcagcagcagcggcggcaaccgcctccccaatccaggtcctgaccgttctgtccgtcacttcccagatccgcgctttctctgtccttcctgtgcgacggttacgccgctccatgagcttatcgcgaataaatacctgtgacggaagatcacttcgcagaataaataaatcctggtgtccctgttgataccgggaagccctgggccaacttttggcgaaaatgagacgttgatcggcacgtaagaggttccaactttcaccataatgaaataagatcactaccgggcgtattttttgagttatcgagattttcaggagctaaggaagctaaaatggagaaaaaaatcactggatataccaccgttgatatatcccaatggcatcgtaaagaacattttgaggcatttcagtcagttgctcaatgtacctataaccagaccgttcagctggatattacggcctttttaaagaccgtaaagaaaaataagcacaagttttatccggcctttattcacattcttgcccgcctgatgaatgctcatccggagttccgtatggcaatgaaagacggtgagctggtgatatgggatagtgttcacccttgttacaccgttttccatgagcaaactgaaacgttttcatcgctctggagtgaataccacgacgatttccggcagtttctacacatatattcgcaagatgtggcgtgttacggtgaaaacctggcctatttccctaaagggtttattgagaatatgtttttcgtctcagccaatccctgggtgagtttcaccagttttgatttaaacgtggccaatatggacaacttcttcgcccccgttttcaccatgggcaaatattatacgcaaggcgacaaggtgctgatgccgctggcgattcaggttcatcatgccgtttgtgatggcttccatgtcggcagaatgcttaatgaattacaacagtactgcgatgagtggcagggcggggcgtaatttttttaaggcagttattggtgcccttaaacgcctggttgctacgcctgaataagtgataataagcggatgaatggcagaaattcgatgataagctgtcaaacatgagaattggtcgacggcgcgccaaagcttgcatgcctgcagccgcgtaacctggcaaaatcggttacggttgagtaataaatggatgccctgcgtaagcggggcacatttcattacctctttctccgcacccgacatagataataacttcgtatagtatacattatacgaagttatctagtagacttaattaaggatcgatccggcgcgccaatagtcatgccccgcgcccaccggaaggagctgactgggttgaaggctctcaagggcatcggtcgagcttgacattgtaggactatattgctctaataaatttgcggccgctaatacgactcactatagggagag |
| pRC1787  (Bp6++ EGFP Flag) | gatccgcggaattgGTTAATAACAGGGGACGTGGTAATCCGTCCCCTTTTTATTTCTGACTGAGTTAATAACAGGCCTGCTTCGGCAGGCCTTTTTATTTCTGACTGAGTTCTTCTCAGGCCTGCTGGTAATCGCAGGCCTTTTTATTTTCTAGTCAATTCCGACGTCTAAGAAACCATTATTATCATGACATTAACCTATAAAAATAGGCGTATCACGAGGCCCTTTCGTCTTCACCTCGAGTCCCTATCAGTGATAGAGATTGACATCCCTATCAGTGATAGAGATACTGAGCACATCAGCAGGACGCACTGACCACTTTAAGAAGGAGATATACATATGGTGAGCAAGGGCGAGGAGCTGTTCACCGGGGTGGTGCCCATCCTGGTCGAGCTGGACGGCGACGTAAACGGCCACAAGTTCAGCGTGCGCGGCGAGGGCGAGGGCGATGCCACCAACGGCAAGCTGACCCTGAAGTTCATCTGCACCACCGGCAAGCTGCCCGTGCCCTGGCCCACCCTCGTGACCACCCTGACCTACGGCGTGCAGTGCTTCAGCCGCTACCCCGACCACATGAAGCAGCACGACTTCTTCAAGTCCGCCATGCCCGAAGGCTACGTCCAGGAGCGCACCATCTCCTTCAAGGACGACGGCACCTACAAGACCCGCGCCGAGGTGAAGTTCGAGGGCGACACCCTGGTGAACCGCATCGAGCTGAAGGGCATCGACTTCAAGGAGGACGGCAACATCCTGGGGCACAAGCTGGAGTACAACTACAACAGCCACAACGTCTATATCACCGCTGACAAGCAGAAGAACGGCATCAAGGCCAACTTCAAGATCCGCCACAACATCGAGGACGGCAGCGTGCAGCTCGCCGACCACTACCAGCAGAACACCCCCATCGGCGACGGCCCCGTGCTGCTGCCCGACAACCACTACCTGAGCACCCAGTCCGCCCTGAGCAAAGACCCCAACGAGAAGCGCGATCACATGGTCCTGCTGGAGTTCGTGACCGCCGCCGGGATCACTCTCGGCATGGACGAGCTGTACAAGTAAGGATCCgactacaaggacgacgatgacaagGAAgattataaagatgatgacgataaaAGAgactacaaggacgacgatgacaagTAAAAGCTTGTTCTTCTCAGGCCTGCTGGTAATCGCAGGCCTTTTTATTTGAATTCcgcggatccttctatagtgtcacctaaatgtcgacggccaggcggccgccaggcctacccactagtcaattcgggaggatcgaaacggcagatcgcaaaaaacagtacatacagaaggagacatgaacatgaacatcaaaaaaattgtaaaacaagccacagttctgacttttacgactgcacttctggcaggaggagcgactcaagccttcgcgaaagaaaataaccaaaaagcatacaaagaaacgtacggcgtctctcatattacacgccatgatatgctgcagatccctaaacagcagcaaaacgaaaaataccaagtgcctcaattcgatcaatcaacgattaaaaatattgagtctgcaaaaggacttgatgtgtgggacagctggccgctgcaaaacgctgacggaacagtagctgaatacaacggctatcacgttgtgtttgctcttgcgggaagcccgaaagacgctgatgacacatcaatctacatgttttatcaaaaggtcggcgacaactcaatcgacagctggaaaaacgcgggccgtgtctttaaagacagcgataagttcgacgccaacgatccgatcctgaaagatcagacgcaagaatggtccggttctgcaacctttacatctgacggaaaaatccgtttattctacactgactattccggtaaacattacggcaaacaaagcctgacaacagcgcaggtaaatgtgtcaaaatctgatgacacactcaaaatcaacggagtggaagatcacaaaacgatttttgacggagacggaaaaacatatcagaacgttcagcagtttatcgatgaaggcaattatacatccggcgacaaccatacgctgagagaccctcactacgttgaagacaaaggccataaataccttgtattcgaagccaacacgggaacagaaaacggataccaaggcgaagaatctttatttaacaaagcgtactacggcggcggcacgaacttcttccgtaaagaaagccagaagcttcagcagagcgctaaaaaacgcgatgctgagttagcgaacggcgccctcggtatcatagagttaaataatgattacacattgaaaaaagtaatgaagccgctgatcacttcaaacacggtaactgatgaaatcgagcgcgcgaatgttttcaaaatgaacggcaaatggtacttgttcactgattcacgcggttcaaaaatgacgatcgatggtattaactcaaacgatatttacatgcttggttatgtatcaaactctttaaccggcccttacaagccgctgaacaaaacagggcttgtgctgcaaatgggtcttgatccaaacgatgtgacattcacttactctcacttcgcagtgccgcaagccaaaggcaacaatgtggttatcacaagctacatgacaaacagaggcttcttcgaggataaaaaggcaacatttgcgccaagcttcttaatgaacatcaaaggcaataaaacatccgttgtcaaaaacagcatcctggagcaaggacagctgacagtcaactaataacagcaaaaagaaaatgccgatacttcattggcattttcttttatttctcaacaagatggtgaattgactagtgggtagatccacaggacgggtgtggtcgccatgatcgcgtagtcgatagtggctccaagtagcgaagcgagcaggactgggcggcggccaaagcggtcggacagtgctccgagaacgggtgcgcatagaaattgcatcaacgcatatagcgctagcagcacgccatagtgactggcgatgctgtcggaatggacgatatcccgcaagaggcccggcagtaccggcataaccaagcctatgcctacagcatccagggtgacggtgccgaggatgacgatgagcgcattgttagatttcatacacggtgcctgactgcgttagcaatttaactgtgataaactaccgcattaaagcttatcgatgataagctgtcaaacatgagaattgatccggaacccttaatataacttcgtataatgtatgctatacgaagttattaggtccctcgactatagggtcaccgtcgacagcgacacacttgcatcggatgcagcccggttaacgtgccggcacggcctgggtaaccaggtattttgtccacataaccgtgcgcaaaatgttgtggataagcaggacacagcagcaatccacagcaggcatacaaccgcacaccgaggttactccgttctacaggttacgacgacatgtcaatacttgcccttgacaggcattgatggaatcgtagtctcacgctgatagtctgatcgacaatacaagtgggaccgtggtcccagaccgataatcagaccgacaacacgagtgggatcgtggtcccagactaataatcagaccgacgatacgagtgggaccgtggtcccagactaataatcagaccgacgatacgagtgggaccgtggttccagactaataatcagaccgacgatacgagtgggaccgtggtcccagactaataatcagaccgacgatacgagtgggaccatggtcccagactaataatcagaccgacgatacgagtgggaccgtggtcccagtctgattatcagaccgacgatacgagtgggaccgtggtcccagactaataatcagaccgacgatacgagtgggaccgtggtcccagactaataatcagaccgacgatacgagtgggaccgtggtcccagtctgattatcagaccgacgatacaagtggaacagtgggcccagagagaatattcaggccagttatgctttctggcctgtaacaaaggacattaagtaaagacagataaacgtagactaaaacgtggtcgcatcagggtgctggcttttcaagttccttaagaatggcctcaattttctctatacactcagttggaacacgagacctgtccaggttaagcaccattttatcgcccttatacaatactgtcgctccaggagcaaactgatgtcgtgagcttaaactagttcttgatgcagatgacgttttaagcacagaagttaaaagagtgataacttcttcagcttcaaatatcaccccagcttttttctgctcatgaaggttagatgcctgctgcttaagtaattcctctttatctgtaaaggctttttgaagtgcatcacctgaccgggcagatagttcaccggggtgagaaaaaagagcaacaactgatttaggcaatttggcggtgttgatacagcgggtaataatcttacgtgaaatattttccgcatcagccagcgcagaaatatttccagcaaattcattctgcaatcggcttgcataacgctgaccacgttcataagcacttgttgggcgataatcgttacccaatctggataatgcagccatctgctcatcatccagctcgccaaccagaacacgataatcactttcggtaagtgcagcagctttacgacggcgactcccatcggcaatttctatgacaccagatactcttcgaccgaacgccggtgtctgttgaccagtcagtagaaaagaagggatgagatcatccagtgcgtcctcagtaagcagctcctggtcacgttcattacctgaccatacccgagaggtcttctcaacactatcaccccggagcacttcaagagtaaacttcacatcccgaccacatacaggcaaagtaatggcattaccgcgagccattactcctacgcgcgcaattaacgaatccaccatcggggcagctggtgtcgataacgaagtatcttcaaccggttgagtattgagcgtatgttttggaataacaggcgcacgcttcattatctaatctcccagcgtggtttaatcagacgatcgaaaatttcattgcagacaggttcccaaatagaaagagcatttctccaggcaccagttgaagagcgttgatcaatggcctgttcaaaaacagttctcatccggatctgacctttaccaacttcatccgtttcacgtacaacattttttagaaccatgcttccccaggcatcccgaatttgctcctccatccacggggactgagagccattactattgctgtatttggtaagcaaaatacgtacatcaggctcgaaccctttaagatcaacgttcttgagcagatcacgaagcatatcgaaaaactgcagtgcggaggtgtagtcaaacaactcagcaggcgtgggaacaatcagcacatcagcagcacatacgacattaatcgtgccgatacccaggttaggcgcgctgtcaataactatgacatcatagtcatgagcaacagtttcaatggccagtcggagcatcaggtgtggatcggtgggcagtttaccttcatcaaatttgcccattaactcagtttcaatacggtgcagagccagacaggaaggaataatgtcaagccccggccagcaagtgggctttattgcataagtgacatcgtccttttccccaagatagaaaggcaggagagtgtcttctgcatgaatatgaagatctggtacccatccgtgatacattgaggctgttccctgggggtcgttaccttccacgagcaaaacacgtagccccttcagagccagatcctgagcaagatgaacagaaactgaggttttgtaaacgccacctttatgggcagcaaccccgatcaccggtggaaatacgtcttcagcacgtcgcaatcgcgtaccaaacacatcacgcatatgattaatttgttcaattgtataaccaacacgttgctcaacccgtcctcgaatttccatatccgggtgcggtagtcgccctgctttctcggcatctctgatagcctgagaagaaaccccaactaaatccgctgcttcacctattctccagcgccgggttattttcctcgcttccgggctgtcatcattaaactgtgcaatggcgatagccttcgtcatttcatgaccagcgtttatgcactggttaagtgtttccatgagtttcattctgaacatcctttaatcattgctttgcgtttttttattaaatcttgcaatttactgcaaagcaacaacaaaatcgcaaagtcatcaaaaaaccgcaaagttgtttaaaataagagcaacactacaaaaggagataagaagagcacatacctcagtcacttattatcactagcgctcgccgcagccgtgtaaccgagcatagcgagcgaactggcgaggaagcaaagaagaactgttctgtcagatagctcttacgctcagcgcaagaagaaatatccaccgtgggaaaaactccaggtagaggtacacacgcggatagccaattcagagtaataaactgtgataatcaaccctcatcaatgatgacgaactaacccccgatatcaggtcacatgacgaagggaaagagaaggaaatcaactgtgacaaactgccctcaaatttggcttccttaaaaattacagttcaaaaagtatgagaaaatccatgcaggctgaaggaaacagcaaaactgtgacaaattaccctcagtaggtcagaacaaatgtgacgaaccaccctcaaatctgtgacagataaccctcagactatcctgtcgtcatggaagtgatatcgcggaaggaaaatacgatatgagtcgtctggcggcctttctttttctcaatgtatgagaggcgcattggagttctgctgttgatctcattaacacagacctgcaggaagcggcggcggaagtcaggcatacgctggtaactttgaggcagctggtaacgctctatgatccagtcgattttcagagagacgatgcctgagccatccggcttacgatactgacacagggattcgtataaacgcatggcatacggattggtgatttcttttgtttcactaagccgaaactgcgtaaaccggttctgtaacccgataaagaagggaatgagatatgggttgatatgtacactgtaaagccctctggatggactgtgcgcacgtttgataaaccaaggaaaagattcatagcctttttcatcgccggcatcctcttcagggcgataaaaaaccacttccttccccgcgaaactcttcaatgcctgccgtatatccttactggcttccgcagaggtcaatccgaatatttcagcatatttagcaacatggatctcgcagataccgtcatgttcctgtagggtgccatcagattttctgatctggtcaacgaacagatacagcatacgtttttgatcccgggagagactatatgccgcctcagtgaggtcgtttgactggacgattcgcgggctatttttacgtttcttgtgattgataaccgctgtttccgccatgacagatccatgtgaagtgtgacaagtttttagattgtcacactaaataaaaaagagtcaataagcagggataactttgtgaaaaaacagcttcttctgagggcaatttgtcacagggttaagggcaatttgtcacagACAGGACTGTCATTTGAGGGTGATTTGTCACACTGAAAGGGCAATTTGTCACAACACCTTCTCTAGAACCAGCATGGATAAAGGCCTACAAGGCGCTCTAAAAAAGAAGATCTAAAAACTATAAAAAAAATAATTATAAAAATATCCCCGTGGATAAGTGGATAACCCCAAGGGAAGTTTTTTCAGGCATCGTGTGTAAGCAGAATATATAAGTGCTGTTCCCTGGTGCTTCCTCGCTCACTCGAGGGCTTCGCCCTGTCGCTCAACTGCGGCGAGCACTACTGGCTGTAAaaggacagaccacatcatggttctgtgttcattaggttgttctgtccattgctgacataatccgctccacttcaacgtaacaccgcacgaagatttctattgttcctgaaggcatattcaaatcgttttcgttaccgcttgcaggcatcatgacagaacactacttcctataaacgctacacaggctcctgagattaataatgcggatctctacgataatgggagattttcccgactgtttcgttcgcttctcagtggataacagccagcttctctgtttaacagacaaaaacagcatatccactcagttccacatttccatataaaggccaaggcatttattctcaggataattgtttcagcatcgcaaccgcatcagactccggcatcgcaaactgcacccggtgccgggcagccacatccagcgcaaaaaccttcgtgtagacttccgttgaactgatggacttatgtcccatcaggctttgcagaactttcagcggtataccggcatacagcatgtgcatcgcataggaatggcggaacgtatgtggtgtgaccggaacagagaacgtcacaccgtcagcagcagcggcggcaaccgcctccccaatccaggtcctgaccgttctgtccgtcacttcccagatccgcgctttctctgtccttcctgtgcgacggttacgccgctccatgagcttatcgcgaataaatacctgtgacggaagatcacttcgcagaataaataaatcctggtgtccctgttgataccgggaagccctgggccaacttttggcgaaaatgagacgttgatcggcacgtaagaggttccaactttcaccataatgaaataagatcactaccgggcgtattttttgagttatcgagattttcaggagctaaggaagctaaaatggagaaaaaaatcactggatataccaccgttgatatatcccaatggcatcgtaaagaacattttgaggcatttcagtcagttgctcaatgtacctataaccagaccgttcagctggatattacggcctttttaaagaccgtaaagaaaaataagcacaagttttatccggcctttattcacattcttgcccgcctgatgaatgctcatccggagttccgtatggcaatgaaagacggtgagctggtgatatgggatagtgttcacccttgttacaccgttttccatgagcaaactgaaacgttttcatcgctctggagtgaataccacgacgatttccggcagtttctacacatatattcgcaagatgtggcgtgttacggtgaaaacctggcctatttccctaaagggtttattgagaatatgtttttcgtctcagccaatccctgggtgagtttcaccagttttgatttaaacgtggccaatatggacaacttcttcgcccccgttttcaccatgggcaaatattatacgcaaggcgacaaggtgctgatgccgctggcgattcaggttcatcatgccgtttgtgatggcttccatgtcggcagaatgcttaatgaattacaacagtactgcgatgagtggcagggcggggcgtaatttttttaaggcagttattggtgcccttaaacgcctggttgctacgcctgaataagtgataataagcggatgaatggcagaaattcgatgataagctgtcaaacatgagaattggtcgacggcgcgccaaagcttgcatgcctgcagccgcgtaacctggcaaaatcggttacggttgagtaataaatggatgccctgcgtaagcggggcacatttcattacctctttctccgcacccgacatagataataacttcgtatagtatacattatacgaagttatctagtagacttaattaaggatcgatccggcgcgccaatagtcatgccccgcgcccaccggaaggagctgactgggttgaaggctctcaagggcatcggtcgagcttgacattgtaggactatattgctctaataaatttgcggccgctaatacgactcactatagggagag |
| pRC1794  (Bp-EE- EGFP Flag) | gatccgcggaattgTTAATAACAGGGGACGTGGTAATCCGTCCCCTTTTTATTTCTGACTGAGTTAATAACAGGCCTGCTTCGGCAGGCCTTTTTATTTCTGACTGAGTTCTTCTCAGGCCTGCTGGTAATCGCAGGCCTTTTTATTTTCTAGgCTGACTGACTGACTGACTGACTGACTGACTGACTGACTGACTGACTGACTGACTGACTGACTGACTGACTGACTGACTGACTGACTGACTGACTGACTGACTGACTGACTGACTGACTGACTGACTGACTGACTGACTGACTGACTGACTGACTGACTtctcctCTGACCATATGGTGAGCAAGGGCGAGGAGCTGTTCACCGGGGTGGTGCCCATCCTGGTCGAGCTGGACGGCGACGTAAACGGCCACAAGTTCAGCGTGCGCGGCGAGGGCGAGGGCGATGCCACCAACGGCAAGCTGACCCTGAAGTTCATCTGCACCACCGGCAAGCTGCCCGTGCCCTGGCCCACCCTCGTGACCACCCTGACCTACGGCGTGCAGTGCTTCAGCCGCTACCCCGACCACATGAAGCAGCACGACTTCTTCAAGTCCGCCATGCCCGAAGGCTACGTCCAGGAGCGCACCATCTCCTTCAAGGACGACGGCACCTACAAGACCCGCGCCGAGGTGAAGTTCGAGGGCGACACCCTGGTGAACCGCATCGAGCTGAAGGGCATCGACTTCAAGGAGGACGGCAACATCCTGGGGCACAAGCTGGAGTACAACTACAACAGCCACAACGTCTATATCACCGCTGACAAGCAGAAGAACGGCATCAAGGCCAACTTCAAGATCCGCCACAACATCGAGGACGGCAGCGTGCAGCTCGCCGACCACTACCAGCAGAACACCCCCATCGGCGACGGCCCCGTGCTGCTGCCCGACAACCACTACCTGAGCACCCAGTCCGCCCTGAGCAAAGACCCCAACGAGAAGCGCGATCACATGGTCCTGCTGGAGTTCGTGACCGCCGCCGGGATCACTCTCGGCATGGACGAGCTGTACAAGTAAGGATCCgactacaaggacgacgatgacaagGAAgattataaagatgatgacgataaaAGAgactacaaggacgacgatgacaagTAAAAGCTTGTTCTTCTCAGGCCTGCTGGTAATCGCAGGCCTTTTTATTTGAATTCcgcggatccttctatagtgtcacctaaatgtcgacggccaggcggccgccaggcctacccactagtcaattcgggaggatcgaaacggcagatcgcaaaaaacagtacatacagaaggagacatgaacatgaacatcaaaaaaattgtaaaacaagccacagttctgacttttacgactgcacttctggcaggaggagcgactcaagccttcgcgaaagaaaataaccaaaaagcatacaaagaaacgtacggcgtctctcatattacacgccatgatatgctgcagatccctaaacagcagcaaaacgaaaaataccaagtgcctcaattcgatcaatcaacgattaaaaatattgagtctgcaaaaggacttgatgtgtgggacagctggccgctgcaaaacgctgacggaacagtagctgaatacaacggctatcacgttgtgtttgctcttgcgggaagcccgaaagacgctgatgacacatcaatctacatgttttatcaaaaggtcggcgacaactcaatcgacagctggaaaaacgcgggccgtgtctttaaagacagcgataagttcgacgccaacgatccgatcctgaaagatcagacgcaagaatggtccggttctgcaacctttacatctgacggaaaaatccgtttattctacactgactattccggtaaacattacggcaaacaaagcctgacaacagcgcaggtaaatgtgtcaaaatctgatgacacactcaaaatcaacggagtggaagatcacaaaacgatttttgacggagacggaaaaacatatcagaacgttcagcagtttatcgatgaaggcaattatacatccggcgacaaccatacgctgagagaccctcactacgttgaagacaaaggccataaataccttgtattcgaagccaacacgggaacagaaaacggataccaaggcgaagaatctttatttaacaaagcgtactacggcggcggcacgaacttcttccgtaaagaaagccagaagcttcagcagagcgctaaaaaacgcgatgctgagttagcgaacggcgccctcggtatcatagagttaaataatgattacacattgaaaaaagtaatgaagccgctgatcacttcaaacacggtaactgatgaaatcgagcgcgcgaatgttttcaaaatgaacggcaaatggtacttgttcactgattcacgcggttcaaaaatgacgatcgatggtattaactcaaacgatatttacatgcttggttatgtatcaaactctttaaccggcccttacaagccgctgaacaaaacagggcttgtgctgcaaatgggtcttgatccaaacgatgtgacattcacttactctcacttcgcagtgccgcaagccaaaggcaacaatgtggttatcacaagctacatgacaaacagaggcttcttcgaggataaaaaggcaacatttgcgccaagcttcttaatgaacatcaaaggcaataaaacatccgttgtcaaaaacagcatcctggagcaaggacagctgacagtcaactaataacagcaaaaagaaaatgccgatacttcattggcattttcttttatttctcaacaagatggtgaattgactagtgggtagatccacaggacgggtgtggtcgccatgatcgcgtagtcgatagtggctccaagtagcgaagcgagcaggactgggcggcggccaaagcggtcggacagtgctccgagaacgggtgcgcatagaaattgcatcaacgcatatagcgctagcagcacgccatagtgactggcgatgctgtcggaatggacgatatcccgcaagaggcccggcagtaccggcataaccaagcctatgcctacagcatccagggtgacggtgccgaggatgacgatgagcgcattgttagatttcatacacggtgcctgactgcgttagcaatttaactgtgataaactaccgcattaaagcttatcgatgataagctgtcaaacatgagaattgatccggaacccttaatataacttcgtataatgtatgctatacgaagttattaggtccctcgactatagggtcaccgtcgacagcgacacacttgcatcggatgcagcccggttaacgtgccggcacggcctgggtaaccaggtattttgtccacataaccgtgcgcaaaatgttgtggataagcaggacacagcagcaatccacagcaggcatacaaccgcacaccgaggttactccgttctacaggttacgacgacatgtcaatacttgcccttgacaggcattgatggaatcgtagtctcacgctgatagtctgatcgacaatacaagtgggaccgtggtcccagaccgataatcagaccgacaacacgagtgggatcgtggtcccagactaataatcagaccgacgatacgagtgggaccgtggtcccagactaataatcagaccgacgatacgagtgggaccgtggttccagactaataatcagaccgacgatacgagtgggaccgtggtcccagactaataatcagaccgacgatacgagtgggaccatggtcccagactaataatcagaccgacgatacgagtgggaccgtggtcccagtctgattatcagaccgacgatacgagtgggaccgtggtcccagactaataatcagaccgacgatacgagtgggaccgtggtcccagactaataatcagaccgacgatacgagtgggaccgtggtcccagtctgattatcagaccgacgatacaagtggaacagtgggcccagagagaatattcaggccagttatgctttctggcctgtaacaaaggacattaagtaaagacagataaacgtagactaaaacgtggtcgcatcagggtgctggcttttcaagttccttaagaatggcctcaattttctctatacactcagttggaacacgagacctgtccaggttaagcaccattttatcgcccttatacaatactgtcgctccaggagcaaactgatgtcgtgagcttaaactagttcttgatgcagatgacgttttaagcacagaagttaaaagagtgataacttcttcagcttcaaatatcaccccagcttttttctgctcatgaaggttagatgcctgctgcttaagtaattcctctttatctgtaaaggctttttgaagtgcatcacctgaccgggcagatagttcaccggggtgagaaaaaagagcaacaactgatttaggcaatttggcggtgttgatacagcgggtaataatcttacgtgaaatattttccgcatcagccagcgcagaaatatttccagcaaattcattctgcaatcggcttgcataacgctgaccacgttcataagcacttgttgggcgataatcgttacccaatctggataatgcagccatctgctcatcatccagctcgccaaccagaacacgataatcactttcggtaagtgcagcagctttacgacggcgactcccatcggcaatttctatgacaccagatactcttcgaccgaacgccggtgtctgttgaccagtcagtagaaaagaagggatgagatcatccagtgcgtcctcagtaagcagctcctggtcacgttcattacctgaccatacccgagaggtcttctcaacactatcaccccggagcacttcaagagtaaacttcacatcccgaccacatacaggcaaagtaatggcattaccgcgagccattactcctacgcgcgcaattaacgaatccaccatcggggcagctggtgtcgataacgaagtatcttcaaccggttgagtattgagcgtatgttttggaataacaggcgcacgcttcattatctaatctcccagcgtggtttaatcagacgatcgaaaatttcattgcagacaggttcccaaatagaaagagcatttctccaggcaccagttgaagagcgttgatcaatggcctgttcaaaaacagttctcatccggatctgacctttaccaacttcatccgtttcacgtacaacattttttagaaccatgcttccccaggcatcccgaatttgctcctccatccacggggactgagagccattactattgctgtatttggtaagcaaaatacgtacatcaggctcgaaccctttaagatcaacgttcttgagcagatcacgaagcatatcgaaaaactgcagtgcggaggtgtagtcaaacaactcagcaggcgtgggaacaatcagcacatcagcagcacatacgacattaatcgtgccgatacccaggttaggcgcgctgtcaataactatgacatcatagtcatgagcaacagtttcaatggccagtcggagcatcaggtgtggatcggtgggcagtttaccttcatcaaatttgcccattaactcagtttcaatacggtgcagagccagacaggaaggaataatgtcaagccccggccagcaagtgggctttattgcataagtgacatcgtccttttccccaagatagaaaggcaggagagtgtcttctgcatgaatatgaagatctggtacccatccgtgatacattgaggctgttccctgggggtcgttaccttccacgagcaaaacacgtagccccttcagagccagatcctgagcaagatgaacagaaactgaggttttgtaaacgccacctttatgggcagcaaccccgatcaccggtggaaatacgtcttcagcacgtcgcaatcgcgtaccaaacacatcacgcatatgattaatttgttcaattgtataaccaacacgttgctcaacccgtcctcgaatttccatatccgggtgcggtagtcgccctgctttctcggcatctctgatagcctgagaagaaaccccaactaaatccgctgcttcacctattctccagcgccgggttattttcctcgcttccgggctgtcatcattaaactgtgcaatggcgatagccttcgtcatttcatgaccagcgtttatgcactggttaagtgtttccatgagtttcattctgaacatcctttaatcattgctttgcgtttttttattaaatcttgcaatttactgcaaagcaacaacaaaatcgcaaagtcatcaaaaaaccgcaaagttgtttaaaataagagcaacactacaaaaggagataagaagagcacatacctcagtcacttattatcactagcgctcgccgcagccgtgtaaccgagcatagcgagcgaactggcgaggaagcaaagaagaactgttctgtcagatagctcttacgctcagcgcaagaagaaatatccaccgtgggaaaaactccaggtagaggtacacacgcggatagccaattcagagtaataaactgtgataatcaaccctcatcaatgatgacgaactaacccccgatatcaggtcacatgacgaagggaaagagaaggaaatcaactgtgacaaactgccctcaaatttggcttccttaaaaattacagttcaaaaagtatgagaaaatccatgcaggctgaaggaaacagcaaaactgtgacaaattaccctcagtaggtcagaacaaatgtgacgaaccaccctcaaatctgtgacagataaccctcagactatcctgtcgtcatggaagtgatatcgcggaaggaaaatacgatatgagtcgtctggcggcctttctttttctcaatgtatgagaggcgcattggagttctgctgttgatctcattaacacagacctgcaggaagcggcggcggaagtcaggcatacgctggtaactttgaggcagctggtaacgctctatgatccagtcgattttcagagagacgatgcctgagccatccggcttacgatactgacacagggattcgtataaacgcatggcatacggattggtgatttcttttgtttcactaagccgaaactgcgtaaaccggttctgtaacccgataaagaagggaatgagatatgggttgatatgtacactgtaaagccctctggatggactgtgcgcacgtttgataaaccaaggaaaagattcatagcctttttcatcgccggcatcctcttcagggcgataaaaaaccacttccttccccgcgaaactcttcaatgcctgccgtatatccttactggcttccgcagaggtcaatccgaatatttcagcatatttagcaacatggatctcgcagataccgtcatgttcctgtagggtgccatcagattttctgatctggtcaacgaacagatacagcatacgtttttgatcccgggagagactatatgccgcctcagtgaggtcgtttgactggacgattcgcgggctatttttacgtttcttgtgattgataaccgctgtttccgccatgacagatccatgtgaagtgtgacaagtttttagattgtcacactaaataaaaaagagtcaataagcagggataactttgtgaaaaaacagcttcttctgagggcaatttgtcacagggttaagggcaatttgtcacagACAGGACTGTCATTTGAGGGTGATTTGTCACACTGAAAGGGCAATTTGTCACAACACCTTCTCTAGAACCAGCATGGATAAAGGCCTACAAGGCGCTCTAAAAAAGAAGATCTAAAAACTATAAAAAAAATAATTATAAAAATATCCCCGTGGATAAGTGGATAACCCCAAGGGAAGTTTTTTCAGGCATCGTGTGTAAGCAGAATATATAAGTGCTGTTCCCTGGTGCTTCCTCGCTCACTCGAGGGCTTCGCCCTGTCGCTCAACTGCGGCGAGCACTACTGGCTGTAAaaggacagaccacatcatggttctgtgttcattaggttgttctgtccattgctgacataatccgctccacttcaacgtaacaccgcacgaagatttctattgttcctgaaggcatattcaaatcgttttcgttaccgcttgcaggcatcatgacagaacactacttcctataaacgctacacaggctcctgagattaataatgcggatctctacgataatgggagattttcccgactgtttcgttcgcttctcagtggataacagccagcttctctgtttaacagacaaaaacagcatatccactcagttccacatttccatataaaggccaaggcatttattctcaggataattgtttcagcatcgcaaccgcatcagactccggcatcgcaaactgcacccggtgccgggcagccacatccagcgcaaaaaccttcgtgtagacttccgttgaactgatggacttatgtcccatcaggctttgcagaactttcagcggtataccggcatacagcatgtgcatcgcataggaatggcggaacgtatgtggtgtgaccggaacagagaacgtcacaccgtcagcagcagcggcggcaaccgcctccccaatccaggtcctgaccgttctgtccgtcacttcccagatccgcgctttctctgtccttcctgtgcgacggttacgccgctccatgagcttatcgcgaataaatacctgtgacggaagatcacttcgcagaataaataaatcctggtgtccctgttgataccgggaagccctgggccaacttttggcgaaaatgagacgttgatcggcacgtaagaggttccaactttcaccataatgaaataagatcactaccgggcgtattttttgagttatcgagattttcaggagctaaggaagctaaaatggagaaaaaaatcactggatataccaccgttgatatatcccaatggcatcgtaaagaacattttgaggcatttcagtcagttgctcaatgtacctataaccagaccgttcagctggatattacggcctttttaaagaccgtaaagaaaaataagcacaagttttatccggcctttattcacattcttgcccgcctgatgaatgctcatccggagttccgtatggcaatgaaagacggtgagctggtgatatgggatagtgttcacccttgttacaccgttttccatgagcaaactgaaacgttttcatcgctctggagtgaataccacgacgatttccggcagtttctacacatatattcgcaagatgtggcgtgttacggtgaaaacctggcctatttccctaaagggtttattgagaatatgtttttcgtctcagccaatccctgggtgagtttcaccagttttgatttaaacgtggccaatatggacaacttcttcgcccccgttttcaccatgggcaaatattatacgcaaggcgacaaggtgctgatgccgctggcgattcaggttcatcatgccgtttgtgatggcttccatgtcggcagaatgcttaatgaattacaacagtactgcgatgagtggcagggcggggcgtaatttttttaaggcagttattggtgcccttaaacgcctggttgctacgcctgaataagtgataataagcggatgaatggcagaaattcgatgataagctgtcaaacatgagaattggtcgacggcgcgccaaagcttgcatgcctgcagccgcgtaacctggcaaaatcggttacggttgagtaataaatggatgccctgcgtaagcggggcacatttcattacctctttctccgcacccgacatagataataacttcgtatagtatacattatacgaagttatctagtagacttaattaaggatcgatccggcgcgccaatagtcatgccccgcgcccaccggaaggagctgactgggttgaaggctctcaagggcatcggtcgagcttgacattgtaggactatattgctctaataaatttgcggccgctaatacgactcactatagggagag |
| pRC1795  (Bp2- EGFP Flag) | gatccgcggaattgTGGTTAATAACAGGGGACGTGGTAATCCGTCCCCTTTTTATTTCTGACTGAGTTAATAACAGGCCTGCTTCGGCAGGCCTTTTTATTTCTGACTGAGTTCTTCTCAGGCCTGCTGGTAATCGCAGGCCTTTTTATTTTCTAGaCAATTCCGACGTCTAAGGAAACCATTATCATGACATCAACCTATAAAAATAGGCGTATCACGAGGCCCTCTCGTCTCCACCTCAAGCTCCCTATCTAGTGATAGCGATTGACATCCCTATCAGTGACGGAGATATTGAGCACATCAGCAGGACGCACTGACCACTTTAAGtctcctATATACATATGGTGAGCAAGGGCGAGGAGCTGTTCACCGGGGTGGTGCCCATCCTGGTCGAGCTGGACGGCGACGTAAACGGCCACAAGTTCAGCGTGCGCGGCGAGGGCGAGGGCGATGCCACCAACGGCAAGCTGACCCTGAAGTTCATCTGCACCACCGGCAAGCTGCCCGTGCCCTGGCCCACCCTCGTGACCACCCTGACCTACGGCGTGCAGTGCTTCAGCCGCTACCCCGACCACATGAAGCAGCACGACTTCTTCAAGTCCGCCATGCCCGAAGGCTACGTCCAGGAGCGCACCATCTCCTTCAAGGACGACGGCACCTACAAGACCCGCGCCGAGGTGAAGTTCGAGGGCGACACCCTGGTGAACCGCATCGAGCTGAAGGGCATCGACTTCAAGGAGGACGGCAACATCCTGGGGCACAAGCTGGAGTACAACTACAACAGCCACAACGTCTATATCACCGCTGACAAGCAGAAGAACGGCATCAAGGCCAACTTCAAGATCCGCCACAACATCGAGGACGGCAGCGTGCAGCTCGCCGACCACTACCAGCAGAACACCCCCATCGGCGACGGCCCCGTGCTGCTGCCCGACAACCACTACCTGAGCACCCAGTCCGCCCTGAGCAAAGACCCCAACGAGAAGCGCGATCACATGGTCCTGCTGGAGTTCGTGACCGCCGCCGGGATCACTCTCGGCATGGACGAGCTGTACAAGTAAGGATCCgactacaaggacgacgatgacaagGAAgattataaagatgatgacgataaaAGAgactacaaggacgacgatgacaagTAAAAGCTTGTTCTTCTCAGGCCTGCTGGTAATCGCAGGCCTTTTTATTTGAATTCcgcggatccttctatagtgtcacctaaatgtcgacggccaggcggccgccaggcctacccactagtcaattcgggaggatcgaaacggcagatcgcaaaaaacagtacatacagaaggagacatgaacatgaacatcaaaaaaattgtaaaacaagccacagttctgacttttacgactgcacttctggcaggaggagcgactcaagccttcgcgaaagaaaataaccaaaaagcatacaaagaaacgtacggcgtctctcatattacacgccatgatatgctgcagatccctaaacagcagcaaaacgaaaaataccaagtgcctcaattcgatcaatcaacgattaaaaatattgagtctgcaaaaggacttgatgtgtgggacagctggccgctgcaaaacgctgacggaacagtagctgaatacaacggctatcacgttgtgtttgctcttgcgggaagcccgaaagacgctgatgacacatcaatctacatgttttatcaaaaggtcggcgacaactcaatcgacagctggaaaaacgcgggccgtgtctttaaagacagcgataagttcgacgccaacgatccgatcctgaaagatcagacgcaagaatggtccggttctgcaacctttacatctgacggaaaaatccgtttattctacactgactattccggtaaacattacggcaaacaaagcctgacaacagcgcaggtaaatgtgtcaaaatctgatgacacactcaaaatcaacggagtggaagatcacaaaacgatttttgacggagacggaaaaacatatcagaacgttcagcagtttatcgatgaaggcaattatacatccggcgacaaccatacgctgagagaccctcactacgttgaagacaaaggccataaataccttgtattcgaagccaacacgggaacagaaaacggataccaaggcgaagaatctttatttaacaaagcgtactacggcggcggcacgaacttcttccgtaaagaaagccagaagcttcagcagagcgctaaaaaacgcgatgctgagttagcgaacggcgccctcggtatcatagagttaaataatgattacacattgaaaaaagtaatgaagccgctgatcacttcaaacacggtaactgatgaaatcgagcgcgcgaatgttttcaaaatgaacggcaaatggtacttgttcactgattcacgcggttcaaaaatgacgatcgatggtattaactcaaacgatatttacatgcttggttatgtatcaaactctttaaccggcccttacaagccgctgaacaaaacagggcttgtgctgcaaatgggtcttgatccaaacgatgtgacattcacttactctcacttcgcagtgccgcaagccaaaggcaacaatgtggttatcacaagctacatgacaaacagaggcttcttcgaggataaaaaggcaacatttgcgccaagcttcttaatgaacatcaaaggcaataaaacatccgttgtcaaaaacagcatcctggagcaaggacagctgacagtcaactaataacagcaaaaagaaaatgccgatacttcattggcattttcttttatttctcaacaagatggtgaattgactagtgggtagatccacaggacgggtgtggtcgccatgatcgcgtagtcgatagtggctccaagtagcgaagcgagcaggactgggcggcggccaaagcggtcggacagtgctccgagaacgggtgcgcatagaaattgcatcaacgcatatagcgctagcagcacgccatagtgactggcgatgctgtcggaatggacgatatcccgcaagaggcccggcagtaccggcataaccaagcctatgcctacagcatccagggtgacggtgccgaggatgacgatgagcgcattgttagatttcatacacggtgcctgactgcgttagcaatttaactgtgataaactaccgcattaaagcttatcgatgataagctgtcaaacatgagaattgatccggaacccttaatataacttcgtataatgtatgctatacgaagttattaggtccctcgactatagggtcaccgtcgacagcgacacacttgcatcggatgcagcccggttaacgtgccggcacggcctgggtaaccaggtattttgtccacataaccgtgcgcaaaatgttgtggataagcaggacacagcagcaatccacagcaggcatacaaccgcacaccgaggttactccgttctacaggttacgacgacatgtcaatacttgcccttgacaggcattgatggaatcgtagtctcacgctgatagtctgatcgacaatacaagtgggaccgtggtcccagaccgataatcagaccgacaacacgagtgggatcgtggtcccagactaataatcagaccgacgatacgagtgggaccgtggtcccagactaataatcagaccgacgatacgagtgggaccgtggttccagactaataatcagaccgacgatacgagtgggaccgtggtcccagactaataatcagaccgacgatacgagtgggaccatggtcccagactaataatcagaccgacgatacgagtgggaccgtggtcccagtctgattatcagaccgacgatacgagtgggaccgtggtcccagactaataatcagaccgacgatacgagtgggaccgtggtcccagactaataatcagaccgacgatacgagtgggaccgtggtcccagtctgattatcagaccgacgatacaagtggaacagtgggcccagagagaatattcaggccagttatgctttctggcctgtaacaaaggacattaagtaaagacagataaacgtagactaaaacgtggtcgcatcagggtgctggcttttcaagttccttaagaatggcctcaattttctctatacactcagttggaacacgagacctgtccaggttaagcaccattttatcgcccttatacaatactgtcgctccaggagcaaactgatgtcgtgagcttaaactagttcttgatgcagatgacgttttaagcacagaagttaaaagagtgataacttcttcagcttcaaatatcaccccagcttttttctgctcatgaaggttagatgcctgctgcttaagtaattcctctttatctgtaaaggctttttgaagtgcatcacctgaccgggcagatagttcaccggggtgagaaaaaagagcaacaactgatttaggcaatttggcggtgttgatacagcgggtaataatcttacgtgaaatattttccgcatcagccagcgcagaaatatttccagcaaattcattctgcaatcggcttgcataacgctgaccacgttcataagcacttgttgggcgataatcgttacccaatctggataatgcagccatctgctcatcatccagctcgccaaccagaacacgataatcactttcggtaagtgcagcagctttacgacggcgactcccatcggcaatttctatgacaccagatactcttcgaccgaacgccggtgtctgttgaccagtcagtagaaaagaagggatgagatcatccagtgcgtcctcagtaagcagctcctggtcacgttcattacctgaccatacccgagaggtcttctcaacactatcaccccggagcacttcaagagtaaacttcacatcccgaccacatacaggcaaagtaatggcattaccgcgagccattactcctacgcgcgcaattaacgaatccaccatcggggcagctggtgtcgataacgaagtatcttcaaccggttgagtattgagcgtatgttttggaataacaggcgcacgcttcattatctaatctcccagcgtggtttaatcagacgatcgaaaatttcattgcagacaggttcccaaatagaaagagcatttctccaggcaccagttgaagagcgttgatcaatggcctgttcaaaaacagttctcatccggatctgacctttaccaacttcatccgtttcacgtacaacattttttagaaccatgcttccccaggcatcccgaatttgctcctccatccacggggactgagagccattactattgctgtatttggtaagcaaaatacgtacatcaggctcgaaccctttaagatcaacgttcttgagcagatcacgaagcatatcgaaaaactgcagtgcggaggtgtagtcaaacaactcagcaggcgtgggaacaatcagcacatcagcagcacatacgacattaatcgtgccgatacccaggttaggcgcgctgtcaataactatgacatcatagtcatgagcaacagtttcaatggccagtcggagcatcaggtgtggatcggtgggcagtttaccttcatcaaatttgcccattaactcagtttcaatacggtgcagagccagacaggaaggaataatgtcaagccccggccagcaagtgggctttattgcataagtgacatcgtccttttccccaagatagaaaggcaggagagtgtcttctgcatgaatatgaagatctggtacccatccgtgatacattgaggctgttccctgggggtcgttaccttccacgagcaaaacacgtagccccttcagagccagatcctgagcaagatgaacagaaactgaggttttgtaaacgccacctttatgggcagcaaccccgatcaccggtggaaatacgtcttcagcacgtcgcaatcgcgtaccaaacacatcacgcatatgattaatttgttcaattgtataaccaacacgttgctcaacccgtcctcgaatttccatatccgggtgcggtagtcgccctgctttctcggcatctctgatagcctgagaagaaaccccaactaaatccgctgcttcacctattctccagcgccgggttattttcctcgcttccgggctgtcatcattaaactgtgcaatggcgatagccttcgtcatttcatgaccagcgtttatgcactggttaagtgtttccatgagtttcattctgaacatcctttaatcattgctttgcgtttttttattaaatcttgcaatttactgcaaagcaacaacaaaatcgcaaagtcatcaaaaaaccgcaaagttgtttaaaataagagcaacactacaaaaggagataagaagagcacatacctcagtcacttattatcactagcgctcgccgcagccgtgtaaccgagcatagcgagcgaactggcgaggaagcaaagaagaactgttctgtcagatagctcttacgctcagcgcaagaagaaatatccaccgtgggaaaaactccaggtagaggtacacacgcggatagccaattcagagtaataaactgtgataatcaaccctcatcaatgatgacgaactaacccccgatatcaggtcacatgacgaagggaaagagaaggaaatcaactgtgacaaactgccctcaaatttggcttccttaaaaattacagttcaaaaagtatgagaaaatccatgcaggctgaaggaaacagcaaaactgtgacaaattaccctcagtaggtcagaacaaatgtgacgaaccaccctcaaatctgtgacagataaccctcagactatcctgtcgtcatggaagtgatatcgcggaaggaaaatacgatatgagtcgtctggcggcctttctttttctcaatgtatgagaggcgcattggagttctgctgttgatctcattaacacagacctgcaggaagcggcggcggaagtcaggcatacgctggtaactttgaggcagctggtaacgctctatgatccagtcgattttcagagagacgatgcctgagccatccggcttacgatactgacacagggattcgtataaacgcatggcatacggattggtgatttcttttgtttcactaagccgaaactgcgtaaaccggttctgtaacccgataaagaagggaatgagatatgggttgatatgtacactgtaaagccctctggatggactgtgcgcacgtttgataaaccaaggaaaagattcatagcctttttcatcgccggcatcctcttcagggcgataaaaaaccacttccttccccgcgaaactcttcaatgcctgccgtatatccttactggcttccgcagaggtcaatccgaatatttcagcatatttagcaacatggatctcgcagataccgtcatgttcctgtagggtgccatcagattttctgatctggtcaacgaacagatacagcatacgtttttgatcccgggagagactatatgccgcctcagtgaggtcgtttgactggacgattcgcgggctatttttacgtttcttgtgattgataaccgctgtttccgccatgacagatccatgtgaagtgtgacaagtttttagattgtcacactaaataaaaaagagtcaataagcagggataactttgtgaaaaaacagcttcttctgagggcaatttgtcacagggttaagggcaatttgtcacagACAGGACTGTCATTTGAGGGTGATTTGTCACACTGAAAGGGCAATTTGTCACAACACCTTCTCTAGAACCAGCATGGATAAAGGCCTACAAGGCGCTCTAAAAAAGAAGATCTAAAAACTATAAAAAAAATAATTATAAAAATATCCCCGTGGATAAGTGGATAACCCCAAGGGAAGTTTTTTCAGGCATCGTGTGTAAGCAGAATATATAAGTGCTGTTCCCTGGTGCTTCCTCGCTCACTCGAGGGCTTCGCCCTGTCGCTCAACTGCGGCGAGCACTACTGGCTGTAAaaggacagaccacatcatggttctgtgttcattaggttgttctgtccattgctgacataatccgctccacttcaacgtaacaccgcacgaagatttctattgttcctgaaggcatattcaaatcgttttcgttaccgcttgcaggcatcatgacagaacactacttcctataaacgctacacaggctcctgagattaataatgcggatctctacgataatgggagattttcccgactgtttcgttcgcttctcagtggataacagccagcttctctgtttaacagacaaaaacagcatatccactcagttccacatttccatataaaggccaaggcatttattctcaggataattgtttcagcatcgcaaccgcatcagactccggcatcgcaaactgcacccggtgccgggcagccacatccagcgcaaaaaccttcgtgtagacttccgttgaactgatggacttatgtcccatcaggctttgcagaactttcagcggtataccggcatacagcatgtgcatcgcataggaatggcggaacgtatgtggtgtgaccggaacagagaacgtcacaccgtcagcagcagcggcggcaaccgcctccccaatccaggtcctgaccgttctgtccgtcacttcccagatccgcgctttctctgtccttcctgtgcgacggttacgccgctccatgagcttatcgcgaataaatacctgtgacggaagatcacttcgcagaataaataaatcctggtgtccctgttgataccgggaagccctgggccaacttttggcgaaaatgagacgttgatcggcacgtaagaggttccaactttcaccataatgaaataagatcactaccgggcgtattttttgagttatcgagattttcaggagctaaggaagctaaaatggagaaaaaaatcactggatataccaccgttgatatatcccaatggcatcgtaaagaacattttgaggcatttcagtcagttgctcaatgtacctataaccagaccgttcagctggatattacggcctttttaaagaccgtaaagaaaaataagcacaagttttatccggcctttattcacattcttgcccgcctgatgaatgctcatccggagttccgtatggcaatgaaagacggtgagctggtgatatgggatagtgttcacccttgttacaccgttttccatgagcaaactgaaacgttttcatcgctctggagtgaataccacgacgatttccggcagtttctacacatatattcgcaagatgtggcgtgttacggtgaaaacctggcctatttccctaaagggtttattgagaatatgtttttcgtctcagccaatccctgggtgagtttcaccagttttgatttaaacgtggccaatatggacaacttcttcgcccccgttttcaccatgggcaaatattatacgcaaggcgacaaggtgctgatgccgctggcgattcaggttcatcatgccgtttgtgatggcttccatgtcggcagaatgcttaatgaattacaacagtactgcgatgagtggcagggcggggcgtaatttttttaaggcagttattggtgcccttaaacgcctggttgctacgcctgaataagtgataataagcggatgaatggcagaaattcgatgataagctgtcaaacatgagaattggtcgacggcgcgccaaagcttgcatgcctgcagccgcgtaacctggcaaaatcggttacggttgagtaataaatggatgccctgcgtaagcggggcacatttcattacctctttctccgcacccgacatagataataacttcgtatagtatacattatacgaagttatctagtagacttaattaaggatcgatccggcgcgccaatagtcatgccccgcgcccaccggaaggagctgactgggttgaaggctctcaagggcatcggtcgagcttgacattgtaggactatattgctctaataaatttgcggccgctaatacgactcactatagggagag |
| pRC1796  (Bp3- EGFP Flag) | gatccgcggaattgTGGTTAATAACAGGGGACGTGGTAATCCGTCCCCTTTTTATTTCTGACTGAGTTAATAACAGGCCTGCTTCGGCAGGCCTTTTTATTTCTGACTGAGTTCTTCTCAGGCCTGCTGGTAATCGCAGGCCTTTTTATTTTCTAGaCAATTCCGACGTCTAAGAAACCATTATTATCATGACATTAACCTATAAAAATAGGCGTATCACGAGGCCCTTTCGTCTTCACCTCGAGTCCCTATCAGTGATAGAGATTGACCTCCCTATCAGTGATAGAGATACTGAGCACATCAGCAGGACGCACTGACCACTTTAAGtctcctATATACATATGGTGAGCAAGGGCGAGGAGCTGTTCACCGGGGTGGTGCCCATCCTGGTCGAGCTGGACGGCGACGTAAACGGCCACAAGTTCAGCGTGCGCGGCGAGGGCGAGGGCGATGCCACCAACGGCAAGCTGACCCTGAAGTTCATCTGCACCACCGGCAAGCTGCCCGTGCCCTGGCCCACCCTCGTGACCACCCTGACCTACGGCGTGCAGTGCTTCAGCCGCTACCCCGACCACATGAAGCAGCACGACTTCTTCAAGTCCGCCATGCCCGAAGGCTACGTCCAGGAGCGCACCATCTCCTTCAAGGACGACGGCACCTACAAGACCCGCGCCGAGGTGAAGTTCGAGGGCGACACCCTGGTGAACCGCATCGAGCTGAAGGGCATCGACTTCAAGGAGGACGGCAACATCCTGGGGCACAAGCTGGAGTACAACTACAACAGCCACAACGTCTATATCACCGCTGACAAGCAGAAGAACGGCATCAAGGCCAACTTCAAGATCCGCCACAACATCGAGGACGGCAGCGTGCAGCTCGCCGACCACTACCAGCAGAACACCCCCATCGGCGACGGCCCCGTGCTGCTGCCCGACAACCACTACCTGAGCACCCAGTCCGCCCTGAGCAAAGACCCCAACGAGAAGCGCGATCACATGGTCCTGCTGGAGTTCGTGACCGCCGCCGGGATCACTCTCGGCATGGACGAGCTGTACAAGTAAGGATCCgactacaaggacgacgatgacaagGAAgattataaagatgatgacgataaaAGAgactacaaggacgacgatgacaagTAAAAGCTTGTTCTTCTCAGGCCTGCTGGTAATCGCAGGCCTTTTTATTTGAATTCcgcggatccttctatagtgtcacctaaatgtcgacggccaggcggccgccaggcctacccactagtcaattcgggaggatcgaaacggcagatcgcaaaaaacagtacatacagaaggagacatgaacatgaacatcaaaaaaattgtaaaacaagccacagttctgacttttacgactgcacttctggcaggaggagcgactcaagccttcgcgaaagaaaataaccaaaaagcatacaaagaaacgtacggcgtctctcatattacacgccatgatatgctgcagatccctaaacagcagcaaaacgaaaaataccaagtgcctcaattcgatcaatcaacgattaaaaatattgagtctgcaaaaggacttgatgtgtgggacagctggccgctgcaaaacgctgacggaacagtagctgaatacaacggctatcacgttgtgtttgctcttgcgggaagcccgaaagacgctgatgacacatcaatctacatgttttatcaaaaggtcggcgacaactcaatcgacagctggaaaaacgcgggccgtgtctttaaagacagcgataagttcgacgccaacgatccgatcctgaaagatcagacgcaagaatggtccggttctgcaacctttacatctgacggaaaaatccgtttattctacactgactattccggtaaacattacggcaaacaaagcctgacaacagcgcaggtaaatgtgtcaaaatctgatgacacactcaaaatcaacggagtggaagatcacaaaacgatttttgacggagacggaaaaacatatcagaacgttcagcagtttatcgatgaaggcaattatacatccggcgacaaccatacgctgagagaccctcactacgttgaagacaaaggccataaataccttgtattcgaagccaacacgggaacagaaaacggataccaaggcgaagaatctttatttaacaaagcgtactacggcggcggcacgaacttcttccgtaaagaaagccagaagcttcagcagagcgctaaaaaacgcgatgctgagttagcgaacggcgccctcggtatcatagagttaaataatgattacacattgaaaaaagtaatgaagccgctgatcacttcaaacacggtaactgatgaaatcgagcgcgcgaatgttttcaaaatgaacggcaaatggtacttgttcactgattcacgcggttcaaaaatgacgatcgatggtattaactcaaacgatatttacatgcttggttatgtatcaaactctttaaccggcccttacaagccgctgaacaaaacagggcttgtgctgcaaatgggtcttgatccaaacgatgtgacattcacttactctcacttcgcagtgccgcaagccaaaggcaacaatgtggttatcacaagctacatgacaaacagaggcttcttcgaggataaaaaggcaacatttgcgccaagcttcttaatgaacatcaaaggcaataaaacatccgttgtcaaaaacagcatcctggagcaaggacagctgacagtcaactaataacagcaaaaagaaaatgccgatacttcattggcattttcttttatttctcaacaagatggtgaattgactagtgggtagatccacaggacgggtgtggtcgccatgatcgcgtagtcgatagtggctccaagtagcgaagcgagcaggactgggcggcggccaaagcggtcggacagtgctccgagaacgggtgcgcatagaaattgcatcaacgcatatagcgctagcagcacgccatagtgactggcgatgctgtcggaatggacgatatcccgcaagaggcccggcagtaccggcataaccaagcctatgcctacagcatccagggtgacggtgccgaggatgacgatgagcgcattgttagatttcatacacggtgcctgactgcgttagcaatttaactgtgataaactaccgcattaaagcttatcgatgataagctgtcaaacatgagaattgatccggaacccttaatataacttcgtataatgtatgctatacgaagttattaggtccctcgactatagggtcaccgtcgacagcgacacacttgcatcggatgcagcccggttaacgtgccggcacggcctgggtaaccaggtattttgtccacataaccgtgcgcaaaatgttgtggataagcaggacacagcagcaatccacagcaggcatacaaccgcacaccgaggttactccgttctacaggttacgacgacatgtcaatacttgcccttgacaggcattgatggaatcgtagtctcacgctgatagtctgatcgacaatacaagtgggaccgtggtcccagaccgataatcagaccgacaacacgagtgggatcgtggtcccagactaataatcagaccgacgatacgagtgggaccgtggtcccagactaataatcagaccgacgatacgagtgggaccgtggttccagactaataatcagaccgacgatacgagtgggaccgtggtcccagactaataatcagaccgacgatacgagtgggaccatggtcccagactaataatcagaccgacgatacgagtgggaccgtggtcccagtctgattatcagaccgacgatacgagtgggaccgtggtcccagactaataatcagaccgacgatacgagtgggaccgtggtcccagactaataatcagaccgacgatacgagtgggaccgtggtcccagtctgattatcagaccgacgatacaagtggaacagtgggcccagagagaatattcaggccagttatgctttctggcctgtaacaaaggacattaagtaaagacagataaacgtagactaaaacgtggtcgcatcagggtgctggcttttcaagttccttaagaatggcctcaattttctctatacactcagttggaacacgagacctgtccaggttaagcaccattttatcgcccttatacaatactgtcgctccaggagcaaactgatgtcgtgagcttaaactagttcttgatgcagatgacgttttaagcacagaagttaaaagagtgataacttcttcagcttcaaatatcaccccagcttttttctgctcatgaaggttagatgcctgctgcttaagtaattcctctttatctgtaaaggctttttgaagtgcatcacctgaccgggcagatagttcaccggggtgagaaaaaagagcaacaactgatttaggcaatttggcggtgttgatacagcgggtaataatcttacgtgaaatattttccgcatcagccagcgcagaaatatttccagcaaattcattctgcaatcggcttgcataacgctgaccacgttcataagcacttgttgggcgataatcgttacccaatctggataatgcagccatctgctcatcatccagctcgccaaccagaacacgataatcactttcggtaagtgcagcagctttacgacggcgactcccatcggcaatttctatgacaccagatactcttcgaccgaacgccggtgtctgttgaccagtcagtagaaaagaagggatgagatcatccagtgcgtcctcagtaagcagctcctggtcacgttcattacctgaccatacccgagaggtcttctcaacactatcaccccggagcacttcaagagtaaacttcacatcccgaccacatacaggcaaagtaatggcattaccgcgagccattactcctacgcgcgcaattaacgaatccaccatcggggcagctggtgtcgataacgaagtatcttcaaccggttgagtattgagcgtatgttttggaataacaggcgcacgcttcattatctaatctcccagcgtggtttaatcagacgatcgaaaatttcattgcagacaggttcccaaatagaaagagcatttctccaggcaccagttgaagagcgttgatcaatggcctgttcaaaaacagttctcatccggatctgacctttaccaacttcatccgtttcacgtacaacattttttagaaccatgcttccccaggcatcccgaatttgctcctccatccacggggactgagagccattactattgctgtatttggtaagcaaaatacgtacatcaggctcgaaccctttaagatcaacgttcttgagcagatcacgaagcatatcgaaaaactgcagtgcggaggtgtagtcaaacaactcagcaggcgtgggaacaatcagcacatcagcagcacatacgacattaatcgtgccgatacccaggttaggcgcgctgtcaataactatgacatcatagtcatgagcaacagtttcaatggccagtcggagcatcaggtgtggatcggtgggcagtttaccttcatcaaatttgcccattaactcagtttcaatacggtgcagagccagacaggaaggaataatgtcaagccccggccagcaagtgggctttattgcataagtgacatcgtccttttccccaagatagaaaggcaggagagtgtcttctgcatgaatatgaagatctggtacccatccgtgatacattgaggctgttccctgggggtcgttaccttccacgagcaaaacacgtagccccttcagagccagatcctgagcaagatgaacagaaactgaggttttgtaaacgccacctttatgggcagcaaccccgatcaccggtggaaatacgtcttcagcacgtcgcaatcgcgtaccaaacacatcacgcatatgattaatttgttcaattgtataaccaacacgttgctcaacccgtcctcgaatttccatatccgggtgcggtagtcgccctgctttctcggcatctctgatagcctgagaagaaaccccaactaaatccgctgcttcacctattctccagcgccgggttattttcctcgcttccgggctgtcatcattaaactgtgcaatggcgatagccttcgtcatttcatgaccagcgtttatgcactggttaagtgtttccatgagtttcattctgaacatcctttaatcattgctttgcgtttttttattaaatcttgcaatttactgcaaagcaacaacaaaatcgcaaagtcatcaaaaaaccgcaaagttgtttaaaataagagcaacactacaaaaggagataagaagagcacatacctcagtcacttattatcactagcgctcgccgcagccgtgtaaccgagcatagcgagcgaactggcgaggaagcaaagaagaactgttctgtcagatagctcttacgctcagcgcaagaagaaatatccaccgtgggaaaaactccaggtagaggtacacacgcggatagccaattcagagtaataaactgtgataatcaaccctcatcaatgatgacgaactaacccccgatatcaggtcacatgacgaagggaaagagaaggaaatcaactgtgacaaactgccctcaaatttggcttccttaaaaattacagttcaaaaagtatgagaaaatccatgcaggctgaaggaaacagcaaaactgtgacaaattaccctcagtaggtcagaacaaatgtgacgaaccaccctcaaatctgtgacagataaccctcagactatcctgtcgtcatggaagtgatatcgcggaaggaaaatacgatatgagtcgtctggcggcctttctttttctcaatgtatgagaggcgcattggagttctgctgttgatctcattaacacagacctgcaggaagcggcggcggaagtcaggcatacgctggtaactttgaggcagctggtaacgctctatgatccagtcgattttcagagagacgatgcctgagccatccggcttacgatactgacacagggattcgtataaacgcatggcatacggattggtgatttcttttgtttcactaagccgaaactgcgtaaaccggttctgtaacccgataaagaagggaatgagatatgggttgatatgtacactgtaaagccctctggatggactgtgcgcacgtttgataaaccaaggaaaagattcatagcctttttcatcgccggcatcctcttcagggcgataaaaaaccacttccttccccgcgaaactcttcaatgcctgccgtatatccttactggcttccgcagaggtcaatccgaatatttcagcatatttagcaacatggatctcgcagataccgtcatgttcctgtagggtgccatcagattttctgatctggtcaacgaacagatacagcatacgtttttgatcccgggagagactatatgccgcctcagtgaggtcgtttgactggacgattcgcgggctatttttacgtttcttgtgattgataaccgctgtttccgccatgacagatccatgtgaagtgtgacaagtttttagattgtcacactaaataaaaaagagtcaataagcagggataactttgtgaaaaaacagcttcttctgagggcaatttgtcacagggttaagggcaatttgtcacagACAGGACTGTCATTTGAGGGTGATTTGTCACACTGAAAGGGCAATTTGTCACAACACCTTCTCTAGAACCAGCATGGATAAAGGCCTACAAGGCGCTCTAAAAAAGAAGATCTAAAAACTATAAAAAAAATAATTATAAAAATATCCCCGTGGATAAGTGGATAACCCCAAGGGAAGTTTTTTCAGGCATCGTGTGTAAGCAGAATATATAAGTGCTGTTCCCTGGTGCTTCCTCGCTCACTCGAGGGCTTCGCCCTGTCGCTCAACTGCGGCGAGCACTACTGGCTGTAAaaggacagaccacatcatggttctgtgttcattaggttgttctgtccattgctgacataatccgctccacttcaacgtaacaccgcacgaagatttctattgttcctgaaggcatattcaaatcgttttcgttaccgcttgcaggcatcatgacagaacactacttcctataaacgctacacaggctcctgagattaataatgcggatctctacgataatgggagattttcccgactgtttcgttcgcttctcagtggataacagccagcttctctgtttaacagacaaaaacagcatatccactcagttccacatttccatataaaggccaaggcatttattctcaggataattgtttcagcatcgcaaccgcatcagactccggcatcgcaaactgcacccggtgccgggcagccacatccagcgcaaaaaccttcgtgtagacttccgttgaactgatggacttatgtcccatcaggctttgcagaactttcagcggtataccggcatacagcatgtgcatcgcataggaatggcggaacgtatgtggtgtgaccggaacagagaacgtcacaccgtcagcagcagcggcggcaaccgcctccccaatccaggtcctgaccgttctgtccgtcacttcccagatccgcgctttctctgtccttcctgtgcgacggttacgccgctccatgagcttatcgcgaataaatacctgtgacggaagatcacttcgcagaataaataaatcctggtgtccctgttgataccgggaagccctgggccaacttttggcgaaaatgagacgttgatcggcacgtaagaggttccaactttcaccataatgaaataagatcactaccgggcgtattttttgagttatcgagattttcaggagctaaggaagctaaaatggagaaaaaaatcactggatataccaccgttgatatatcccaatggcatcgtaaagaacattttgaggcatttcagtcagttgctcaatgtacctataaccagaccgttcagctggatattacggcctttttaaagaccgtaaagaaaaataagcacaagttttatccggcctttattcacattcttgcccgcctgatgaatgctcatccggagttccgtatggcaatgaaagacggtgagctggtgatatgggatagtgttcacccttgttacaccgttttccatgagcaaactgaaacgttttcatcgctctggagtgaataccacgacgatttccggcagtttctacacatatattcgcaagatgtggcgtgttacggtgaaaacctggcctatttccctaaagggtttattgagaatatgtttttcgtctcagccaatccctgggtgagtttcaccagttttgatttaaacgtggccaatatggacaacttcttcgcccccgttttcaccatgggcaaatattatacgcaaggcgacaaggtgctgatgccgctggcgattcaggttcatcatgccgtttgtgatggcttccatgtcggcagaatgcttaatgaattacaacagtactgcgatgagtggcagggcggggcgtaatttttttaaggcagttattggtgcccttaaacgcctggttgctacgcctgaataagtgataataagcggatgaatggcagaaattcgatgataagctgtcaaacatgagaattggtcgacggcgcgccaaagcttgcatgcctgcagccgcgtaacctggcaaaatcggttacggttgagtaataaatggatgccctgcgtaagcggggcacatttcattacctctttctccgcacccgacatagataataacttcgtatagtatacattatacgaagttatctagtagacttaattaaggatcgatccggcgcgccaatagtcatgccccgcgcccaccggaaggagctgactgggttgaaggctctcaagggcatcggtcgagcttgacattgtaggactatattgctctaataaatttgcggccgctaatacgactcactatagggagag |
| pRC1797  (Bp4- EGFP Flag) | gatccgcggaattgTGGTTAATAACAGGGGACGTGGTAATCCGTCCCCTTTTTATTTCTGACTGAGTTAATAACAGGCCTGCTTCGGCAGGCCTTTTTATTTCTGACTGAGTTCTTCTCAGGCCTGCTGGTAATCGCAGGCCTTTTTATTTTCTAGgCAATTCCGACGTCTAAGAAACCATTATTATCATGACATTAACCTATAAAAATAGGCGTATCACGAGGCCCTCTCGTCTTCACCTCGAGTCCCTATCAGTGATAGGGATTGACATCCCTATCAGTGATAGAGACACTGGGCACATCAGCAGGACGCACTGACCACTTTAAGtctcctATATACATATGGTGAGCAAGGGCGAGGAGCTGTTCACCGGGGTGGTGCCCATCCTGGTCGAGCTGGACGGCGACGTAAACGGCCACAAGTTCAGCGTGCGCGGCGAGGGCGAGGGCGATGCCACCAACGGCAAGCTGACCCTGAAGTTCATCTGCACCACCGGCAAGCTGCCCGTGCCCTGGCCCACCCTCGTGACCACCCTGACCTACGGCGTGCAGTGCTTCAGCCGCTACCCCGACCACATGAAGCAGCACGACTTCTTCAAGTCCGCCATGCCCGAAGGCTACGTCCAGGAGCGCACCATCTCCTTCAAGGACGACGGCACCTACAAGACCCGCGCCGAGGTGAAGTTCGAGGGCGACACCCTGGTGAACCGCATCGAGCTGAAGGGCATCGACTTCAAGGAGGACGGCAACATCCTGGGGCACAAGCTGGAGTACAACTACAACAGCCACAACGTCTATATCACCGCTGACAAGCAGAAGAACGGCATCAAGGCCAACTTCAAGATCCGCCACAACATCGAGGACGGCAGCGTGCAGCTCGCCGACCACTACCAGCAGAACACCCCCATCGGCGACGGCCCCGTGCTGCTGCCCGACAACCACTACCTGAGCACCCAGTCCGCCCTGAGCAAAGACCCCAACGAGAAGCGCGATCACATGGTCCTGCTGGAGTTCGTGACCGCCGCCGGGATCACTCTCGGCATGGACGAGCTGTACAAGTAAGGATCCgactacaaggacgacgatgacaagGAAgattataaagatgatgacgataaaAGAgactacaaggacgacgatgacaagTAAAAGCTTGTTCTTCTCAGGCCTGCTGGTAATCGCAGGCCTTTTTATTTGAATTCcgcggatccttctatagtgtcacctaaatgtcgacggccaggcggccgccaggcctacccactagtcaattcgggaggatcgaaacggcagatcgcaaaaaacagtacatacagaaggagacatgaacatgaacatcaaaaaaattgtaaaacaagccacagttctgacttttacgactgcacttctggcaggaggagcgactcaagccttcgcgaaagaaaataaccaaaaagcatacaaagaaacgtacggcgtctctcatattacacgccatgatatgctgcagatccctaaacagcagcaaaacgaaaaataccaagtgcctcaattcgatcaatcaacgattaaaaatattgagtctgcaaaaggacttgatgtgtgggacagctggccgctgcaaaacgctgacggaacagtagctgaatacaacggctatcacgttgtgtttgctcttgcgggaagcccgaaagacgctgatgacacatcaatctacatgttttatcaaaaggtcggcgacaactcaatcgacagctggaaaaacgcgggccgtgtctttaaagacagcgataagttcgacgccaacgatccgatcctgaaagatcagacgcaagaatggtccggttctgcaacctttacatctgacggaaaaatccgtttattctacactgactattccggtaaacattacggcaaacaaagcctgacaacagcgcaggtaaatgtgtcaaaatctgatgacacactcaaaatcaacggagtggaagatcacaaaacgatttttgacggagacggaaaaacatatcagaacgttcagcagtttatcgatgaaggcaattatacatccggcgacaaccatacgctgagagaccctcactacgttgaagacaaaggccataaataccttgtattcgaagccaacacgggaacagaaaacggataccaaggcgaagaatctttatttaacaaagcgtactacggcggcggcacgaacttcttccgtaaagaaagccagaagcttcagcagagcgctaaaaaacgcgatgctgagttagcgaacggcgccctcggtatcatagagttaaataatgattacacattgaaaaaagtaatgaagccgctgatcacttcaaacacggtaactgatgaaatcgagcgcgcgaatgttttcaaaatgaacggcaaatggtacttgttcactgattcacgcggttcaaaaatgacgatcgatggtattaactcaaacgatatttacatgcttggttatgtatcaaactctttaaccggcccttacaagccgctgaacaaaacagggcttgtgctgcaaatgggtcttgatccaaacgatgtgacattcacttactctcacttcgcagtgccgcaagccaaaggcaacaatgtggttatcacaagctacatgacaaacagaggcttcttcgaggataaaaaggcaacatttgcgccaagcttcttaatgaacatcaaaggcaataaaacatccgttgtcaaaaacagcatcctggagcaaggacagctgacagtcaactaataacagcaaaaagaaaatgccgatacttcattggcattttcttttatttctcaacaagatggtgaattgactagtgggtagatccacaggacgggtgtggtcgccatgatcgcgtagtcgatagtggctccaagtagcgaagcgagcaggactgggcggcggccaaagcggtcggacagtgctccgagaacgggtgcgcatagaaattgcatcaacgcatatagcgctagcagcacgccatagtgactggcgatgctgtcggaatggacgatatcccgcaagaggcccggcagtaccggcataaccaagcctatgcctacagcatccagggtgacggtgccgaggatgacgatgagcgcattgttagatttcatacacggtgcctgactgcgttagcaatttaactgtgataaactaccgcattaaagcttatcgatgataagctgtcaaacatgagaattgatccggaacccttaatataacttcgtataatgtatgctatacgaagttattaggtccctcgactatagggtcaccgtcgacagcgacacacttgcatcggatgcagcccggttaacgtgccggcacggcctgggtaaccaggtattttgtccacataaccgtgcgcaaaatgttgtggataagcaggacacagcagcaatccacagcaggcatacaaccgcacaccgaggttactccgttctacaggttacgacgacatgtcaatacttgcccttgacaggcattgatggaatcgtagtctcacgctgatagtctgatcgacaatacaagtgggaccgtggtcccagaccgataatcagaccgacaacacgagtgggatcgtggtcccagactaataatcagaccgacgatacgagtgggaccgtggtcccagactaataatcagaccgacgatacgagtgggaccgtggttccagactaataatcagaccgacgatacgagtgggaccgtggtcccagactaataatcagaccgacgatacgagtgggaccatggtcccagactaataatcagaccgacgatacgagtgggaccgtggtcccagtctgattatcagaccgacgatacgagtgggaccgtggtcccagactaataatcagaccgacgatacgagtgggaccgtggtcccagactaataatcagaccgacgatacgagtgggaccgtggtcccagtctgattatcagaccgacgatacaagtggaacagtgggcccagagagaatattcaggccagttatgctttctggcctgtaacaaaggacattaagtaaagacagataaacgtagactaaaacgtggtcgcatcagggtgctggcttttcaagttccttaagaatggcctcaattttctctatacactcagttggaacacgagacctgtccaggttaagcaccattttatcgcccttatacaatactgtcgctccaggagcaaactgatgtcgtgagcttaaactagttcttgatgcagatgacgttttaagcacagaagttaaaagagtgataacttcttcagcttcaaatatcaccccagcttttttctgctcatgaaggttagatgcctgctgcttaagtaattcctctttatctgtaaaggctttttgaagtgcatcacctgaccgggcagatagttcaccggggtgagaaaaaagagcaacaactgatttaggcaatttggcggtgttgatacagcgggtaataatcttacgtgaaatattttccgcatcagccagcgcagaaatatttccagcaaattcattctgcaatcggcttgcataacgctgaccacgttcataagcacttgttgggcgataatcgttacccaatctggataatgcagccatctgctcatcatccagctcgccaaccagaacacgataatcactttcggtaagtgcagcagctttacgacggcgactcccatcggcaatttctatgacaccagatactcttcgaccgaacgccggtgtctgttgaccagtcagtagaaaagaagggatgagatcatccagtgcgtcctcagtaagcagctcctggtcacgttcattacctgaccatacccgagaggtcttctcaacactatcaccccggagcacttcaagagtaaacttcacatcccgaccacatacaggcaaagtaatggcattaccgcgagccattactcctacgcgcgcaattaacgaatccaccatcggggcagctggtgtcgataacgaagtatcttcaaccggttgagtattgagcgtatgttttggaataacaggcgcacgcttcattatctaatctcccagcgtggtttaatcagacgatcgaaaatttcattgcagacaggttcccaaatagaaagagcatttctccaggcaccagttgaagagcgttgatcaatggcctgttcaaaaacagttctcatccggatctgacctttaccaacttcatccgtttcacgtacaacattttttagaaccatgcttccccaggcatcccgaatttgctcctccatccacggggactgagagccattactattgctgtatttggtaagcaaaatacgtacatcaggctcgaaccctttaagatcaacgttcttgagcagatcacgaagcatatcgaaaaactgcagtgcggaggtgtagtcaaacaactcagcaggcgtgggaacaatcagcacatcagcagcacatacgacattaatcgtgccgatacccaggttaggcgcgctgtcaataactatgacatcatagtcatgagcaacagtttcaatggccagtcggagcatcaggtgtggatcggtgggcagtttaccttcatcaaatttgcccattaactcagtttcaatacggtgcagagccagacaggaaggaataatgtcaagccccggccagcaagtgggctttattgcataagtgacatcgtccttttccccaagatagaaaggcaggagagtgtcttctgcatgaatatgaagatctggtacccatccgtgatacattgaggctgttccctgggggtcgttaccttccacgagcaaaacacgtagccccttcagagccagatcctgagcaagatgaacagaaactgaggttttgtaaacgccacctttatgggcagcaaccccgatcaccggtggaaatacgtcttcagcacgtcgcaatcgcgtaccaaacacatcacgcatatgattaatttgttcaattgtataaccaacacgttgctcaacccgtcctcgaatttccatatccgggtgcggtagtcgccctgctttctcggcatctctgatagcctgagaagaaaccccaactaaatccgctgcttcacctattctccagcgccgggttattttcctcgcttccgggctgtcatcattaaactgtgcaatggcgatagccttcgtcatttcatgaccagcgtttatgcactggttaagtgtttccatgagtttcattctgaacatcctttaatcattgctttgcgtttttttattaaatcttgcaatttactgcaaagcaacaacaaaatcgcaaagtcatcaaaaaaccgcaaagttgtttaaaataagagcaacactacaaaaggagataagaagagcacatacctcagtcacttattatcactagcgctcgccgcagccgtgtaaccgagcatagcgagcgaactggcgaggaagcaaagaagaactgttctgtcagatagctcttacgctcagcgcaagaagaaatatccaccgtgggaaaaactccaggtagaggtacacacgcggatagccaattcagagtaataaactgtgataatcaaccctcatcaatgatgacgaactaacccccgatatcaggtcacatgacgaagggaaagagaaggaaatcaactgtgacaaactgccctcaaatttggcttccttaaaaattacagttcaaaaagtatgagaaaatccatgcaggctgaaggaaacagcaaaactgtgacaaattaccctcagtaggtcagaacaaatgtgacgaaccaccctcaaatctgtgacagataaccctcagactatcctgtcgtcatggaagtgatatcgcggaaggaaaatacgatatgagtcgtctggcggcctttctttttctcaatgtatgagaggcgcattggagttctgctgttgatctcattaacacagacctgcaggaagcggcggcggaagtcaggcatacgctggtaactttgaggcagctggtaacgctctatgatccagtcgattttcagagagacgatgcctgagccatccggcttacgatactgacacagggattcgtataaacgcatggcatacggattggtgatttcttttgtttcactaagccgaaactgcgtaaaccggttctgtaacccgataaagaagggaatgagatatgggttgatatgtacactgtaaagccctctggatggactgtgcgcacgtttgataaaccaaggaaaagattcatagcctttttcatcgccggcatcctcttcagggcgataaaaaaccacttccttccccgcgaaactcttcaatgcctgccgtatatccttactggcttccgcagaggtcaatccgaatatttcagcatatttagcaacatggatctcgcagataccgtcatgttcctgtagggtgccatcagattttctgatctggtcaacgaacagatacagcatacgtttttgatcccgggagagactatatgccgcctcagtgaggtcgtttgactggacgattcgcgggctatttttacgtttcttgtgattgataaccgctgtttccgccatgacagatccatgtgaagtgtgacaagtttttagattgtcacactaaataaaaaagagtcaataagcagggataactttgtgaaaaaacagcttcttctgagggcaatttgtcacagggttaagggcaatttgtcacagACAGGACTGTCATTTGAGGGTGATTTGTCACACTGAAAGGGCAATTTGTCACAACACCTTCTCTAGAACCAGCATGGATAAAGGCCTACAAGGCGCTCTAAAAAAGAAGATCTAAAAACTATAAAAAAAATAATTATAAAAATATCCCCGTGGATAAGTGGATAACCCCAAGGGAAGTTTTTTCAGGCATCGTGTGTAAGCAGAATATATAAGTGCTGTTCCCTGGTGCTTCCTCGCTCACTCGAGGGCTTCGCCCTGTCGCTCAACTGCGGCGAGCACTACTGGCTGTAAaaggacagaccacatcatggttctgtgttcattaggttgttctgtccattgctgacataatccgctccacttcaacgtaacaccgcacgaagatttctattgttcctgaaggcatattcaaatcgttttcgttaccgcttgcaggcatcatgacagaacactacttcctataaacgctacacaggctcctgagattaataatgcggatctctacgataatgggagattttcccgactgtttcgttcgcttctcagtggataacagccagcttctctgtttaacagacaaaaacagcatatccactcagttccacatttccatataaaggccaaggcatttattctcaggataattgtttcagcatcgcaaccgcatcagactccggcatcgcaaactgcacccggtgccgggcagccacatccagcgcaaaaaccttcgtgtagacttccgttgaactgatggacttatgtcccatcaggctttgcagaactttcagcggtataccggcatacagcatgtgcatcgcataggaatggcggaacgtatgtggtgtgaccggaacagagaacgtcacaccgtcagcagcagcggcggcaaccgcctccccaatccaggtcctgaccgttctgtccgtcacttcccagatccgcgctttctctgtccttcctgtgcgacggttacgccgctccatgagcttatcgcgaataaatacctgtgacggaagatcacttcgcagaataaataaatcctggtgtccctgttgataccgggaagccctgggccaacttttggcgaaaatgagacgttgatcggcacgtaagaggttccaactttcaccataatgaaataagatcactaccgggcgtattttttgagttatcgagattttcaggagctaaggaagctaaaatggagaaaaaaatcactggatataccaccgttgatatatcccaatggcatcgtaaagaacattttgaggcatttcagtcagttgctcaatgtacctataaccagaccgttcagctggatattacggcctttttaaagaccgtaaagaaaaataagcacaagttttatccggcctttattcacattcttgcccgcctgatgaatgctcatccggagttccgtatggcaatgaaagacggtgagctggtgatatgggatagtgttcacccttgttacaccgttttccatgagcaaactgaaacgttttcatcgctctggagtgaataccacgacgatttccggcagtttctacacatatattcgcaagatgtggcgtgttacggtgaaaacctggcctatttccctaaagggtttattgagaatatgtttttcgtctcagccaatccctgggtgagtttcaccagttttgatttaaacgtggccaatatggacaacttcttcgcccccgttttcaccatgggcaaatattatacgcaaggcgacaaggtgctgatgccgctggcgattcaggttcatcatgccgtttgtgatggcttccatgtcggcagaatgcttaatgaattacaacagtactgcgatgagtggcagggcggggcgtaatttttttaaggcagttattggtgcccttaaacgcctggttgctacgcctgaataagtgataataagcggatgaatggcagaaattcgatgataagctgtcaaacatgagaattggtcgacggcgcgccaaagcttgcatgcctgcagccgcgtaacctggcaaaatcggttacggttgagtaataaatggatgccctgcgtaagcggggcacatttcattacctctttctccgcacccgacatagataataacttcgtatagtatacattatacgaagttatctagtagacttaattaaggatcgatccggcgcgccaatagtcatgccccgcgcccaccggaaggagctgactgggttgaaggctctcaagggcatcggtcgagcttgacattgtaggactatattgctctaataaatttgcggccgctaatacgactcactatagggagag |
| pRC1798  (Bp5- EGFP Flag) | gatccgcggaattgGTTAATAACAGGGGACGTGGTAATCCGTCCCCTTTTTATTTCTGACTGAGTTAATAACAGGCCTGCTTCGGCAGGCCTTTTTATTTCTGACTGAGTTCTTCTCAGGCCTGCTGGTAATCGCAGGCCTTTTTATTTTCTAGcCAATTCCGACGCCTAAGAAACCATTATTATCATGACATTAGCCTATAAAAATAGGCGTACCACGAGGCCCTTTCGTCTTCACCTCGAGTCCCTATCAGTGATAGAGATTGACACCCCTATCAGTGATAGAGATACTGAGCACATCAGCAGGACGCACTGACCACTTTAAGtctcctATATACATATGGTGAGCAAGGGCGAGGAGCTGTTCACCGGGGTGGTGCCCATCCTGGTCGAGCTGGACGGCGACGTAAACGGCCACAAGTTCAGCGTGCGCGGCGAGGGCGAGGGCGATGCCACCAACGGCAAGCTGACCCTGAAGTTCATCTGCACCACCGGCAAGCTGCCCGTGCCCTGGCCCACCCTCGTGACCACCCTGACCTACGGCGTGCAGTGCTTCAGCCGCTACCCCGACCACATGAAGCAGCACGACTTCTTCAAGTCCGCCATGCCCGAAGGCTACGTCCAGGAGCGCACCATCTCCTTCAAGGACGACGGCACCTACAAGACCCGCGCCGAGGTGAAGTTCGAGGGCGACACCCTGGTGAACCGCATCGAGCTGAAGGGCATCGACTTCAAGGAGGACGGCAACATCCTGGGGCACAAGCTGGAGTACAACTACAACAGCCACAACGTCTATATCACCGCTGACAAGCAGAAGAACGGCATCAAGGCCAACTTCAAGATCCGCCACAACATCGAGGACGGCAGCGTGCAGCTCGCCGACCACTACCAGCAGAACACCCCCATCGGCGACGGCCCCGTGCTGCTGCCCGACAACCACTACCTGAGCACCCAGTCCGCCCTGAGCAAAGACCCCAACGAGAAGCGCGATCACATGGTCCTGCTGGAGTTCGTGACCGCCGCCGGGATCACTCTCGGCATGGACGAGCTGTACAAGTAAGGATCCgactacaaggacgacgatgacaagGAAgattataaagatgatgacgataaaAGAgactacaaggacgacgatgacaagTAAAAGCTTGTTCTTCTCAGGCCTGCTGGTAATCGCAGGCCTTTTTATTTGAATTCcgcggatccttctatagtgtcacctaaatgtcgacggccaggcggccgccaggcctacccactagtcaattcgggaggatcgaaacggcagatcgcaaaaaacagtacatacagaaggagacatgaacatgaacatcaaaaaaattgtaaaacaagccacagttctgacttttacgactgcacttctggcaggaggagcgactcaagccttcgcgaaagaaaataaccaaaaagcatacaaagaaacgtacggcgtctctcatattacacgccatgatatgctgcagatccctaaacagcagcaaaacgaaaaataccaagtgcctcaattcgatcaatcaacgattaaaaatattgagtctgcaaaaggacttgatgtgtgggacagctggccgctgcaaaacgctgacggaacagtagctgaatacaacggctatcacgttgtgtttgctcttgcgggaagcccgaaagacgctgatgacacatcaatctacatgttttatcaaaaggtcggcgacaactcaatcgacagctggaaaaacgcgggccgtgtctttaaagacagcgataagttcgacgccaacgatccgatcctgaaagatcagacgcaagaatggtccggttctgcaacctttacatctgacggaaaaatccgtttattctacactgactattccggtaaacattacggcaaacaaagcctgacaacagcgcaggtaaatgtgtcaaaatctgatgacacactcaaaatcaacggagtggaagatcacaaaacgatttttgacggagacggaaaaacatatcagaacgttcagcagtttatcgatgaaggcaattatacatccggcgacaaccatacgctgagagaccctcactacgttgaagacaaaggccataaataccttgtattcgaagccaacacgggaacagaaaacggataccaaggcgaagaatctttatttaacaaagcgtactacggcggcggcacgaacttcttccgtaaagaaagccagaagcttcagcagagcgctaaaaaacgcgatgctgagttagcgaacggcgccctcggtatcatagagttaaataatgattacacattgaaaaaagtaatgaagccgctgatcacttcaaacacggtaactgatgaaatcgagcgcgcgaatgttttcaaaatgaacggcaaatggtacttgttcactgattcacgcggttcaaaaatgacgatcgatggtattaactcaaacgatatttacatgcttggttatgtatcaaactctttaaccggcccttacaagccgctgaacaaaacagggcttgtgctgcaaatgggtcttgatccaaacgatgtgacattcacttactctcacttcgcagtgccgcaagccaaaggcaacaatgtggttatcacaagctacatgacaaacagaggcttcttcgaggataaaaaggcaacatttgcgccaagcttcttaatgaacatcaaaggcaataaaacatccgttgtcaaaaacagcatcctggagcaaggacagctgacagtcaactaataacagcaaaaagaaaatgccgatacttcattggcattttcttttatttctcaacaagatggtgaattgactagtgggtagatccacaggacgggtgtggtcgccatgatcgcgtagtcgatagtggctccaagtagcgaagcgagcaggactgggcggcggccaaagcggtcggacagtgctccgagaacgggtgcgcatagaaattgcatcaacgcatatagcgctagcagcacgccatagtgactggcgatgctgtcggaatggacgatatcccgcaagaggcccggcagtaccggcataaccaagcctatgcctacagcatccagggtgacggtgccgaggatgacgatgagcgcattgttagatttcatacacggtgcctgactgcgttagcaatttaactgtgataaactaccgcattaaagcttatcgatgataagctgtcaaacatgagaattgatccggaacccttaatataacttcgtataatgtatgctatacgaagttattaggtccctcgactatagggtcaccgtcgacagcgacacacttgcatcggatgcagcccggttaacgtgccggcacggcctgggtaaccaggtattttgtccacataaccgtgcgcaaaatgttgtggataagcaggacacagcagcaatccacagcaggcatacaaccgcacaccgaggttactccgttctacaggttacgacgacatgtcaatacttgcccttgacaggcattgatggaatcgtagtctcacgctgatagtctgatcgacaatacaagtgggaccgtggtcccagaccgataatcagaccgacaacacgagtgggatcgtggtcccagactaataatcagaccgacgatacgagtgggaccgtggtcccagactaataatcagaccgacgatacgagtgggaccgtggttccagactaataatcagaccgacgatacgagtgggaccgtggtcccagactaataatcagaccgacgatacgagtgggaccatggtcccagactaataatcagaccgacgatacgagtgggaccgtggtcccagtctgattatcagaccgacgatacgagtgggaccgtggtcccagactaataatcagaccgacgatacgagtgggaccgtggtcccagactaataatcagaccgacgatacgagtgggaccgtggtcccagtctgattatcagaccgacgatacaagtggaacagtgggcccagagagaatattcaggccagttatgctttctggcctgtaacaaaggacattaagtaaagacagataaacgtagactaaaacgtggtcgcatcagggtgctggcttttcaagttccttaagaatggcctcaattttctctatacactcagttggaacacgagacctgtccaggttaagcaccattttatcgcccttatacaatactgtcgctccaggagcaaactgatgtcgtgagcttaaactagttcttgatgcagatgacgttttaagcacagaagttaaaagagtgataacttcttcagcttcaaatatcaccccagcttttttctgctcatgaaggttagatgcctgctgcttaagtaattcctctttatctgtaaaggctttttgaagtgcatcacctgaccgggcagatagttcaccggggtgagaaaaaagagcaacaactgatttaggcaatttggcggtgttgatacagcgggtaataatcttacgtgaaatattttccgcatcagccagcgcagaaatatttccagcaaattcattctgcaatcggcttgcataacgctgaccacgttcataagcacttgttgggcgataatcgttacccaatctggataatgcagccatctgctcatcatccagctcgccaaccagaacacgataatcactttcggtaagtgcagcagctttacgacggcgactcccatcggcaatttctatgacaccagatactcttcgaccgaacgccggtgtctgttgaccagtcagtagaaaagaagggatgagatcatccagtgcgtcctcagtaagcagctcctggtcacgttcattacctgaccatacccgagaggtcttctcaacactatcaccccggagcacttcaagagtaaacttcacatcccgaccacatacaggcaaagtaatggcattaccgcgagccattactcctacgcgcgcaattaacgaatccaccatcggggcagctggtgtcgataacgaagtatcttcaaccggttgagtattgagcgtatgttttggaataacaggcgcacgcttcattatctaatctcccagcgtggtttaatcagacgatcgaaaatttcattgcagacaggttcccaaatagaaagagcatttctccaggcaccagttgaagagcgttgatcaatggcctgttcaaaaacagttctcatccggatctgacctttaccaacttcatccgtttcacgtacaacattttttagaaccatgcttccccaggcatcccgaatttgctcctccatccacggggactgagagccattactattgctgtatttggtaagcaaaatacgtacatcaggctcgaaccctttaagatcaacgttcttgagcagatcacgaagcatatcgaaaaactgcagtgcggaggtgtagtcaaacaactcagcaggcgtgggaacaatcagcacatcagcagcacatacgacattaatcgtgccgatacccaggttaggcgcgctgtcaataactatgacatcatagtcatgagcaacagtttcaatggccagtcggagcatcaggtgtggatcggtgggcagtttaccttcatcaaatttgcccattaactcagtttcaatacggtgcagagccagacaggaaggaataatgtcaagccccggccagcaagtgggctttattgcataagtgacatcgtccttttccccaagatagaaaggcaggagagtgtcttctgcatgaatatgaagatctggtacccatccgtgatacattgaggctgttccctgggggtcgttaccttccacgagcaaaacacgtagccccttcagagccagatcctgagcaagatgaacagaaactgaggttttgtaaacgccacctttatgggcagcaaccccgatcaccggtggaaatacgtcttcagcacgtcgcaatcgcgtaccaaacacatcacgcatatgattaatttgttcaattgtataaccaacacgttgctcaacccgtcctcgaatttccatatccgggtgcggtagtcgccctgctttctcggcatctctgatagcctgagaagaaaccccaactaaatccgctgcttcacctattctccagcgccgggttattttcctcgcttccgggctgtcatcattaaactgtgcaatggcgatagccttcgtcatttcatgaccagcgtttatgcactggttaagtgtttccatgagtttcattctgaacatcctttaatcattgctttgcgtttttttattaaatcttgcaatttactgcaaagcaacaacaaaatcgcaaagtcatcaaaaaaccgcaaagttgtttaaaataagagcaacactacaaaaggagataagaagagcacatacctcagtcacttattatcactagcgctcgccgcagccgtgtaaccgagcatagcgagcgaactggcgaggaagcaaagaagaactgttctgtcagatagctcttacgctcagcgcaagaagaaatatccaccgtgggaaaaactccaggtagaggtacacacgcggatagccaattcagagtaataaactgtgataatcaaccctcatcaatgatgacgaactaacccccgatatcaggtcacatgacgaagggaaagagaaggaaatcaactgtgacaaactgccctcaaatttggcttccttaaaaattacagttcaaaaagtatgagaaaatccatgcaggctgaaggaaacagcaaaactgtgacaaattaccctcagtaggtcagaacaaatgtgacgaaccaccctcaaatctgtgacagataaccctcagactatcctgtcgtcatggaagtgatatcgcggaaggaaaatacgatatgagtcgtctggcggcctttctttttctcaatgtatgagaggcgcattggagttctgctgttgatctcattaacacagacctgcaggaagcggcggcggaagtcaggcatacgctggtaactttgaggcagctggtaacgctctatgatccagtcgattttcagagagacgatgcctgagccatccggcttacgatactgacacagggattcgtataaacgcatggcatacggattggtgatttcttttgtttcactaagccgaaactgcgtaaaccggttctgtaacccgataaagaagggaatgagatatgggttgatatgtacactgtaaagccctctggatggactgtgcgcacgtttgataaaccaaggaaaagattcatagcctttttcatcgccggcatcctcttcagggcgataaaaaaccacttccttccccgcgaaactcttcaatgcctgccgtatatccttactggcttccgcagaggtcaatccgaatatttcagcatatttagcaacatggatctcgcagataccgtcatgttcctgtagggtgccatcagattttctgatctggtcaacgaacagatacagcatacgtttttgatcccgggagagactatatgccgcctcagtgaggtcgtttgactggacgattcgcgggctatttttacgtttcttgtgattgataaccgctgtttccgccatgacagatccatgtgaagtgtgacaagtttttagattgtcacactaaataaaaaagagtcaataagcagggataactttgtgaaaaaacagcttcttctgagggcaatttgtcacagggttaagggcaatttgtcacagACAGGACTGTCATTTGAGGGTGATTTGTCACACTGAAAGGGCAATTTGTCACAACACCTTCTCTAGAACCAGCATGGATAAAGGCCTACAAGGCGCTCTAAAAAAGAAGATCTAAAAACTATAAAAAAAATAATTATAAAAATATCCCCGTGGATAAGTGGATAACCCCAAGGGAAGTTTTTTCAGGCATCGTGTGTAAGCAGAATATATAAGTGCTGTTCCCTGGTGCTTCCTCGCTCACTCGAGGGCTTCGCCCTGTCGCTCAACTGCGGCGAGCACTACTGGCTGTAAaaggacagaccacatcatggttctgtgttcattaggttgttctgtccattgctgacataatccgctccacttcaacgtaacaccgcacgaagatttctattgttcctgaaggcatattcaaatcgttttcgttaccgcttgcaggcatcatgacagaacactacttcctataaacgctacacaggctcctgagattaataatgcggatctctacgataatgggagattttcccgactgtttcgttcgcttctcagtggataacagccagcttctctgtttaacagacaaaaacagcatatccactcagttccacatttccatataaaggccaaggcatttattctcaggataattgtttcagcatcgcaaccgcatcagactccggcatcgcaaactgcacccggtgccgggcagccacatccagcgcaaaaaccttcgtgtagacttccgttgaactgatggacttatgtcccatcaggctttgcagaactttcagcggtataccggcatacagcatgtgcatcgcataggaatggcggaacgtatgtggtgtgaccggaacagagaacgtcacaccgtcagcagcagcggcggcaaccgcctccccaatccaggtcctgaccgttctgtccgtcacttcccagatccgcgctttctctgtccttcctgtgcgacggttacgccgctccatgagcttatcgcgaataaatacctgtgacggaagatcacttcgcagaataaataaatcctggtgtccctgttgataccgggaagccctgggccaacttttggcgaaaatgagacgttgatcggcacgtaagaggttccaactttcaccataatgaaataagatcactaccgggcgtattttttgagttatcgagattttcaggagctaaggaagctaaaatggagaaaaaaatcactggatataccaccgttgatatatcccaatggcatcgtaaagaacattttgaggcatttcagtcagttgctcaatgtacctataaccagaccgttcagctggatattacggcctttttaaagaccgtaaagaaaaataagcacaagttttatccggcctttattcacattcttgcccgcctgatgaatgctcatccggagttccgtatggcaatgaaagacggtgagctggtgatatgggatagtgttcacccttgttacaccgttttccatgagcaaactgaaacgttttcatcgctctggagtgaataccacgacgatttccggcagtttctacacatatattcgcaagatgtggcgtgttacggtgaaaacctggcctatttccctaaagggtttattgagaatatgtttttcgtctcagccaatccctgggtgagtttcaccagttttgatttaaacgtggccaatatggacaacttcttcgcccccgttttcaccatgggcaaatattatacgcaaggcgacaaggtgctgatgccgctggcgattcaggttcatcatgccgtttgtgatggcttccatgtcggcagaatgcttaatgaattacaacagtactgcgatgagtggcagggcggggcgtaatttttttaaggcagttattggtgcccttaaacgcctggttgctacgcctgaataagtgataataagcggatgaatggcagaaattcgatgataagctgtcaaacatgagaattggtcgacggcgcgccaaagcttgcatgcctgcagccgcgtaacctggcaaaatcggttacggttgagtaataaatggatgccctgcgtaagcggggcacatttcattacctctttctccgcacccgacatagataataacttcgtatagtatacattatacgaagttatctagtagacttaattaaggatcgatccggcgcgccaatagtcatgccccgcgcccaccggaaggagctgactgggttgaaggctctcaagggcatcggtcgagcttgacattgtaggactatattgctctaataaatttgcggccgctaatacgactcactatagggagag |
| pRC1799  (Bp6- EGFP Flag) | gatccgcggaattgGTTAATAACAGGGGACGTGGTAATCCGTCCCCTTTTTATTTCTGACTGAGTTAATAACAGGCCTGCTTCGGCAGGCCTTTTTATTTCTGACTGAGTTCTTCTCAGGCCTGCTGGTAATCGCAGGCCTTTTTATTTTCTAGTCAATTCCGACGTCTAAGAAACCATTATTATCATGACATTAACCTATAAAAATAGGCGTATCACGAGGCCCTTTCGTCTTCACCTCGAGTCCCTATCAGTGATAGAGATTGACATCCCTATCAGTGATAGAGATACTGAGCACATCAGCAGGACGCACTGACCACTTTAAGtctcctATATACATATGGTGAGCAAGGGCGAGGAGCTGTTCACCGGGGTGGTGCCCATCCTGGTCGAGCTGGACGGCGACGTAAACGGCCACAAGTTCAGCGTGCGCGGCGAGGGCGAGGGCGATGCCACCAACGGCAAGCTGACCCTGAAGTTCATCTGCACCACCGGCAAGCTGCCCGTGCCCTGGCCCACCCTCGTGACCACCCTGACCTACGGCGTGCAGTGCTTCAGCCGCTACCCCGACCACATGAAGCAGCACGACTTCTTCAAGTCCGCCATGCCCGAAGGCTACGTCCAGGAGCGCACCATCTCCTTCAAGGACGACGGCACCTACAAGACCCGCGCCGAGGTGAAGTTCGAGGGCGACACCCTGGTGAACCGCATCGAGCTGAAGGGCATCGACTTCAAGGAGGACGGCAACATCCTGGGGCACAAGCTGGAGTACAACTACAACAGCCACAACGTCTATATCACCGCTGACAAGCAGAAGAACGGCATCAAGGCCAACTTCAAGATCCGCCACAACATCGAGGACGGCAGCGTGCAGCTCGCCGACCACTACCAGCAGAACACCCCCATCGGCGACGGCCCCGTGCTGCTGCCCGACAACCACTACCTGAGCACCCAGTCCGCCCTGAGCAAAGACCCCAACGAGAAGCGCGATCACATGGTCCTGCTGGAGTTCGTGACCGCCGCCGGGATCACTCTCGGCATGGACGAGCTGTACAAGTAAGGATCCgactacaaggacgacgatgacaagGAAgattataaagatgatgacgataaaAGAgactacaaggacgacgatgacaagTAAAAGCTTGTTCTTCTCAGGCCTGCTGGTAATCGCAGGCCTTTTTATTTGAATTCcgcggatccttctatagtgtcacctaaatgtcgacggccaggcggccgccaggcctacccactagtcaattcgggaggatcgaaacggcagatcgcaaaaaacagtacatacagaaggagacatgaacatgaacatcaaaaaaattgtaaaacaagccacagttctgacttttacgactgcacttctggcaggaggagcgactcaagccttcgcgaaagaaaataaccaaaaagcatacaaagaaacgtacggcgtctctcatattacacgccatgatatgctgcagatccctaaacagcagcaaaacgaaaaataccaagtgcctcaattcgatcaatcaacgattaaaaatattgagtctgcaaaaggacttgatgtgtgggacagctggccgctgcaaaacgctgacggaacagtagctgaatacaacggctatcacgttgtgtttgctcttgcgggaagcccgaaagacgctgatgacacatcaatctacatgttttatcaaaaggtcggcgacaactcaatcgacagctggaaaaacgcgggccgtgtctttaaagacagcgataagttcgacgccaacgatccgatcctgaaagatcagacgcaagaatggtccggttctgcaacctttacatctgacggaaaaatccgtttattctacactgactattccggtaaacattacggcaaacaaagcctgacaacagcgcaggtaaatgtgtcaaaatctgatgacacactcaaaatcaacggagtggaagatcacaaaacgatttttgacggagacggaaaaacatatcagaacgttcagcagtttatcgatgaaggcaattatacatccggcgacaaccatacgctgagagaccctcactacgttgaagacaaaggccataaataccttgtattcgaagccaacacgggaacagaaaacggataccaaggcgaagaatctttatttaacaaagcgtactacggcggcggcacgaacttcttccgtaaagaaagccagaagcttcagcagagcgctaaaaaacgcgatgctgagttagcgaacggcgccctcggtatcatagagttaaataatgattacacattgaaaaaagtaatgaagccgctgatcacttcaaacacggtaactgatgaaatcgagcgcgcgaatgttttcaaaatgaacggcaaatggtacttgttcactgattcacgcggttcaaaaatgacgatcgatggtattaactcaaacgatatttacatgcttggttatgtatcaaactctttaaccggcccttacaagccgctgaacaaaacagggcttgtgctgcaaatgggtcttgatccaaacgatgtgacattcacttactctcacttcgcagtgccgcaagccaaaggcaacaatgtggttatcacaagctacatgacaaacagaggcttcttcgaggataaaaaggcaacatttgcgccaagcttcttaatgaacatcaaaggcaataaaacatccgttgtcaaaaacagcatcctggagcaaggacagctgacagtcaactaataacagcaaaaagaaaatgccgatacttcattggcattttcttttatttctcaacaagatggtgaattgactagtgggtagatccacaggacgggtgtggtcgccatgatcgcgtagtcgatagtggctccaagtagcgaagcgagcaggactgggcggcggccaaagcggtcggacagtgctccgagaacgggtgcgcatagaaattgcatcaacgcatatagcgctagcagcacgccatagtgactggcgatgctgtcggaatggacgatatcccgcaagaggcccggcagtaccggcataaccaagcctatgcctacagcatccagggtgacggtgccgaggatgacgatgagcgcattgttagatttcatacacggtgcctgactgcgttagcaatttaactgtgataaactaccgcattaaagcttatcgatgataagctgtcaaacatgagaattgatccggaacccttaatataacttcgtataatgtatgctatacgaagttattaggtccctcgactatagggtcaccgtcgacagcgacacacttgcatcggatgcagcccggttaacgtgccggcacggcctgggtaaccaggtattttgtccacataaccgtgcgcaaaatgttgtggataagcaggacacagcagcaatccacagcaggcatacaaccgcacaccgaggttactccgttctacaggttacgacgacatgtcaatacttgcccttgacaggcattgatggaatcgtagtctcacgctgatagtctgatcgacaatacaagtgggaccgtggtcccagaccgataatcagaccgacaacacgagtgggatcgtggtcccagactaataatcagaccgacgatacgagtgggaccgtggtcccagactaataatcagaccgacgatacgagtgggaccgtggttccagactaataatcagaccgacgatacgagtgggaccgtggtcccagactaataatcagaccgacgatacgagtgggaccatggtcccagactaataatcagaccgacgatacgagtgggaccgtggtcccagtctgattatcagaccgacgatacgagtgggaccgtggtcccagactaataatcagaccgacgatacgagtgggaccgtggtcccagactaataatcagaccgacgatacgagtgggaccgtggtcccagtctgattatcagaccgacgatacaagtggaacagtgggcccagagagaatattcaggccagttatgctttctggcctgtaacaaaggacattaagtaaagacagataaacgtagactaaaacgtggtcgcatcagggtgctggcttttcaagttccttaagaatggcctcaattttctctatacactcagttggaacacgagacctgtccaggttaagcaccattttatcgcccttatacaatactgtcgctccaggagcaaactgatgtcgtgagcttaaactagttcttgatgcagatgacgttttaagcacagaagttaaaagagtgataacttcttcagcttcaaatatcaccccagcttttttctgctcatgaaggttagatgcctgctgcttaagtaattcctctttatctgtaaaggctttttgaagtgcatcacctgaccgggcagatagttcaccggggtgagaaaaaagagcaacaactgatttaggcaatttggcggtgttgatacagcgggtaataatcttacgtgaaatattttccgcatcagccagcgcagaaatatttccagcaaattcattctgcaatcggcttgcataacgctgaccacgttcataagcacttgttgggcgataatcgttacccaatctggataatgcagccatctgctcatcatccagctcgccaaccagaacacgataatcactttcggtaagtgcagcagctttacgacggcgactcccatcggcaatttctatgacaccagatactcttcgaccgaacgccggtgtctgttgaccagtcagtagaaaagaagggatgagatcatccagtgcgtcctcagtaagcagctcctggtcacgttcattacctgaccatacccgagaggtcttctcaacactatcaccccggagcacttcaagagtaaacttcacatcccgaccacatacaggcaaagtaatggcattaccgcgagccattactcctacgcgcgcaattaacgaatccaccatcggggcagctggtgtcgataacgaagtatcttcaaccggttgagtattgagcgtatgttttggaataacaggcgcacgcttcattatctaatctcccagcgtggtttaatcagacgatcgaaaatttcattgcagacaggttcccaaatagaaagagcatttctccaggcaccagttgaagagcgttgatcaatggcctgttcaaaaacagttctcatccggatctgacctttaccaacttcatccgtttcacgtacaacattttttagaaccatgcttccccaggcatcccgaatttgctcctccatccacggggactgagagccattactattgctgtatttggtaagcaaaatacgtacatcaggctcgaaccctttaagatcaacgttcttgagcagatcacgaagcatatcgaaaaactgcagtgcggaggtgtagtcaaacaactcagcaggcgtgggaacaatcagcacatcagcagcacatacgacattaatcgtgccgatacccaggttaggcgcgctgtcaataactatgacatcatagtcatgagcaacagtttcaatggccagtcggagcatcaggtgtggatcggtgggcagtttaccttcatcaaatttgcccattaactcagtttcaatacggtgcagagccagacaggaaggaataatgtcaagccccggccagcaagtgggctttattgcataagtgacatcgtccttttccccaagatagaaaggcaggagagtgtcttctgcatgaatatgaagatctggtacccatccgtgatacattgaggctgttccctgggggtcgttaccttccacgagcaaaacacgtagccccttcagagccagatcctgagcaagatgaacagaaactgaggttttgtaaacgccacctttatgggcagcaaccccgatcaccggtggaaatacgtcttcagcacgtcgcaatcgcgtaccaaacacatcacgcatatgattaatttgttcaattgtataaccaacacgttgctcaacccgtcctcgaatttccatatccgggtgcggtagtcgccctgctttctcggcatctctgatagcctgagaagaaaccccaactaaatccgctgcttcacctattctccagcgccgggttattttcctcgcttccgggctgtcatcattaaactgtgcaatggcgatagccttcgtcatttcatgaccagcgtttatgcactggttaagtgtttccatgagtttcattctgaacatcctttaatcattgctttgcgtttttttattaaatcttgcaatttactgcaaagcaacaacaaaatcgcaaagtcatcaaaaaaccgcaaagttgtttaaaataagagcaacactacaaaaggagataagaagagcacatacctcagtcacttattatcactagcgctcgccgcagccgtgtaaccgagcatagcgagcgaactggcgaggaagcaaagaagaactgttctgtcagatagctcttacgctcagcgcaagaagaaatatccaccgtgggaaaaactccaggtagaggtacacacgcggatagccaattcagagtaataaactgtgataatcaaccctcatcaatgatgacgaactaacccccgatatcaggtcacatgacgaagggaaagagaaggaaatcaactgtgacaaactgccctcaaatttggcttccttaaaaattacagttcaaaaagtatgagaaaatccatgcaggctgaaggaaacagcaaaactgtgacaaattaccctcagtaggtcagaacaaatgtgacgaaccaccctcaaatctgtgacagataaccctcagactatcctgtcgtcatggaagtgatatcgcggaaggaaaatacgatatgagtcgtctggcggcctttctttttctcaatgtatgagaggcgcattggagttctgctgttgatctcattaacacagacctgcaggaagcggcggcggaagtcaggcatacgctggtaactttgaggcagctggtaacgctctatgatccagtcgattttcagagagacgatgcctgagccatccggcttacgatactgacacagggattcgtataaacgcatggcatacggattggtgatttcttttgtttcactaagccgaaactgcgtaaaccggttctgtaacccgataaagaagggaatgagatatgggttgatatgtacactgtaaagccctctggatggactgtgcgcacgtttgataaaccaaggaaaagattcatagcctttttcatcgccggcatcctcttcagggcgataaaaaaccacttccttccccgcgaaactcttcaatgcctgccgtatatccttactggcttccgcagaggtcaatccgaatatttcagcatatttagcaacatggatctcgcagataccgtcatgttcctgtagggtgccatcagattttctgatctggtcaacgaacagatacagcatacgtttttgatcccgggagagactatatgccgcctcagtgaggtcgtttgactggacgattcgcgggctatttttacgtttcttgtgattgataaccgctgtttccgccatgacagatccatgtgaagtgtgacaagtttttagattgtcacactaaataaaaaagagtcaataagcagggataactttgtgaaaaaacagcttcttctgagggcaatttgtcacagggttaagggcaatttgtcacagACAGGACTGTCATTTGAGGGTGATTTGTCACACTGAAAGGGCAATTTGTCACAACACCTTCTCTAGAACCAGCATGGATAAAGGCCTACAAGGCGCTCTAAAAAAGAAGATCTAAAAACTATAAAAAAAATAATTATAAAAATATCCCCGTGGATAAGTGGATAACCCCAAGGGAAGTTTTTTCAGGCATCGTGTGTAAGCAGAATATATAAGTGCTGTTCCCTGGTGCTTCCTCGCTCACTCGAGGGCTTCGCCCTGTCGCTCAACTGCGGCGAGCACTACTGGCTGTAAaaggacagaccacatcatggttctgtgttcattaggttgttctgtccattgctgacataatccgctccacttcaacgtaacaccgcacgaagatttctattgttcctgaaggcatattcaaatcgttttcgttaccgcttgcaggcatcatgacagaacactacttcctataaacgctacacaggctcctgagattaataatgcggatctctacgataatgggagattttcccgactgtttcgttcgcttctcagtggataacagccagcttctctgtttaacagacaaaaacagcatatccactcagttccacatttccatataaaggccaaggcatttattctcaggataattgtttcagcatcgcaaccgcatcagactccggcatcgcaaactgcacccggtgccgggcagccacatccagcgcaaaaaccttcgtgtagacttccgttgaactgatggacttatgtcccatcaggctttgcagaactttcagcggtataccggcatacagcatgtgcatcgcataggaatggcggaacgtatgtggtgtgaccggaacagagaacgtcacaccgtcagcagcagcggcggcaaccgcctccccaatccaggtcctgaccgttctgtccgtcacttcccagatccgcgctttctctgtccttcctgtgcgacggttacgccgctccatgagcttatcgcgaataaatacctgtgacggaagatcacttcgcagaataaataaatcctggtgtccctgttgataccgggaagccctgggccaacttttggcgaaaatgagacgttgatcggcacgtaagaggttccaactttcaccataatgaaataagatcactaccgggcgtattttttgagttatcgagattttcaggagctaaggaagctaaaatggagaaaaaaatcactggatataccaccgttgatatatcccaatggcatcgtaaagaacattttgaggcatttcagtcagttgctcaatgtacctataaccagaccgttcagctggatattacggcctttttaaagaccgtaaagaaaaataagcacaagttttatccggcctttattcacattcttgcccgcctgatgaatgctcatccggagttccgtatggcaatgaaagacggtgagctggtgatatgggatagtgttcacccttgttacaccgttttccatgagcaaactgaaacgttttcatcgctctggagtgaataccacgacgatttccggcagtttctacacatatattcgcaagatgtggcgtgttacggtgaaaacctggcctatttccctaaagggtttattgagaatatgtttttcgtctcagccaatccctgggtgagtttcaccagttttgatttaaacgtggccaatatggacaacttcttcgcccccgttttcaccatgggcaaatattatacgcaaggcgacaaggtgctgatgccgctggcgattcaggttcatcatgccgtttgtgatggcttccatgtcggcagaatgcttaatgaattacaacagtactgcgatgagtggcagggcggggcgtaatttttttaaggcagttattggtgcccttaaacgcctggttgctacgcctgaataagtgataataagcggatgaatggcagaaattcgatgataagctgtcaaacatgagaattggtcgacggcgcgccaaagcttgcatgcctgcagccgcgtaacctggcaaaatcggttacggttgagtaataaatggatgccctgcgtaagcggggcacatttcattacctctttctccgcacccgacatagataataacttcgtatagtatacattatacgaagttatctagtagacttaattaaggatcgatccggcgcgccaatagtcatgccccgcgcccaccggaaggagctgactgggttgaaggctctcaagggcatcggtcgagcttgacattgtaggactatattgctctaataaatttgcggccgctaatacgactcactatagggagag |
| pRC1806  (Bp-EE+ EGFP Flag) | gatccgcggaattgTTAATAACAGGGGACGTGGTAATCCGTCCCCTTTTTATTTCTGACTGAGTTAATAACAGGCCTGCTTCGGCAGGCCTTTTTATTTCTGACTGAGTTCTTCTCAGGCCTGCTGGTAATCGCAGGCCTTTTTATTTTCTAGgCTGACTGACTGACTGACTGACTGACTGACTGACTGACTGACTGACTGACTGACTGACTGACTGACTGACTGACTGACTGACTGACTGACTGACTGACTGACTGACTGACTGACTGACTGACTGACTGACTGACTGACTGACTGACTGACTGACTGACTGACTGACTGACCATATGGTGAGCAAGGGCGAGGAGCTGTTCACCGGGGTGGTGCCCATCCTGGTCGAGCTGGACGGCGACGTAAACGGCCACAAGTTCAGCGTGCGCGGCGAGGGCGAGGGCGATGCCACCAACGGCAAGCTGACCCTGAAGTTCATCTGCACCACCGGCAAGCTGCCCGTGCCCTGGCCCACCCTCGTGACCACCCTGACCTACGGCGTGCAGTGCTTCAGCCGCTACCCCGACCACATGAAGCAGCACGACTTCTTCAAGTCCGCCATGCCCGAAGGCTACGTCCAGGAGCGCACCATCTCCTTCAAGGACGACGGCACCTACAAGACCCGCGCCGAGGTGAAGTTCGAGGGCGACACCCTGGTGAACCGCATCGAGCTGAAGGGCATCGACTTCAAGGAGGACGGCAACATCCTGGGGCACAAGCTGGAGTACAACTACAACAGCCACAACGTCTATATCACCGCTGACAAGCAGAAGAACGGCATCAAGGCCAACTTCAAGATCCGCCACAACATCGAGGACGGCAGCGTGCAGCTCGCCGACCACTACCAGCAGAACACCCCCATCGGCGACGGCCCCGTGCTGCTGCCCGACAACCACTACCTGAGCACCCAGTCCGCCCTGAGCAAAGACCCCAACGAGAAGCGCGATCACATGGTCCTGCTGGAGTTCGTGACCGCCGCCGGGATCACTCTCGGCATGGACGAGCTGTACAAGTAAGGATCCgactacaaggacgacgatgacaagGAAgattataaagatgatgacgataaaAGAgactacaaggacgacgatgacaagTAAAAGCTTGTTCTTCTCAGGCCTGCTGGTAATCGCAGGCCTTTTTATTTGAATTCcgcggatccttctatagtgtcacctaaatgtcgacggccaggcggccgccaggcctacccactagtcaattcgggaggatcgaaacggcagatcgcaaaaaacagtacatacagaaggagacatgaacatgaacatcaaaaaaattgtaaaacaagccacagttctgacttttacgactgcacttctggcaggaggagcgactcaagccttcgcgaaagaaaataaccaaaaagcatacaaagaaacgtacggcgtctctcatattacacgccatgatatgctgcagatccctaaacagcagcaaaacgaaaaataccaagtgcctcaattcgatcaatcaacgattaaaaatattgagtctgcaaaaggacttgatgtgtgggacagctggccgctgcaaaacgctgacggaacagtagctgaatacaacggctatcacgttgtgtttgctcttgcgggaagcccgaaagacgctgatgacacatcaatctacatgttttatcaaaaggtcggcgacaactcaatcgacagctggaaaaacgcgggccgtgtctttaaagacagcgataagttcgacgccaacgatccgatcctgaaagatcagacgcaagaatggtccggttctgcaacctttacatctgacggaaaaatccgtttattctacactgactattccggtaaacattacggcaaacaaagcctgacaacagcgcaggtaaatgtgtcaaaatctgatgacacactcaaaatcaacggagtggaagatcacaaaacgatttttgacggagacggaaaaacatatcagaacgttcagcagtttatcgatgaaggcaattatacatccggcgacaaccatacgctgagagaccctcactacgttgaagacaaaggccataaataccttgtattcgaagccaacacgggaacagaaaacggataccaaggcgaagaatctttatttaacaaagcgtactacggcggcggcacgaacttcttccgtaaagaaagccagaagcttcagcagagcgctaaaaaacgcgatgctgagttagcgaacggcgccctcggtatcatagagttaaataatgattacacattgaaaaaagtaatgaagccgctgatcacttcaaacacggtaactgatgaaatcgagcgcgcgaatgttttcaaaatgaacggcaaatggtacttgttcactgattcacgcggttcaaaaatgacgatcgatggtattaactcaaacgatatttacatgcttggttatgtatcaaactctttaaccggcccttacaagccgctgaacaaaacagggcttgtgctgcaaatgggtcttgatccaaacgatgtgacattcacttactctcacttcgcagtgccgcaagccaaaggcaacaatgtggttatcacaagctacatgacaaacagaggcttcttcgaggataaaaaggcaacatttgcgccaagcttcttaatgaacatcaaaggcaataaaacatccgttgtcaaaaacagcatcctggagcaaggacagctgacagtcaactaataacagcaaaaagaaaatgccgatacttcattggcattttcttttatttctcaacaagatggtgaattgactagtgggtagatccacaggacgggtgtggtcgccatgatcgcgtagtcgatagtggctccaagtagcgaagcgagcaggactgggcggcggccaaagcggtcggacagtgctccgagaacgggtgcgcatagaaattgcatcaacgcatatagcgctagcagcacgccatagtgactggcgatgctgtcggaatggacgatatcccgcaagaggcccggcagtaccggcataaccaagcctatgcctacagcatccagggtgacggtgccgaggatgacgatgagcgcattgttagatttcatacacggtgcctgactgcgttagcaatttaactgtgataaactaccgcattaaagcttatcgatgataagctgtcaaacatgagaattgatccggaacccttaatataacttcgtataatgtatgctatacgaagttattaggtccctcgactatagggtcaccgtcgacagcgacacacttgcatcggatgcagcccggttaacgtgccggcacggcctgggtaaccaggtattttgtccacataaccgtgcgcaaaatgttgtggataagcaggacacagcagcaatccacagcaggcatacaaccgcacaccgaggttactccgttctacaggttacgacgacatgtcaatacttgcccttgacaggcattgatggaatcgtagtctcacgctgatagtctgatcgacaatacaagtgggaccgtggtcccagaccgataatcagaccgacaacacgagtgggatcgtggtcccagactaataatcagaccgacgatacgagtgggaccgtggtcccagactaataatcagaccgacgatacgagtgggaccgtggttccagactaataatcagaccgacgatacgagtgggaccgtggtcccagactaataatcagaccgacgatacgagtgggaccatggtcccagactaataatcagaccgacgatacgagtgggaccgtggtcccagtctgattatcagaccgacgatacgagtgggaccgtggtcccagactaataatcagaccgacgatacgagtgggaccgtggtcccagactaataatcagaccgacgatacgagtgggaccgtggtcccagtctgattatcagaccgacgatacaagtggaacagtgggcccagagagaatattcaggccagttatgctttctggcctgtaacaaaggacattaagtaaagacagataaacgtagactaaaacgtggtcgcatcagggtgctggcttttcaagttccttaagaatggcctcaattttctctatacactcagttggaacacgagacctgtccaggttaagcaccattttatcgcccttatacaatactgtcgctccaggagcaaactgatgtcgtgagcttaaactagttcttgatgcagatgacgttttaagcacagaagttaaaagagtgataacttcttcagcttcaaatatcaccccagcttttttctgctcatgaaggttagatgcctgctgcttaagtaattcctctttatctgtaaaggctttttgaagtgcatcacctgaccgggcagatagttcaccggggtgagaaaaaagagcaacaactgatttaggcaatttggcggtgttgatacagcgggtaataatcttacgtgaaatattttccgcatcagccagcgcagaaatatttccagcaaattcattctgcaatcggcttgcataacgctgaccacgttcataagcacttgttgggcgataatcgttacccaatctggataatgcagccatctgctcatcatccagctcgccaaccagaacacgataatcactttcggtaagtgcagcagctttacgacggcgactcccatcggcaatttctatgacaccagatactcttcgaccgaacgccggtgtctgttgaccagtcagtagaaaagaagggatgagatcatccagtgcgtcctcagtaagcagctcctggtcacgttcattacctgaccatacccgagaggtcttctcaacactatcaccccggagcacttcaagagtaaacttcacatcccgaccacatacaggcaaagtaatggcattaccgcgagccattactcctacgcgcgcaattaacgaatccaccatcggggcagctggtgtcgataacgaagtatcttcaaccggttgagtattgagcgtatgttttggaataacaggcgcacgcttcattatctaatctcccagcgtggtttaatcagacgatcgaaaatttcattgcagacaggttcccaaatagaaagagcatttctccaggcaccagttgaagagcgttgatcaatggcctgttcaaaaacagttctcatccggatctgacctttaccaacttcatccgtttcacgtacaacattttttagaaccatgcttccccaggcatcccgaatttgctcctccatccacggggactgagagccattactattgctgtatttggtaagcaaaatacgtacatcaggctcgaaccctttaagatcaacgttcttgagcagatcacgaagcatatcgaaaaactgcagtgcggaggtgtagtcaaacaactcagcaggcgtgggaacaatcagcacatcagcagcacatacgacattaatcgtgccgatacccaggttaggcgcgctgtcaataactatgacatcatagtcatgagcaacagtttcaatggccagtcggagcatcaggtgtggatcggtgggcagtttaccttcatcaaatttgcccattaactcagtttcaatacggtgcagagccagacaggaaggaataatgtcaagccccggccagcaagtgggctttattgcataagtgacatcgtccttttccccaagatagaaaggcaggagagtgtcttctgcatgaatatgaagatctggtacccatccgtgatacattgaggctgttccctgggggtcgttaccttccacgagcaaaacacgtagccccttcagagccagatcctgagcaagatgaacagaaactgaggttttgtaaacgccacctttatgggcagcaaccccgatcaccggtggaaatacgtcttcagcacgtcgcaatcgcgtaccaaacacatcacgcatatgattaatttgttcaattgtataaccaacacgttgctcaacccgtcctcgaatttccatatccgggtgcggtagtcgccctgctttctcggcatctctgatagcctgagaagaaaccccaactaaatccgctgcttcacctattctccagcgccgggttattttcctcgcttccgggctgtcatcattaaactgtgcaatggcgatagccttcgtcatttcatgaccagcgtttatgcactggttaagtgtttccatgagtttcattctgaacatcctttaatcattgctttgcgtttttttattaaatcttgcaatttactgcaaagcaacaacaaaatcgcaaagtcatcaaaaaaccgcaaagttgtttaaaataagagcaacactacaaaaggagataagaagagcacatacctcagtcacttattatcactagcgctcgccgcagccgtgtaaccgagcatagcgagcgaactggcgaggaagcaaagaagaactgttctgtcagatagctcttacgctcagcgcaagaagaaatatccaccgtgggaaaaactccaggtagaggtacacacgcggatagccaattcagagtaataaactgtgataatcaaccctcatcaatgatgacgaactaacccccgatatcaggtcacatgacgaagggaaagagaaggaaatcaactgtgacaaactgccctcaaatttggcttccttaaaaattacagttcaaaaagtatgagaaaatccatgcaggctgaaggaaacagcaaaactgtgacaaattaccctcagtaggtcagaacaaatgtgacgaaccaccctcaaatctgtgacagataaccctcagactatcctgtcgtcatggaagtgatatcgcggaaggaaaatacgatatgagtcgtctggcggcctttctttttctcaatgtatgagaggcgcattggagttctgctgttgatctcattaacacagacctgcaggaagcggcggcggaagtcaggcatacgctggtaactttgaggcagctggtaacgctctatgatccagtcgattttcagagagacgatgcctgagccatccggcttacgatactgacacagggattcgtataaacgcatggcatacggattggtgatttcttttgtttcactaagccgaaactgcgtaaaccggttctgtaacccgataaagaagggaatgagatatgggttgatatgtacactgtaaagccctctggatggactgtgcgcacgtttgataaaccaaggaaaagattcatagcctttttcatcgccggcatcctcttcagggcgataaaaaaccacttccttccccgcgaaactcttcaatgcctgccgtatatccttactggcttccgcagaggtcaatccgaatatttcagcatatttagcaacatggatctcgcagataccgtcatgttcctgtagggtgccatcagattttctgatctggtcaacgaacagatacagcatacgtttttgatcccgggagagactatatgccgcctcagtgaggtcgtttgactggacgattcgcgggctatttttacgtttcttgtgattgataaccgctgtttccgccatgacagatccatgtgaagtgtgacaagtttttagattgtcacactaaataaaaaagagtcaataagcagggataactttgtgaaaaaacagcttcttctgagggcaatttgtcacagggttaagggcaatttgtcacagACAGGACTGTCATTTGAGGGTGATTTGTCACACTGAAAGGGCAATTTGTCACAACACCTTCTCTAGAACCAGCATGGATAAAGGCCTACAAGGCGCTCTAAAAAAGAAGATCTAAAAACTATAAAAAAAATAATTATAAAAATATCCCCGTGGATAAGTGGATAACCCCAAGGGAAGTTTTTTCAGGCATCGTGTGTAAGCAGAATATATAAGTGCTGTTCCCTGGTGCTTCCTCGCTCACTCGAGGGCTTCGCCCTGTCGCTCAACTGCGGCGAGCACTACTGGCTGTAAaaggacagaccacatcatggttctgtgttcattaggttgttctgtccattgctgacataatccgctccacttcaacgtaacaccgcacgaagatttctattgttcctgaaggcatattcaaatcgttttcgttaccgcttgcaggcatcatgacagaacactacttcctataaacgctacacaggctcctgagattaataatgcggatctctacgataatgggagattttcccgactgtttcgttcgcttctcagtggataacagccagcttctctgtttaacagacaaaaacagcatatccactcagttccacatttccatataaaggccaaggcatttattctcaggataattgtttcagcatcgcaaccgcatcagactccggcatcgcaaactgcacccggtgccgggcagccacatccagcgcaaaaaccttcgtgtagacttccgttgaactgatggacttatgtcccatcaggctttgcagaactttcagcggtataccggcatacagcatgtgcatcgcataggaatggcggaacgtatgtggtgtgaccggaacagagaacgtcacaccgtcagcagcagcggcggcaaccgcctccccaatccaggtcctgaccgttctgtccgtcacttcccagatccgcgctttctctgtccttcctgtgcgacggttacgccgctccatgagcttatcgcgaataaatacctgtgacggaagatcacttcgcagaataaataaatcctggtgtccctgttgataccgggaagccctgggccaacttttggcgaaaatgagacgttgatcggcacgtaagaggttccaactttcaccataatgaaataagatcactaccgggcgtattttttgagttatcgagattttcaggagctaaggaagctaaaatggagaaaaaaatcactggatataccaccgttgatatatcccaatggcatcgtaaagaacattttgaggcatttcagtcagttgctcaatgtacctataaccagaccgttcagctggatattacggcctttttaaagaccgtaaagaaaaataagcacaagttttatccggcctttattcacattcttgcccgcctgatgaatgctcatccggagttccgtatggcaatgaaagacggtgagctggtgatatgggatagtgttcacccttgttacaccgttttccatgagcaaactgaaacgttttcatcgctctggagtgaataccacgacgatttccggcagtttctacacatatattcgcaagatgtggcgtgttacggtgaaaacctggcctatttccctaaagggtttattgagaatatgtttttcgtctcagccaatccctgggtgagtttcaccagttttgatttaaacgtggccaatatggacaacttcttcgcccccgttttcaccatgggcaaatattatacgcaaggcgacaaggtgctgatgccgctggcgattcaggttcatcatgccgtttgtgatggcttccatgtcggcagaatgcttaatgaattacaacagtactgcgatgagtggcagggcggggcgtaatttttttaaggcagttattggtgcccttaaacgcctggttgctacgcctgaataagtgataataagcggatgaatggcagaaattcgatgataagctgtcaaacatgagaattggtcgacggcgcgccaaagcttgcatgcctgcagccgcgtaacctggcaaaatcggttacggttgagtaataaatggatgccctgcgtaagcggggcacatttcattacctctttctccgcacccgacatagataataacttcgtatagtatacattatacgaagttatctagtagacttaattaaggatcgatccggcgcgccaatagtcatgccccgcgcccaccggaaggagctgactgggttgaaggctctcaagggcatcggtcgagcttgacattgtaggactatattgctctaataaatttgcggccgctaatacgactcactatagggagag |
| pRC1723  (Bp-EE+ Hsmar1, no Flag) | gatccgcggaattgCAGCTGGTTAATAACAGGGGACGTGGTAATCCGTCCCCTTTTTATTTCTGACTGAGTTAATAACAGGCCTGCTTCGGCAGGCCTTTTTATTTCTGACTGAGTTCTTCTCAGGCCTGCTGGTAATCGCAGGCCTTTTTATTTTCTAGTCTGACTGACTGACTGACTGACTGACTGACTGACTGACTGACTGACTGACTGACTGACTGACTGACTGACTGACTGACTGACTGACTGACTGACTGACTGACTGACTGACTGACTGACTGACTGACTGACTGACTGACTGACTGACTGACTGACTGACTGACTGACTGACTgactgaCTGACCATATGGAAATGATGCTCGATAAGAAACAGATTCGTGCGATCTTTCTCTTTGAGTTTAAAATGGGTCGCAAAGCGGCGGAGACGACGCGTAATATTAACAACGCGTTCGGTCCTGGCACCGCGAACGAGCGTACCGTGCAATGGTGGTTCAAAAAGTTTCGCAAAGGCGACGAATCTCTGGAGGACGAAGAGCGTTCTGGCCGCCCGTCCGAGGTTGACAACGACCAGCTGCGTGCAATCATCGAAGCTGATCCGCTGACTACCACCCGCGAAGTTGCTGAAGAACTGAATGTGGATCACTCTACTGTGGTTCGCCACCTGAAACAGATCGGTAAAGTAAAAAAACTGGACAAATGGGTTCCTCATGAACTGTCTGAAAACCAGAAAAACCGTCGTTTCGAAGTTAGCTCCTCTCTGATTCTGCGTAACAACAACGAACCGTTCCTGGATCGTATCGTAACCTGTGATGAGAAATGGATTCTGTATGATAACCGTCGCCGCTCTGCTCAGTGGCTGGATCGCGAAGAAGCTCCAAAACACTTCCCGAAACCGAATCTGCACCAGAAGAAAGTCATGGTAACCGTATGGTGGTCTGCCGCAGGTGTTATCCACTATTCCTTCCTGAACCCGGGCGAAACTATCACCAGCGAAAAATACTGCCAGCAGATTGACGAAATGCACCGTAAACTGCAGCGTCTGCAGCCAGCACTGGTGAATCGTAAAGGTCCGATCCTGCTGCATGATAACGCCCGTCCGCACGTTGCCCAACCGACCCTGCAGAAACTGAACGAACTGGGCTATGAAGTTCTGCCACACCCGCCGTACTCCCCGGATCTGTCCCCGACTGACTACCATTTCTTCAAGCATCTGGACAACTTCCTGCAGGGTAAACGTTTTCACAACCAACAGGACGCAGAAAACGCTTTCCAGGAGTTCGTCGAAAGCCGTTCCACTGACTTCTACGCGACCGGTATCAACAAGCTGATCAGCCGTTGGCAGAAATGCGTGGACTGTAACGGCAGCTACTTCGATTAAGGATCCGTTCTTCTCAGGCCTGCTGGTAATCGCAGGCCTTTTTATTTCAGCTGcaattccgcggatccttctatagtgtcacctaaatgtcgacggccaggcggccgccaggcctacccactagtcaattcgggaggatcgaaacggcagatcgcaaaaaacagtacatacagaaggagacatgaacatgaacatcaaaaaaattgtaaaacaagccacagttctgacttttacgactgcacttctggcaggaggagcgactcaagccttcgcgaaagaaaataaccaaaaagcatacaaagaaacgtacggcgtctctcatattacacgccatgatatgctgcagatccctaaacagcagcaaaacgaaaaataccaagtgcctcaattcgatcaatcaacgattaaaaatattgagtctgcaaaaggacttgatgtgtgggacagctggccgctgcaaaacgctgacggaacagtagctgaatacaacggctatcacgttgtgtttgctcttgcgggaagcccgaaagacgctgatgacacatcaatctacatgttttatcaaaaggtcggcgacaactcaatcgacagctggaaaaacgcgggccgtgtctttaaagacagcgataagttcgacgccaacgatccgatcctgaaagatcagacgcaagaatggtccggttctgcaacctttacatctgacggaaaaatccgtttattctacactgactattccggtaaacattacggcaaacaaagcctgacaacagcgcaggtaaatgtgtcaaaatctgatgacacactcaaaatcaacggagtggaagatcacaaaacgatttttgacggagacggaaaaacatatcagaacgttcagcagtttatcgatgaaggcaattatacatccggcgacaaccatacgctgagagaccctcactacgttgaagacaaaggccataaataccttgtattcgaagccaacacgggaacagaaaacggataccaaggcgaagaatctttatttaacaaagcgtactacggcggcggcacgaacttcttccgtaaagaaagccagaagcttcagcagagcgctaaaaaacgcgatgctgagttagcgaacggcgccctcggtatcatagagttaaataatgattacacattgaaaaaagtaatgaagccgctgatcacttcaaacacggtaactgatgaaatcgagcgcgcgaatgttttcaaaatgaacggcaaatggtacttgttcactgattcacgcggttcaaaaatgacgatcgatggtattaactcaaacgatatttacatgcttggttatgtatcaaactctttaaccggcccttacaagccgctgaacaaaacagggcttgtgctgcaaatgggtcttgatccaaacgatgtgacattcacttactctcacttcgcagtgccgcaagccaaaggcaacaatgtggttatcacaagctacatgacaaacagaggcttcttcgaggataaaaaggcaacatttgcgccaagcttcttaatgaacatcaaaggcaataaaacatccgttgtcaaaaacagcatcctggagcaaggacagctgacagtcaactaataacagcaaaaagaaaatgccgatacttcattggcattttcttttatttctcaacaagatggtgaattgactagtgggtagatccacaggacgggtgtggtcgccatgatcgcgtagtcgatagtggctccaagtagcgaagcgagcaggactgggcggcggccaaagcggtcggacagtgctccgagaacgggtgcgcatagaaattgcatcaacgcatatagcgctagcagcacgccatagtgactggcgatgctgtcggaatggacgatatcccgcaagaggcccggcagtaccggcataaccaagcctatgcctacagcatccagggtgacggtgccgaggatgacgatgagcgcattgttagatttcatacacggtgcctgactgcgttagcaatttaactgtgataaactaccgcattaaagcttatcgatgataagctgtcaaacatgagaattgatccggaacccttaatataacttcgtataatgtatgctatacgaagttattaggtccctcgactatagggtcaccgtcgacagcgacacacttgcatcggatgcagcccggttaacgtgccggcacggcctgggtaaccaggtattttgtccacataaccgtgcgcaaaatgttgtggataagcaggacacagcagcaatccacagcaggcatacaaccgcacaccgaggttactccgttctacaggttacgacgacatgtcaatacttgcccttgacaggcattgatggaatcgtagtctcacgctgatagtctgatcgacaatacaagtgggaccgtggtcccagaccgataatcagaccgacaacacgagtgggatcgtggtcccagactaataatcagaccgacgatacgagtgggaccgtggtcccagactaataatcagaccgacgatacgagtgggaccgtggttccagactaataatcagaccgacgatacgagtgggaccgtggtcccagactaataatcagaccgacgatacgagtgggaccatggtcccagactaataatcagaccgacgatacgagtgggaccgtggtcccagtctgattatcagaccgacgatacgagtgggaccgtggtcccagactaataatcagaccgacgatacgagtgggaccgtggtcccagactaataatcagaccgacgatacgagtgggaccgtggtcccagtctgattatcagaccgacgatacaagtggaacagtgggcccagagagaatattcaggccagttatgctttctggcctgtaacaaaggacattaagtaaagacagataaacgtagactaaaacgtggtcgcatcagggtgctggcttttcaagttccttaagaatggcctcaattttctctatacactcagttggaacacgagacctgtccaggttaagcaccattttatcgcccttatacaatactgtcgctccaggagcaaactgatgtcgtgagcttaaactagttcttgatgcagatgacgttttaagcacagaagttaaaagagtgataacttcttcagcttcaaatatcaccccagcttttttctgctcatgaaggttagatgcctgctgcttaagtaattcctctttatctgtaaaggctttttgaagtgcatcacctgaccgggcagatagttcaccggggtgagaaaaaagagcaacaactgatttaggcaatttggcggtgttgatacagcgggtaataatcttacgtgaaatattttccgcatcagccagcgcagaaatatttccagcaaattcattctgcaatcggcttgcataacgctgaccacgttcataagcacttgttgggcgataatcgttacccaatctggataatgcagccatctgctcatcatccagctcgccaaccagaacacgataatcactttcggtaagtgcagcagctttacgacggcgactcccatcggcaatttctatgacaccagatactcttcgaccgaacgccggtgtctgttgaccagtcagtagaaaagaagggatgagatcatccagtgcgtcctcagtaagcagctcctggtcacgttcattacctgaccatacccgagaggtcttctcaacactatcaccccggagcacttcaagagtaaacttcacatcccgaccacatacaggcaaagtaatggcattaccgcgagccattactcctacgcgcgcaattaacgaatccaccatcggggcagctggtgtcgataacgaagtatcttcaaccggttgagtattgagcgtatgttttggaataacaggcgcacgcttcattatctaatctcccagcgtggtttaatcagacgatcgaaaatttcattgcagacaggttcccaaatagaaagagcatttctccaggcaccagttgaagagcgttgatcaatggcctgttcaaaaacagttctcatccggatctgacctttaccaacttcatccgtttcacgtacaacattttttagaaccatgcttccccaggcatcccgaatttgctcctccatccacggggactgagagccattactattgctgtatttggtaagcaaaatacgtacatcaggctcgaaccctttaagatcaacgttcttgagcagatcacgaagcatatcgaaaaactgcagtgcggaggtgtagtcaaacaactcagcaggcgtgggaacaatcagcacatcagcagcacatacgacattaatcgtgccgatacccaggttaggcgcgctgtcaataactatgacatcatagtcatgagcaacagtttcaatggccagtcggagcatcaggtgtggatcggtgggcagtttaccttcatcaaatttgcccattaactcagtttcaatacggtgcagagccagacaggaaggaataatgtcaagccccggccagcaagtgggctttattgcataagtgacatcgtccttttccccaagatagaaaggcaggagagtgtcttctgcatgaatatgaagatctggtacccatccgtgatacattgaggctgttccctgggggtcgttaccttccacgagcaaaacacgtagccccttcagagccagatcctgagcaagatgaacagaaactgaggttttgtaaacgccacctttatgggcagcaaccccgatcaccggtggaaatacgtcttcagcacgtcgcaatcgcgtaccaaacacatcacgcatatgattaatttgttcaattgtataaccaacacgttgctcaacccgtcctcgaatttccatatccgggtgcggtagtcgccctgctttctcggcatctctgatagcctgagaagaaaccccaactaaatccgctgcttcacctattctccagcgccgggttattttcctcgcttccgggctgtcatcattaaactgtgcaatggcgatagccttcgtcatttcatgaccagcgtttatgcactggttaagtgtttccatgagtttcattctgaacatcctttaatcattgctttgcgtttttttattaaatcttgcaatttactgcaaagcaacaacaaaatcgcaaagtcatcaaaaaaccgcaaagttgtttaaaataagagcaacactacaaaaggagataagaagagcacatacctcagtcacttattatcactagcgctcgccgcagccgtgtaaccgagcatagcgagcgaactggcgaggaagcaaagaagaactgttctgtcagatagctcttacgctcagcgcaagaagaaatatccaccgtgggaaaaactccaggtagaggtacacacgcggatagccaattcagagtaataaactgtgataatcaaccctcatcaatgatgacgaactaacccccgatatcaggtcacatgacgaagggaaagagaaggaaatcaactgtgacaaactgccctcaaatttggcttccttaaaaattacagttcaaaaagtatgagaaaatccatgcaggctgaaggaaacagcaaaactgtgacaaattaccctcagtaggtcagaacaaatgtgacgaaccaccctcaaatctgtgacagataaccctcagactatcctgtcgtcatggaagtgatatcgcggaaggaaaatacgatatgagtcgtctggcggcctttctttttctcaatgtatgagaggcgcattggagttctgctgttgatctcattaacacagacctgcaggaagcggcggcggaagtcaggcatacgctggtaactttgaggcagctggtaacgctctatgatccagtcgattttcagagagacgatgcctgagccatccggcttacgatactgacacagggattcgtataaacgcatggcatacggattggtgatttcttttgtttcactaagccgaaactgcgtaaaccggttctgtaacccgataaagaagggaatgagatatgggttgatatgtacactgtaaagccctctggatggactgtgcgcacgtttgataaaccaaggaaaagattcatagcctttttcatcgccggcatcctcttcagggcgataaaaaaccacttccttccccgcgaaactcttcaatgcctgccgtatatccttactggcttccgcagaggtcaatccgaatatttcagcatatttagcaacatggatctcgcagataccgtcatgttcctgtagggtgccatcagattttctgatctggtcaacgaacagatacagcatacgtttttgatcccgggagagactatatgccgcctcagtgaggtcgtttgactggacgattcgcgggctatttttacgtttcttgtgattgataaccgctgtttccgccatgacagatccatgtgaagtgtgacaagtttttagattgtcacactaaataaaaaagagtcaataagcagggataactttgtgaaaaaacagcttcttctgagggcaatttgtcacagggttaagggcaatttgtcacagACAGGACTGTCATTTGAGGGTGATTTGTCACACTGAAAGGGCAATTTGTCACAACACCTTCTCTAGAACCAGCATGGATAAAGGCCTACAAGGCGCTCTAAAAAAGAAGATCTAAAAACTATAAAAAAAATAATTATAAAAATATCCCCGTGGATAAGTGGATAACCCCAAGGGAAGTTTTTTCAGGCATCGTGTGTAAGCAGAATATATAAGTGCTGTTCCCTGGTGCTTCCTCGCTCACTCGAGGGCTTCGCCCTGTCGCTCAACTGCGGCGAGCACTACTGGCTGTAAaaggacagaccacatcatggttctgtgttcattaggttgttctgtccattgctgacataatccgctccacttcaacgtaacaccgcacgaagatttctattgttcctgaaggcatattcaaatcgttttcgttaccgcttgcaggcatcatgacagaacactacttcctataaacgctacacaggctcctgagattaataatgcggatctctacgataatgggagattttcccgactgtttcgttcgcttctcagtggataacagccagcttctctgtttaacagacaaaaacagcatatccactcagttccacatttccatataaaggccaaggcatttattctcaggataattgtttcagcatcgcaaccgcatcagactccggcatcgcaaactgcacccggtgccgggcagccacatccagcgcaaaaaccttcgtgtagacttccgttgaactgatggacttatgtcccatcaggctttgcagaactttcagcggtataccggcatacagcatgtgcatcgcataggaatggcggaacgtatgtggtgtgaccggaacagagaacgtcacaccgtcagcagcagcggcggcaaccgcctccccaatccaggtcctgaccgttctgtccgtcacttcccagatccgcgctttctctgtccttcctgtgcgacggttacgccgctccatgagcttatcgcgaataaatacctgtgacggaagatcacttcgcagaataaataaatcctggtgtccctgttgataccgggaagccctgggccaacttttggcgaaaatgagacgttgatcggcacgtaagaggttccaactttcaccataatgaaataagatcactaccgggcgtattttttgagttatcgagattttcaggagctaaggaagctaaaatggagaaaaaaatcactggatataccaccgttgatatatcccaatggcatcgtaaagaacattttgaggcatttcagtcagttgctcaatgtacctataaccagaccgttcagctggatattacggcctttttaaagaccgtaaagaaaaataagcacaagttttatccggcctttattcacattcttgcccgcctgatgaatgctcatccggagttccgtatggcaatgaaagacggtgagctggtgatatgggatagtgttcacccttgttacaccgttttccatgagcaaactgaaacgttttcatcgctctggagtgaataccacgacgatttccggcagtttctacacatatattcgcaagatgtggcgtgttacggtgaaaacctggcctatttccctaaagggtttattgagaatatgtttttcgtctcagccaatccctgggtgagtttcaccagttttgatttaaacgtggccaatatggacaacttcttcgcccccgttttcaccatgggcaaatattatacgcaaggcgacaaggtgctgatgccgctggcgattcaggttcatcatgccgtttgtgatggcttccatgtcggcagaatgcttaatgaattacaacagtactgcgatgagtggcagggcggggcgtaatttttttaaggcagttattggtgcccttaaacgcctggttgctacgcctgaataagtgataataagcggatgaatggcagaaattcgatgataagctgtcaaacatgagaattggtcgacggcgcgccaaagcttgcatgcctgcagccgcgtaacctggcaaaatcggttacggttgagtaataaatggatgccctgcgtaagcggggcacatttcattacctctttctccgcacccgacatagataataacttcgtatagtatacattatacgaagttatctagtagacttaattaaggatcgatccggcgcgccaatagtcatgccccgcgcccaccggaaggagctgactgggttgaaggctctcaagggcatcggtcgagcttgacattgtaggactatattgctctaataaatttgcggccgctaatacgactcactatagggagag |
| pRC1724  (Bp2++ Hsmar1, no Flag) | gatccgcggaattgCAGCTGGTTAATAACAGGGGACGTGGTAATCCGTCCCCTTTTTATTTCTGACTGAGTTAATAACAGGCCTGCTTCGGCAGGCCTTTTTATTTCTGACTGAGTTCTTCTCAGGCCTGCTGGTAATCGCAGGCCTTTTTATTTTCTAGACAATTCCGACGTCTAAGGAAACCATTATCATGACATCAACCTATAAAAATAGGCGTATCACGAGGCCCTCTCGTCTCCACCTCAAGCTCCCTATCTAGTGATAGCGATTGACATCCCTATCAGTGACGGAGATATTGAGCACATCAGCAGGACGCACTGACCACTTTAAGaaggagATATACATATGGAAATGATGCTCGATAAGAAACAGATTCGTGCGATCTTTCTCTTTGAGTTTAAAATGGGTCGCAAAGCGGCGGAGACGACGCGTAATATTAACAACGCGTTCGGTCCTGGCACCGCGAACGAGCGTACCGTGCAATGGTGGTTCAAAAAGTTTCGCAAAGGCGACGAATCTCTGGAGGACGAAGAGCGTTCTGGCCGCCCGTCCGAGGTTGACAACGACCAGCTGCGTGCAATCATCGAAGCTGATCCGCTGACTACCACCCGCGAAGTTGCTGAAGAACTGAATGTGGATCACTCTACTGTGGTTCGCCACCTGAAACAGATCGGTAAAGTAAAAAAACTGGACAAATGGGTTCCTCATGAACTGTCTGAAAACCAGAAAAACCGTCGTTTCGAAGTTAGCTCCTCTCTGATTCTGCGTAACAACAACGAACCGTTCCTGGATCGTATCGTAACCTGTGATGAGAAATGGATTCTGTATGATAACCGTCGCCGCTCTGCTCAGTGGCTGGATCGCGAAGAAGCTCCAAAACACTTCCCGAAACCGAATCTGCACCAGAAGAAAGTCATGGTAACCGTATGGTGGTCTGCCGCAGGTGTTATCCACTATTCCTTCCTGAACCCGGGCGAAACTATCACCAGCGAAAAATACTGCCAGCAGATTGACGAAATGCACCGTAAACTGCAGCGTCTGCAGCCAGCACTGGTGAATCGTAAAGGTCCGATCCTGCTGCATGATAACGCCCGTCCGCACGTTGCCCAACCGACCCTGCAGAAACTGAACGAACTGGGCTATGAAGTTCTGCCACACCCGCCGTACTCCCCGGATCTGTCCCCGACTGACTACCATTTCTTCAAGCATCTGGACAACTTCCTGCAGGGTAAACGTTTTCACAACCAACAGGACGCAGAAAACGCTTTCCAGGAGTTCGTCGAAAGCCGTTCCACTGACTTCTACGCGACCGGTATCAACAAGCTGATCAGCCGTTGGCAGAAATGCGTGGACTGTAACGGCAGCTACTTCGATTAAGGATCCGTTCTTCTCAGGCCTGCTGGTAATCGCAGGCCTTTTTATTTCAGCTGcaattccgcggatccttctatagtgtcacctaaatgtcgacggccaggcggccgccaggcctacccactagtcaattcgggaggatcgaaacggcagatcgcaaaaaacagtacatacagaaggagacatgaacatgaacatcaaaaaaattgtaaaacaagccacagttctgacttttacgactgcacttctggcaggaggagcgactcaagccttcgcgaaagaaaataaccaaaaagcatacaaagaaacgtacggcgtctctcatattacacgccatgatatgctgcagatccctaaacagcagcaaaacgaaaaataccaagtgcctcaattcgatcaatcaacgattaaaaatattgagtctgcaaaaggacttgatgtgtgggacagctggccgctgcaaaacgctgacggaacagtagctgaatacaacggctatcacgttgtgtttgctcttgcgggaagcccgaaagacgctgatgacacatcaatctacatgttttatcaaaaggtcggcgacaactcaatcgacagctggaaaaacgcgggccgtgtctttaaagacagcgataagttcgacgccaacgatccgatcctgaaagatcagacgcaagaatggtccggttctgcaacctttacatctgacggaaaaatccgtttattctacactgactattccggtaaacattacggcaaacaaagcctgacaacagcgcaggtaaatgtgtcaaaatctgatgacacactcaaaatcaacggagtggaagatcacaaaacgatttttgacggagacggaaaaacatatcagaacgttcagcagtttatcgatgaaggcaattatacatccggcgacaaccatacgctgagagaccctcactacgttgaagacaaaggccataaataccttgtattcgaagccaacacgggaacagaaaacggataccaaggcgaagaatctttatttaacaaagcgtactacggcggcggcacgaacttcttccgtaaagaaagccagaagcttcagcagagcgctaaaaaacgcgatgctgagttagcgaacggcgccctcggtatcatagagttaaataatgattacacattgaaaaaagtaatgaagccgctgatcacttcaaacacggtaactgatgaaatcgagcgcgcgaatgttttcaaaatgaacggcaaatggtacttgttcactgattcacgcggttcaaaaatgacgatcgatggtattaactcaaacgatatttacatgcttggttatgtatcaaactctttaaccggcccttacaagccgctgaacaaaacagggcttgtgctgcaaatgggtcttgatccaaacgatgtgacattcacttactctcacttcgcagtgccgcaagccaaaggcaacaatgtggttatcacaagctacatgacaaacagaggcttcttcgaggataaaaaggcaacatttgcgccaagcttcttaatgaacatcaaaggcaataaaacatccgttgtcaaaaacagcatcctggagcaaggacagctgacagtcaactaataacagcaaaaagaaaatgccgatacttcattggcattttcttttatttctcaacaagatggtgaattgactagtgggtagatccacaggacgggtgtggtcgccatgatcgcgtagtcgatagtggctccaagtagcgaagcgagcaggactgggcggcggccaaagcggtcggacagtgctccgagaacgggtgcgcatagaaattgcatcaacgcatatagcgctagcagcacgccatagtgactggcgatgctgtcggaatggacgatatcccgcaagaggcccggcagtaccggcataaccaagcctatgcctacagcatccagggtgacggtgccgaggatgacgatgagcgcattgttagatttcatacacggtgcctgactgcgttagcaatttaactgtgataaactaccgcattaaagcttatcgatgataagctgtcaaacatgagaattgatccggaacccttaatataacttcgtataatgtatgctatacgaagttattaggtccctcgactatagggtcaccgtcgacagcgacacacttgcatcggatgcagcccggttaacgtgccggcacggcctgggtaaccaggtattttgtccacataaccgtgcgcaaaatgttgtggataagcaggacacagcagcaatccacagcaggcatacaaccgcacaccgaggttactccgttctacaggttacgacgacatgtcaatacttgcccttgacaggcattgatggaatcgtagtctcacgctgatagtctgatcgacaatacaagtgggaccgtggtcccagaccgataatcagaccgacaacacgagtgggatcgtggtcccagactaataatcagaccgacgatacgagtgggaccgtggtcccagactaataatcagaccgacgatacgagtgggaccgtggttccagactaataatcagaccgacgatacgagtgggaccgtggtcccagactaataatcagaccgacgatacgagtgggaccatggtcccagactaataatcagaccgacgatacgagtgggaccgtggtcccagtctgattatcagaccgacgatacgagtgggaccgtggtcccagactaataatcagaccgacgatacgagtgggaccgtggtcccagactaataatcagaccgacgatacgagtgggaccgtggtcccagtctgattatcagaccgacgatacaagtggaacagtgggcccagagagaatattcaggccagttatgctttctggcctgtaacaaaggacattaagtaaagacagataaacgtagactaaaacgtggtcgcatcagggtgctggcttttcaagttccttaagaatggcctcaattttctctatacactcagttggaacacgagacctgtccaggttaagcaccattttatcgcccttatacaatactgtcgctccaggagcaaactgatgtcgtgagcttaaactagttcttgatgcagatgacgttttaagcacagaagttaaaagagtgataacttcttcagcttcaaatatcaccccagcttttttctgctcatgaaggttagatgcctgctgcttaagtaattcctctttatctgtaaaggctttttgaagtgcatcacctgaccgggcagatagttcaccggggtgagaaaaaagagcaacaactgatttaggcaatttggcggtgttgatacagcgggtaataatcttacgtgaaatattttccgcatcagccagcgcagaaatatttccagcaaattcattctgcaatcggcttgcataacgctgaccacgttcataagcacttgttgggcgataatcgttacccaatctggataatgcagccatctgctcatcatccagctcgccaaccagaacacgataatcactttcggtaagtgcagcagctttacgacggcgactcccatcggcaatttctatgacaccagatactcttcgaccgaacgccggtgtctgttgaccagtcagtagaaaagaagggatgagatcatccagtgcgtcctcagtaagcagctcctggtcacgttcattacctgaccatacccgagaggtcttctcaacactatcaccccggagcacttcaagagtaaacttcacatcccgaccacatacaggcaaagtaatggcattaccgcgagccattactcctacgcgcgcaattaacgaatccaccatcggggcagctggtgtcgataacgaagtatcttcaaccggttgagtattgagcgtatgttttggaataacaggcgcacgcttcattatctaatctcccagcgtggtttaatcagacgatcgaaaatttcattgcagacaggttcccaaatagaaagagcatttctccaggcaccagttgaagagcgttgatcaatggcctgttcaaaaacagttctcatccggatctgacctttaccaacttcatccgtttcacgtacaacattttttagaaccatgcttccccaggcatcccgaatttgctcctccatccacggggactgagagccattactattgctgtatttggtaagcaaaatacgtacatcaggctcgaaccctttaagatcaacgttcttgagcagatcacgaagcatatcgaaaaactgcagtgcggaggtgtagtcaaacaactcagcaggcgtgggaacaatcagcacatcagcagcacatacgacattaatcgtgccgatacccaggttaggcgcgctgtcaataactatgacatcatagtcatgagcaacagtttcaatggccagtcggagcatcaggtgtggatcggtgggcagtttaccttcatcaaatttgcccattaactcagtttcaatacggtgcagagccagacaggaaggaataatgtcaagccccggccagcaagtgggctttattgcataagtgacatcgtccttttccccaagatagaaaggcaggagagtgtcttctgcatgaatatgaagatctggtacccatccgtgatacattgaggctgttccctgggggtcgttaccttccacgagcaaaacacgtagccccttcagagccagatcctgagcaagatgaacagaaactgaggttttgtaaacgccacctttatgggcagcaaccccgatcaccggtggaaatacgtcttcagcacgtcgcaatcgcgtaccaaacacatcacgcatatgattaatttgttcaattgtataaccaacacgttgctcaacccgtcctcgaatttccatatccgggtgcggtagtcgccctgctttctcggcatctctgatagcctgagaagaaaccccaactaaatccgctgcttcacctattctccagcgccgggttattttcctcgcttccgggctgtcatcattaaactgtgcaatggcgatagccttcgtcatttcatgaccagcgtttatgcactggttaagtgtttccatgagtttcattctgaacatcctttaatcattgctttgcgtttttttattaaatcttgcaatttactgcaaagcaacaacaaaatcgcaaagtcatcaaaaaaccgcaaagttgtttaaaataagagcaacactacaaaaggagataagaagagcacatacctcagtcacttattatcactagcgctcgccgcagccgtgtaaccgagcatagcgagcgaactggcgaggaagcaaagaagaactgttctgtcagatagctcttacgctcagcgcaagaagaaatatccaccgtgggaaaaactccaggtagaggtacacacgcggatagccaattcagagtaataaactgtgataatcaaccctcatcaatgatgacgaactaacccccgatatcaggtcacatgacgaagggaaagagaaggaaatcaactgtgacaaactgccctcaaatttggcttccttaaaaattacagttcaaaaagtatgagaaaatccatgcaggctgaaggaaacagcaaaactgtgacaaattaccctcagtaggtcagaacaaatgtgacgaaccaccctcaaatctgtgacagataaccctcagactatcctgtcgtcatggaagtgatatcgcggaaggaaaatacgatatgagtcgtctggcggcctttctttttctcaatgtatgagaggcgcattggagttctgctgttgatctcattaacacagacctgcaggaagcggcggcggaagtcaggcatacgctggtaactttgaggcagctggtaacgctctatgatccagtcgattttcagagagacgatgcctgagccatccggcttacgatactgacacagggattcgtataaacgcatggcatacggattggtgatttcttttgtttcactaagccgaaactgcgtaaaccggttctgtaacccgataaagaagggaatgagatatgggttgatatgtacactgtaaagccctctggatggactgtgcgcacgtttgataaaccaaggaaaagattcatagcctttttcatcgccggcatcctcttcagggcgataaaaaaccacttccttccccgcgaaactcttcaatgcctgccgtatatccttactggcttccgcagaggtcaatccgaatatttcagcatatttagcaacatggatctcgcagataccgtcatgttcctgtagggtgccatcagattttctgatctggtcaacgaacagatacagcatacgtttttgatcccgggagagactatatgccgcctcagtgaggtcgtttgactggacgattcgcgggctatttttacgtttcttgtgattgataaccgctgtttccgccatgacagatccatgtgaagtgtgacaagtttttagattgtcacactaaataaaaaagagtcaataagcagggataactttgtgaaaaaacagcttcttctgagggcaatttgtcacagggttaagggcaatttgtcacagACAGGACTGTCATTTGAGGGTGATTTGTCACACTGAAAGGGCAATTTGTCACAACACCTTCTCTAGAACCAGCATGGATAAAGGCCTACAAGGCGCTCTAAAAAAGAAGATCTAAAAACTATAAAAAAAATAATTATAAAAATATCCCCGTGGATAAGTGGATAACCCCAAGGGAAGTTTTTTCAGGCATCGTGTGTAAGCAGAATATATAAGTGCTGTTCCCTGGTGCTTCCTCGCTCACTCGAGGGCTTCGCCCTGTCGCTCAACTGCGGCGAGCACTACTGGCTGTAAaaggacagaccacatcatggttctgtgttcattaggttgttctgtccattgctgacataatccgctccacttcaacgtaacaccgcacgaagatttctattgttcctgaaggcatattcaaatcgttttcgttaccgcttgcaggcatcatgacagaacactacttcctataaacgctacacaggctcctgagattaataatgcggatctctacgataatgggagattttcccgactgtttcgttcgcttctcagtggataacagccagcttctctgtttaacagacaaaaacagcatatccactcagttccacatttccatataaaggccaaggcatttattctcaggataattgtttcagcatcgcaaccgcatcagactccggcatcgcaaactgcacccggtgccgggcagccacatccagcgcaaaaaccttcgtgtagacttccgttgaactgatggacttatgtcccatcaggctttgcagaactttcagcggtataccggcatacagcatgtgcatcgcataggaatggcggaacgtatgtggtgtgaccggaacagagaacgtcacaccgtcagcagcagcggcggcaaccgcctccccaatccaggtcctgaccgttctgtccgtcacttcccagatccgcgctttctctgtccttcctgtgcgacggttacgccgctccatgagcttatcgcgaataaatacctgtgacggaagatcacttcgcagaataaataaatcctggtgtccctgttgataccgggaagccctgggccaacttttggcgaaaatgagacgttgatcggcacgtaagaggttccaactttcaccataatgaaataagatcactaccgggcgtattttttgagttatcgagattttcaggagctaaggaagctaaaatggagaaaaaaatcactggatataccaccgttgatatatcccaatggcatcgtaaagaacattttgaggcatttcagtcagttgctcaatgtacctataaccagaccgttcagctggatattacggcctttttaaagaccgtaaagaaaaataagcacaagttttatccggcctttattcacattcttgcccgcctgatgaatgctcatccggagttccgtatggcaatgaaagacggtgagctggtgatatgggatagtgttcacccttgttacaccgttttccatgagcaaactgaaacgttttcatcgctctggagtgaataccacgacgatttccggcagtttctacacatatattcgcaagatgtggcgtgttacggtgaaaacctggcctatttccctaaagggtttattgagaatatgtttttcgtctcagccaatccctgggtgagtttcaccagttttgatttaaacgtggccaatatggacaacttcttcgcccccgttttcaccatgggcaaatattatacgcaaggcgacaaggtgctgatgccgctggcgattcaggttcatcatgccgtttgtgatggcttccatgtcggcagaatgcttaatgaattacaacagtactgcgatgagtggcagggcggggcgtaatttttttaaggcagttattggtgcccttaaacgcctggttgctacgcctgaataagtgataataagcggatgaatggcagaaattcgatgataagctgtcaaacatgagaattggtcgacggcgcgccaaagcttgcatgcctgcagccgcgtaacctggcaaaatcggttacggttgagtaataaatggatgccctgcgtaagcggggcacatttcattacctctttctccgcacccgacatagataataacttcgtatagtatacattatacgaagttatctagtagacttaattaaggatcgatccggcgcgccaatagtcatgccccgcgcccaccggaaggagctgactgggttgaaggctctcaagggcatcggtcgagcttgacattgtaggactatattgctctaataaatttgcggccgctaatacgactcactatagggagag |
| pRC1725  (Bp3++ Hsmar1, no Flag) | gatccgcggaattgCAGCTGGTTAATAACAGGGGACGTGGTAATCCGTCCCCTTTTTATTTCTGACTGAGTTAATAACAGGCCTGCTTCGGCAGGCCTTTTTATTTCTGACTGAGTTCTTCTCAGGCCTGCTGGTAATCGCAGGCCTTTTTATTTTCTAGACAATTCCGACGTCTAAGAAACCATTATTATCATGACATTAACCTATAAAAATAGGCGTATCACGAGGCCCTTTCGTCTTCACCTCGAGTCCCTATCAGTGATAGAGATTGACCTCCCTATCAGTGATAGAGATACTGAGCACATCAGCAGGACGCACTGACCACTTTAAGAAGGAGATATACATATGGAAATGATGCTCGATAAGAAACAGATTCGTGCGATCTTTCTCTTTGAGTTTAAAATGGGTCGCAAAGCGGCGGAGACGACGCGTAATATTAACAACGCGTTCGGTCCTGGCACCGCGAACGAGCGTACCGTGCAATGGTGGTTCAAAAAGTTTCGCAAAGGCGACGAATCTCTGGAGGACGAAGAGCGTTCTGGCCGCCCGTCCGAGGTTGACAACGACCAGCTGCGTGCAATCATCGAAGCTGATCCGCTGACTACCACCCGCGAAGTTGCTGAAGAACTGAATGTGGATCACTCTACTGTGGTTCGCCACCTGAAACAGATCGGTAAAGTAAAAAAACTGGACAAATGGGTTCCTCATGAACTGTCTGAAAACCAGAAAAACCGTCGTTTCGAAGTTAGCTCCTCTCTGATTCTGCGTAACAACAACGAACCGTTCCTGGATCGTATCGTAACCTGTGATGAGAAATGGATTCTGTATGATAACCGTCGCCGCTCTGCTCAGTGGCTGGATCGCGAAGAAGCTCCAAAACACTTCCCGAAACCGAATCTGCACCAGAAGAAAGTCATGGTAACCGTATGGTGGTCTGCCGCAGGTGTTATCCACTATTCCTTCCTGAACCCGGGCGAAACTATCACCAGCGAAAAATACTGCCAGCAGATTGACGAAATGCACCGTAAACTGCAGCGTCTGCAGCCAGCACTGGTGAATCGTAAAGGTCCGATCCTGCTGCATGATAACGCCCGTCCGCACGTTGCCCAACCGACCCTGCAGAAACTGAACGAACTGGGCTATGAAGTTCTGCCACACCCGCCGTACTCCCCGGATCTGTCCCCGACTGACTACCATTTCTTCAAGCATCTGGACAACTTCCTGCAGGGTAAACGTTTTCACAACCAACAGGACGCAGAAAACGCTTTCCAGGAGTTCGTCGAAAGCCGTTCCACTGACTTCTACGCGACCGGTATCAACAAGCTGATCAGCCGTTGGCAGAAATGCGTGGACTGTAACGGCAGCTACTTCGATTAAGGATCCGTTCTTCTCAGGCCTGCTGGTAATCGCAGGCCTTTTTATTTCAGCTGcaattccgcggatccttctatagtgtcacctaaatgtcgacggccaggcggccgccaggcctacccactagtcaattcgggaggatcgaaacggcagatcgcaaaaaacagtacatacagaaggagacatgaacatgaacatcaaaaaaattgtaaaacaagccacagttctgacttttacgactgcacttctggcaggaggagcgactcaagccttcgcgaaagaaaataaccaaaaagcatacaaagaaacgtacggcgtctctcatattacacgccatgatatgctgcagatccctaaacagcagcaaaacgaaaaataccaagtgcctcaattcgatcaatcaacgattaaaaatattgagtctgcaaaaggacttgatgtgtgggacagctggccgctgcaaaacgctgacggaacagtagctgaatacaacggctatcacgttgtgtttgctcttgcgggaagcccgaaagacgctgatgacacatcaatctacatgttttatcaaaaggtcggcgacaactcaatcgacagctggaaaaacgcgggccgtgtctttaaagacagcgataagttcgacgccaacgatccgatcctgaaagatcagacgcaagaatggtccggttctgcaacctttacatctgacggaaaaatccgtttattctacactgactattccggtaaacattacggcaaacaaagcctgacaacagcgcaggtaaatgtgtcaaaatctgatgacacactcaaaatcaacggagtggaagatcacaaaacgatttttgacggagacggaaaaacatatcagaacgttcagcagtttatcgatgaaggcaattatacatccggcgacaaccatacgctgagagaccctcactacgttgaagacaaaggccataaataccttgtattcgaagccaacacgggaacagaaaacggataccaaggcgaagaatctttatttaacaaagcgtactacggcggcggcacgaacttcttccgtaaagaaagccagaagcttcagcagagcgctaaaaaacgcgatgctgagttagcgaacggcgccctcggtatcatagagttaaataatgattacacattgaaaaaagtaatgaagccgctgatcacttcaaacacggtaactgatgaaatcgagcgcgcgaatgttttcaaaatgaacggcaaatggtacttgttcactgattcacgcggttcaaaaatgacgatcgatggtattaactcaaacgatatttacatgcttggttatgtatcaaactctttaaccggcccttacaagccgctgaacaaaacagggcttgtgctgcaaatgggtcttgatccaaacgatgtgacattcacttactctcacttcgcagtgccgcaagccaaaggcaacaatgtggttatcacaagctacatgacaaacagaggcttcttcgaggataaaaaggcaacatttgcgccaagcttcttaatgaacatcaaaggcaataaaacatccgttgtcaaaaacagcatcctggagcaaggacagctgacagtcaactaataacagcaaaaagaaaatgccgatacttcattggcattttcttttatttctcaacaagatggtgaattgactagtgggtagatccacaggacgggtgtggtcgccatgatcgcgtagtcgatagtggctccaagtagcgaagcgagcaggactgggcggcggccaaagcggtcggacagtgctccgagaacgggtgcgcatagaaattgcatcaacgcatatagcgctagcagcacgccatagtgactggcgatgctgtcggaatggacgatatcccgcaagaggcccggcagtaccggcataaccaagcctatgcctacagcatccagggtgacggtgccgaggatgacgatgagcgcattgttagatttcatacacggtgcctgactgcgttagcaatttaactgtgataaactaccgcattaaagcttatcgatgataagctgtcaaacatgagaattgatccggaacccttaatataacttcgtataatgtatgctatacgaagttattaggtccctcgactatagggtcaccgtcgacagcgacacacttgcatcggatgcagcccggttaacgtgccggcacggcctgggtaaccaggtattttgtccacataaccgtgcgcaaaatgttgtggataagcaggacacagcagcaatccacagcaggcatacaaccgcacaccgaggttactccgttctacaggttacgacgacatgtcaatacttgcccttgacaggcattgatggaatcgtagtctcacgctgatagtctgatcgacaatacaagtgggaccgtggtcccagaccgataatcagaccgacaacacgagtgggatcgtggtcccagactaataatcagaccgacgatacgagtgggaccgtggtcccagactaataatcagaccgacgatacgagtgggaccgtggttccagactaataatcagaccgacgatacgagtgggaccgtggtcccagactaataatcagaccgacgatacgagtgggaccatggtcccagactaataatcagaccgacgatacgagtgggaccgtggtcccagtctgattatcagaccgacgatacgagtgggaccgtggtcccagactaataatcagaccgacgatacgagtgggaccgtggtcccagactaataatcagaccgacgatacgagtgggaccgtggtcccagtctgattatcagaccgacgatacaagtggaacagtgggcccagagagaatattcaggccagttatgctttctggcctgtaacaaaggacattaagtaaagacagataaacgtagactaaaacgtggtcgcatcagggtgctggcttttcaagttccttaagaatggcctcaattttctctatacactcagttggaacacgagacctgtccaggttaagcaccattttatcgcccttatacaatactgtcgctccaggagcaaactgatgtcgtgagcttaaactagttcttgatgcagatgacgttttaagcacagaagttaaaagagtgataacttcttcagcttcaaatatcaccccagcttttttctgctcatgaaggttagatgcctgctgcttaagtaattcctctttatctgtaaaggctttttgaagtgcatcacctgaccgggcagatagttcaccggggtgagaaaaaagagcaacaactgatttaggcaatttggcggtgttgatacagcgggtaataatcttacgtgaaatattttccgcatcagccagcgcagaaatatttccagcaaattcattctgcaatcggcttgcataacgctgaccacgttcataagcacttgttgggcgataatcgttacccaatctggataatgcagccatctgctcatcatccagctcgccaaccagaacacgataatcactttcggtaagtgcagcagctttacgacggcgactcccatcggcaatttctatgacaccagatactcttcgaccgaacgccggtgtctgttgaccagtcagtagaaaagaagggatgagatcatccagtgcgtcctcagtaagcagctcctggtcacgttcattacctgaccatacccgagaggtcttctcaacactatcaccccggagcacttcaagagtaaacttcacatcccgaccacatacaggcaaagtaatggcattaccgcgagccattactcctacgcgcgcaattaacgaatccaccatcggggcagctggtgtcgataacgaagtatcttcaaccggttgagtattgagcgtatgttttggaataacaggcgcacgcttcattatctaatctcccagcgtggtttaatcagacgatcgaaaatttcattgcagacaggttcccaaatagaaagagcatttctccaggcaccagttgaagagcgttgatcaatggcctgttcaaaaacagttctcatccggatctgacctttaccaacttcatccgtttcacgtacaacattttttagaaccatgcttccccaggcatcccgaatttgctcctccatccacggggactgagagccattactattgctgtatttggtaagcaaaatacgtacatcaggctcgaaccctttaagatcaacgttcttgagcagatcacgaagcatatcgaaaaactgcagtgcggaggtgtagtcaaacaactcagcaggcgtgggaacaatcagcacatcagcagcacatacgacattaatcgtgccgatacccaggttaggcgcgctgtcaataactatgacatcatagtcatgagcaacagtttcaatggccagtcggagcatcaggtgtggatcggtgggcagtttaccttcatcaaatttgcccattaactcagtttcaatacggtgcagagccagacaggaaggaataatgtcaagccccggccagcaagtgggctttattgcataagtgacatcgtccttttccccaagatagaaaggcaggagagtgtcttctgcatgaatatgaagatctggtacccatccgtgatacattgaggctgttccctgggggtcgttaccttccacgagcaaaacacgtagccccttcagagccagatcctgagcaagatgaacagaaactgaggttttgtaaacgccacctttatgggcagcaaccccgatcaccggtggaaatacgtcttcagcacgtcgcaatcgcgtaccaaacacatcacgcatatgattaatttgttcaattgtataaccaacacgttgctcaacccgtcctcgaatttccatatccgggtgcggtagtcgccctgctttctcggcatctctgatagcctgagaagaaaccccaactaaatccgctgcttcacctattctccagcgccgggttattttcctcgcttccgggctgtcatcattaaactgtgcaatggcgatagccttcgtcatttcatgaccagcgtttatgcactggttaagtgtttccatgagtttcattctgaacatcctttaatcattgctttgcgtttttttattaaatcttgcaatttactgcaaagcaacaacaaaatcgcaaagtcatcaaaaaaccgcaaagttgtttaaaataagagcaacactacaaaaggagataagaagagcacatacctcagtcacttattatcactagcgctcgccgcagccgtgtaaccgagcatagcgagcgaactggcgaggaagcaaagaagaactgttctgtcagatagctcttacgctcagcgcaagaagaaatatccaccgtgggaaaaactccaggtagaggtacacacgcggatagccaattcagagtaataaactgtgataatcaaccctcatcaatgatgacgaactaacccccgatatcaggtcacatgacgaagggaaagagaaggaaatcaactgtgacaaactgccctcaaatttggcttccttaaaaattacagttcaaaaagtatgagaaaatccatgcaggctgaaggaaacagcaaaactgtgacaaattaccctcagtaggtcagaacaaatgtgacgaaccaccctcaaatctgtgacagataaccctcagactatcctgtcgtcatggaagtgatatcgcggaaggaaaatacgatatgagtcgtctggcggcctttctttttctcaatgtatgagaggcgcattggagttctgctgttgatctcattaacacagacctgcaggaagcggcggcggaagtcaggcatacgctggtaactttgaggcagctggtaacgctctatgatccagtcgattttcagagagacgatgcctgagccatccggcttacgatactgacacagggattcgtataaacgcatggcatacggattggtgatttcttttgtttcactaagccgaaactgcgtaaaccggttctgtaacccgataaagaagggaatgagatatgggttgatatgtacactgtaaagccctctggatggactgtgcgcacgtttgataaaccaaggaaaagattcatagcctttttcatcgccggcatcctcttcagggcgataaaaaaccacttccttccccgcgaaactcttcaatgcctgccgtatatccttactggcttccgcagaggtcaatccgaatatttcagcatatttagcaacatggatctcgcagataccgtcatgttcctgtagggtgccatcagattttctgatctggtcaacgaacagatacagcatacgtttttgatcccgggagagactatatgccgcctcagtgaggtcgtttgactggacgattcgcgggctatttttacgtttcttgtgattgataaccgctgtttccgccatgacagatccatgtgaagtgtgacaagtttttagattgtcacactaaataaaaaagagtcaataagcagggataactttgtgaaaaaacagcttcttctgagggcaatttgtcacagggttaagggcaatttgtcacagACAGGACTGTCATTTGAGGGTGATTTGTCACACTGAAAGGGCAATTTGTCACAACACCTTCTCTAGAACCAGCATGGATAAAGGCCTACAAGGCGCTCTAAAAAAGAAGATCTAAAAACTATAAAAAAAATAATTATAAAAATATCCCCGTGGATAAGTGGATAACCCCAAGGGAAGTTTTTTCAGGCATCGTGTGTAAGCAGAATATATAAGTGCTGTTCCCTGGTGCTTCCTCGCTCACTCGAGGGCTTCGCCCTGTCGCTCAACTGCGGCGAGCACTACTGGCTGTAAaaggacagaccacatcatggttctgtgttcattaggttgttctgtccattgctgacataatccgctccacttcaacgtaacaccgcacgaagatttctattgttcctgaaggcatattcaaatcgttttcgttaccgcttgcaggcatcatgacagaacactacttcctataaacgctacacaggctcctgagattaataatgcggatctctacgataatgggagattttcccgactgtttcgttcgcttctcagtggataacagccagcttctctgtttaacagacaaaaacagcatatccactcagttccacatttccatataaaggccaaggcatttattctcaggataattgtttcagcatcgcaaccgcatcagactccggcatcgcaaactgcacccggtgccgggcagccacatccagcgcaaaaaccttcgtgtagacttccgttgaactgatggacttatgtcccatcaggctttgcagaactttcagcggtataccggcatacagcatgtgcatcgcataggaatggcggaacgtatgtggtgtgaccggaacagagaacgtcacaccgtcagcagcagcggcggcaaccgcctccccaatccaggtcctgaccgttctgtccgtcacttcccagatccgcgctttctctgtccttcctgtgcgacggttacgccgctccatgagcttatcgcgaataaatacctgtgacggaagatcacttcgcagaataaataaatcctggtgtccctgttgataccgggaagccctgggccaacttttggcgaaaatgagacgttgatcggcacgtaagaggttccaactttcaccataatgaaataagatcactaccgggcgtattttttgagttatcgagattttcaggagctaaggaagctaaaatggagaaaaaaatcactggatataccaccgttgatatatcccaatggcatcgtaaagaacattttgaggcatttcagtcagttgctcaatgtacctataaccagaccgttcagctggatattacggcctttttaaagaccgtaaagaaaaataagcacaagttttatccggcctttattcacattcttgcccgcctgatgaatgctcatccggagttccgtatggcaatgaaagacggtgagctggtgatatgggatagtgttcacccttgttacaccgttttccatgagcaaactgaaacgttttcatcgctctggagtgaataccacgacgatttccggcagtttctacacatatattcgcaagatgtggcgtgttacggtgaaaacctggcctatttccctaaagggtttattgagaatatgtttttcgtctcagccaatccctgggtgagtttcaccagttttgatttaaacgtggccaatatggacaacttcttcgcccccgttttcaccatgggcaaatattatacgcaaggcgacaaggtgctgatgccgctggcgattcaggttcatcatgccgtttgtgatggcttccatgtcggcagaatgcttaatgaattacaacagtactgcgatgagtggcagggcggggcgtaatttttttaaggcagttattggtgcccttaaacgcctggttgctacgcctgaataagtgataataagcggatgaatggcagaaattcgatgataagctgtcaaacatgagaattggtcgacggcgcgccaaagcttgcatgcctgcagccgcgtaacctggcaaaatcggttacggttgagtaataaatggatgccctgcgtaagcggggcacatttcattacctctttctccgcacccgacatagataataacttcgtatagtatacattatacgaagttatctagtagacttaattaaggatcgatccggcgcgccaatagtcatgccccgcgcccaccggaaggagctgactgggttgaaggctctcaagggcatcggtcgagcttgacattgtaggactatattgctctaataaatttgcggccgctaatacgactcactatagggagag |
| pRC1726  (Bp4++ Hsmar1, no Flag) | gatccgcggaattgCAGCTGGTTAATAACAGGGGACGTGGTAATCCGTCCCCTTTTTATTTCTGACTGAGTTAATAACAGGCCTGCTTCGGCAGGCCTTTTTATTTCTGACTGAGTTCTTCTCAGGCCTGCTGGTAATCGCAGGCCTTTTTATTTTCTAGGCAATTCCGACGTCTAAGAAACCATTATTATCATGACATTAACCTATAAAAATAGGCGTATCACGAGGCCCTCTCGTCTTCACCTCGAGTCCCTATCAGTGATAGGGATTGACATCCCTATCAGTGATAGAGACACTGGGCACATCAGCAGGACGCACTGACCACTTTAAGAAGGAGATATACATATGGAAATGATGCTCGATAAGAAACAGATTCGTGCGATCTTTCTCTTTGAGTTTAAAATGGGTCGCAAAGCGGCGGAGACGACGCGTAATATTAACAACGCGTTCGGTCCTGGCACCGCGAACGAGCGTACCGTGCAATGGTGGTTCAAAAAGTTTCGCAAAGGCGACGAATCTCTGGAGGACGAAGAGCGTTCTGGCCGCCCGTCCGAGGTTGACAACGACCAGCTGCGTGCAATCATCGAAGCTGATCCGCTGACTACCACCCGCGAAGTTGCTGAAGAACTGAATGTGGATCACTCTACTGTGGTTCGCCACCTGAAACAGATCGGTAAAGTAAAAAAACTGGACAAATGGGTTCCTCATGAACTGTCTGAAAACCAGAAAAACCGTCGTTTCGAAGTTAGCTCCTCTCTGATTCTGCGTAACAACAACGAACCGTTCCTGGATCGTATCGTAACCTGTGATGAGAAATGGATTCTGTATGATAACCGTCGCCGCTCTGCTCAGTGGCTGGATCGCGAAGAAGCTCCAAAACACTTCCCGAAACCGAATCTGCACCAGAAGAAAGTCATGGTAACCGTATGGTGGTCTGCCGCAGGTGTTATCCACTATTCCTTCCTGAACCCGGGCGAAACTATCACCAGCGAAAAATACTGCCAGCAGATTGACGAAATGCACCGTAAACTGCAGCGTCTGCAGCCAGCACTGGTGAATCGTAAAGGTCCGATCCTGCTGCATGATAACGCCCGTCCGCACGTTGCCCAACCGACCCTGCAGAAACTGAACGAACTGGGCTATGAAGTTCTGCCACACCCGCCGTACTCCCCGGATCTGTCCCCGACTGACTACCATTTCTTCAAGCATCTGGACAACTTCCTGCAGGGTAAACGTTTTCACAACCAACAGGACGCAGAAAACGCTTTCCAGGAGTTCGTCGAAAGCCGTTCCACTGACTTCTACGCGACCGGTATCAACAAGCTGATCAGCCGTTGGCAGAAATGCGTGGACTGTAACGGCAGCTACTTCGATTAAGGATCCGTTCTTCTCAGGCCTGCTGGTAATCGCAGGCCTTTTTATTTCAGCTGcaattccgcggatccttctatagtgtcacctaaatgtcgacggccaggcggccgccaggcctacccactagtcaattcgggaggatcgaaacggcagatcgcaaaaaacagtacatacagaaggagacatgaacatgaacatcaaaaaaattgtaaaacaagccacagttctgacttttacgactgcacttctggcaggaggagcgactcaagccttcgcgaaagaaaataaccaaaaagcatacaaagaaacgtacggcgtctctcatattacacgccatgatatgctgcagatccctaaacagcagcaaaacgaaaaataccaagtgcctcaattcgatcaatcaacgattaaaaatattgagtctgcaaaaggacttgatgtgtgggacagctggccgctgcaaaacgctgacggaacagtagctgaatacaacggctatcacgttgtgtttgctcttgcgggaagcccgaaagacgctgatgacacatcaatctacatgttttatcaaaaggtcggcgacaactcaatcgacagctggaaaaacgcgggccgtgtctttaaagacagcgataagttcgacgccaacgatccgatcctgaaagatcagacgcaagaatggtccggttctgcaacctttacatctgacggaaaaatccgtttattctacactgactattccggtaaacattacggcaaacaaagcctgacaacagcgcaggtaaatgtgtcaaaatctgatgacacactcaaaatcaacggagtggaagatcacaaaacgatttttgacggagacggaaaaacatatcagaacgttcagcagtttatcgatgaaggcaattatacatccggcgacaaccatacgctgagagaccctcactacgttgaagacaaaggccataaataccttgtattcgaagccaacacgggaacagaaaacggataccaaggcgaagaatctttatttaacaaagcgtactacggcggcggcacgaacttcttccgtaaagaaagccagaagcttcagcagagcgctaaaaaacgcgatgctgagttagcgaacggcgccctcggtatcatagagttaaataatgattacacattgaaaaaagtaatgaagccgctgatcacttcaaacacggtaactgatgaaatcgagcgcgcgaatgttttcaaaatgaacggcaaatggtacttgttcactgattcacgcggttcaaaaatgacgatcgatggtattaactcaaacgatatttacatgcttggttatgtatcaaactctttaaccggcccttacaagccgctgaacaaaacagggcttgtgctgcaaatgggtcttgatccaaacgatgtgacattcacttactctcacttcgcagtgccgcaagccaaaggcaacaatgtggttatcacaagctacatgacaaacagaggcttcttcgaggataaaaaggcaacatttgcgccaagcttcttaatgaacatcaaaggcaataaaacatccgttgtcaaaaacagcatcctggagcaaggacagctgacagtcaactaataacagcaaaaagaaaatgccgatacttcattggcattttcttttatttctcaacaagatggtgaattgactagtgggtagatccacaggacgggtgtggtcgccatgatcgcgtagtcgatagtggctccaagtagcgaagcgagcaggactgggcggcggccaaagcggtcggacagtgctccgagaacgggtgcgcatagaaattgcatcaacgcatatagcgctagcagcacgccatagtgactggcgatgctgtcggaatggacgatatcccgcaagaggcccggcagtaccggcataaccaagcctatgcctacagcatccagggtgacggtgccgaggatgacgatgagcgcattgttagatttcatacacggtgcctgactgcgttagcaatttaactgtgataaactaccgcattaaagcttatcgatgataagctgtcaaacatgagaattgatccggaacccttaatataacttcgtataatgtatgctatacgaagttattaggtccctcgactatagggtcaccgtcgacagcgacacacttgcatcggatgcagcccggttaacgtgccggcacggcctgggtaaccaggtattttgtccacataaccgtgcgcaaaatgttgtggataagcaggacacagcagcaatccacagcaggcatacaaccgcacaccgaggttactccgttctacaggttacgacgacatgtcaatacttgcccttgacaggcattgatggaatcgtagtctcacgctgatagtctgatcgacaatacaagtgggaccgtggtcccagaccgataatcagaccgacaacacgagtgggatcgtggtcccagactaataatcagaccgacgatacgagtgggaccgtggtcccagactaataatcagaccgacgatacgagtgggaccgtggttccagactaataatcagaccgacgatacgagtgggaccgtggtcccagactaataatcagaccgacgatacgagtgggaccatggtcccagactaataatcagaccgacgatacgagtgggaccgtggtcccagtctgattatcagaccgacgatacgagtgggaccgtggtcccagactaataatcagaccgacgatacgagtgggaccgtggtcccagactaataatcagaccgacgatacgagtgggaccgtggtcccagtctgattatcagaccgacgatacaagtggaacagtgggcccagagagaatattcaggccagttatgctttctggcctgtaacaaaggacattaagtaaagacagataaacgtagactaaaacgtggtcgcatcagggtgctggcttttcaagttccttaagaatggcctcaattttctctatacactcagttggaacacgagacctgtccaggttaagcaccattttatcgcccttatacaatactgtcgctccaggagcaaactgatgtcgtgagcttaaactagttcttgatgcagatgacgttttaagcacagaagttaaaagagtgataacttcttcagcttcaaatatcaccccagcttttttctgctcatgaaggttagatgcctgctgcttaagtaattcctctttatctgtaaaggctttttgaagtgcatcacctgaccgggcagatagttcaccggggtgagaaaaaagagcaacaactgatttaggcaatttggcggtgttgatacagcgggtaataatcttacgtgaaatattttccgcatcagccagcgcagaaatatttccagcaaattcattctgcaatcggcttgcataacgctgaccacgttcataagcacttgttgggcgataatcgttacccaatctggataatgcagccatctgctcatcatccagctcgccaaccagaacacgataatcactttcggtaagtgcagcagctttacgacggcgactcccatcggcaatttctatgacaccagatactcttcgaccgaacgccggtgtctgttgaccagtcagtagaaaagaagggatgagatcatccagtgcgtcctcagtaagcagctcctggtcacgttcattacctgaccatacccgagaggtcttctcaacactatcaccccggagcacttcaagagtaaacttcacatcccgaccacatacaggcaaagtaatggcattaccgcgagccattactcctacgcgcgcaattaacgaatccaccatcggggcagctggtgtcgataacgaagtatcttcaaccggttgagtattgagcgtatgttttggaataacaggcgcacgcttcattatctaatctcccagcgtggtttaatcagacgatcgaaaatttcattgcagacaggttcccaaatagaaagagcatttctccaggcaccagttgaagagcgttgatcaatggcctgttcaaaaacagttctcatccggatctgacctttaccaacttcatccgtttcacgtacaacattttttagaaccatgcttccccaggcatcccgaatttgctcctccatccacggggactgagagccattactattgctgtatttggtaagcaaaatacgtacatcaggctcgaaccctttaagatcaacgttcttgagcagatcacgaagcatatcgaaaaactgcagtgcggaggtgtagtcaaacaactcagcaggcgtgggaacaatcagcacatcagcagcacatacgacattaatcgtgccgatacccaggttaggcgcgctgtcaataactatgacatcatagtcatgagcaacagtttcaatggccagtcggagcatcaggtgtggatcggtgggcagtttaccttcatcaaatttgcccattaactcagtttcaatacggtgcagagccagacaggaaggaataatgtcaagccccggccagcaagtgggctttattgcataagtgacatcgtccttttccccaagatagaaaggcaggagagtgtcttctgcatgaatatgaagatctggtacccatccgtgatacattgaggctgttccctgggggtcgttaccttccacgagcaaaacacgtagccccttcagagccagatcctgagcaagatgaacagaaactgaggttttgtaaacgccacctttatgggcagcaaccccgatcaccggtggaaatacgtcttcagcacgtcgcaatcgcgtaccaaacacatcacgcatatgattaatttgttcaattgtataaccaacacgttgctcaacccgtcctcgaatttccatatccgggtgcggtagtcgccctgctttctcggcatctctgatagcctgagaagaaaccccaactaaatccgctgcttcacctattctccagcgccgggttattttcctcgcttccgggctgtcatcattaaactgtgcaatggcgatagccttcgtcatttcatgaccagcgtttatgcactggttaagtgtttccatgagtttcattctgaacatcctttaatcattgctttgcgtttttttattaaatcttgcaatttactgcaaagcaacaacaaaatcgcaaagtcatcaaaaaaccgcaaagttgtttaaaataagagcaacactacaaaaggagataagaagagcacatacctcagtcacttattatcactagcgctcgccgcagccgtgtaaccgagcatagcgagcgaactggcgaggaagcaaagaagaactgttctgtcagatagctcttacgctcagcgcaagaagaaatatccaccgtgggaaaaactccaggtagaggtacacacgcggatagccaattcagagtaataaactgtgataatcaaccctcatcaatgatgacgaactaacccccgatatcaggtcacatgacgaagggaaagagaaggaaatcaactgtgacaaactgccctcaaatttggcttccttaaaaattacagttcaaaaagtatgagaaaatccatgcaggctgaaggaaacagcaaaactgtgacaaattaccctcagtaggtcagaacaaatgtgacgaaccaccctcaaatctgtgacagataaccctcagactatcctgtcgtcatggaagtgatatcgcggaaggaaaatacgatatgagtcgtctggcggcctttctttttctcaatgtatgagaggcgcattggagttctgctgttgatctcattaacacagacctgcaggaagcggcggcggaagtcaggcatacgctggtaactttgaggcagctggtaacgctctatgatccagtcgattttcagagagacgatgcctgagccatccggcttacgatactgacacagggattcgtataaacgcatggcatacggattggtgatttcttttgtttcactaagccgaaactgcgtaaaccggttctgtaacccgataaagaagggaatgagatatgggttgatatgtacactgtaaagccctctggatggactgtgcgcacgtttgataaaccaaggaaaagattcatagcctttttcatcgccggcatcctcttcagggcgataaaaaaccacttccttccccgcgaaactcttcaatgcctgccgtatatccttactggcttccgcagaggtcaatccgaatatttcagcatatttagcaacatggatctcgcagataccgtcatgttcctgtagggtgccatcagattttctgatctggtcaacgaacagatacagcatacgtttttgatcccgggagagactatatgccgcctcagtgaggtcgtttgactggacgattcgcgggctatttttacgtttcttgtgattgataaccgctgtttccgccatgacagatccatgtgaagtgtgacaagtttttagattgtcacactaaataaaaaagagtcaataagcagggataactttgtgaaaaaacagcttcttctgagggcaatttgtcacagggttaagggcaatttgtcacagACAGGACTGTCATTTGAGGGTGATTTGTCACACTGAAAGGGCAATTTGTCACAACACCTTCTCTAGAACCAGCATGGATAAAGGCCTACAAGGCGCTCTAAAAAAGAAGATCTAAAAACTATAAAAAAAATAATTATAAAAATATCCCCGTGGATAAGTGGATAACCCCAAGGGAAGTTTTTTCAGGCATCGTGTGTAAGCAGAATATATAAGTGCTGTTCCCTGGTGCTTCCTCGCTCACTCGAGGGCTTCGCCCTGTCGCTCAACTGCGGCGAGCACTACTGGCTGTAAaaggacagaccacatcatggttctgtgttcattaggttgttctgtccattgctgacataatccgctccacttcaacgtaacaccgcacgaagatttctattgttcctgaaggcatattcaaatcgttttcgttaccgcttgcaggcatcatgacagaacactacttcctataaacgctacacaggctcctgagattaataatgcggatctctacgataatgggagattttcccgactgtttcgttcgcttctcagtggataacagccagcttctctgtttaacagacaaaaacagcatatccactcagttccacatttccatataaaggccaaggcatttattctcaggataattgtttcagcatcgcaaccgcatcagactccggcatcgcaaactgcacccggtgccgggcagccacatccagcgcaaaaaccttcgtgtagacttccgttgaactgatggacttatgtcccatcaggctttgcagaactttcagcggtataccggcatacagcatgtgcatcgcataggaatggcggaacgtatgtggtgtgaccggaacagagaacgtcacaccgtcagcagcagcggcggcaaccgcctccccaatccaggtcctgaccgttctgtccgtcacttcccagatccgcgctttctctgtccttcctgtgcgacggttacgccgctccatgagcttatcgcgaataaatacctgtgacggaagatcacttcgcagaataaataaatcctggtgtccctgttgataccgggaagccctgggccaacttttggcgaaaatgagacgttgatcggcacgtaagaggttccaactttcaccataatgaaataagatcactaccgggcgtattttttgagttatcgagattttcaggagctaaggaagctaaaatggagaaaaaaatcactggatataccaccgttgatatatcccaatggcatcgtaaagaacattttgaggcatttcagtcagttgctcaatgtacctataaccagaccgttcagctggatattacggcctttttaaagaccgtaaagaaaaataagcacaagttttatccggcctttattcacattcttgcccgcctgatgaatgctcatccggagttccgtatggcaatgaaagacggtgagctggtgatatgggatagtgttcacccttgttacaccgttttccatgagcaaactgaaacgttttcatcgctctggagtgaataccacgacgatttccggcagtttctacacatatattcgcaagatgtggcgtgttacggtgaaaacctggcctatttccctaaagggtttattgagaatatgtttttcgtctcagccaatccctgggtgagtttcaccagttttgatttaaacgtggccaatatggacaacttcttcgcccccgttttcaccatgggcaaatattatacgcaaggcgacaaggtgctgatgccgctggcgattcaggttcatcatgccgtttgtgatggcttccatgtcggcagaatgcttaatgaattacaacagtactgcgatgagtggcagggcggggcgtaatttttttaaggcagttattggtgcccttaaacgcctggttgctacgcctgaataagtgataataagcggatgaatggcagaaattcgatgataagctgtcaaacatgagaattggtcgacggcgcgccaaagcttgcatgcctgcagccgcgtaacctggcaaaatcggttacggttgagtaataaatggatgccctgcgtaagcggggcacatttcattacctctttctccgcacccgacatagataataacttcgtatagtatacattatacgaagttatctagtagacttaattaaggatcgatccggcgcgccaatagtcatgccccgcgcccaccggaaggagctgactgggttgaaggctctcaagggcatcggtcgagcttgacattgtaggactatattgctctaataaatttgcggccgctaatacgactcactatagggagag |
| pRC1727  (Bp5++ Hsmar1, no Flag) | gatccgcggaattgCAGCTGGTTAATAACAGGGGACGTGGTAATCCGTCCCCTTTTTATTTCTGACTGAGTTAATAACAGGCCTGCTTCGGCAGGCCTTTTTATTTCTGACTGAGTTCTTCTCAGGCCTGCTGGTAATCGCAGGCCTTTTTATTTTCTAGCCAATTCCGACGCCTAAGAAACCATTATTATCATGACATTAGCCTATAAAAATAGGCGTACCACGAGGCCCTTTCGTCTTCACCTCGAGTCCCTATCAGTGATAGAGATTGACACCCCTATCAGTGATAGAGATACTGAGCACATCAGCAGGACGCACTGACCACTTTAAGAAGGAGATATACATATGGAAATGATGCTCGATAAGAAACAGATTCGTGCGATCTTTCTCTTTGAGTTTAAAATGGGTCGCAAAGCGGCGGAGACGACGCGTAATATTAACAACGCGTTCGGTCCTGGCACCGCGAACGAGCGTACCGTGCAATGGTGGTTCAAAAAGTTTCGCAAAGGCGACGAATCTCTGGAGGACGAAGAGCGTTCTGGCCGCCCGTCCGAGGTTGACAACGACCAGCTGCGTGCAATCATCGAAGCTGATCCGCTGACTACCACCCGCGAAGTTGCTGAAGAACTGAATGTGGATCACTCTACTGTGGTTCGCCACCTGAAACAGATCGGTAAAGTAAAAAAACTGGACAAATGGGTTCCTCATGAACTGTCTGAAAACCAGAAAAACCGTCGTTTCGAAGTTAGCTCCTCTCTGATTCTGCGTAACAACAACGAACCGTTCCTGGATCGTATCGTAACCTGTGATGAGAAATGGATTCTGTATGATAACCGTCGCCGCTCTGCTCAGTGGCTGGATCGCGAAGAAGCTCCAAAACACTTCCCGAAACCGAATCTGCACCAGAAGAAAGTCATGGTAACCGTATGGTGGTCTGCCGCAGGTGTTATCCACTATTCCTTCCTGAACCCGGGCGAAACTATCACCAGCGAAAAATACTGCCAGCAGATTGACGAAATGCACCGTAAACTGCAGCGTCTGCAGCCAGCACTGGTGAATCGTAAAGGTCCGATCCTGCTGCATGATAACGCCCGTCCGCACGTTGCCCAACCGACCCTGCAGAAACTGAACGAACTGGGCTATGAAGTTCTGCCACACCCGCCGTACTCCCCGGATCTGTCCCCGACTGACTACCATTTCTTCAAGCATCTGGACAACTTCCTGCAGGGTAAACGTTTTCACAACCAACAGGACGCAGAAAACGCTTTCCAGGAGTTCGTCGAAAGCCGTTCCACTGACTTCTACGCGACCGGTATCAACAAGCTGATCAGCCGTTGGCAGAAATGCGTGGACTGTAACGGCAGCTACTTCGATTAAGGATCCGTTCTTCTCAGGCCTGCTGGTAATCGCAGGCCTTTTTATTTCAGCTGcaattccgcggatccttctatagtgtcacctaaatgtcgacggccaggcggccgccaggcctacccactagtcaattcgggaggatcgaaacggcagatcgcaaaaaacagtacatacagaaggagacatgaacatgaacatcaaaaaaattgtaaaacaagccacagttctgacttttacgactgcacttctggcaggaggagcgactcaagccttcgcgaaagaaaataaccaaaaagcatacaaagaaacgtacggcgtctctcatattacacgccatgatatgctgcagatccctaaacagcagcaaaacgaaaaataccaagtgcctcaattcgatcaatcaacgattaaaaatattgagtctgcaaaaggacttgatgtgtgggacagctggccgctgcaaaacgctgacggaacagtagctgaatacaacggctatcacgttgtgtttgctcttgcgggaagcccgaaagacgctgatgacacatcaatctacatgttttatcaaaaggtcggcgacaactcaatcgacagctggaaaaacgcgggccgtgtctttaaagacagcgataagttcgacgccaacgatccgatcctgaaagatcagacgcaagaatggtccggttctgcaacctttacatctgacggaaaaatccgtttattctacactgactattccggtaaacattacggcaaacaaagcctgacaacagcgcaggtaaatgtgtcaaaatctgatgacacactcaaaatcaacggagtggaagatcacaaaacgatttttgacggagacggaaaaacatatcagaacgttcagcagtttatcgatgaaggcaattatacatccggcgacaaccatacgctgagagaccctcactacgttgaagacaaaggccataaataccttgtattcgaagccaacacgggaacagaaaacggataccaaggcgaagaatctttatttaacaaagcgtactacggcggcggcacgaacttcttccgtaaagaaagccagaagcttcagcagagcgctaaaaaacgcgatgctgagttagcgaacggcgccctcggtatcatagagttaaataatgattacacattgaaaaaagtaatgaagccgctgatcacttcaaacacggtaactgatgaaatcgagcgcgcgaatgttttcaaaatgaacggcaaatggtacttgttcactgattcacgcggttcaaaaatgacgatcgatggtattaactcaaacgatatttacatgcttggttatgtatcaaactctttaaccggcccttacaagccgctgaacaaaacagggcttgtgctgcaaatgggtcttgatccaaacgatgtgacattcacttactctcacttcgcagtgccgcaagccaaaggcaacaatgtggttatcacaagctacatgacaaacagaggcttcttcgaggataaaaaggcaacatttgcgccaagcttcttaatgaacatcaaaggcaataaaacatccgttgtcaaaaacagcatcctggagcaaggacagctgacagtcaactaataacagcaaaaagaaaatgccgatacttcattggcattttcttttatttctcaacaagatggtgaattgactagtgggtagatccacaggacgggtgtggtcgccatgatcgcgtagtcgatagtggctccaagtagcgaagcgagcaggactgggcggcggccaaagcggtcggacagtgctccgagaacgggtgcgcatagaaattgcatcaacgcatatagcgctagcagcacgccatagtgactggcgatgctgtcggaatggacgatatcccgcaagaggcccggcagtaccggcataaccaagcctatgcctacagcatccagggtgacggtgccgaggatgacgatgagcgcattgttagatttcatacacggtgcctgactgcgttagcaatttaactgtgataaactaccgcattaaagcttatcgatgataagctgtcaaacatgagaattgatccggaacccttaatataacttcgtataatgtatgctatacgaagttattaggtccctcgactatagggtcaccgtcgacagcgacacacttgcatcggatgcagcccggttaacgtgccggcacggcctgggtaaccaggtattttgtccacataaccgtgcgcaaaatgttgtggataagcaggacacagcagcaatccacagcaggcatacaaccgcacaccgaggttactccgttctacaggttacgacgacatgtcaatacttgcccttgacaggcattgatggaatcgtagtctcacgctgatagtctgatcgacaatacaagtgggaccgtggtcccagaccgataatcagaccgacaacacgagtgggatcgtggtcccagactaataatcagaccgacgatacgagtgggaccgtggtcccagactaataatcagaccgacgatacgagtgggaccgtggttccagactaataatcagaccgacgatacgagtgggaccgtggtcccagactaataatcagaccgacgatacgagtgggaccatggtcccagactaataatcagaccgacgatacgagtgggaccgtggtcccagtctgattatcagaccgacgatacgagtgggaccgtggtcccagactaataatcagaccgacgatacgagtgggaccgtggtcccagactaataatcagaccgacgatacgagtgggaccgtggtcccagtctgattatcagaccgacgatacaagtggaacagtgggcccagagagaatattcaggccagttatgctttctggcctgtaacaaaggacattaagtaaagacagataaacgtagactaaaacgtggtcgcatcagggtgctggcttttcaagttccttaagaatggcctcaattttctctatacactcagttggaacacgagacctgtccaggttaagcaccattttatcgcccttatacaatactgtcgctccaggagcaaactgatgtcgtgagcttaaactagttcttgatgcagatgacgttttaagcacagaagttaaaagagtgataacttcttcagcttcaaatatcaccccagcttttttctgctcatgaaggttagatgcctgctgcttaagtaattcctctttatctgtaaaggctttttgaagtgcatcacctgaccgggcagatagttcaccggggtgagaaaaaagagcaacaactgatttaggcaatttggcggtgttgatacagcgggtaataatcttacgtgaaatattttccgcatcagccagcgcagaaatatttccagcaaattcattctgcaatcggcttgcataacgctgaccacgttcataagcacttgttgggcgataatcgttacccaatctggataatgcagccatctgctcatcatccagctcgccaaccagaacacgataatcactttcggtaagtgcagcagctttacgacggcgactcccatcggcaatttctatgacaccagatactcttcgaccgaacgccggtgtctgttgaccagtcagtagaaaagaagggatgagatcatccagtgcgtcctcagtaagcagctcctggtcacgttcattacctgaccatacccgagaggtcttctcaacactatcaccccggagcacttcaagagtaaacttcacatcccgaccacatacaggcaaagtaatggcattaccgcgagccattactcctacgcgcgcaattaacgaatccaccatcggggcagctggtgtcgataacgaagtatcttcaaccggttgagtattgagcgtatgttttggaataacaggcgcacgcttcattatctaatctcccagcgtggtttaatcagacgatcgaaaatttcattgcagacaggttcccaaatagaaagagcatttctccaggcaccagttgaagagcgttgatcaatggcctgttcaaaaacagttctcatccggatctgacctttaccaacttcatccgtttcacgtacaacattttttagaaccatgcttccccaggcatcccgaatttgctcctccatccacggggactgagagccattactattgctgtatttggtaagcaaaatacgtacatcaggctcgaaccctttaagatcaacgttcttgagcagatcacgaagcatatcgaaaaactgcagtgcggaggtgtagtcaaacaactcagcaggcgtgggaacaatcagcacatcagcagcacatacgacattaatcgtgccgatacccaggttaggcgcgctgtcaataactatgacatcatagtcatgagcaacagtttcaatggccagtcggagcatcaggtgtggatcggtgggcagtttaccttcatcaaatttgcccattaactcagtttcaatacggtgcagagccagacaggaaggaataatgtcaagccccggccagcaagtgggctttattgcataagtgacatcgtccttttccccaagatagaaaggcaggagagtgtcttctgcatgaatatgaagatctggtacccatccgtgatacattgaggctgttccctgggggtcgttaccttccacgagcaaaacacgtagccccttcagagccagatcctgagcaagatgaacagaaactgaggttttgtaaacgccacctttatgggcagcaaccccgatcaccggtggaaatacgtcttcagcacgtcgcaatcgcgtaccaaacacatcacgcatatgattaatttgttcaattgtataaccaacacgttgctcaacccgtcctcgaatttccatatccgggtgcggtagtcgccctgctttctcggcatctctgatagcctgagaagaaaccccaactaaatccgctgcttcacctattctccagcgccgggttattttcctcgcttccgggctgtcatcattaaactgtgcaatggcgatagccttcgtcatttcatgaccagcgtttatgcactggttaagtgtttccatgagtttcattctgaacatcctttaatcattgctttgcgtttttttattaaatcttgcaatttactgcaaagcaacaacaaaatcgcaaagtcatcaaaaaaccgcaaagttgtttaaaataagagcaacactacaaaaggagataagaagagcacatacctcagtcacttattatcactagcgctcgccgcagccgtgtaaccgagcatagcgagcgaactggcgaggaagcaaagaagaactgttctgtcagatagctcttacgctcagcgcaagaagaaatatccaccgtgggaaaaactccaggtagaggtacacacgcggatagccaattcagagtaataaactgtgataatcaaccctcatcaatgatgacgaactaacccccgatatcaggtcacatgacgaagggaaagagaaggaaatcaactgtgacaaactgccctcaaatttggcttccttaaaaattacagttcaaaaagtatgagaaaatccatgcaggctgaaggaaacagcaaaactgtgacaaattaccctcagtaggtcagaacaaatgtgacgaaccaccctcaaatctgtgacagataaccctcagactatcctgtcgtcatggaagtgatatcgcggaaggaaaatacgatatgagtcgtctggcggcctttctttttctcaatgtatgagaggcgcattggagttctgctgttgatctcattaacacagacctgcaggaagcggcggcggaagtcaggcatacgctggtaactttgaggcagctggtaacgctctatgatccagtcgattttcagagagacgatgcctgagccatccggcttacgatactgacacagggattcgtataaacgcatggcatacggattggtgatttcttttgtttcactaagccgaaactgcgtaaaccggttctgtaacccgataaagaagggaatgagatatgggttgatatgtacactgtaaagccctctggatggactgtgcgcacgtttgataaaccaaggaaaagattcatagcctttttcatcgccggcatcctcttcagggcgataaaaaaccacttccttccccgcgaaactcttcaatgcctgccgtatatccttactggcttccgcagaggtcaatccgaatatttcagcatatttagcaacatggatctcgcagataccgtcatgttcctgtagggtgccatcagattttctgatctggtcaacgaacagatacagcatacgtttttgatcccgggagagactatatgccgcctcagtgaggtcgtttgactggacgattcgcgggctatttttacgtttcttgtgattgataaccgctgtttccgccatgacagatccatgtgaagtgtgacaagtttttagattgtcacactaaataaaaaagagtcaataagcagggataactttgtgaaaaaacagcttcttctgagggcaatttgtcacagggttaagggcaatttgtcacagACAGGACTGTCATTTGAGGGTGATTTGTCACACTGAAAGGGCAATTTGTCACAACACCTTCTCTAGAACCAGCATGGATAAAGGCCTACAAGGCGCTCTAAAAAAGAAGATCTAAAAACTATAAAAAAAATAATTATAAAAATATCCCCGTGGATAAGTGGATAACCCCAAGGGAAGTTTTTTCAGGCATCGTGTGTAAGCAGAATATATAAGTGCTGTTCCCTGGTGCTTCCTCGCTCACTCGAGGGCTTCGCCCTGTCGCTCAACTGCGGCGAGCACTACTGGCTGTAAaaggacagaccacatcatggttctgtgttcattaggttgttctgtccattgctgacataatccgctccacttcaacgtaacaccgcacgaagatttctattgttcctgaaggcatattcaaatcgttttcgttaccgcttgcaggcatcatgacagaacactacttcctataaacgctacacaggctcctgagattaataatgcggatctctacgataatgggagattttcccgactgtttcgttcgcttctcagtggataacagccagcttctctgtttaacagacaaaaacagcatatccactcagttccacatttccatataaaggccaaggcatttattctcaggataattgtttcagcatcgcaaccgcatcagactccggcatcgcaaactgcacccggtgccgggcagccacatccagcgcaaaaaccttcgtgtagacttccgttgaactgatggacttatgtcccatcaggctttgcagaactttcagcggtataccggcatacagcatgtgcatcgcataggaatggcggaacgtatgtggtgtgaccggaacagagaacgtcacaccgtcagcagcagcggcggcaaccgcctccccaatccaggtcctgaccgttctgtccgtcacttcccagatccgcgctttctctgtccttcctgtgcgacggttacgccgctccatgagcttatcgcgaataaatacctgtgacggaagatcacttcgcagaataaataaatcctggtgtccctgttgataccgggaagccctgggccaacttttggcgaaaatgagacgttgatcggcacgtaagaggttccaactttcaccataatgaaataagatcactaccgggcgtattttttgagttatcgagattttcaggagctaaggaagctaaaatggagaaaaaaatcactggatataccaccgttgatatatcccaatggcatcgtaaagaacattttgaggcatttcagtcagttgctcaatgtacctataaccagaccgttcagctggatattacggcctttttaaagaccgtaaagaaaaataagcacaagttttatccggcctttattcacattcttgcccgcctgatgaatgctcatccggagttccgtatggcaatgaaagacggtgagctggtgatatgggatagtgttcacccttgttacaccgttttccatgagcaaactgaaacgttttcatcgctctggagtgaataccacgacgatttccggcagtttctacacatatattcgcaagatgtggcgtgttacggtgaaaacctggcctatttccctaaagggtttattgagaatatgtttttcgtctcagccaatccctgggtgagtttcaccagttttgatttaaacgtggccaatatggacaacttcttcgcccccgttttcaccatgggcaaatattatacgcaaggcgacaaggtgctgatgccgctggcgattcaggttcatcatgccgtttgtgatggcttccatgtcggcagaatgcttaatgaattacaacagtactgcgatgagtggcagggcggggcgtaatttttttaaggcagttattggtgcccttaaacgcctggttgctacgcctgaataagtgataataagcggatgaatggcagaaattcgatgataagctgtcaaacatgagaattggtcgacggcgcgccaaagcttgcatgcctgcagccgcgtaacctggcaaaatcggttacggttgagtaataaatggatgccctgcgtaagcggggcacatttcattacctctttctccgcacccgacatagataataacttcgtatagtatacattatacgaagttatctagtagacttaattaaggatcgatccggcgcgccaatagtcatgccccgcgcccaccggaaggagctgactgggttgaaggctctcaagggcatcggtcgagcttgacattgtaggactatattgctctaataaatttgcggccgctaatacgactcactatagggagag |
| pRC1728  (Bp6++ Hsmar1, no Flag) | gatccgcggaattgCAGCTGGTTAATAACAGGGGACGTGGTAATCCGTCCCCTTTTTATTTCTGACTGAGTTAATAACAGGCCTGCTTCGGCAGGCCTTTTTATTTCTGACTGAGTTCTTCTCAGGCCTGCTGGTAATCGCAGGCCTTTTTATTTTCTAGTCAATTCCGACGTCTAAGAAACCATTATTATCATGACATTAACCTATAAAAATAGGCGTATCACGAGGCCCTTTCGTCTTCACCTCGAGTCCCTATCAGTGATAGAGATTGACATCCCTATCAGTGATAGAGATACTGAGCACATCAGCAGGACGCACTGACCACTTTAAGAAGGAGATATACATATGGAAATGATGCTCGATAAGAAACAGATTCGTGCGATCTTTCTCTTTGAGTTTAAAATGGGTCGCAAAGCGGCGGAGACGACGCGTAATATTAACAACGCGTTCGGTCCTGGCACCGCGAACGAGCGTACCGTGCAATGGTGGTTCAAAAAGTTTCGCAAAGGCGACGAATCTCTGGAGGACGAAGAGCGTTCTGGCCGCCCGTCCGAGGTTGACAACGACCAGCTGCGTGCAATCATCGAAGCTGATCCGCTGACTACCACCCGCGAAGTTGCTGAAGAACTGAATGTGGATCACTCTACTGTGGTTCGCCACCTGAAACAGATCGGTAAAGTAAAAAAACTGGACAAATGGGTTCCTCATGAACTGTCTGAAAACCAGAAAAACCGTCGTTTCGAAGTTAGCTCCTCTCTGATTCTGCGTAACAACAACGAACCGTTCCTGGATCGTATCGTAACCTGTGATGAGAAATGGATTCTGTATGATAACCGTCGCCGCTCTGCTCAGTGGCTGGATCGCGAAGAAGCTCCAAAACACTTCCCGAAACCGAATCTGCACCAGAAGAAAGTCATGGTAACCGTATGGTGGTCTGCCGCAGGTGTTATCCACTATTCCTTCCTGAACCCGGGCGAAACTATCACCAGCGAAAAATACTGCCAGCAGATTGACGAAATGCACCGTAAACTGCAGCGTCTGCAGCCAGCACTGGTGAATCGTAAAGGTCCGATCCTGCTGCATGATAACGCCCGTCCGCACGTTGCCCAACCGACCCTGCAGAAACTGAACGAACTGGGCTATGAAGTTCTGCCACACCCGCCGTACTCCCCGGATCTGTCCCCGACTGACTACCATTTCTTCAAGCATCTGGACAACTTCCTGCAGGGTAAACGTTTTCACAACCAACAGGACGCAGAAAACGCTTTCCAGGAGTTCGTCGAAAGCCGTTCCACTGACTTCTACGCGACCGGTATCAACAAGCTGATCAGCCGTTGGCAGAAATGCGTGGACTGTAACGGCAGCTACTTCGATTAAGGATCCGTTCTTCTCAGGCCTGCTGGTAATCGCAGGCCTTTTTATTTCAGCTGcaattccgcggatccttctatagtgtcacctaaatgtcgacggccaggcggccgccaggcctacccactagtcaattcgggaggatcgaaacggcagatcgcaaaaaacagtacatacagaaggagacatgaacatgaacatcaaaaaaattgtaaaacaagccacagttctgacttttacgactgcacttctggcaggaggagcgactcaagccttcgcgaaagaaaataaccaaaaagcatacaaagaaacgtacggcgtctctcatattacacgccatgatatgctgcagatccctaaacagcagcaaaacgaaaaataccaagtgcctcaattcgatcaatcaacgattaaaaatattgagtctgcaaaaggacttgatgtgtgggacagctggccgctgcaaaacgctgacggaacagtagctgaatacaacggctatcacgttgtgtttgctcttgcgggaagcccgaaagacgctgatgacacatcaatctacatgttttatcaaaaggtcggcgacaactcaatcgacagctggaaaaacgcgggccgtgtctttaaagacagcgataagttcgacgccaacgatccgatcctgaaagatcagacgcaagaatggtccggttctgcaacctttacatctgacggaaaaatccgtttattctacactgactattccggtaaacattacggcaaacaaagcctgacaacagcgcaggtaaatgtgtcaaaatctgatgacacactcaaaatcaacggagtggaagatcacaaaacgatttttgacggagacggaaaaacatatcagaacgttcagcagtttatcgatgaaggcaattatacatccggcgacaaccatacgctgagagaccctcactacgttgaagacaaaggccataaataccttgtattcgaagccaacacgggaacagaaaacggataccaaggcgaagaatctttatttaacaaagcgtactacggcggcggcacgaacttcttccgtaaagaaagccagaagcttcagcagagcgctaaaaaacgcgatgctgagttagcgaacggcgccctcggtatcatagagttaaataatgattacacattgaaaaaagtaatgaagccgctgatcacttcaaacacggtaactgatgaaatcgagcgcgcgaatgttttcaaaatgaacggcaaatggtacttgttcactgattcacgcggttcaaaaatgacgatcgatggtattaactcaaacgatatttacatgcttggttatgtatcaaactctttaaccggcccttacaagccgctgaacaaaacagggcttgtgctgcaaatgggtcttgatccaaacgatgtgacattcacttactctcacttcgcagtgccgcaagccaaaggcaacaatgtggttatcacaagctacatgacaaacagaggcttcttcgaggataaaaaggcaacatttgcgccaagcttcttaatgaacatcaaaggcaataaaacatccgttgtcaaaaacagcatcctggagcaaggacagctgacagtcaactaataacagcaaaaagaaaatgccgatacttcattggcattttcttttatttctcaacaagatggtgaattgactagtgggtagatccacaggacgggtgtggtcgccatgatcgcgtagtcgatagtggctccaagtagcgaagcgagcaggactgggcggcggccaaagcggtcggacagtgctccgagaacgggtgcgcatagaaattgcatcaacgcatatagcgctagcagcacgccatagtgactggcgatgctgtcggaatggacgatatcccgcaagaggcccggcagtaccggcataaccaagcctatgcctacagcatccagggtgacggtgccgaggatgacgatgagcgcattgttagatttcatacacggtgcctgactgcgttagcaatttaactgtgataaactaccgcattaaagcttatcgatgataagctgtcaaacatgagaattgatccggaacccttaatataacttcgtataatgtatgctatacgaagttattaggtccctcgactatagggtcaccgtcgacagcgacacacttgcatcggatgcagcccggttaacgtgccggcacggcctgggtaaccaggtattttgtccacataaccgtgcgcaaaatgttgtggataagcaggacacagcagcaatccacagcaggcatacaaccgcacaccgaggttactccgttctacaggttacgacgacatgtcaatacttgcccttgacaggcattgatggaatcgtagtctcacgctgatagtctgatcgacaatacaagtgggaccgtggtcccagaccgataatcagaccgacaacacgagtgggatcgtggtcccagactaataatcagaccgacgatacgagtgggaccgtggtcccagactaataatcagaccgacgatacgagtgggaccgtggttccagactaataatcagaccgacgatacgagtgggaccgtggtcccagactaataatcagaccgacgatacgagtgggaccatggtcccagactaataatcagaccgacgatacgagtgggaccgtggtcccagtctgattatcagaccgacgatacgagtgggaccgtggtcccagactaataatcagaccgacgatacgagtgggaccgtggtcccagactaataatcagaccgacgatacgagtgggaccgtggtcccagtctgattatcagaccgacgatacaagtggaacagtgggcccagagagaatattcaggccagttatgctttctggcctgtaacaaaggacattaagtaaagacagataaacgtagactaaaacgtggtcgcatcagggtgctggcttttcaagttccttaagaatggcctcaattttctctatacactcagttggaacacgagacctgtccaggttaagcaccattttatcgcccttatacaatactgtcgctccaggagcaaactgatgtcgtgagcttaaactagttcttgatgcagatgacgttttaagcacagaagttaaaagagtgataacttcttcagcttcaaatatcaccccagcttttttctgctcatgaaggttagatgcctgctgcttaagtaattcctctttatctgtaaaggctttttgaagtgcatcacctgaccgggcagatagttcaccggggtgagaaaaaagagcaacaactgatttaggcaatttggcggtgttgatacagcgggtaataatcttacgtgaaatattttccgcatcagccagcgcagaaatatttccagcaaattcattctgcaatcggcttgcataacgctgaccacgttcataagcacttgttgggcgataatcgttacccaatctggataatgcagccatctgctcatcatccagctcgccaaccagaacacgataatcactttcggtaagtgcagcagctttacgacggcgactcccatcggcaatttctatgacaccagatactcttcgaccgaacgccggtgtctgttgaccagtcagtagaaaagaagggatgagatcatccagtgcgtcctcagtaagcagctcctggtcacgttcattacctgaccatacccgagaggtcttctcaacactatcaccccggagcacttcaagagtaaacttcacatcccgaccacatacaggcaaagtaatggcattaccgcgagccattactcctacgcgcgcaattaacgaatccaccatcggggcagctggtgtcgataacgaagtatcttcaaccggttgagtattgagcgtatgttttggaataacaggcgcacgcttcattatctaatctcccagcgtggtttaatcagacgatcgaaaatttcattgcagacaggttcccaaatagaaagagcatttctccaggcaccagttgaagagcgttgatcaatggcctgttcaaaaacagttctcatccggatctgacctttaccaacttcatccgtttcacgtacaacattttttagaaccatgcttccccaggcatcccgaatttgctcctccatccacggggactgagagccattactattgctgtatttggtaagcaaaatacgtacatcaggctcgaaccctttaagatcaacgttcttgagcagatcacgaagcatatcgaaaaactgcagtgcggaggtgtagtcaaacaactcagcaggcgtgggaacaatcagcacatcagcagcacatacgacattaatcgtgccgatacccaggttaggcgcgctgtcaataactatgacatcatagtcatgagcaacagtttcaatggccagtcggagcatcaggtgtggatcggtgggcagtttaccttcatcaaatttgcccattaactcagtttcaatacggtgcagagccagacaggaaggaataatgtcaagccccggccagcaagtgggctttattgcataagtgacatcgtccttttccccaagatagaaaggcaggagagtgtcttctgcatgaatatgaagatctggtacccatccgtgatacattgaggctgttccctgggggtcgttaccttccacgagcaaaacacgtagccccttcagagccagatcctgagcaagatgaacagaaactgaggttttgtaaacgccacctttatgggcagcaaccccgatcaccggtggaaatacgtcttcagcacgtcgcaatcgcgtaccaaacacatcacgcatatgattaatttgttcaattgtataaccaacacgttgctcaacccgtcctcgaatttccatatccgggtgcggtagtcgccctgctttctcggcatctctgatagcctgagaagaaaccccaactaaatccgctgcttcacctattctccagcgccgggttattttcctcgcttccgggctgtcatcattaaactgtgcaatggcgatagccttcgtcatttcatgaccagcgtttatgcactggttaagtgtttccatgagtttcattctgaacatcctttaatcattgctttgcgtttttttattaaatcttgcaatttactgcaaagcaacaacaaaatcgcaaagtcatcaaaaaaccgcaaagttgtttaaaataagagcaacactacaaaaggagataagaagagcacatacctcagtcacttattatcactagcgctcgccgcagccgtgtaaccgagcatagcgagcgaactggcgaggaagcaaagaagaactgttctgtcagatagctcttacgctcagcgcaagaagaaatatccaccgtgggaaaaactccaggtagaggtacacacgcggatagccaattcagagtaataaactgtgataatcaaccctcatcaatgatgacgaactaacccccgatatcaggtcacatgacgaagggaaagagaaggaaatcaactgtgacaaactgccctcaaatttggcttccttaaaaattacagttcaaaaagtatgagaaaatccatgcaggctgaaggaaacagcaaaactgtgacaaattaccctcagtaggtcagaacaaatgtgacgaaccaccctcaaatctgtgacagataaccctcagactatcctgtcgtcatggaagtgatatcgcggaaggaaaatacgatatgagtcgtctggcggcctttctttttctcaatgtatgagaggcgcattggagttctgctgttgatctcattaacacagacctgcaggaagcggcggcggaagtcaggcatacgctggtaactttgaggcagctggtaacgctctatgatccagtcgattttcagagagacgatgcctgagccatccggcttacgatactgacacagggattcgtataaacgcatggcatacggattggtgatttcttttgtttcactaagccgaaactgcgtaaaccggttctgtaacccgataaagaagggaatgagatatgggttgatatgtacactgtaaagccctctggatggactgtgcgcacgtttgataaaccaaggaaaagattcatagcctttttcatcgccggcatcctcttcagggcgataaaaaaccacttccttccccgcgaaactcttcaatgcctgccgtatatccttactggcttccgcagaggtcaatccgaatatttcagcatatttagcaacatggatctcgcagataccgtcatgttcctgtagggtgccatcagattttctgatctggtcaacgaacagatacagcatacgtttttgatcccgggagagactatatgccgcctcagtgaggtcgtttgactggacgattcgcgggctatttttacgtttcttgtgattgataaccgctgtttccgccatgacagatccatgtgaagtgtgacaagtttttagattgtcacactaaataaaaaagagtcaataagcagggataactttgtgaaaaaacagcttcttctgagggcaatttgtcacagggttaagggcaatttgtcacagACAGGACTGTCATTTGAGGGTGATTTGTCACACTGAAAGGGCAATTTGTCACAACACCTTCTCTAGAACCAGCATGGATAAAGGCCTACAAGGCGCTCTAAAAAAGAAGATCTAAAAACTATAAAAAAAATAATTATAAAAATATCCCCGTGGATAAGTGGATAACCCCAAGGGAAGTTTTTTCAGGCATCGTGTGTAAGCAGAATATATAAGTGCTGTTCCCTGGTGCTTCCTCGCTCACTCGAGGGCTTCGCCCTGTCGCTCAACTGCGGCGAGCACTACTGGCTGTAAaaggacagaccacatcatggttctgtgttcattaggttgttctgtccattgctgacataatccgctccacttcaacgtaacaccgcacgaagatttctattgttcctgaaggcatattcaaatcgttttcgttaccgcttgcaggcatcatgacagaacactacttcctataaacgctacacaggctcctgagattaataatgcggatctctacgataatgggagattttcccgactgtttcgttcgcttctcagtggataacagccagcttctctgtttaacagacaaaaacagcatatccactcagttccacatttccatataaaggccaaggcatttattctcaggataattgtttcagcatcgcaaccgcatcagactccggcatcgcaaactgcacccggtgccgggcagccacatccagcgcaaaaaccttcgtgtagacttccgttgaactgatggacttatgtcccatcaggctttgcagaactttcagcggtataccggcatacagcatgtgcatcgcataggaatggcggaacgtatgtggtgtgaccggaacagagaacgtcacaccgtcagcagcagcggcggcaaccgcctccccaatccaggtcctgaccgttctgtccgtcacttcccagatccgcgctttctctgtccttcctgtgcgacggttacgccgctccatgagcttatcgcgaataaatacctgtgacggaagatcacttcgcagaataaataaatcctggtgtccctgttgataccgggaagccctgggccaacttttggcgaaaatgagacgttgatcggcacgtaagaggttccaactttcaccataatgaaataagatcactaccgggcgtattttttgagttatcgagattttcaggagctaaggaagctaaaatggagaaaaaaatcactggatataccaccgttgatatatcccaatggcatcgtaaagaacattttgaggcatttcagtcagttgctcaatgtacctataaccagaccgttcagctggatattacggcctttttaaagaccgtaaagaaaaataagcacaagttttatccggcctttattcacattcttgcccgcctgatgaatgctcatccggagttccgtatggcaatgaaagacggtgagctggtgatatgggatagtgttcacccttgttacaccgttttccatgagcaaactgaaacgttttcatcgctctggagtgaataccacgacgatttccggcagtttctacacatatattcgcaagatgtggcgtgttacggtgaaaacctggcctatttccctaaagggtttattgagaatatgtttttcgtctcagccaatccctgggtgagtttcaccagttttgatttaaacgtggccaatatggacaacttcttcgcccccgttttcaccatgggcaaatattatacgcaaggcgacaaggtgctgatgccgctggcgattcaggttcatcatgccgtttgtgatggcttccatgtcggcagaatgcttaatgaattacaacagtactgcgatgagtggcagggcggggcgtaatttttttaaggcagttattggtgcccttaaacgcctggttgctacgcctgaataagtgataataagcggatgaatggcagaaattcgatgataagctgtcaaacatgagaattggtcgacggcgcgccaaagcttgcatgcctgcagccgcgtaacctggcaaaatcggttacggttgagtaataaatggatgccctgcgtaagcggggcacatttcattacctctttctccgcacccgacatagataataacttcgtatagtatacattatacgaagttatctagtagacttaattaaggatcgatccggcgcgccaatagtcatgccccgcgcccaccggaaggagctgactgggttgaaggctctcaagggcatcggtcgagcttgacattgtaggactatattgctctaataaatttgcggccgctaatacgactcactatagggagag |
| pRC1821  (Bp-EE++ Hsmar1 Flag) | gatccgcggaattgTTAATAACAGGGGACGTGGTAATCCGTCCCCTTTTTATTTCTGACTGAGTTAATAACAGGCCTGCTTCGGCAGGCCTTTTTATTTCTGACTGAGTTCTTCTCAGGCCTGCTGGTAATCGCAGGCCTTTTTATTTTCTAGgCTGACTGACTGACTGACTGACTGACTGACTGACTGACTGACTGACTGACTGACTGACTGACTGACTGACTGACTGACTGACTGACTGACTGACTGACTGACTaaggagCTGACCATATGGAAATGATGCTCGATAAGAAACAGATTCGTGCGATCTTTCTCTTTGAGTTTAAAATGGGTCGCAAAGCGGCGGAGACGACGCGTAATATTAACAACGCGTTCGGTCCTGGCACCGCGAACGAGCGTACCGTGCAATGGTGGTTCAAAAAGTTTCGCAAAGGCGACGAATCTCTGGAGGACGAAGAGCGTTCTGGCCGCCCGTCCGAGGTTGACAACGACCAGCTGCGTGCAATCATCGAAGCTGATCCGCTGACTACCACCCGCGAAGTTGCTGAAGAACTGAATGTGGATCACTCTACTGTGGTTCGCCACCTGAAACAGATCGGTAAAGTAAAAAAACTGGACAAATGGGTTCCTCATGAACTGTCTGAAAACCAGAAAAACCGTCGTTTCGAAGTTAGCTCCTCTCTGATTCTGCGTAACAACAACGAACCGTTCCTGGATCGTATCGTAACCTGTGATGAGAAATGGATTCTGTATGATAACCGTCGCCGCTCTGCTCAGTGGCTGGATCGCGAAGAAGCTCCAAAACACTTCCCGAAACCGAATCTGCACCAGAAGAAAGTCATGGTAACCGTATGGTGGTCTGCCGCAGGTGTTATCCACTATTCCTTCCTGAACCCGGGCGAAACTATCACCAGCGAAAAATACTGCCAGCAGATTGACGAAATGCACCGTAAACTGCAGCGTCTGCAGCCAGCACTGGTGAATCGTAAAGGTCCGATCCTGCTGCATGATAACGCCCGTCCGCACGTTGCCCAACCGACCCTGCAGAAACTGAACGAACTGGGCTATGAAGTTCTGCCACACCCGCCGTACTCCCCGGATCTGTCCCCGACTGACTACCATTTCTTCAAGCATCTGGACAACTTCCTGCAGGGTAAACGTTTTCACAACCAACAGGACGCAGAAAACGCTTTCCAGGAGTTCGTCGAAAGCCGTTCCACTGACTTCTACGCGACCGGTATCAACAAGCTGATCAGCCGTTGGCAGAAATGCGTGGACTGTAACGGCAGCTACTTCGATGGATCCgactacaaggacgacgatgacaagGAAgattataaagatgatgacgataaaAGAgactacaaggacgacgatgacaagTAAAAGCTTGTTCTTCTCAGGCCTGCTGGTAATCGCAGGCCTTTTTATTTGAATTCcgcggatccttctatagtgtcacctaaatgtcgacggccaggcggccgccaggcctacccactagtcaattcgggaggatcgaaacggcagatcgcaaaaaacagtacatacagaaggagacatgaacatgaacatcaaaaaaattgtaaaacaagccacagttctgacttttacgactgcacttctggcaggaggagcgactcaagccttcgcgaaagaaaataaccaaaaagcatacaaagaaacgtacggcgtctctcatattacacgccatgatatgctgcagatccctaaacagcagcaaaacgaaaaataccaagtgcctcaattcgatcaatcaacgattaaaaatattgagtctgcaaaaggacttgatgtgtgggacagctggccgctgcaaaacgctgacggaacagtagctgaatacaacggctatcacgttgtgtttgctcttgcgggaagcccgaaagacgctgatgacacatcaatctacatgttttatcaaaaggtcggcgacaactcaatcgacagctggaaaaacgcgggccgtgtctttaaagacagcgataagttcgacgccaacgatccgatcctgaaagatcagacgcaagaatggtccggttctgcaacctttacatctgacggaaaaatccgtttattctacactgactattccggtaaacattacggcaaacaaagcctgacaacagcgcaggtaaatgtgtcaaaatctgatgacacactcaaaatcaacggagtggaagatcacaaaacgatttttgacggagacggaaaaacatatcagaacgttcagcagtttatcgatgaaggcaattatacatccggcgacaaccatacgctgagagaccctcactacgttgaagacaaaggccataaataccttgtattcgaagccaacacgggaacagaaaacggataccaaggcgaagaatctttatttaacaaagcgtactacggcggcggcacgaacttcttccgtaaagaaagccagaagcttcagcagagcgctaaaaaacgcgatgctgagttagcgaacggcgccctcggtatcatagagttaaataatgattacacattgaaaaaagtaatgaagccgctgatcacttcaaacacggtaactgatgaaatcgagcgcgcgaatgttttcaaaatgaacggcaaatggtacttgttcactgattcacgcggttcaaaaatgacgatcgatggtattaactcaaacgatatttacatgcttggttatgtatcaaactctttaaccggcccttacaagccgctgaacaaaacagggcttgtgctgcaaatgggtcttgatccaaacgatgtgacattcacttactctcacttcgcagtgccgcaagccaaaggcaacaatgtggttatcacaagctacatgacaaacagaggcttcttcgaggataaaaaggcaacatttgcgccaagcttcttaatgaacatcaaaggcaataaaacatccgttgtcaaaaacagcatcctggagcaaggacagctgacagtcaactaataacagcaaaaagaaaatgccgatacttcattggcattttcttttatttctcaacaagatggtgaattgactagtgggtagatccacaggacgggtgtggtcgccatgatcgcgtagtcgatagtggctccaagtagcgaagcgagcaggactgggcggcggccaaagcggtcggacagtgctccgagaacgggtgcgcatagaaattgcatcaacgcatatagcgctagcagcacgccatagtgactggcgatgctgtcggaatggacgatatcccgcaagaggcccggcagtaccggcataaccaagcctatgcctacagcatccagggtgacggtgccgaggatgacgatgagcgcattgttagatttcatacacggtgcctgactgcgttagcaatttaactgtgataaactaccgcattaaagcttatcgatgataagctgtcaaacatgagaattgatccggaacccttaatataacttcgtataatgtatgctatacgaagttattaggtccctcgactatagggtcaccgtcgacagcgacacacttgcatcggatgcagcccggttaacgtgccggcacggcctgggtaaccaggtattttgtccacataaccgtgcgcaaaatgttgtggataagcaggacacagcagcaatccacagcaggcatacaaccgcacaccgaggttactccgttctacaggttacgacgacatgtcaatacttgcccttgacaggcattgatggaatcgtagtctcacgctgatagtctgatcgacaatacaagtgggaccgtggtcccagaccgataatcagaccgacaacacgagtgggatcgtggtcccagactaataatcagaccgacgatacgagtgggaccgtggtcccagactaataatcagaccgacgatacgagtgggaccgtggttccagactaataatcagaccgacgatacgagtgggaccgtggtcccagactaataatcagaccgacgatacgagtgggaccatggtcccagactaataatcagaccgacgatacgagtgggaccgtggtcccagtctgattatcagaccgacgatacgagtgggaccgtggtcccagactaataatcagaccgacgatacgagtgggaccgtggtcccagactaataatcagaccgacgatacgagtgggaccgtggtcccagtctgattatcagaccgacgatacaagtggaacagtgggcccagagagaatattcaggccagttatgctttctggcctgtaacaaaggacattaagtaaagacagataaacgtagactaaaacgtggtcgcatcagggtgctggcttttcaagttccttaagaatggcctcaattttctctatacactcagttggaacacgagacctgtccaggttaagcaccattttatcgcccttatacaatactgtcgctccaggagcaaactgatgtcgtgagcttaaactagttcttgatgcagatgacgttttaagcacagaagttaaaagagtgataacttcttcagcttcaaatatcaccccagcttttttctgctcatgaaggttagatgcctgctgcttaagtaattcctctttatctgtaaaggctttttgaagtgcatcacctgaccgggcagatagttcaccggggtgagaaaaaagagcaacaactgatttaggcaatttggcggtgttgatacagcgggtaataatcttacgtgaaatattttccgcatcagccagcgcagaaatatttccagcaaattcattctgcaatcggcttgcataacgctgaccacgttcataagcacttgttgggcgataatcgttacccaatctggataatgcagccatctgctcatcatccagctcgccaaccagaacacgataatcactttcggtaagtgcagcagctttacgacggcgactcccatcggcaatttctatgacaccagatactcttcgaccgaacgccggtgtctgttgaccagtcagtagaaaagaagggatgagatcatccagtgcgtcctcagtaagcagctcctggtcacgttcattacctgaccatacccgagaggtcttctcaacactatcaccccggagcacttcaagagtaaacttcacatcccgaccacatacaggcaaagtaatggcattaccgcgagccattactcctacgcgcgcaattaacgaatccaccatcggggcagctggtgtcgataacgaagtatcttcaaccggttgagtattgagcgtatgttttggaataacaggcgcacgcttcattatctaatctcccagcgtggtttaatcagacgatcgaaaatttcattgcagacaggttcccaaatagaaagagcatttctccaggcaccagttgaagagcgttgatcaatggcctgttcaaaaacagttctcatccggatctgacctttaccaacttcatccgtttcacgtacaacattttttagaaccatgcttccccaggcatcccgaatttgctcctccatccacggggactgagagccattactattgctgtatttggtaagcaaaatacgtacatcaggctcgaaccctttaagatcaacgttcttgagcagatcacgaagcatatcgaaaaactgcagtgcggaggtgtagtcaaacaactcagcaggcgtgggaacaatcagcacatcagcagcacatacgacattaatcgtgccgatacccaggttaggcgcgctgtcaataactatgacatcatagtcatgagcaacagtttcaatggccagtcggagcatcaggtgtggatcggtgggcagtttaccttcatcaaatttgcccattaactcagtttcaatacggtgcagagccagacaggaaggaataatgtcaagccccggccagcaagtgggctttattgcataagtgacatcgtccttttccccaagatagaaaggcaggagagtgtcttctgcatgaatatgaagatctggtacccatccgtgatacattgaggctgttccctgggggtcgttaccttccacgagcaaaacacgtagccccttcagagccagatcctgagcaagatgaacagaaactgaggttttgtaaacgccacctttatgggcagcaaccccgatcaccggtggaaatacgtcttcagcacgtcgcaatcgcgtaccaaacacatcacgcatatgattaatttgttcaattgtataaccaacacgttgctcaacccgtcctcgaatttccatatccgggtgcggtagtcgccctgctttctcggcatctctgatagcctgagaagaaaccccaactaaatccgctgcttcacctattctccagcgccgggttattttcctcgcttccgggctgtcatcattaaactgtgcaatggcgatagccttcgtcatttcatgaccagcgtttatgcactggttaagtgtttccatgagtttcattctgaacatcctttaatcattgctttgcgtttttttattaaatcttgcaatttactgcaaagcaacaacaaaatcgcaaagtcatcaaaaaaccgcaaagttgtttaaaataagagcaacactacaaaaggagataagaagagcacatacctcagtcacttattatcactagcgctcgccgcagccgtgtaaccgagcatagcgagcgaactggcgaggaagcaaagaagaactgttctgtcagatagctcttacgctcagcgcaagaagaaatatccaccgtgggaaaaactccaggtagaggtacacacgcggatagccaattcagagtaataaactgtgataatcaaccctcatcaatgatgacgaactaacccccgatatcaggtcacatgacgaagggaaagagaaggaaatcaactgtgacaaactgccctcaaatttggcttccttaaaaattacagttcaaaaagtatgagaaaatccatgcaggctgaaggaaacagcaaaactgtgacaaattaccctcagtaggtcagaacaaatgtgacgaaccaccctcaaatctgtgacagataaccctcagactatcctgtcgtcatggaagtgatatcgcggaaggaaaatacgatatgagtcgtctggcggcctttctttttctcaatgtatgagaggcgcattggagttctgctgttgatctcattaacacagacctgcaggaagcggcggcggaagtcaggcatacgctggtaactttgaggcagctggtaacgctctatgatccagtcgattttcagagagacgatgcctgagccatccggcttacgatactgacacagggattcgtataaacgcatggcatacggattggtgatttcttttgtttcactaagccgaaactgcgtaaaccggttctgtaacccgataaagaagggaatgagatatgggttgatatgtacactgtaaagccctctggatggactgtgcgcacgtttgataaaccaaggaaaagattcatagcctttttcatcgccggcatcctcttcagggcgataaaaaaccacttccttccccgcgaaactcttcaatgcctgccgtatatccttactggcttccgcagaggtcaatccgaatatttcagcatatttagcaacatggatctcgcagataccgtcatgttcctgtagggtgccatcagattttctgatctggtcaacgaacagatacagcatacgtttttgatcccgggagagactatatgccgcctcagtgaggtcgtttgactggacgattcgcgggctatttttacgtttcttgtgattgataaccgctgtttccgccatgacagatccatgtgaagtgtgacaagtttttagattgtcacactaaataaaaaagagtcaataagcagggataactttgtgaaaaaacagcttcttctgagggcaatttgtcacagggttaagggcaatttgtcacagACAGGACTGTCATTTGAGGGTGATTTGTCACACTGAAAGGGCAATTTGTCACAACACCTTCTCTAGAACCAGCATGGATAAAGGCCTACAAGGCGCTCTAAAAAAGAAGATCTAAAAACTATAAAAAAAATAATTATAAAAATATCCCCGTGGATAAGTGGATAACCCCAAGGGAAGTTTTTTCAGGCATCGTGTGTAAGCAGAATATATAAGTGCTGTTCCCTGGTGCTTCCTCGCTCACTCGAGGGCTTCGCCCTGTCGCTCAACTGCGGCGAGCACTACTGGCTGTAAaaggacagaccacatcatggttctgtgttcattaggttgttctgtccattgctgacataatccgctccacttcaacgtaacaccgcacgaagatttctattgttcctgaaggcatattcaaatcgttttcgttaccgcttgcaggcatcatgacagaacactacttcctataaacgctacacaggctcctgagattaataatgcggatctctacgataatgggagattttcccgactgtttcgttcgcttctcagtggataacagccagcttctctgtttaacagacaaaaacagcatatccactcagttccacatttccatataaaggccaaggcatttattctcaggataattgtttcagcatcgcaaccgcatcagactccggcatcgcaaactgcacccggtgccgggcagccacatccagcgcaaaaaccttcgtgtagacttccgttgaactgatggacttatgtcccatcaggctttgcagaactttcagcggtataccggcatacagcatgtgcatcgcataggaatggcggaacgtatgtggtgtgaccggaacagagaacgtcacaccgtcagcagcagcggcggcaaccgcctccccaatccaggtcctgaccgttctgtccgtcacttcccagatccgcgctttctctgtccttcctgtgcgacggttacgccgctccatgagcttatcgcgaataaatacctgtgacggaagatcacttcgcagaataaataaatcctggtgtccctgttgataccgggaagccctgggccaacttttggcgaaaatgagacgttgatcggcacgtaagaggttccaactttcaccataatgaaataagatcactaccgggcgtattttttgagttatcgagattttcaggagctaaggaagctaaaatggagaaaaaaatcactggatataccaccgttgatatatcccaatggcatcgtaaagaacattttgaggcatttcagtcagttgctcaatgtacctataaccagaccgttcagctggatattacggcctttttaaagaccgtaaagaaaaataagcacaagttttatccggcctttattcacattcttgcccgcctgatgaatgctcatccggagttccgtatggcaatgaaagacggtgagctggtgatatgggatagtgttcacccttgttacaccgttttccatgagcaaactgaaacgttttcatcgctctggagtgaataccacgacgatttccggcagtttctacacatatattcgcaagatgtggcgtgttacggtgaaaacctggcctatttccctaaagggtttattgagaatatgtttttcgtctcagccaatccctgggtgagtttcaccagttttgatttaaacgtggccaatatggacaacttcttcgcccccgttttcaccatgggcaaatattatacgcaaggcgacaaggtgctgatgccgctggcgattcaggttcatcatgccgtttgtgatggcttccatgtcggcagaatgcttaatgaattacaacagtactgcgatgagtggcagggcggggcgtaatttttttaaggcagttattggtgcccttaaacgcctggttgctacgcctgaataagtgataataagcggatgaatggcagaaattcgatgataagctgtcaaacatgagaattggtcgacggcgcgccaaagcttgcatgcctgcagccgcgtaacctggcaaaatcggttacggttgagtaataaatggatgccctgcgtaagcggggcacatttcattacctctttctccgcacccgacatagataataacttcgtatagtatacattatacgaagttatctagtagacttaattaaggatcgatccggcgcgccaatagtcatgccccgcgcccaccggaaggagctgactgggttgaaggctctcaagggcatcggtcgagcttgacattgtaggactatattgctctaataaatttgcggccgctaatacgactcactatagggagag |
| pRC1822  (Bp2 ++ Hsmar1 Flag) | gatccgcggaattgTGGTTAATAACAGGGGACGTGGTAATCCGTCCCCTTTTTATTTCTGACTGAGTTAATAACAGGCCTGCTTCGGCAGGCCTTTTTATTTCTGACTGAGTTCTTCTCAGGCCTGCTGGTAATCGCAGGCCTTTTTATTTTCTAGaCAATTCCGACGTCTAAGGAAACCATTATCATGACATCAACCTATAAAAATAGGCGTATCACGAGGCCCTCTCGTCTCCACCTCAAGCTCCCTATCTAGTGATAGCGATTGACATCCCTATCAGTGACGGAGATATTGAGCACATCAGCAGGACGCACTGACCACTTTAAGaaggagATATACATATGGAAATGATGCTCGATAAGAAACAGATTCGTGCGATCTTTCTCTTTGAGTTTAAAATGGGTCGCAAAGCGGCGGAGACGACGCGTAATATTAACAACGCGTTCGGTCCTGGCACCGCGAACGAGCGTACCGTGCAATGGTGGTTCAAAAAGTTTCGCAAAGGCGACGAATCTCTGGAGGACGAAGAGCGTTCTGGCCGCCCGTCCGAGGTTGACAACGACCAGCTGCGTGCAATCATCGAAGCTGATCCGCTGACTACCACCCGCGAAGTTGCTGAAGAACTGAATGTGGATCACTCTACTGTGGTTCGCCACCTGAAACAGATCGGTAAAGTAAAAAAACTGGACAAATGGGTTCCTCATGAACTGTCTGAAAACCAGAAAAACCGTCGTTTCGAAGTTAGCTCCTCTCTGATTCTGCGTAACAACAACGAACCGTTCCTGGATCGTATCGTAACCTGTGATGAGAAATGGATTCTGTATGATAACCGTCGCCGCTCTGCTCAGTGGCTGGATCGCGAAGAAGCTCCAAAACACTTCCCGAAACCGAATCTGCACCAGAAGAAAGTCATGGTAACCGTATGGTGGTCTGCCGCAGGTGTTATCCACTATTCCTTCCTGAACCCGGGCGAAACTATCACCAGCGAAAAATACTGCCAGCAGATTGACGAAATGCACCGTAAACTGCAGCGTCTGCAGCCAGCACTGGTGAATCGTAAAGGTCCGATCCTGCTGCATGATAACGCCCGTCCGCACGTTGCCCAACCGACCCTGCAGAAACTGAACGAACTGGGCTATGAAGTTCTGCCACACCCGCCGTACTCCCCGGATCTGTCCCCGACTGACTACCATTTCTTCAAGCATCTGGACAACTTCCTGCAGGGTAAACGTTTTCACAACCAACAGGACGCAGAAAACGCTTTCCAGGAGTTCGTCGAAAGCCGTTCCACTGACTTCTACGCGACCGGTATCAACAAGCTGATCAGCCGTTGGCAGAAATGCGTGGACTGTAACGGCAGCTACTTCGATGGATCCgactacaaggacgacgatgacaagGAAgattataaagatgatgacgataaaAGAgactacaaggacgacgatgacaagTAAAAGCTTGTTCTTCTCAGGCCTGCTGGTAATCGCAGGCCTTTTTATTTGAATTCcgcggatccttctatagtgtcacctaaatgtcgacggccaggcggccgccaggcctacccactagtcaattcgggaggatcgaaacggcagatcgcaaaaaacagtacatacagaaggagacatgaacatgaacatcaaaaaaattgtaaaacaagccacagttctgacttttacgactgcacttctggcaggaggagcgactcaagccttcgcgaaagaaaataaccaaaaagcatacaaagaaacgtacggcgtctctcatattacacgccatgatatgctgcagatccctaaacagcagcaaaacgaaaaataccaagtgcctcaattcgatcaatcaacgattaaaaatattgagtctgcaaaaggacttgatgtgtgggacagctggccgctgcaaaacgctgacggaacagtagctgaatacaacggctatcacgttgtgtttgctcttgcgggaagcccgaaagacgctgatgacacatcaatctacatgttttatcaaaaggtcggcgacaactcaatcgacagctggaaaaacgcgggccgtgtctttaaagacagcgataagttcgacgccaacgatccgatcctgaaagatcagacgcaagaatggtccggttctgcaacctttacatctgacggaaaaatccgtttattctacactgactattccggtaaacattacggcaaacaaagcctgacaacagcgcaggtaaatgtgtcaaaatctgatgacacactcaaaatcaacggagtggaagatcacaaaacgatttttgacggagacggaaaaacatatcagaacgttcagcagtttatcgatgaaggcaattatacatccggcgacaaccatacgctgagagaccctcactacgttgaagacaaaggccataaataccttgtattcgaagccaacacgggaacagaaaacggataccaaggcgaagaatctttatttaacaaagcgtactacggcggcggcacgaacttcttccgtaaagaaagccagaagcttcagcagagcgctaaaaaacgcgatgctgagttagcgaacggcgccctcggtatcatagagttaaataatgattacacattgaaaaaagtaatgaagccgctgatcacttcaaacacggtaactgatgaaatcgagcgcgcgaatgttttcaaaatgaacggcaaatggtacttgttcactgattcacgcggttcaaaaatgacgatcgatggtattaactcaaacgatatttacatgcttggttatgtatcaaactctttaaccggcccttacaagccgctgaacaaaacagggcttgtgctgcaaatgggtcttgatccaaacgatgtgacattcacttactctcacttcgcagtgccgcaagccaaaggcaacaatgtggttatcacaagctacatgacaaacagaggcttcttcgaggataaaaaggcaacatttgcgccaagcttcttaatgaacatcaaaggcaataaaacatccgttgtcaaaaacagcatcctggagcaaggacagctgacagtcaactaataacagcaaaaagaaaatgccgatacttcattggcattttcttttatttctcaacaagatggtgaattgactagtgggtagatccacaggacgggtgtggtcgccatgatcgcgtagtcgatagtggctccaagtagcgaagcgagcaggactgggcggcggccaaagcggtcggacagtgctccgagaacgggtgcgcatagaaattgcatcaacgcatatagcgctagcagcacgccatagtgactggcgatgctgtcggaatggacgatatcccgcaagaggcccggcagtaccggcataaccaagcctatgcctacagcatccagggtgacggtgccgaggatgacgatgagcgcattgttagatttcatacacggtgcctgactgcgttagcaatttaactgtgataaactaccgcattaaagcttatcgatgataagctgtcaaacatgagaattgatccggaacccttaatataacttcgtataatgtatgctatacgaagttattaggtccctcgactatagggtcaccgtcgacagcgacacacttgcatcggatgcagcccggttaacgtgccggcacggcctgggtaaccaggtattttgtccacataaccgtgcgcaaaatgttgtggataagcaggacacagcagcaatccacagcaggcatacaaccgcacaccgaggttactccgttctacaggttacgacgacatgtcaatacttgcccttgacaggcattgatggaatcgtagtctcacgctgatagtctgatcgacaatacaagtgggaccgtggtcccagaccgataatcagaccgacaacacgagtgggatcgtggtcccagactaataatcagaccgacgatacgagtgggaccgtggtcccagactaataatcagaccgacgatacgagtgggaccgtggttccagactaataatcagaccgacgatacgagtgggaccgtggtcccagactaataatcagaccgacgatacgagtgggaccatggtcccagactaataatcagaccgacgatacgagtgggaccgtggtcccagtctgattatcagaccgacgatacgagtgggaccgtggtcccagactaataatcagaccgacgatacgagtgggaccgtggtcccagactaataatcagaccgacgatacgagtgggaccgtggtcccagtctgattatcagaccgacgatacaagtggaacagtgggcccagagagaatattcaggccagttatgctttctggcctgtaacaaaggacattaagtaaagacagataaacgtagactaaaacgtggtcgcatcagggtgctggcttttcaagttccttaagaatggcctcaattttctctatacactcagttggaacacgagacctgtccaggttaagcaccattttatcgcccttatacaatactgtcgctccaggagcaaactgatgtcgtgagcttaaactagttcttgatgcagatgacgttttaagcacagaagttaaaagagtgataacttcttcagcttcaaatatcaccccagcttttttctgctcatgaaggttagatgcctgctgcttaagtaattcctctttatctgtaaaggctttttgaagtgcatcacctgaccgggcagatagttcaccggggtgagaaaaaagagcaacaactgatttaggcaatttggcggtgttgatacagcgggtaataatcttacgtgaaatattttccgcatcagccagcgcagaaatatttccagcaaattcattctgcaatcggcttgcataacgctgaccacgttcataagcacttgttgggcgataatcgttacccaatctggataatgcagccatctgctcatcatccagctcgccaaccagaacacgataatcactttcggtaagtgcagcagctttacgacggcgactcccatcggcaatttctatgacaccagatactcttcgaccgaacgccggtgtctgttgaccagtcagtagaaaagaagggatgagatcatccagtgcgtcctcagtaagcagctcctggtcacgttcattacctgaccatacccgagaggtcttctcaacactatcaccccggagcacttcaagagtaaacttcacatcccgaccacatacaggcaaagtaatggcattaccgcgagccattactcctacgcgcgcaattaacgaatccaccatcggggcagctggtgtcgataacgaagtatcttcaaccggttgagtattgagcgtatgttttggaataacaggcgcacgcttcattatctaatctcccagcgtggtttaatcagacgatcgaaaatttcattgcagacaggttcccaaatagaaagagcatttctccaggcaccagttgaagagcgttgatcaatggcctgttcaaaaacagttctcatccggatctgacctttaccaacttcatccgtttcacgtacaacattttttagaaccatgcttccccaggcatcccgaatttgctcctccatccacggggactgagagccattactattgctgtatttggtaagcaaaatacgtacatcaggctcgaaccctttaagatcaacgttcttgagcagatcacgaagcatatcgaaaaactgcagtgcggaggtgtagtcaaacaactcagcaggcgtgggaacaatcagcacatcagcagcacatacgacattaatcgtgccgatacccaggttaggcgcgctgtcaataactatgacatcatagtcatgagcaacagtttcaatggccagtcggagcatcaggtgtggatcggtgggcagtttaccttcatcaaatttgcccattaactcagtttcaatacggtgcagagccagacaggaaggaataatgtcaagccccggccagcaagtgggctttattgcataagtgacatcgtccttttccccaagatagaaaggcaggagagtgtcttctgcatgaatatgaagatctggtacccatccgtgatacattgaggctgttccctgggggtcgttaccttccacgagcaaaacacgtagccccttcagagccagatcctgagcaagatgaacagaaactgaggttttgtaaacgccacctttatgggcagcaaccccgatcaccggtggaaatacgtcttcagcacgtcgcaatcgcgtaccaaacacatcacgcatatgattaatttgttcaattgtataaccaacacgttgctcaacccgtcctcgaatttccatatccgggtgcggtagtcgccctgctttctcggcatctctgatagcctgagaagaaaccccaactaaatccgctgcttcacctattctccagcgccgggttattttcctcgcttccgggctgtcatcattaaactgtgcaatggcgatagccttcgtcatttcatgaccagcgtttatgcactggttaagtgtttccatgagtttcattctgaacatcctttaatcattgctttgcgtttttttattaaatcttgcaatttactgcaaagcaacaacaaaatcgcaaagtcatcaaaaaaccgcaaagttgtttaaaataagagcaacactacaaaaggagataagaagagcacatacctcagtcacttattatcactagcgctcgccgcagccgtgtaaccgagcatagcgagcgaactggcgaggaagcaaagaagaactgttctgtcagatagctcttacgctcagcgcaagaagaaatatccaccgtgggaaaaactccaggtagaggtacacacgcggatagccaattcagagtaataaactgtgataatcaaccctcatcaatgatgacgaactaacccccgatatcaggtcacatgacgaagggaaagagaaggaaatcaactgtgacaaactgccctcaaatttggcttccttaaaaattacagttcaaaaagtatgagaaaatccatgcaggctgaaggaaacagcaaaactgtgacaaattaccctcagtaggtcagaacaaatgtgacgaaccaccctcaaatctgtgacagataaccctcagactatcctgtcgtcatggaagtgatatcgcggaaggaaaatacgatatgagtcgtctggcggcctttctttttctcaatgtatgagaggcgcattggagttctgctgttgatctcattaacacagacctgcaggaagcggcggcggaagtcaggcatacgctggtaactttgaggcagctggtaacgctctatgatccagtcgattttcagagagacgatgcctgagccatccggcttacgatactgacacagggattcgtataaacgcatggcatacggattggtgatttcttttgtttcactaagccgaaactgcgtaaaccggttctgtaacccgataaagaagggaatgagatatgggttgatatgtacactgtaaagccctctggatggactgtgcgcacgtttgataaaccaaggaaaagattcatagcctttttcatcgccggcatcctcttcagggcgataaaaaaccacttccttccccgcgaaactcttcaatgcctgccgtatatccttactggcttccgcagaggtcaatccgaatatttcagcatatttagcaacatggatctcgcagataccgtcatgttcctgtagggtgccatcagattttctgatctggtcaacgaacagatacagcatacgtttttgatcccgggagagactatatgccgcctcagtgaggtcgtttgactggacgattcgcgggctatttttacgtttcttgtgattgataaccgctgtttccgccatgacagatccatgtgaagtgtgacaagtttttagattgtcacactaaataaaaaagagtcaataagcagggataactttgtgaaaaaacagcttcttctgagggcaatttgtcacagggttaagggcaatttgtcacagACAGGACTGTCATTTGAGGGTGATTTGTCACACTGAAAGGGCAATTTGTCACAACACCTTCTCTAGAACCAGCATGGATAAAGGCCTACAAGGCGCTCTAAAAAAGAAGATCTAAAAACTATAAAAAAAATAATTATAAAAATATCCCCGTGGATAAGTGGATAACCCCAAGGGAAGTTTTTTCAGGCATCGTGTGTAAGCAGAATATATAAGTGCTGTTCCCTGGTGCTTCCTCGCTCACTCGAGGGCTTCGCCCTGTCGCTCAACTGCGGCGAGCACTACTGGCTGTAAaaggacagaccacatcatggttctgtgttcattaggttgttctgtccattgctgacataatccgctccacttcaacgtaacaccgcacgaagatttctattgttcctgaaggcatattcaaatcgttttcgttaccgcttgcaggcatcatgacagaacactacttcctataaacgctacacaggctcctgagattaataatgcggatctctacgataatgggagattttcccgactgtttcgttcgcttctcagtggataacagccagcttctctgtttaacagacaaaaacagcatatccactcagttccacatttccatataaaggccaaggcatttattctcaggataattgtttcagcatcgcaaccgcatcagactccggcatcgcaaactgcacccggtgccgggcagccacatccagcgcaaaaaccttcgtgtagacttccgttgaactgatggacttatgtcccatcaggctttgcagaactttcagcggtataccggcatacagcatgtgcatcgcataggaatggcggaacgtatgtggtgtgaccggaacagagaacgtcacaccgtcagcagcagcggcggcaaccgcctccccaatccaggtcctgaccgttctgtccgtcacttcccagatccgcgctttctctgtccttcctgtgcgacggttacgccgctccatgagcttatcgcgaataaatacctgtgacggaagatcacttcgcagaataaataaatcctggtgtccctgttgataccgggaagccctgggccaacttttggcgaaaatgagacgttgatcggcacgtaagaggttccaactttcaccataatgaaataagatcactaccgggcgtattttttgagttatcgagattttcaggagctaaggaagctaaaatggagaaaaaaatcactggatataccaccgttgatatatcccaatggcatcgtaaagaacattttgaggcatttcagtcagttgctcaatgtacctataaccagaccgttcagctggatattacggcctttttaaagaccgtaaagaaaaataagcacaagttttatccggcctttattcacattcttgcccgcctgatgaatgctcatccggagttccgtatggcaatgaaagacggtgagctggtgatatgggatagtgttcacccttgttacaccgttttccatgagcaaactgaaacgttttcatcgctctggagtgaataccacgacgatttccggcagtttctacacatatattcgcaagatgtggcgtgttacggtgaaaacctggcctatttccctaaagggtttattgagaatatgtttttcgtctcagccaatccctgggtgagtttcaccagttttgatttaaacgtggccaatatggacaacttcttcgcccccgttttcaccatgggcaaatattatacgcaaggcgacaaggtgctgatgccgctggcgattcaggttcatcatgccgtttgtgatggcttccatgtcggcagaatgcttaatgaattacaacagtactgcgatgagtggcagggcggggcgtaatttttttaaggcagttattggtgcccttaaacgcctggttgctacgcctgaataagtgataataagcggatgaatggcagaaattcgatgataagctgtcaaacatgagaattggtcgacggcgcgccaaagcttgcatgcctgcagccgcgtaacctggcaaaatcggttacggttgagtaataaatggatgccctgcgtaagcggggcacatttcattacctctttctccgcacccgacatagataataacttcgtatagtatacattatacgaagttatctagtagacttaattaaggatcgatccggcgcgccaatagtcatgccccgcgcccaccggaaggagctgactgggttgaaggctctcaagggcatcggtcgagcttgacattgtaggactatattgctctaataaatttgcggccgctaatacgactcactatagggagag |
| pRC1823  (Bp3 ++ Hsmar1 Flag) | gatccgcggaattgTGGTTAATAACAGGGGACGTGGTAATCCGTCCCCTTTTTATTTCTGACTGAGTTAATAACAGGCCTGCTTCGGCAGGCCTTTTTATTTCTGACTGAGTTCTTCTCAGGCCTGCTGGTAATCGCAGGCCTTTTTATTTTCTAGaCAATTCCGACGTCTAAGAAACCATTATTATCATGACATTAACCTATAAAAATAGGCGTATCACGAGGCCCTTTCGTCTTCACCTCGAGTCCCTATCAGTGATAGAGATTGACCTCCCTATCAGTGATAGAGATACTGAGCACATCAGCAGGACGCACTGACCACTTTAAGAAGGAGATATACATATGGAAATGATGCTCGATAAGAAACAGATTCGTGCGATCTTTCTCTTTGAGTTTAAAATGGGTCGCAAAGCGGCGGAGACGACGCGTAATATTAACAACGCGTTCGGTCCTGGCACCGCGAACGAGCGTACCGTGCAATGGTGGTTCAAAAAGTTTCGCAAAGGCGACGAATCTCTGGAGGACGAAGAGCGTTCTGGCCGCCCGTCCGAGGTTGACAACGACCAGCTGCGTGCAATCATCGAAGCTGATCCGCTGACTACCACCCGCGAAGTTGCTGAAGAACTGAATGTGGATCACTCTACTGTGGTTCGCCACCTGAAACAGATCGGTAAAGTAAAAAAACTGGACAAATGGGTTCCTCATGAACTGTCTGAAAACCAGAAAAACCGTCGTTTCGAAGTTAGCTCCTCTCTGATTCTGCGTAACAACAACGAACCGTTCCTGGATCGTATCGTAACCTGTGATGAGAAATGGATTCTGTATGATAACCGTCGCCGCTCTGCTCAGTGGCTGGATCGCGAAGAAGCTCCAAAACACTTCCCGAAACCGAATCTGCACCAGAAGAAAGTCATGGTAACCGTATGGTGGTCTGCCGCAGGTGTTATCCACTATTCCTTCCTGAACCCGGGCGAAACTATCACCAGCGAAAAATACTGCCAGCAGATTGACGAAATGCACCGTAAACTGCAGCGTCTGCAGCCAGCACTGGTGAATCGTAAAGGTCCGATCCTGCTGCATGATAACGCCCGTCCGCACGTTGCCCAACCGACCCTGCAGAAACTGAACGAACTGGGCTATGAAGTTCTGCCACACCCGCCGTACTCCCCGGATCTGTCCCCGACTGACTACCATTTCTTCAAGCATCTGGACAACTTCCTGCAGGGTAAACGTTTTCACAACCAACAGGACGCAGAAAACGCTTTCCAGGAGTTCGTCGAAAGCCGTTCCACTGACTTCTACGCGACCGGTATCAACAAGCTGATCAGCCGTTGGCAGAAATGCGTGGACTGTAACGGCAGCTACTTCGATGGATCCgactacaaggacgacgatgacaagGAAgattataaagatgatgacgataaaAGAgactacaaggacgacgatgacaagTAAAAGCTTGTTCTTCTCAGGCCTGCTGGTAATCGCAGGCCTTTTTATTTGAATTCcgcggatccttctatagtgtcacctaaatgtcgacggccaggcggccgccaggcctacccactagtcaattcgggaggatcgaaacggcagatcgcaaaaaacagtacatacagaaggagacatgaacatgaacatcaaaaaaattgtaaaacaagccacagttctgacttttacgactgcacttctggcaggaggagcgactcaagccttcgcgaaagaaaataaccaaaaagcatacaaagaaacgtacggcgtctctcatattacacgccatgatatgctgcagatccctaaacagcagcaaaacgaaaaataccaagtgcctcaattcgatcaatcaacgattaaaaatattgagtctgcaaaaggacttgatgtgtgggacagctggccgctgcaaaacgctgacggaacagtagctgaatacaacggctatcacgttgtgtttgctcttgcgggaagcccgaaagacgctgatgacacatcaatctacatgttttatcaaaaggtcggcgacaactcaatcgacagctggaaaaacgcgggccgtgtctttaaagacagcgataagttcgacgccaacgatccgatcctgaaagatcagacgcaagaatggtccggttctgcaacctttacatctgacggaaaaatccgtttattctacactgactattccggtaaacattacggcaaacaaagcctgacaacagcgcaggtaaatgtgtcaaaatctgatgacacactcaaaatcaacggagtggaagatcacaaaacgatttttgacggagacggaaaaacatatcagaacgttcagcagtttatcgatgaaggcaattatacatccggcgacaaccatacgctgagagaccctcactacgttgaagacaaaggccataaataccttgtattcgaagccaacacgggaacagaaaacggataccaaggcgaagaatctttatttaacaaagcgtactacggcggcggcacgaacttcttccgtaaagaaagccagaagcttcagcagagcgctaaaaaacgcgatgctgagttagcgaacggcgccctcggtatcatagagttaaataatgattacacattgaaaaaagtaatgaagccgctgatcacttcaaacacggtaactgatgaaatcgagcgcgcgaatgttttcaaaatgaacggcaaatggtacttgttcactgattcacgcggttcaaaaatgacgatcgatggtattaactcaaacgatatttacatgcttggttatgtatcaaactctttaaccggcccttacaagccgctgaacaaaacagggcttgtgctgcaaatgggtcttgatccaaacgatgtgacattcacttactctcacttcgcagtgccgcaagccaaaggcaacaatgtggttatcacaagctacatgacaaacagaggcttcttcgaggataaaaaggcaacatttgcgccaagcttcttaatgaacatcaaaggcaataaaacatccgttgtcaaaaacagcatcctggagcaaggacagctgacagtcaactaataacagcaaaaagaaaatgccgatacttcattggcattttcttttatttctcaacaagatggtgaattgactagtgggtagatccacaggacgggtgtggtcgccatgatcgcgtagtcgatagtggctccaagtagcgaagcgagcaggactgggcggcggccaaagcggtcggacagtgctccgagaacgggtgcgcatagaaattgcatcaacgcatatagcgctagcagcacgccatagtgactggcgatgctgtcggaatggacgatatcccgcaagaggcccggcagtaccggcataaccaagcctatgcctacagcatccagggtgacggtgccgaggatgacgatgagcgcattgttagatttcatacacggtgcctgactgcgttagcaatttaactgtgataaactaccgcattaaagcttatcgatgataagctgtcaaacatgagaattgatccggaacccttaatataacttcgtataatgtatgctatacgaagttattaggtccctcgactatagggtcaccgtcgacagcgacacacttgcatcggatgcagcccggttaacgtgccggcacggcctgggtaaccaggtattttgtccacataaccgtgcgcaaaatgttgtggataagcaggacacagcagcaatccacagcaggcatacaaccgcacaccgaggttactccgttctacaggttacgacgacatgtcaatacttgcccttgacaggcattgatggaatcgtagtctcacgctgatagtctgatcgacaatacaagtgggaccgtggtcccagaccgataatcagaccgacaacacgagtgggatcgtggtcccagactaataatcagaccgacgatacgagtgggaccgtggtcccagactaataatcagaccgacgatacgagtgggaccgtggttccagactaataatcagaccgacgatacgagtgggaccgtggtcccagactaataatcagaccgacgatacgagtgggaccatggtcccagactaataatcagaccgacgatacgagtgggaccgtggtcccagtctgattatcagaccgacgatacgagtgggaccgtggtcccagactaataatcagaccgacgatacgagtgggaccgtggtcccagactaataatcagaccgacgatacgagtgggaccgtggtcccagtctgattatcagaccgacgatacaagtggaacagtgggcccagagagaatattcaggccagttatgctttctggcctgtaacaaaggacattaagtaaagacagataaacgtagactaaaacgtggtcgcatcagggtgctggcttttcaagttccttaagaatggcctcaattttctctatacactcagttggaacacgagacctgtccaggttaagcaccattttatcgcccttatacaatactgtcgctccaggagcaaactgatgtcgtgagcttaaactagttcttgatgcagatgacgttttaagcacagaagttaaaagagtgataacttcttcagcttcaaatatcaccccagcttttttctgctcatgaaggttagatgcctgctgcttaagtaattcctctttatctgtaaaggctttttgaagtgcatcacctgaccgggcagatagttcaccggggtgagaaaaaagagcaacaactgatttaggcaatttggcggtgttgatacagcgggtaataatcttacgtgaaatattttccgcatcagccagcgcagaaatatttccagcaaattcattctgcaatcggcttgcataacgctgaccacgttcataagcacttgttgggcgataatcgttacccaatctggataatgcagccatctgctcatcatccagctcgccaaccagaacacgataatcactttcggtaagtgcagcagctttacgacggcgactcccatcggcaatttctatgacaccagatactcttcgaccgaacgccggtgtctgttgaccagtcagtagaaaagaagggatgagatcatccagtgcgtcctcagtaagcagctcctggtcacgttcattacctgaccatacccgagaggtcttctcaacactatcaccccggagcacttcaagagtaaacttcacatcccgaccacatacaggcaaagtaatggcattaccgcgagccattactcctacgcgcgcaattaacgaatccaccatcggggcagctggtgtcgataacgaagtatcttcaaccggttgagtattgagcgtatgttttggaataacaggcgcacgcttcattatctaatctcccagcgtggtttaatcagacgatcgaaaatttcattgcagacaggttcccaaatagaaagagcatttctccaggcaccagttgaagagcgttgatcaatggcctgttcaaaaacagttctcatccggatctgacctttaccaacttcatccgtttcacgtacaacattttttagaaccatgcttccccaggcatcccgaatttgctcctccatccacggggactgagagccattactattgctgtatttggtaagcaaaatacgtacatcaggctcgaaccctttaagatcaacgttcttgagcagatcacgaagcatatcgaaaaactgcagtgcggaggtgtagtcaaacaactcagcaggcgtgggaacaatcagcacatcagcagcacatacgacattaatcgtgccgatacccaggttaggcgcgctgtcaataactatgacatcatagtcatgagcaacagtttcaatggccagtcggagcatcaggtgtggatcggtgggcagtttaccttcatcaaatttgcccattaactcagtttcaatacggtgcagagccagacaggaaggaataatgtcaagccccggccagcaagtgggctttattgcataagtgacatcgtccttttccccaagatagaaaggcaggagagtgtcttctgcatgaatatgaagatctggtacccatccgtgatacattgaggctgttccctgggggtcgttaccttccacgagcaaaacacgtagccccttcagagccagatcctgagcaagatgaacagaaactgaggttttgtaaacgccacctttatgggcagcaaccccgatcaccggtggaaatacgtcttcagcacgtcgcaatcgcgtaccaaacacatcacgcatatgattaatttgttcaattgtataaccaacacgttgctcaacccgtcctcgaatttccatatccgggtgcggtagtcgccctgctttctcggcatctctgatagcctgagaagaaaccccaactaaatccgctgcttcacctattctccagcgccgggttattttcctcgcttccgggctgtcatcattaaactgtgcaatggcgatagccttcgtcatttcatgaccagcgtttatgcactggttaagtgtttccatgagtttcattctgaacatcctttaatcattgctttgcgtttttttattaaatcttgcaatttactgcaaagcaacaacaaaatcgcaaagtcatcaaaaaaccgcaaagttgtttaaaataagagcaacactacaaaaggagataagaagagcacatacctcagtcacttattatcactagcgctcgccgcagccgtgtaaccgagcatagcgagcgaactggcgaggaagcaaagaagaactgttctgtcagatagctcttacgctcagcgcaagaagaaatatccaccgtgggaaaaactccaggtagaggtacacacgcggatagccaattcagagtaataaactgtgataatcaaccctcatcaatgatgacgaactaacccccgatatcaggtcacatgacgaagggaaagagaaggaaatcaactgtgacaaactgccctcaaatttggcttccttaaaaattacagttcaaaaagtatgagaaaatccatgcaggctgaaggaaacagcaaaactgtgacaaattaccctcagtaggtcagaacaaatgtgacgaaccaccctcaaatctgtgacagataaccctcagactatcctgtcgtcatggaagtgatatcgcggaaggaaaatacgatatgagtcgtctggcggcctttctttttctcaatgtatgagaggcgcattggagttctgctgttgatctcattaacacagacctgcaggaagcggcggcggaagtcaggcatacgctggtaactttgaggcagctggtaacgctctatgatccagtcgattttcagagagacgatgcctgagccatccggcttacgatactgacacagggattcgtataaacgcatggcatacggattggtgatttcttttgtttcactaagccgaaactgcgtaaaccggttctgtaacccgataaagaagggaatgagatatgggttgatatgtacactgtaaagccctctggatggactgtgcgcacgtttgataaaccaaggaaaagattcatagcctttttcatcgccggcatcctcttcagggcgataaaaaaccacttccttccccgcgaaactcttcaatgcctgccgtatatccttactggcttccgcagaggtcaatccgaatatttcagcatatttagcaacatggatctcgcagataccgtcatgttcctgtagggtgccatcagattttctgatctggtcaacgaacagatacagcatacgtttttgatcccgggagagactatatgccgcctcagtgaggtcgtttgactggacgattcgcgggctatttttacgtttcttgtgattgataaccgctgtttccgccatgacagatccatgtgaagtgtgacaagtttttagattgtcacactaaataaaaaagagtcaataagcagggataactttgtgaaaaaacagcttcttctgagggcaatttgtcacagggttaagggcaatttgtcacagACAGGACTGTCATTTGAGGGTGATTTGTCACACTGAAAGGGCAATTTGTCACAACACCTTCTCTAGAACCAGCATGGATAAAGGCCTACAAGGCGCTCTAAAAAAGAAGATCTAAAAACTATAAAAAAAATAATTATAAAAATATCCCCGTGGATAAGTGGATAACCCCAAGGGAAGTTTTTTCAGGCATCGTGTGTAAGCAGAATATATAAGTGCTGTTCCCTGGTGCTTCCTCGCTCACTCGAGGGCTTCGCCCTGTCGCTCAACTGCGGCGAGCACTACTGGCTGTAAaaggacagaccacatcatggttctgtgttcattaggttgttctgtccattgctgacataatccgctccacttcaacgtaacaccgcacgaagatttctattgttcctgaaggcatattcaaatcgttttcgttaccgcttgcaggcatcatgacagaacactacttcctataaacgctacacaggctcctgagattaataatgcggatctctacgataatgggagattttcccgactgtttcgttcgcttctcagtggataacagccagcttctctgtttaacagacaaaaacagcatatccactcagttccacatttccatataaaggccaaggcatttattctcaggataattgtttcagcatcgcaaccgcatcagactccggcatcgcaaactgcacccggtgccgggcagccacatccagcgcaaaaaccttcgtgtagacttccgttgaactgatggacttatgtcccatcaggctttgcagaactttcagcggtataccggcatacagcatgtgcatcgcataggaatggcggaacgtatgtggtgtgaccggaacagagaacgtcacaccgtcagcagcagcggcggcaaccgcctccccaatccaggtcctgaccgttctgtccgtcacttcccagatccgcgctttctctgtccttcctgtgcgacggttacgccgctccatgagcttatcgcgaataaatacctgtgacggaagatcacttcgcagaataaataaatcctggtgtccctgttgataccgggaagccctgggccaacttttggcgaaaatgagacgttgatcggcacgtaagaggttccaactttcaccataatgaaataagatcactaccgggcgtattttttgagttatcgagattttcaggagctaaggaagctaaaatggagaaaaaaatcactggatataccaccgttgatatatcccaatggcatcgtaaagaacattttgaggcatttcagtcagttgctcaatgtacctataaccagaccgttcagctggatattacggcctttttaaagaccgtaaagaaaaataagcacaagttttatccggcctttattcacattcttgcccgcctgatgaatgctcatccggagttccgtatggcaatgaaagacggtgagctggtgatatgggatagtgttcacccttgttacaccgttttccatgagcaaactgaaacgttttcatcgctctggagtgaataccacgacgatttccggcagtttctacacatatattcgcaagatgtggcgtgttacggtgaaaacctggcctatttccctaaagggtttattgagaatatgtttttcgtctcagccaatccctgggtgagtttcaccagttttgatttaaacgtggccaatatggacaacttcttcgcccccgttttcaccatgggcaaatattatacgcaaggcgacaaggtgctgatgccgctggcgattcaggttcatcatgccgtttgtgatggcttccatgtcggcagaatgcttaatgaattacaacagtactgcgatgagtggcagggcggggcgtaatttttttaaggcagttattggtgcccttaaacgcctggttgctacgcctgaataagtgataataagcggatgaatggcagaaattcgatgataagctgtcaaacatgagaattggtcgacggcgcgccaaagcttgcatgcctgcagccgcgtaacctggcaaaatcggttacggttgagtaataaatggatgccctgcgtaagcggggcacatttcattacctctttctccgcacccgacatagataataacttcgtatagtatacattatacgaagttatctagtagacttaattaaggatcgatccggcgcgccaatagtcatgccccgcgcccaccggaaggagctgactgggttgaaggctctcaagggcatcggtcgagcttgacattgtaggactatattgctctaataaatttgcggccgctaatacgactcactatagggagag |
| pRC1824  (Bp4 ++ Hsmar1 Flag) | gatccgcggaattgTGGTTAATAACAGGGGACGTGGTAATCCGTCCCCTTTTTATTTCTGACTGAGTTAATAACAGGCCTGCTTCGGCAGGCCTTTTTATTTCTGACTGAGTTCTTCTCAGGCCTGCTGGTAATCGCAGGCCTTTTTATTTTCTAGgCAATTCCGACGTCTAAGAAACCATTATTATCATGACATTAACCTATAAAAATAGGCGTATCACGAGGCCCTCTCGTCTTCACCTCGAGTCCCTATCAGTGATAGGGATTGACATCCCTATCAGTGATAGAGACACTGGGCACATCAGCAGGACGCACTGACCACTTTAAGAAGGAGATATACATATGGAAATGATGCTCGATAAGAAACAGATTCGTGCGATCTTTCTCTTTGAGTTTAAAATGGGTCGCAAAGCGGCGGAGACGACGCGTAATATTAACAACGCGTTCGGTCCTGGCACCGCGAACGAGCGTACCGTGCAATGGTGGTTCAAAAAGTTTCGCAAAGGCGACGAATCTCTGGAGGACGAAGAGCGTTCTGGCCGCCCGTCCGAGGTTGACAACGACCAGCTGCGTGCAATCATCGAAGCTGATCCGCTGACTACCACCCGCGAAGTTGCTGAAGAACTGAATGTGGATCACTCTACTGTGGTTCGCCACCTGAAACAGATCGGTAAAGTAAAAAAACTGGACAAATGGGTTCCTCATGAACTGTCTGAAAACCAGAAAAACCGTCGTTTCGAAGTTAGCTCCTCTCTGATTCTGCGTAACAACAACGAACCGTTCCTGGATCGTATCGTAACCTGTGATGAGAAATGGATTCTGTATGATAACCGTCGCCGCTCTGCTCAGTGGCTGGATCGCGAAGAAGCTCCAAAACACTTCCCGAAACCGAATCTGCACCAGAAGAAAGTCATGGTAACCGTATGGTGGTCTGCCGCAGGTGTTATCCACTATTCCTTCCTGAACCCGGGCGAAACTATCACCAGCGAAAAATACTGCCAGCAGATTGACGAAATGCACCGTAAACTGCAGCGTCTGCAGCCAGCACTGGTGAATCGTAAAGGTCCGATCCTGCTGCATGATAACGCCCGTCCGCACGTTGCCCAACCGACCCTGCAGAAACTGAACGAACTGGGCTATGAAGTTCTGCCACACCCGCCGTACTCCCCGGATCTGTCCCCGACTGACTACCATTTCTTCAAGCATCTGGACAACTTCCTGCAGGGTAAACGTTTTCACAACCAACAGGACGCAGAAAACGCTTTCCAGGAGTTCGTCGAAAGCCGTTCCACTGACTTCTACGCGACCGGTATCAACAAGCTGATCAGCCGTTGGCAGAAATGCGTGGACTGTAACGGCAGCTACTTCGATGGATCCgactacaaggacgacgatgacaagGAAgattataaagatgatgacgataaaAGAgactacaaggacgacgatgacaagTAAAAGCTTGTTCTTCTCAGGCCTGCTGGTAATCGCAGGCCTTTTTATTTGAATTCcgcggatccttctatagtgtcacctaaatgtcgacggccaggcggccgccaggcctacccactagtcaattcgggaggatcgaaacggcagatcgcaaaaaacagtacatacagaaggagacatgaacatgaacatcaaaaaaattgtaaaacaagccacagttctgacttttacgactgcacttctggcaggaggagcgactcaagccttcgcgaaagaaaataaccaaaaagcatacaaagaaacgtacggcgtctctcatattacacgccatgatatgctgcagatccctaaacagcagcaaaacgaaaaataccaagtgcctcaattcgatcaatcaacgattaaaaatattgagtctgcaaaaggacttgatgtgtgggacagctggccgctgcaaaacgctgacggaacagtagctgaatacaacggctatcacgttgtgtttgctcttgcgggaagcccgaaagacgctgatgacacatcaatctacatgttttatcaaaaggtcggcgacaactcaatcgacagctggaaaaacgcgggccgtgtctttaaagacagcgataagttcgacgccaacgatccgatcctgaaagatcagacgcaagaatggtccggttctgcaacctttacatctgacggaaaaatccgtttattctacactgactattccggtaaacattacggcaaacaaagcctgacaacagcgcaggtaaatgtgtcaaaatctgatgacacactcaaaatcaacggagtggaagatcacaaaacgatttttgacggagacggaaaaacatatcagaacgttcagcagtttatcgatgaaggcaattatacatccggcgacaaccatacgctgagagaccctcactacgttgaagacaaaggccataaataccttgtattcgaagccaacacgggaacagaaaacggataccaaggcgaagaatctttatttaacaaagcgtactacggcggcggcacgaacttcttccgtaaagaaagccagaagcttcagcagagcgctaaaaaacgcgatgctgagttagcgaacggcgccctcggtatcatagagttaaataatgattacacattgaaaaaagtaatgaagccgctgatcacttcaaacacggtaactgatgaaatcgagcgcgcgaatgttttcaaaatgaacggcaaatggtacttgttcactgattcacgcggttcaaaaatgacgatcgatggtattaactcaaacgatatttacatgcttggttatgtatcaaactctttaaccggcccttacaagccgctgaacaaaacagggcttgtgctgcaaatgggtcttgatccaaacgatgtgacattcacttactctcacttcgcagtgccgcaagccaaaggcaacaatgtggttatcacaagctacatgacaaacagaggcttcttcgaggataaaaaggcaacatttgcgccaagcttcttaatgaacatcaaaggcaataaaacatccgttgtcaaaaacagcatcctggagcaaggacagctgacagtcaactaataacagcaaaaagaaaatgccgatacttcattggcattttcttttatttctcaacaagatggtgaattgactagtgggtagatccacaggacgggtgtggtcgccatgatcgcgtagtcgatagtggctccaagtagcgaagcgagcaggactgggcggcggccaaagcggtcggacagtgctccgagaacgggtgcgcatagaaattgcatcaacgcatatagcgctagcagcacgccatagtgactggcgatgctgtcggaatggacgatatcccgcaagaggcccggcagtaccggcataaccaagcctatgcctacagcatccagggtgacggtgccgaggatgacgatgagcgcattgttagatttcatacacggtgcctgactgcgttagcaatttaactgtgataaactaccgcattaaagcttatcgatgataagctgtcaaacatgagaattgatccggaacccttaatataacttcgtataatgtatgctatacgaagttattaggtccctcgactatagggtcaccgtcgacagcgacacacttgcatcggatgcagcccggttaacgtgccggcacggcctgggtaaccaggtattttgtccacataaccgtgcgcaaaatgttgtggataagcaggacacagcagcaatccacagcaggcatacaaccgcacaccgaggttactccgttctacaggttacgacgacatgtcaatacttgcccttgacaggcattgatggaatcgtagtctcacgctgatagtctgatcgacaatacaagtgggaccgtggtcccagaccgataatcagaccgacaacacgagtgggatcgtggtcccagactaataatcagaccgacgatacgagtgggaccgtggtcccagactaataatcagaccgacgatacgagtgggaccgtggttccagactaataatcagaccgacgatacgagtgggaccgtggtcccagactaataatcagaccgacgatacgagtgggaccatggtcccagactaataatcagaccgacgatacgagtgggaccgtggtcccagtctgattatcagaccgacgatacgagtgggaccgtggtcccagactaataatcagaccgacgatacgagtgggaccgtggtcccagactaataatcagaccgacgatacgagtgggaccgtggtcccagtctgattatcagaccgacgatacaagtggaacagtgggcccagagagaatattcaggccagttatgctttctggcctgtaacaaaggacattaagtaaagacagataaacgtagactaaaacgtggtcgcatcagggtgctggcttttcaagttccttaagaatggcctcaattttctctatacactcagttggaacacgagacctgtccaggttaagcaccattttatcgcccttatacaatactgtcgctccaggagcaaactgatgtcgtgagcttaaactagttcttgatgcagatgacgttttaagcacagaagttaaaagagtgataacttcttcagcttcaaatatcaccccagcttttttctgctcatgaaggttagatgcctgctgcttaagtaattcctctttatctgtaaaggctttttgaagtgcatcacctgaccgggcagatagttcaccggggtgagaaaaaagagcaacaactgatttaggcaatttggcggtgttgatacagcgggtaataatcttacgtgaaatattttccgcatcagccagcgcagaaatatttccagcaaattcattctgcaatcggcttgcataacgctgaccacgttcataagcacttgttgggcgataatcgttacccaatctggataatgcagccatctgctcatcatccagctcgccaaccagaacacgataatcactttcggtaagtgcagcagctttacgacggcgactcccatcggcaatttctatgacaccagatactcttcgaccgaacgccggtgtctgttgaccagtcagtagaaaagaagggatgagatcatccagtgcgtcctcagtaagcagctcctggtcacgttcattacctgaccatacccgagaggtcttctcaacactatcaccccggagcacttcaagagtaaacttcacatcccgaccacatacaggcaaagtaatggcattaccgcgagccattactcctacgcgcgcaattaacgaatccaccatcggggcagctggtgtcgataacgaagtatcttcaaccggttgagtattgagcgtatgttttggaataacaggcgcacgcttcattatctaatctcccagcgtggtttaatcagacgatcgaaaatttcattgcagacaggttcccaaatagaaagagcatttctccaggcaccagttgaagagcgttgatcaatggcctgttcaaaaacagttctcatccggatctgacctttaccaacttcatccgtttcacgtacaacattttttagaaccatgcttccccaggcatcccgaatttgctcctccatccacggggactgagagccattactattgctgtatttggtaagcaaaatacgtacatcaggctcgaaccctttaagatcaacgttcttgagcagatcacgaagcatatcgaaaaactgcagtgcggaggtgtagtcaaacaactcagcaggcgtgggaacaatcagcacatcagcagcacatacgacattaatcgtgccgatacccaggttaggcgcgctgtcaataactatgacatcatagtcatgagcaacagtttcaatggccagtcggagcatcaggtgtggatcggtgggcagtttaccttcatcaaatttgcccattaactcagtttcaatacggtgcagagccagacaggaaggaataatgtcaagccccggccagcaagtgggctttattgcataagtgacatcgtccttttccccaagatagaaaggcaggagagtgtcttctgcatgaatatgaagatctggtacccatccgtgatacattgaggctgttccctgggggtcgttaccttccacgagcaaaacacgtagccccttcagagccagatcctgagcaagatgaacagaaactgaggttttgtaaacgccacctttatgggcagcaaccccgatcaccggtggaaatacgtcttcagcacgtcgcaatcgcgtaccaaacacatcacgcatatgattaatttgttcaattgtataaccaacacgttgctcaacccgtcctcgaatttccatatccgggtgcggtagtcgccctgctttctcggcatctctgatagcctgagaagaaaccccaactaaatccgctgcttcacctattctccagcgccgggttattttcctcgcttccgggctgtcatcattaaactgtgcaatggcgatagccttcgtcatttcatgaccagcgtttatgcactggttaagtgtttccatgagtttcattctgaacatcctttaatcattgctttgcgtttttttattaaatcttgcaatttactgcaaagcaacaacaaaatcgcaaagtcatcaaaaaaccgcaaagttgtttaaaataagagcaacactacaaaaggagataagaagagcacatacctcagtcacttattatcactagcgctcgccgcagccgtgtaaccgagcatagcgagcgaactggcgaggaagcaaagaagaactgttctgtcagatagctcttacgctcagcgcaagaagaaatatccaccgtgggaaaaactccaggtagaggtacacacgcggatagccaattcagagtaataaactgtgataatcaaccctcatcaatgatgacgaactaacccccgatatcaggtcacatgacgaagggaaagagaaggaaatcaactgtgacaaactgccctcaaatttggcttccttaaaaattacagttcaaaaagtatgagaaaatccatgcaggctgaaggaaacagcaaaactgtgacaaattaccctcagtaggtcagaacaaatgtgacgaaccaccctcaaatctgtgacagataaccctcagactatcctgtcgtcatggaagtgatatcgcggaaggaaaatacgatatgagtcgtctggcggcctttctttttctcaatgtatgagaggcgcattggagttctgctgttgatctcattaacacagacctgcaggaagcggcggcggaagtcaggcatacgctggtaactttgaggcagctggtaacgctctatgatccagtcgattttcagagagacgatgcctgagccatccggcttacgatactgacacagggattcgtataaacgcatggcatacggattggtgatttcttttgtttcactaagccgaaactgcgtaaaccggttctgtaacccgataaagaagggaatgagatatgggttgatatgtacactgtaaagccctctggatggactgtgcgcacgtttgataaaccaaggaaaagattcatagcctttttcatcgccggcatcctcttcagggcgataaaaaaccacttccttccccgcgaaactcttcaatgcctgccgtatatccttactggcttccgcagaggtcaatccgaatatttcagcatatttagcaacatggatctcgcagataccgtcatgttcctgtagggtgccatcagattttctgatctggtcaacgaacagatacagcatacgtttttgatcccgggagagactatatgccgcctcagtgaggtcgtttgactggacgattcgcgggctatttttacgtttcttgtgattgataaccgctgtttccgccatgacagatccatgtgaagtgtgacaagtttttagattgtcacactaaataaaaaagagtcaataagcagggataactttgtgaaaaaacagcttcttctgagggcaatttgtcacagggttaagggcaatttgtcacagACAGGACTGTCATTTGAGGGTGATTTGTCACACTGAAAGGGCAATTTGTCACAACACCTTCTCTAGAACCAGCATGGATAAAGGCCTACAAGGCGCTCTAAAAAAGAAGATCTAAAAACTATAAAAAAAATAATTATAAAAATATCCCCGTGGATAAGTGGATAACCCCAAGGGAAGTTTTTTCAGGCATCGTGTGTAAGCAGAATATATAAGTGCTGTTCCCTGGTGCTTCCTCGCTCACTCGAGGGCTTCGCCCTGTCGCTCAACTGCGGCGAGCACTACTGGCTGTAAaaggacagaccacatcatggttctgtgttcattaggttgttctgtccattgctgacataatccgctccacttcaacgtaacaccgcacgaagatttctattgttcctgaaggcatattcaaatcgttttcgttaccgcttgcaggcatcatgacagaacactacttcctataaacgctacacaggctcctgagattaataatgcggatctctacgataatgggagattttcccgactgtttcgttcgcttctcagtggataacagccagcttctctgtttaacagacaaaaacagcatatccactcagttccacatttccatataaaggccaaggcatttattctcaggataattgtttcagcatcgcaaccgcatcagactccggcatcgcaaactgcacccggtgccgggcagccacatccagcgcaaaaaccttcgtgtagacttccgttgaactgatggacttatgtcccatcaggctttgcagaactttcagcggtataccggcatacagcatgtgcatcgcataggaatggcggaacgtatgtggtgtgaccggaacagagaacgtcacaccgtcagcagcagcggcggcaaccgcctccccaatccaggtcctgaccgttctgtccgtcacttcccagatccgcgctttctctgtccttcctgtgcgacggttacgccgctccatgagcttatcgcgaataaatacctgtgacggaagatcacttcgcagaataaataaatcctggtgtccctgttgataccgggaagccctgggccaacttttggcgaaaatgagacgttgatcggcacgtaagaggttccaactttcaccataatgaaataagatcactaccgggcgtattttttgagttatcgagattttcaggagctaaggaagctaaaatggagaaaaaaatcactggatataccaccgttgatatatcccaatggcatcgtaaagaacattttgaggcatttcagtcagttgctcaatgtacctataaccagaccgttcagctggatattacggcctttttaaagaccgtaaagaaaaataagcacaagttttatccggcctttattcacattcttgcccgcctgatgaatgctcatccggagttccgtatggcaatgaaagacggtgagctggtgatatgggatagtgttcacccttgttacaccgttttccatgagcaaactgaaacgttttcatcgctctggagtgaataccacgacgatttccggcagtttctacacatatattcgcaagatgtggcgtgttacggtgaaaacctggcctatttccctaaagggtttattgagaatatgtttttcgtctcagccaatccctgggtgagtttcaccagttttgatttaaacgtggccaatatggacaacttcttcgcccccgttttcaccatgggcaaatattatacgcaaggcgacaaggtgctgatgccgctggcgattcaggttcatcatgccgtttgtgatggcttccatgtcggcagaatgcttaatgaattacaacagtactgcgatgagtggcagggcggggcgtaatttttttaaggcagttattggtgcccttaaacgcctggttgctacgcctgaataagtgataataagcggatgaatggcagaaattcgatgataagctgtcaaacatgagaattggtcgacggcgcgccaaagcttgcatgcctgcagccgcgtaacctggcaaaatcggttacggttgagtaataaatggatgccctgcgtaagcggggcacatttcattacctctttctccgcacccgacatagataataacttcgtatagtatacattatacgaagttatctagtagacttaattaaggatcgatccggcgcgccaatagtcatgccccgcgcccaccggaaggagctgactgggttgaaggctctcaagggcatcggtcgagcttgacattgtaggactatattgctctaataaatttgcggccgctaatacgactcactatagggagag |
| pRC1825  (Bp5++ Hsmar1 Flag) | gatccgcggaattgGTTAATAACAGGGGACGTGGTAATCCGTCCCCTTTTTATTTCTGACTGAGTTAATAACAGGCCTGCTTCGGCAGGCCTTTTTATTTCTGACTGAGTTCTTCTCAGGCCTGCTGGTAATCGCAGGCCTTTTTATTTTCTAGcCAATTCCGACGCCTAAGAAACCATTATTATCATGACATTAGCCTATAAAAATAGGCGTACCACGAGGCCCTTTCGTCTTCACCTCGAGTCCCTATCAGTGATAGAGATTGACACCCCTATCAGTGATAGAGATACTGAGCACATCAGCAGGACGCACTGACCACTTTAAGAAGGAGATATACATATGGAAATGATGCTCGATAAGAAACAGATTCGTGCGATCTTTCTCTTTGAGTTTAAAATGGGTCGCAAAGCGGCGGAGACGACGCGTAATATTAACAACGCGTTCGGTCCTGGCACCGCGAACGAGCGTACCGTGCAATGGTGGTTCAAAAAGTTTCGCAAAGGCGACGAATCTCTGGAGGACGAAGAGCGTTCTGGCCGCCCGTCCGAGGTTGACAACGACCAGCTGCGTGCAATCATCGAAGCTGATCCGCTGACTACCACCCGCGAAGTTGCTGAAGAACTGAATGTGGATCACTCTACTGTGGTTCGCCACCTGAAACAGATCGGTAAAGTAAAAAAACTGGACAAATGGGTTCCTCATGAACTGTCTGAAAACCAGAAAAACCGTCGTTTCGAAGTTAGCTCCTCTCTGATTCTGCGTAACAACAACGAACCGTTCCTGGATCGTATCGTAACCTGTGATGAGAAATGGATTCTGTATGATAACCGTCGCCGCTCTGCTCAGTGGCTGGATCGCGAAGAAGCTCCAAAACACTTCCCGAAACCGAATCTGCACCAGAAGAAAGTCATGGTAACCGTATGGTGGTCTGCCGCAGGTGTTATCCACTATTCCTTCCTGAACCCGGGCGAAACTATCACCAGCGAAAAATACTGCCAGCAGATTGACGAAATGCACCGTAAACTGCAGCGTCTGCAGCCAGCACTGGTGAATCGTAAAGGTCCGATCCTGCTGCATGATAACGCCCGTCCGCACGTTGCCCAACCGACCCTGCAGAAACTGAACGAACTGGGCTATGAAGTTCTGCCACACCCGCCGTACTCCCCGGATCTGTCCCCGACTGACTACCATTTCTTCAAGCATCTGGACAACTTCCTGCAGGGTAAACGTTTTCACAACCAACAGGACGCAGAAAACGCTTTCCAGGAGTTCGTCGAAAGCCGTTCCACTGACTTCTACGCGACCGGTATCAACAAGCTGATCAGCCGTTGGCAGAAATGCGTGGACTGTAACGGCAGCTACTTCGATGGATCCgactacaaggacgacgatgacaagGAAgattataaagatgatgacgataaaAGAgactacaaggacgacgatgacaagTAAAAGCTTGTTCTTCTCAGGCCTGCTGGTAATCGCAGGCCTTTTTATTTGAATTCcgcggatccttctatagtgtcacctaaatgtcgacggccaggcggccgccaggcctacccactagtcaattcgggaggatcgaaacggcagatcgcaaaaaacagtacatacagaaggagacatgaacatgaacatcaaaaaaattgtaaaacaagccacagttctgacttttacgactgcacttctggcaggaggagcgactcaagccttcgcgaaagaaaataaccaaaaagcatacaaagaaacgtacggcgtctctcatattacacgccatgatatgctgcagatccctaaacagcagcaaaacgaaaaataccaagtgcctcaattcgatcaatcaacgattaaaaatattgagtctgcaaaaggacttgatgtgtgggacagctggccgctgcaaaacgctgacggaacagtagctgaatacaacggctatcacgttgtgtttgctcttgcgggaagcccgaaagacgctgatgacacatcaatctacatgttttatcaaaaggtcggcgacaactcaatcgacagctggaaaaacgcgggccgtgtctttaaagacagcgataagttcgacgccaacgatccgatcctgaaagatcagacgcaagaatggtccggttctgcaacctttacatctgacggaaaaatccgtttattctacactgactattccggtaaacattacggcaaacaaagcctgacaacagcgcaggtaaatgtgtcaaaatctgatgacacactcaaaatcaacggagtggaagatcacaaaacgatttttgacggagacggaaaaacatatcagaacgttcagcagtttatcgatgaaggcaattatacatccggcgacaaccatacgctgagagaccctcactacgttgaagacaaaggccataaataccttgtattcgaagccaacacgggaacagaaaacggataccaaggcgaagaatctttatttaacaaagcgtactacggcggcggcacgaacttcttccgtaaagaaagccagaagcttcagcagagcgctaaaaaacgcgatgctgagttagcgaacggcgccctcggtatcatagagttaaataatgattacacattgaaaaaagtaatgaagccgctgatcacttcaaacacggtaactgatgaaatcgagcgcgcgaatgttttcaaaatgaacggcaaatggtacttgttcactgattcacgcggttcaaaaatgacgatcgatggtattaactcaaacgatatttacatgcttggttatgtatcaaactctttaaccggcccttacaagccgctgaacaaaacagggcttgtgctgcaaatgggtcttgatccaaacgatgtgacattcacttactctcacttcgcagtgccgcaagccaaaggcaacaatgtggttatcacaagctacatgacaaacagaggcttcttcgaggataaaaaggcaacatttgcgccaagcttcttaatgaacatcaaaggcaataaaacatccgttgtcaaaaacagcatcctggagcaaggacagctgacagtcaactaataacagcaaaaagaaaatgccgatacttcattggcattttcttttatttctcaacaagatggtgaattgactagtgggtagatccacaggacgggtgtggtcgccatgatcgcgtagtcgatagtggctccaagtagcgaagcgagcaggactgggcggcggccaaagcggtcggacagtgctccgagaacgggtgcgcatagaaattgcatcaacgcatatagcgctagcagcacgccatagtgactggcgatgctgtcggaatggacgatatcccgcaagaggcccggcagtaccggcataaccaagcctatgcctacagcatccagggtgacggtgccgaggatgacgatgagcgcattgttagatttcatacacggtgcctgactgcgttagcaatttaactgtgataaactaccgcattaaagcttatcgatgataagctgtcaaacatgagaattgatccggaacccttaatataacttcgtataatgtatgctatacgaagttattaggtccctcgactatagggtcaccgtcgacagcgacacacttgcatcggatgcagcccggttaacgtgccggcacggcctgggtaaccaggtattttgtccacataaccgtgcgcaaaatgttgtggataagcaggacacagcagcaatccacagcaggcatacaaccgcacaccgaggttactccgttctacaggttacgacgacatgtcaatacttgcccttgacaggcattgatggaatcgtagtctcacgctgatagtctgatcgacaatacaagtgggaccgtggtcccagaccgataatcagaccgacaacacgagtgggatcgtggtcccagactaataatcagaccgacgatacgagtgggaccgtggtcccagactaataatcagaccgacgatacgagtgggaccgtggttccagactaataatcagaccgacgatacgagtgggaccgtggtcccagactaataatcagaccgacgatacgagtgggaccatggtcccagactaataatcagaccgacgatacgagtgggaccgtggtcccagtctgattatcagaccgacgatacgagtgggaccgtggtcccagactaataatcagaccgacgatacgagtgggaccgtggtcccagactaataatcagaccgacgatacgagtgggaccgtggtcccagtctgattatcagaccgacgatacaagtggaacagtgggcccagagagaatattcaggccagttatgctttctggcctgtaacaaaggacattaagtaaagacagataaacgtagactaaaacgtggtcgcatcagggtgctggcttttcaagttccttaagaatggcctcaattttctctatacactcagttggaacacgagacctgtccaggttaagcaccattttatcgcccttatacaatactgtcgctccaggagcaaactgatgtcgtgagcttaaactagttcttgatgcagatgacgttttaagcacagaagttaaaagagtgataacttcttcagcttcaaatatcaccccagcttttttctgctcatgaaggttagatgcctgctgcttaagtaattcctctttatctgtaaaggctttttgaagtgcatcacctgaccgggcagatagttcaccggggtgagaaaaaagagcaacaactgatttaggcaatttggcggtgttgatacagcgggtaataatcttacgtgaaatattttccgcatcagccagcgcagaaatatttccagcaaattcattctgcaatcggcttgcataacgctgaccacgttcataagcacttgttgggcgataatcgttacccaatctggataatgcagccatctgctcatcatccagctcgccaaccagaacacgataatcactttcggtaagtgcagcagctttacgacggcgactcccatcggcaatttctatgacaccagatactcttcgaccgaacgccggtgtctgttgaccagtcagtagaaaagaagggatgagatcatccagtgcgtcctcagtaagcagctcctggtcacgttcattacctgaccatacccgagaggtcttctcaacactatcaccccggagcacttcaagagtaaacttcacatcccgaccacatacaggcaaagtaatggcattaccgcgagccattactcctacgcgcgcaattaacgaatccaccatcggggcagctggtgtcgataacgaagtatcttcaaccggttgagtattgagcgtatgttttggaataacaggcgcacgcttcattatctaatctcccagcgtggtttaatcagacgatcgaaaatttcattgcagacaggttcccaaatagaaagagcatttctccaggcaccagttgaagagcgttgatcaatggcctgttcaaaaacagttctcatccggatctgacctttaccaacttcatccgtttcacgtacaacattttttagaaccatgcttccccaggcatcccgaatttgctcctccatccacggggactgagagccattactattgctgtatttggtaagcaaaatacgtacatcaggctcgaaccctttaagatcaacgttcttgagcagatcacgaagcatatcgaaaaactgcagtgcggaggtgtagtcaaacaactcagcaggcgtgggaacaatcagcacatcagcagcacatacgacattaatcgtgccgatacccaggttaggcgcgctgtcaataactatgacatcatagtcatgagcaacagtttcaatggccagtcggagcatcaggtgtggatcggtgggcagtttaccttcatcaaatttgcccattaactcagtttcaatacggtgcagagccagacaggaaggaataatgtcaagccccggccagcaagtgggctttattgcataagtgacatcgtccttttccccaagatagaaaggcaggagagtgtcttctgcatgaatatgaagatctggtacccatccgtgatacattgaggctgttccctgggggtcgttaccttccacgagcaaaacacgtagccccttcagagccagatcctgagcaagatgaacagaaactgaggttttgtaaacgccacctttatgggcagcaaccccgatcaccggtggaaatacgtcttcagcacgtcgcaatcgcgtaccaaacacatcacgcatatgattaatttgttcaattgtataaccaacacgttgctcaacccgtcctcgaatttccatatccgggtgcggtagtcgccctgctttctcggcatctctgatagcctgagaagaaaccccaactaaatccgctgcttcacctattctccagcgccgggttattttcctcgcttccgggctgtcatcattaaactgtgcaatggcgatagccttcgtcatttcatgaccagcgtttatgcactggttaagtgtttccatgagtttcattctgaacatcctttaatcattgctttgcgtttttttattaaatcttgcaatttactgcaaagcaacaacaaaatcgcaaagtcatcaaaaaaccgcaaagttgtttaaaataagagcaacactacaaaaggagataagaagagcacatacctcagtcacttattatcactagcgctcgccgcagccgtgtaaccgagcatagcgagcgaactggcgaggaagcaaagaagaactgttctgtcagatagctcttacgctcagcgcaagaagaaatatccaccgtgggaaaaactccaggtagaggtacacacgcggatagccaattcagagtaataaactgtgataatcaaccctcatcaatgatgacgaactaacccccgatatcaggtcacatgacgaagggaaagagaaggaaatcaactgtgacaaactgccctcaaatttggcttccttaaaaattacagttcaaaaagtatgagaaaatccatgcaggctgaaggaaacagcaaaactgtgacaaattaccctcagtaggtcagaacaaatgtgacgaaccaccctcaaatctgtgacagataaccctcagactatcctgtcgtcatggaagtgatatcgcggaaggaaaatacgatatgagtcgtctggcggcctttctttttctcaatgtatgagaggcgcattggagttctgctgttgatctcattaacacagacctgcaggaagcggcggcggaagtcaggcatacgctggtaactttgaggcagctggtaacgctctatgatccagtcgattttcagagagacgatgcctgagccatccggcttacgatactgacacagggattcgtataaacgcatggcatacggattggtgatttcttttgtttcactaagccgaaactgcgtaaaccggttctgtaacccgataaagaagggaatgagatatgggttgatatgtacactgtaaagccctctggatggactgtgcgcacgtttgataaaccaaggaaaagattcatagcctttttcatcgccggcatcctcttcagggcgataaaaaaccacttccttccccgcgaaactcttcaatgcctgccgtatatccttactggcttccgcagaggtcaatccgaatatttcagcatatttagcaacatggatctcgcagataccgtcatgttcctgtagggtgccatcagattttctgatctggtcaacgaacagatacagcatacgtttttgatcccgggagagactatatgccgcctcagtgaggtcgtttgactggacgattcgcgggctatttttacgtttcttgtgattgataaccgctgtttccgccatgacagatccatgtgaagtgtgacaagtttttagattgtcacactaaataaaaaagagtcaataagcagggataactttgtgaaaaaacagcttcttctgagggcaatttgtcacagggttaagggcaatttgtcacagACAGGACTGTCATTTGAGGGTGATTTGTCACACTGAAAGGGCAATTTGTCACAACACCTTCTCTAGAACCAGCATGGATAAAGGCCTACAAGGCGCTCTAAAAAAGAAGATCTAAAAACTATAAAAAAAATAATTATAAAAATATCCCCGTGGATAAGTGGATAACCCCAAGGGAAGTTTTTTCAGGCATCGTGTGTAAGCAGAATATATAAGTGCTGTTCCCTGGTGCTTCCTCGCTCACTCGAGGGCTTCGCCCTGTCGCTCAACTGCGGCGAGCACTACTGGCTGTAAaaggacagaccacatcatggttctgtgttcattaggttgttctgtccattgctgacataatccgctccacttcaacgtaacaccgcacgaagatttctattgttcctgaaggcatattcaaatcgttttcgttaccgcttgcaggcatcatgacagaacactacttcctataaacgctacacaggctcctgagattaataatgcggatctctacgataatgggagattttcccgactgtttcgttcgcttctcagtggataacagccagcttctctgtttaacagacaaaaacagcatatccactcagttccacatttccatataaaggccaaggcatttattctcaggataattgtttcagcatcgcaaccgcatcagactccggcatcgcaaactgcacccggtgccgggcagccacatccagcgcaaaaaccttcgtgtagacttccgttgaactgatggacttatgtcccatcaggctttgcagaactttcagcggtataccggcatacagcatgtgcatcgcataggaatggcggaacgtatgtggtgtgaccggaacagagaacgtcacaccgtcagcagcagcggcggcaaccgcctccccaatccaggtcctgaccgttctgtccgtcacttcccagatccgcgctttctctgtccttcctgtgcgacggttacgccgctccatgagcttatcgcgaataaatacctgtgacggaagatcacttcgcagaataaataaatcctggtgtccctgttgataccgggaagccctgggccaacttttggcgaaaatgagacgttgatcggcacgtaagaggttccaactttcaccataatgaaataagatcactaccgggcgtattttttgagttatcgagattttcaggagctaaggaagctaaaatggagaaaaaaatcactggatataccaccgttgatatatcccaatggcatcgtaaagaacattttgaggcatttcagtcagttgctcaatgtacctataaccagaccgttcagctggatattacggcctttttaaagaccgtaaagaaaaataagcacaagttttatccggcctttattcacattcttgcccgcctgatgaatgctcatccggagttccgtatggcaatgaaagacggtgagctggtgatatgggatagtgttcacccttgttacaccgttttccatgagcaaactgaaacgttttcatcgctctggagtgaataccacgacgatttccggcagtttctacacatatattcgcaagatgtggcgtgttacggtgaaaacctggcctatttccctaaagggtttattgagaatatgtttttcgtctcagccaatccctgggtgagtttcaccagttttgatttaaacgtggccaatatggacaacttcttcgcccccgttttcaccatgggcaaatattatacgcaaggcgacaaggtgctgatgccgctggcgattcaggttcatcatgccgtttgtgatggcttccatgtcggcagaatgcttaatgaattacaacagtactgcgatgagtggcagggcggggcgtaatttttttaaggcagttattggtgcccttaaacgcctggttgctacgcctgaataagtgataataagcggatgaatggcagaaattcgatgataagctgtcaaacatgagaattggtcgacggcgcgccaaagcttgcatgcctgcagccgcgtaacctggcaaaatcggttacggttgagtaataaatggatgccctgcgtaagcggggcacatttcattacctctttctccgcacccgacatagataataacttcgtatagtatacattatacgaagttatctagtagacttaattaaggatcgatccggcgcgccaatagtcatgccccgcgcccaccggaaggagctgactgggttgaaggctctcaagggcatcggtcgagcttgacattgtaggactatattgctctaataaatttgcggccgctaatacgactcactatagggagag |
| pRC1826  (Bp6++ Hsmar1 Flag) | gatccgcggaattgGTTAATAACAGGGGACGTGGTAATCCGTCCCCTTTTTATTTCTGACTGAGTTAATAACAGGCCTGCTTCGGCAGGCCTTTTTATTTCTGACTGAGTTCTTCTCAGGCCTGCTGGTAATCGCAGGCCTTTTTATTTTCTAGTCAATTCCGACGTCTAAGAAACCATTATTATCATGACATTAACCTATAAAAATAGGCGTATCACGAGGCCCTTTCGTCTTCACCTCGAGTCCCTATCAGTGATAGAGATTGACATCCCTATCAGTGATAGAGATACTGAGCACATCAGCAGGACGCACTGACCACTTTAAGAAGGAGATATACATATGGAAATGATGCTCGATAAGAAACAGATTCGTGCGATCTTTCTCTTTGAGTTTAAAATGGGTCGCAAAGCGGCGGAGACGACGCGTAATATTAACAACGCGTTCGGTCCTGGCACCGCGAACGAGCGTACCGTGCAATGGTGGTTCAAAAAGTTTCGCAAAGGCGACGAATCTCTGGAGGACGAAGAGCGTTCTGGCCGCCCGTCCGAGGTTGACAACGACCAGCTGCGTGCAATCATCGAAGCTGATCCGCTGACTACCACCCGCGAAGTTGCTGAAGAACTGAATGTGGATCACTCTACTGTGGTTCGCCACCTGAAACAGATCGGTAAAGTAAAAAAACTGGACAAATGGGTTCCTCATGAACTGTCTGAAAACCAGAAAAACCGTCGTTTCGAAGTTAGCTCCTCTCTGATTCTGCGTAACAACAACGAACCGTTCCTGGATCGTATCGTAACCTGTGATGAGAAATGGATTCTGTATGATAACCGTCGCCGCTCTGCTCAGTGGCTGGATCGCGAAGAAGCTCCAAAACACTTCCCGAAACCGAATCTGCACCAGAAGAAAGTCATGGTAACCGTATGGTGGTCTGCCGCAGGTGTTATCCACTATTCCTTCCTGAACCCGGGCGAAACTATCACCAGCGAAAAATACTGCCAGCAGATTGACGAAATGCACCGTAAACTGCAGCGTCTGCAGCCAGCACTGGTGAATCGTAAAGGTCCGATCCTGCTGCATGATAACGCCCGTCCGCACGTTGCCCAACCGACCCTGCAGAAACTGAACGAACTGGGCTATGAAGTTCTGCCACACCCGCCGTACTCCCCGGATCTGTCCCCGACTGACTACCATTTCTTCAAGCATCTGGACAACTTCCTGCAGGGTAAACGTTTTCACAACCAACAGGACGCAGAAAACGCTTTCCAGGAGTTCGTCGAAAGCCGTTCCACTGACTTCTACGCGACCGGTATCAACAAGCTGATCAGCCGTTGGCAGAAATGCGTGGACTGTAACGGCAGCTACTTCGATGGATCCgactacaaggacgacgatgacaagGAAgattataaagatgatgacgataaaAGAgactacaaggacgacgatgacaagTAAAAGCTTGTTCTTCTCAGGCCTGCTGGTAATCGCAGGCCTTTTTATTTGAATTCcgcggatccttctatagtgtcacctaaatgtcgacggccaggcggccgccaggcctacccactagtcaattcgggaggatcgaaacggcagatcgcaaaaaacagtacatacagaaggagacatgaacatgaacatcaaaaaaattgtaaaacaagccacagttctgacttttacgactgcacttctggcaggaggagcgactcaagccttcgcgaaagaaaataaccaaaaagcatacaaagaaacgtacggcgtctctcatattacacgccatgatatgctgcagatccctaaacagcagcaaaacgaaaaataccaagtgcctcaattcgatcaatcaacgattaaaaatattgagtctgcaaaaggacttgatgtgtgggacagctggccgctgcaaaacgctgacggaacagtagctgaatacaacggctatcacgttgtgtttgctcttgcgggaagcccgaaagacgctgatgacacatcaatctacatgttttatcaaaaggtcggcgacaactcaatcgacagctggaaaaacgcgggccgtgtctttaaagacagcgataagttcgacgccaacgatccgatcctgaaagatcagacgcaagaatggtccggttctgcaacctttacatctgacggaaaaatccgtttattctacactgactattccggtaaacattacggcaaacaaagcctgacaacagcgcaggtaaatgtgtcaaaatctgatgacacactcaaaatcaacggagtggaagatcacaaaacgatttttgacggagacggaaaaacatatcagaacgttcagcagtttatcgatgaaggcaattatacatccggcgacaaccatacgctgagagaccctcactacgttgaagacaaaggccataaataccttgtattcgaagccaacacgggaacagaaaacggataccaaggcgaagaatctttatttaacaaagcgtactacggcggcggcacgaacttcttccgtaaagaaagccagaagcttcagcagagcgctaaaaaacgcgatgctgagttagcgaacggcgccctcggtatcatagagttaaataatgattacacattgaaaaaagtaatgaagccgctgatcacttcaaacacggtaactgatgaaatcgagcgcgcgaatgttttcaaaatgaacggcaaatggtacttgttcactgattcacgcggttcaaaaatgacgatcgatggtattaactcaaacgatatttacatgcttggttatgtatcaaactctttaaccggcccttacaagccgctgaacaaaacagggcttgtgctgcaaatgggtcttgatccaaacgatgtgacattcacttactctcacttcgcagtgccgcaagccaaaggcaacaatgtggttatcacaagctacatgacaaacagaggcttcttcgaggataaaaaggcaacatttgcgccaagcttcttaatgaacatcaaaggcaataaaacatccgttgtcaaaaacagcatcctggagcaaggacagctgacagtcaactaataacagcaaaaagaaaatgccgatacttcattggcattttcttttatttctcaacaagatggtgaattgactagtgggtagatccacaggacgggtgtggtcgccatgatcgcgtagtcgatagtggctccaagtagcgaagcgagcaggactgggcggcggccaaagcggtcggacagtgctccgagaacgggtgcgcatagaaattgcatcaacgcatatagcgctagcagcacgccatagtgactggcgatgctgtcggaatggacgatatcccgcaagaggcccggcagtaccggcataaccaagcctatgcctacagcatccagggtgacggtgccgaggatgacgatgagcgcattgttagatttcatacacggtgcctgactgcgttagcaatttaactgtgataaactaccgcattaaagcttatcgatgataagctgtcaaacatgagaattgatccggaacccttaatataacttcgtataatgtatgctatacgaagttattaggtccctcgactatagggtcaccgtcgacagcgacacacttgcatcggatgcagcccggttaacgtgccggcacggcctgggtaaccaggtattttgtccacataaccgtgcgcaaaatgttgtggataagcaggacacagcagcaatccacagcaggcatacaaccgcacaccgaggttactccgttctacaggttacgacgacatgtcaatacttgcccttgacaggcattgatggaatcgtagtctcacgctgatagtctgatcgacaatacaagtgggaccgtggtcccagaccgataatcagaccgacaacacgagtgggatcgtggtcccagactaataatcagaccgacgatacgagtgggaccgtggtcccagactaataatcagaccgacgatacgagtgggaccgtggttccagactaataatcagaccgacgatacgagtgggaccgtggtcccagactaataatcagaccgacgatacgagtgggaccatggtcccagactaataatcagaccgacgatacgagtgggaccgtggtcccagtctgattatcagaccgacgatacgagtgggaccgtggtcccagactaataatcagaccgacgatacgagtgggaccgtggtcccagactaataatcagaccgacgatacgagtgggaccgtggtcccagtctgattatcagaccgacgatacaagtggaacagtgggcccagagagaatattcaggccagttatgctttctggcctgtaacaaaggacattaagtaaagacagataaacgtagactaaaacgtggtcgcatcagggtgctggcttttcaagttccttaagaatggcctcaattttctctatacactcagttggaacacgagacctgtccaggttaagcaccattttatcgcccttatacaatactgtcgctccaggagcaaactgatgtcgtgagcttaaactagttcttgatgcagatgacgttttaagcacagaagttaaaagagtgataacttcttcagcttcaaatatcaccccagcttttttctgctcatgaaggttagatgcctgctgcttaagtaattcctctttatctgtaaaggctttttgaagtgcatcacctgaccgggcagatagttcaccggggtgagaaaaaagagcaacaactgatttaggcaatttggcggtgttgatacagcgggtaataatcttacgtgaaatattttccgcatcagccagcgcagaaatatttccagcaaattcattctgcaatcggcttgcataacgctgaccacgttcataagcacttgttgggcgataatcgttacccaatctggataatgcagccatctgctcatcatccagctcgccaaccagaacacgataatcactttcggtaagtgcagcagctttacgacggcgactcccatcggcaatttctatgacaccagatactcttcgaccgaacgccggtgtctgttgaccagtcagtagaaaagaagggatgagatcatccagtgcgtcctcagtaagcagctcctggtcacgttcattacctgaccatacccgagaggtcttctcaacactatcaccccggagcacttcaagagtaaacttcacatcccgaccacatacaggcaaagtaatggcattaccgcgagccattactcctacgcgcgcaattaacgaatccaccatcggggcagctggtgtcgataacgaagtatcttcaaccggttgagtattgagcgtatgttttggaataacaggcgcacgcttcattatctaatctcccagcgtggtttaatcagacgatcgaaaatttcattgcagacaggttcccaaatagaaagagcatttctccaggcaccagttgaagagcgttgatcaatggcctgttcaaaaacagttctcatccggatctgacctttaccaacttcatccgtttcacgtacaacattttttagaaccatgcttccccaggcatcccgaatttgctcctccatccacggggactgagagccattactattgctgtatttggtaagcaaaatacgtacatcaggctcgaaccctttaagatcaacgttcttgagcagatcacgaagcatatcgaaaaactgcagtgcggaggtgtagtcaaacaactcagcaggcgtgggaacaatcagcacatcagcagcacatacgacattaatcgtgccgatacccaggttaggcgcgctgtcaataactatgacatcatagtcatgagcaacagtttcaatggccagtcggagcatcaggtgtggatcggtgggcagtttaccttcatcaaatttgcccattaactcagtttcaatacggtgcagagccagacaggaaggaataatgtcaagccccggccagcaagtgggctttattgcataagtgacatcgtccttttccccaagatagaaaggcaggagagtgtcttctgcatgaatatgaagatctggtacccatccgtgatacattgaggctgttccctgggggtcgttaccttccacgagcaaaacacgtagccccttcagagccagatcctgagcaagatgaacagaaactgaggttttgtaaacgccacctttatgggcagcaaccccgatcaccggtggaaatacgtcttcagcacgtcgcaatcgcgtaccaaacacatcacgcatatgattaatttgttcaattgtataaccaacacgttgctcaacccgtcctcgaatttccatatccgggtgcggtagtcgccctgctttctcggcatctctgatagcctgagaagaaaccccaactaaatccgctgcttcacctattctccagcgccgggttattttcctcgcttccgggctgtcatcattaaactgtgcaatggcgatagccttcgtcatttcatgaccagcgtttatgcactggttaagtgtttccatgagtttcattctgaacatcctttaatcattgctttgcgtttttttattaaatcttgcaatttactgcaaagcaacaacaaaatcgcaaagtcatcaaaaaaccgcaaagttgtttaaaataagagcaacactacaaaaggagataagaagagcacatacctcagtcacttattatcactagcgctcgccgcagccgtgtaaccgagcatagcgagcgaactggcgaggaagcaaagaagaactgttctgtcagatagctcttacgctcagcgcaagaagaaatatccaccgtgggaaaaactccaggtagaggtacacacgcggatagccaattcagagtaataaactgtgataatcaaccctcatcaatgatgacgaactaacccccgatatcaggtcacatgacgaagggaaagagaaggaaatcaactgtgacaaactgccctcaaatttggcttccttaaaaattacagttcaaaaagtatgagaaaatccatgcaggctgaaggaaacagcaaaactgtgacaaattaccctcagtaggtcagaacaaatgtgacgaaccaccctcaaatctgtgacagataaccctcagactatcctgtcgtcatggaagtgatatcgcggaaggaaaatacgatatgagtcgtctggcggcctttctttttctcaatgtatgagaggcgcattggagttctgctgttgatctcattaacacagacctgcaggaagcggcggcggaagtcaggcatacgctggtaactttgaggcagctggtaacgctctatgatccagtcgattttcagagagacgatgcctgagccatccggcttacgatactgacacagggattcgtataaacgcatggcatacggattggtgatttcttttgtttcactaagccgaaactgcgtaaaccggttctgtaacccgataaagaagggaatgagatatgggttgatatgtacactgtaaagccctctggatggactgtgcgcacgtttgataaaccaaggaaaagattcatagcctttttcatcgccggcatcctcttcagggcgataaaaaaccacttccttccccgcgaaactcttcaatgcctgccgtatatccttactggcttccgcagaggtcaatccgaatatttcagcatatttagcaacatggatctcgcagataccgtcatgttcctgtagggtgccatcagattttctgatctggtcaacgaacagatacagcatacgtttttgatcccgggagagactatatgccgcctcagtgaggtcgtttgactggacgattcgcgggctatttttacgtttcttgtgattgataaccgctgtttccgccatgacagatccatgtgaagtgtgacaagtttttagattgtcacactaaataaaaaagagtcaataagcagggataactttgtgaaaaaacagcttcttctgagggcaatttgtcacagggttaagggcaatttgtcacagACAGGACTGTCATTTGAGGGTGATTTGTCACACTGAAAGGGCAATTTGTCACAACACCTTCTCTAGAACCAGCATGGATAAAGGCCTACAAGGCGCTCTAAAAAAGAAGATCTAAAAACTATAAAAAAAATAATTATAAAAATATCCCCGTGGATAAGTGGATAACCCCAAGGGAAGTTTTTTCAGGCATCGTGTGTAAGCAGAATATATAAGTGCTGTTCCCTGGTGCTTCCTCGCTCACTCGAGGGCTTCGCCCTGTCGCTCAACTGCGGCGAGCACTACTGGCTGTAAaaggacagaccacatcatggttctgtgttcattaggttgttctgtccattgctgacataatccgctccacttcaacgtaacaccgcacgaagatttctattgttcctgaaggcatattcaaatcgttttcgttaccgcttgcaggcatcatgacagaacactacttcctataaacgctacacaggctcctgagattaataatgcggatctctacgataatgggagattttcccgactgtttcgttcgcttctcagtggataacagccagcttctctgtttaacagacaaaaacagcatatccactcagttccacatttccatataaaggccaaggcatttattctcaggataattgtttcagcatcgcaaccgcatcagactccggcatcgcaaactgcacccggtgccgggcagccacatccagcgcaaaaaccttcgtgtagacttccgttgaactgatggacttatgtcccatcaggctttgcagaactttcagcggtataccggcatacagcatgtgcatcgcataggaatggcggaacgtatgtggtgtgaccggaacagagaacgtcacaccgtcagcagcagcggcggcaaccgcctccccaatccaggtcctgaccgttctgtccgtcacttcccagatccgcgctttctctgtccttcctgtgcgacggttacgccgctccatgagcttatcgcgaataaatacctgtgacggaagatcacttcgcagaataaataaatcctggtgtccctgttgataccgggaagccctgggccaacttttggcgaaaatgagacgttgatcggcacgtaagaggttccaactttcaccataatgaaataagatcactaccgggcgtattttttgagttatcgagattttcaggagctaaggaagctaaaatggagaaaaaaatcactggatataccaccgttgatatatcccaatggcatcgtaaagaacattttgaggcatttcagtcagttgctcaatgtacctataaccagaccgttcagctggatattacggcctttttaaagaccgtaaagaaaaataagcacaagttttatccggcctttattcacattcttgcccgcctgatgaatgctcatccggagttccgtatggcaatgaaagacggtgagctggtgatatgggatagtgttcacccttgttacaccgttttccatgagcaaactgaaacgttttcatcgctctggagtgaataccacgacgatttccggcagtttctacacatatattcgcaagatgtggcgtgttacggtgaaaacctggcctatttccctaaagggtttattgagaatatgtttttcgtctcagccaatccctgggtgagtttcaccagttttgatttaaacgtggccaatatggacaacttcttcgcccccgttttcaccatgggcaaatattatacgcaaggcgacaaggtgctgatgccgctggcgattcaggttcatcatgccgtttgtgatggcttccatgtcggcagaatgcttaatgaattacaacagtactgcgatgagtggcagggcggggcgtaatttttttaaggcagttattggtgcccttaaacgcctggttgctacgcctgaataagtgataataagcggatgaatggcagaaattcgatgataagctgtcaaacatgagaattggtcgacggcgcgccaaagcttgcatgcctgcagccgcgtaacctggcaaaatcggttacggttgagtaataaatggatgccctgcgtaagcggggcacatttcattacctctttctccgcacccgacatagataataacttcgtatagtatacattatacgaagttatctagtagacttaattaaggatcgatccggcgcgccaatagtcatgccccgcgcccaccggaaggagctgactgggttgaaggctctcaagggcatcggtcgagcttgacattgtaggactatattgctctaataaatttgcggccgctaatacgactcactatagggagag |
| pRC1833  (Bp-EE- Hsmar1 Flag) | gatccgcggaattgTTAATAACAGGGGACGTGGTAATCCGTCCCCTTTTTATTTCTGACTGAGTTAATAACAGGCCTGCTTCGGCAGGCCTTTTTATTTCTGACTGAGTTCTTCTCAGGCCTGCTGGTAATCGCAGGCCTTTTTATTTTCTAGgCTGACTGACTGACTGACTGACTGACTGACTGACTGACTGACTGACTGACTGACTGACTGACTGACTGACTGACTGACTGACTGACTGACTGACTGACTGACTGACTGACTGACTGACTGACTGACTGACTGACTGACTGACTGACTGACTGACTGACTtctcctCTGACCATATGGAAATGATGCTCGATAAGAAACAGATTCGTGCGATCTTTCTCTTTGAGTTTAAAATGGGTCGCAAAGCGGCGGAGACGACGCGTAATATTAACAACGCGTTCGGTCCTGGCACCGCGAACGAGCGTACCGTGCAATGGTGGTTCAAAAAGTTTCGCAAAGGCGACGAATCTCTGGAGGACGAAGAGCGTTCTGGCCGCCCGTCCGAGGTTGACAACGACCAGCTGCGTGCAATCATCGAAGCTGATCCGCTGACTACCACCCGCGAAGTTGCTGAAGAACTGAATGTGGATCACTCTACTGTGGTTCGCCACCTGAAACAGATCGGTAAAGTAAAAAAACTGGACAAATGGGTTCCTCATGAACTGTCTGAAAACCAGAAAAACCGTCGTTTCGAAGTTAGCTCCTCTCTGATTCTGCGTAACAACAACGAACCGTTCCTGGATCGTATCGTAACCTGTGATGAGAAATGGATTCTGTATGATAACCGTCGCCGCTCTGCTCAGTGGCTGGATCGCGAAGAAGCTCCAAAACACTTCCCGAAACCGAATCTGCACCAGAAGAAAGTCATGGTAACCGTATGGTGGTCTGCCGCAGGTGTTATCCACTATTCCTTCCTGAACCCGGGCGAAACTATCACCAGCGAAAAATACTGCCAGCAGATTGACGAAATGCACCGTAAACTGCAGCGTCTGCAGCCAGCACTGGTGAATCGTAAAGGTCCGATCCTGCTGCATGATAACGCCCGTCCGCACGTTGCCCAACCGACCCTGCAGAAACTGAACGAACTGGGCTATGAAGTTCTGCCACACCCGCCGTACTCCCCGGATCTGTCCCCGACTGACTACCATTTCTTCAAGCATCTGGACAACTTCCTGCAGGGTAAACGTTTTCACAACCAACAGGACGCAGAAAACGCTTTCCAGGAGTTCGTCGAAAGCCGTTCCACTGACTTCTACGCGACCGGTATCAACAAGCTGATCAGCCGTTGGCAGAAATGCGTGGACTGTAACGGCAGCTACTTCGATGGATCCgactacaaggacgacgatgacaagGAAgattataaagatgatgacgataaaAGAgactacaaggacgacgatgacaagTAAAAGCTTGTTCTTCTCAGGCCTGCTGGTAATCGCAGGCCTTTTTATTTGAATTCcgcggatccttctatagtgtcacctaaatgtcgacggccaggcggccgccaggcctacccactagtcaattcgggaggatcgaaacggcagatcgcaaaaaacagtacatacagaaggagacatgaacatgaacatcaaaaaaattgtaaaacaagccacagttctgacttttacgactgcacttctggcaggaggagcgactcaagccttcgcgaaagaaaataaccaaaaagcatacaaagaaacgtacggcgtctctcatattacacgccatgatatgctgcagatccctaaacagcagcaaaacgaaaaataccaagtgcctcaattcgatcaatcaacgattaaaaatattgagtctgcaaaaggacttgatgtgtgggacagctggccgctgcaaaacgctgacggaacagtagctgaatacaacggctatcacgttgtgtttgctcttgcgggaagcccgaaagacgctgatgacacatcaatctacatgttttatcaaaaggtcggcgacaactcaatcgacagctggaaaaacgcgggccgtgtctttaaagacagcgataagttcgacgccaacgatccgatcctgaaagatcagacgcaagaatggtccggttctgcaacctttacatctgacggaaaaatccgtttattctacactgactattccggtaaacattacggcaaacaaagcctgacaacagcgcaggtaaatgtgtcaaaatctgatgacacactcaaaatcaacggagtggaagatcacaaaacgatttttgacggagacggaaaaacatatcagaacgttcagcagtttatcgatgaaggcaattatacatccggcgacaaccatacgctgagagaccctcactacgttgaagacaaaggccataaataccttgtattcgaagccaacacgggaacagaaaacggataccaaggcgaagaatctttatttaacaaagcgtactacggcggcggcacgaacttcttccgtaaagaaagccagaagcttcagcagagcgctaaaaaacgcgatgctgagttagcgaacggcgccctcggtatcatagagttaaataatgattacacattgaaaaaagtaatgaagccgctgatcacttcaaacacggtaactgatgaaatcgagcgcgcgaatgttttcaaaatgaacggcaaatggtacttgttcactgattcacgcggttcaaaaatgacgatcgatggtattaactcaaacgatatttacatgcttggttatgtatcaaactctttaaccggcccttacaagccgctgaacaaaacagggcttgtgctgcaaatgggtcttgatccaaacgatgtgacattcacttactctcacttcgcagtgccgcaagccaaaggcaacaatgtggttatcacaagctacatgacaaacagaggcttcttcgaggataaaaaggcaacatttgcgccaagcttcttaatgaacatcaaaggcaataaaacatccgttgtcaaaaacagcatcctggagcaaggacagctgacagtcaactaataacagcaaaaagaaaatgccgatacttcattggcattttcttttatttctcaacaagatggtgaattgactagtgggtagatccacaggacgggtgtggtcgccatgatcgcgtagtcgatagtggctccaagtagcgaagcgagcaggactgggcggcggccaaagcggtcggacagtgctccgagaacgggtgcgcatagaaattgcatcaacgcatatagcgctagcagcacgccatagtgactggcgatgctgtcggaatggacgatatcccgcaagaggcccggcagtaccggcataaccaagcctatgcctacagcatccagggtgacggtgccgaggatgacgatgagcgcattgttagatttcatacacggtgcctgactgcgttagcaatttaactgtgataaactaccgcattaaagcttatcgatgataagctgtcaaacatgagaattgatccggaacccttaatataacttcgtataatgtatgctatacgaagttattaggtccctcgactatagggtcaccgtcgacagcgacacacttgcatcggatgcagcccggttaacgtgccggcacggcctgggtaaccaggtattttgtccacataaccgtgcgcaaaatgttgtggataagcaggacacagcagcaatccacagcaggcatacaaccgcacaccgaggttactccgttctacaggttacgacgacatgtcaatacttgcccttgacaggcattgatggaatcgtagtctcacgctgatagtctgatcgacaatacaagtgggaccgtggtcccagaccgataatcagaccgacaacacgagtgggatcgtggtcccagactaataatcagaccgacgatacgagtgggaccgtggtcccagactaataatcagaccgacgatacgagtgggaccgtggttccagactaataatcagaccgacgatacgagtgggaccgtggtcccagactaataatcagaccgacgatacgagtgggaccatggtcccagactaataatcagaccgacgatacgagtgggaccgtggtcccagtctgattatcagaccgacgatacgagtgggaccgtggtcccagactaataatcagaccgacgatacgagtgggaccgtggtcccagactaataatcagaccgacgatacgagtgggaccgtggtcccagtctgattatcagaccgacgatacaagtggaacagtgggcccagagagaatattcaggccagttatgctttctggcctgtaacaaaggacattaagtaaagacagataaacgtagactaaaacgtggtcgcatcagggtgctggcttttcaagttccttaagaatggcctcaattttctctatacactcagttggaacacgagacctgtccaggttaagcaccattttatcgcccttatacaatactgtcgctccaggagcaaactgatgtcgtgagcttaaactagttcttgatgcagatgacgttttaagcacagaagttaaaagagtgataacttcttcagcttcaaatatcaccccagcttttttctgctcatgaaggttagatgcctgctgcttaagtaattcctctttatctgtaaaggctttttgaagtgcatcacctgaccgggcagatagttcaccggggtgagaaaaaagagcaacaactgatttaggcaatttggcggtgttgatacagcgggtaataatcttacgtgaaatattttccgcatcagccagcgcagaaatatttccagcaaattcattctgcaatcggcttgcataacgctgaccacgttcataagcacttgttgggcgataatcgttacccaatctggataatgcagccatctgctcatcatccagctcgccaaccagaacacgataatcactttcggtaagtgcagcagctttacgacggcgactcccatcggcaatttctatgacaccagatactcttcgaccgaacgccggtgtctgttgaccagtcagtagaaaagaagggatgagatcatccagtgcgtcctcagtaagcagctcctggtcacgttcattacctgaccatacccgagaggtcttctcaacactatcaccccggagcacttcaagagtaaacttcacatcccgaccacatacaggcaaagtaatggcattaccgcgagccattactcctacgcgcgcaattaacgaatccaccatcggggcagctggtgtcgataacgaagtatcttcaaccggttgagtattgagcgtatgttttggaataacaggcgcacgcttcattatctaatctcccagcgtggtttaatcagacgatcgaaaatttcattgcagacaggttcccaaatagaaagagcatttctccaggcaccagttgaagagcgttgatcaatggcctgttcaaaaacagttctcatccggatctgacctttaccaacttcatccgtttcacgtacaacattttttagaaccatgcttccccaggcatcccgaatttgctcctccatccacggggactgagagccattactattgctgtatttggtaagcaaaatacgtacatcaggctcgaaccctttaagatcaacgttcttgagcagatcacgaagcatatcgaaaaactgcagtgcggaggtgtagtcaaacaactcagcaggcgtgggaacaatcagcacatcagcagcacatacgacattaatcgtgccgatacccaggttaggcgcgctgtcaataactatgacatcatagtcatgagcaacagtttcaatggccagtcggagcatcaggtgtggatcggtgggcagtttaccttcatcaaatttgcccattaactcagtttcaatacggtgcagagccagacaggaaggaataatgtcaagccccggccagcaagtgggctttattgcataagtgacatcgtccttttccccaagatagaaaggcaggagagtgtcttctgcatgaatatgaagatctggtacccatccgtgatacattgaggctgttccctgggggtcgttaccttccacgagcaaaacacgtagccccttcagagccagatcctgagcaagatgaacagaaactgaggttttgtaaacgccacctttatgggcagcaaccccgatcaccggtggaaatacgtcttcagcacgtcgcaatcgcgtaccaaacacatcacgcatatgattaatttgttcaattgtataaccaacacgttgctcaacccgtcctcgaatttccatatccgggtgcggtagtcgccctgctttctcggcatctctgatagcctgagaagaaaccccaactaaatccgctgcttcacctattctccagcgccgggttattttcctcgcttccgggctgtcatcattaaactgtgcaatggcgatagccttcgtcatttcatgaccagcgtttatgcactggttaagtgtttccatgagtttcattctgaacatcctttaatcattgctttgcgtttttttattaaatcttgcaatttactgcaaagcaacaacaaaatcgcaaagtcatcaaaaaaccgcaaagttgtttaaaataagagcaacactacaaaaggagataagaagagcacatacctcagtcacttattatcactagcgctcgccgcagccgtgtaaccgagcatagcgagcgaactggcgaggaagcaaagaagaactgttctgtcagatagctcttacgctcagcgcaagaagaaatatccaccgtgggaaaaactccaggtagaggtacacacgcggatagccaattcagagtaataaactgtgataatcaaccctcatcaatgatgacgaactaacccccgatatcaggtcacatgacgaagggaaagagaaggaaatcaactgtgacaaactgccctcaaatttggcttccttaaaaattacagttcaaaaagtatgagaaaatccatgcaggctgaaggaaacagcaaaactgtgacaaattaccctcagtaggtcagaacaaatgtgacgaaccaccctcaaatctgtgacagataaccctcagactatcctgtcgtcatggaagtgatatcgcggaaggaaaatacgatatgagtcgtctggcggcctttctttttctcaatgtatgagaggcgcattggagttctgctgttgatctcattaacacagacctgcaggaagcggcggcggaagtcaggcatacgctggtaactttgaggcagctggtaacgctctatgatccagtcgattttcagagagacgatgcctgagccatccggcttacgatactgacacagggattcgtataaacgcatggcatacggattggtgatttcttttgtttcactaagccgaaactgcgtaaaccggttctgtaacccgataaagaagggaatgagatatgggttgatatgtacactgtaaagccctctggatggactgtgcgcacgtttgataaaccaaggaaaagattcatagcctttttcatcgccggcatcctcttcagggcgataaaaaaccacttccttccccgcgaaactcttcaatgcctgccgtatatccttactggcttccgcagaggtcaatccgaatatttcagcatatttagcaacatggatctcgcagataccgtcatgttcctgtagggtgccatcagattttctgatctggtcaacgaacagatacagcatacgtttttgatcccgggagagactatatgccgcctcagtgaggtcgtttgactggacgattcgcgggctatttttacgtttcttgtgattgataaccgctgtttccgccatgacagatccatgtgaagtgtgacaagtttttagattgtcacactaaataaaaaagagtcaataagcagggataactttgtgaaaaaacagcttcttctgagggcaatttgtcacagggttaagggcaatttgtcacagACAGGACTGTCATTTGAGGGTGATTTGTCACACTGAAAGGGCAATTTGTCACAACACCTTCTCTAGAACCAGCATGGATAAAGGCCTACAAGGCGCTCTAAAAAAGAAGATCTAAAAACTATAAAAAAAATAATTATAAAAATATCCCCGTGGATAAGTGGATAACCCCAAGGGAAGTTTTTTCAGGCATCGTGTGTAAGCAGAATATATAAGTGCTGTTCCCTGGTGCTTCCTCGCTCACTCGAGGGCTTCGCCCTGTCGCTCAACTGCGGCGAGCACTACTGGCTGTAAaaggacagaccacatcatggttctgtgttcattaggttgttctgtccattgctgacataatccgctccacttcaacgtaacaccgcacgaagatttctattgttcctgaaggcatattcaaatcgttttcgttaccgcttgcaggcatcatgacagaacactacttcctataaacgctacacaggctcctgagattaataatgcggatctctacgataatgggagattttcccgactgtttcgttcgcttctcagtggataacagccagcttctctgtttaacagacaaaaacagcatatccactcagttccacatttccatataaaggccaaggcatttattctcaggataattgtttcagcatcgcaaccgcatcagactccggcatcgcaaactgcacccggtgccgggcagccacatccagcgcaaaaaccttcgtgtagacttccgttgaactgatggacttatgtcccatcaggctttgcagaactttcagcggtataccggcatacagcatgtgcatcgcataggaatggcggaacgtatgtggtgtgaccggaacagagaacgtcacaccgtcagcagcagcggcggcaaccgcctccccaatccaggtcctgaccgttctgtccgtcacttcccagatccgcgctttctctgtccttcctgtgcgacggttacgccgctccatgagcttatcgcgaataaatacctgtgacggaagatcacttcgcagaataaataaatcctggtgtccctgttgataccgggaagccctgggccaacttttggcgaaaatgagacgttgatcggcacgtaagaggttccaactttcaccataatgaaataagatcactaccgggcgtattttttgagttatcgagattttcaggagctaaggaagctaaaatggagaaaaaaatcactggatataccaccgttgatatatcccaatggcatcgtaaagaacattttgaggcatttcagtcagttgctcaatgtacctataaccagaccgttcagctggatattacggcctttttaaagaccgtaaagaaaaataagcacaagttttatccggcctttattcacattcttgcccgcctgatgaatgctcatccggagttccgtatggcaatgaaagacggtgagctggtgatatgggatagtgttcacccttgttacaccgttttccatgagcaaactgaaacgttttcatcgctctggagtgaataccacgacgatttccggcagtttctacacatatattcgcaagatgtggcgtgttacggtgaaaacctggcctatttccctaaagggtttattgagaatatgtttttcgtctcagccaatccctgggtgagtttcaccagttttgatttaaacgtggccaatatggacaacttcttcgcccccgttttcaccatgggcaaatattatacgcaaggcgacaaggtgctgatgccgctggcgattcaggttcatcatgccgtttgtgatggcttccatgtcggcagaatgcttaatgaattacaacagtactgcgatgagtggcagggcggggcgtaatttttttaaggcagttattggtgcccttaaacgcctggttgctacgcctgaataagtgataataagcggatgaatggcagaaattcgatgataagctgtcaaacatgagaattggtcgacggcgcgccaaagcttgcatgcctgcagccgcgtaacctggcaaaatcggttacggttgagtaataaatggatgccctgcgtaagcggggcacatttcattacctctttctccgcacccgacatagataataacttcgtatagtatacattatacgaagttatctagtagacttaattaaggatcgatccggcgcgccaatagtcatgccccgcgcccaccggaaggagctgactgggttgaaggctctcaagggcatcggtcgagcttgacattgtaggactatattgctctaataaatttgcggccgctaatacgactcactatagggagag |
| pRC1834  (Bp2- Hsmar1 Flag) | gatccgcggaattgTGGTTAATAACAGGGGACGTGGTAATCCGTCCCCTTTTTATTTCTGACTGAGTTAATAACAGGCCTGCTTCGGCAGGCCTTTTTATTTCTGACTGAGTTCTTCTCAGGCCTGCTGGTAATCGCAGGCCTTTTTATTTTCTAGaCAATTCCGACGTCTAAGGAAACCATTATCATGACATCAACCTATAAAAATAGGCGTATCACGAGGCCCTCTCGTCTCCACCTCAAGCTCCCTATCTAGTGATAGCGATTGACATCCCTATCAGTGACGGAGATATTGAGCACATCAGCAGGACGCACTGACCACTTTAAGtctcctATATACATATGGAAATGATGCTCGATAAGAAACAGATTCGTGCGATCTTTCTCTTTGAGTTTAAAATGGGTCGCAAAGCGGCGGAGACGACGCGTAATATTAACAACGCGTTCGGTCCTGGCACCGCGAACGAGCGTACCGTGCAATGGTGGTTCAAAAAGTTTCGCAAAGGCGACGAATCTCTGGAGGACGAAGAGCGTTCTGGCCGCCCGTCCGAGGTTGACAACGACCAGCTGCGTGCAATCATCGAAGCTGATCCGCTGACTACCACCCGCGAAGTTGCTGAAGAACTGAATGTGGATCACTCTACTGTGGTTCGCCACCTGAAACAGATCGGTAAAGTAAAAAAACTGGACAAATGGGTTCCTCATGAACTGTCTGAAAACCAGAAAAACCGTCGTTTCGAAGTTAGCTCCTCTCTGATTCTGCGTAACAACAACGAACCGTTCCTGGATCGTATCGTAACCTGTGATGAGAAATGGATTCTGTATGATAACCGTCGCCGCTCTGCTCAGTGGCTGGATCGCGAAGAAGCTCCAAAACACTTCCCGAAACCGAATCTGCACCAGAAGAAAGTCATGGTAACCGTATGGTGGTCTGCCGCAGGTGTTATCCACTATTCCTTCCTGAACCCGGGCGAAACTATCACCAGCGAAAAATACTGCCAGCAGATTGACGAAATGCACCGTAAACTGCAGCGTCTGCAGCCAGCACTGGTGAATCGTAAAGGTCCGATCCTGCTGCATGATAACGCCCGTCCGCACGTTGCCCAACCGACCCTGCAGAAACTGAACGAACTGGGCTATGAAGTTCTGCCACACCCGCCGTACTCCCCGGATCTGTCCCCGACTGACTACCATTTCTTCAAGCATCTGGACAACTTCCTGCAGGGTAAACGTTTTCACAACCAACAGGACGCAGAAAACGCTTTCCAGGAGTTCGTCGAAAGCCGTTCCACTGACTTCTACGCGACCGGTATCAACAAGCTGATCAGCCGTTGGCAGAAATGCGTGGACTGTAACGGCAGCTACTTCGATGGATCCgactacaaggacgacgatgacaagGAAgattataaagatgatgacgataaaAGAgactacaaggacgacgatgacaagTAAAAGCTTGTTCTTCTCAGGCCTGCTGGTAATCGCAGGCCTTTTTATTTGAATTCcgcggatccttctatagtgtcacctaaatgtcgacggccaggcggccgccaggcctacccactagtcaattcgggaggatcgaaacggcagatcgcaaaaaacagtacatacagaaggagacatgaacatgaacatcaaaaaaattgtaaaacaagccacagttctgacttttacgactgcacttctggcaggaggagcgactcaagccttcgcgaaagaaaataaccaaaaagcatacaaagaaacgtacggcgtctctcatattacacgccatgatatgctgcagatccctaaacagcagcaaaacgaaaaataccaagtgcctcaattcgatcaatcaacgattaaaaatattgagtctgcaaaaggacttgatgtgtgggacagctggccgctgcaaaacgctgacggaacagtagctgaatacaacggctatcacgttgtgtttgctcttgcgggaagcccgaaagacgctgatgacacatcaatctacatgttttatcaaaaggtcggcgacaactcaatcgacagctggaaaaacgcgggccgtgtctttaaagacagcgataagttcgacgccaacgatccgatcctgaaagatcagacgcaagaatggtccggttctgcaacctttacatctgacggaaaaatccgtttattctacactgactattccggtaaacattacggcaaacaaagcctgacaacagcgcaggtaaatgtgtcaaaatctgatgacacactcaaaatcaacggagtggaagatcacaaaacgatttttgacggagacggaaaaacatatcagaacgttcagcagtttatcgatgaaggcaattatacatccggcgacaaccatacgctgagagaccctcactacgttgaagacaaaggccataaataccttgtattcgaagccaacacgggaacagaaaacggataccaaggcgaagaatctttatttaacaaagcgtactacggcggcggcacgaacttcttccgtaaagaaagccagaagcttcagcagagcgctaaaaaacgcgatgctgagttagcgaacggcgccctcggtatcatagagttaaataatgattacacattgaaaaaagtaatgaagccgctgatcacttcaaacacggtaactgatgaaatcgagcgcgcgaatgttttcaaaatgaacggcaaatggtacttgttcactgattcacgcggttcaaaaatgacgatcgatggtattaactcaaacgatatttacatgcttggttatgtatcaaactctttaaccggcccttacaagccgctgaacaaaacagggcttgtgctgcaaatgggtcttgatccaaacgatgtgacattcacttactctcacttcgcagtgccgcaagccaaaggcaacaatgtggttatcacaagctacatgacaaacagaggcttcttcgaggataaaaaggcaacatttgcgccaagcttcttaatgaacatcaaaggcaataaaacatccgttgtcaaaaacagcatcctggagcaaggacagctgacagtcaactaataacagcaaaaagaaaatgccgatacttcattggcattttcttttatttctcaacaagatggtgaattgactagtgggtagatccacaggacgggtgtggtcgccatgatcgcgtagtcgatagtggctccaagtagcgaagcgagcaggactgggcggcggccaaagcggtcggacagtgctccgagaacgggtgcgcatagaaattgcatcaacgcatatagcgctagcagcacgccatagtgactggcgatgctgtcggaatggacgatatcccgcaagaggcccggcagtaccggcataaccaagcctatgcctacagcatccagggtgacggtgccgaggatgacgatgagcgcattgttagatttcatacacggtgcctgactgcgttagcaatttaactgtgataaactaccgcattaaagcttatcgatgataagctgtcaaacatgagaattgatccggaacccttaatataacttcgtataatgtatgctatacgaagttattaggtccctcgactatagggtcaccgtcgacagcgacacacttgcatcggatgcagcccggttaacgtgccggcacggcctgggtaaccaggtattttgtccacataaccgtgcgcaaaatgttgtggataagcaggacacagcagcaatccacagcaggcatacaaccgcacaccgaggttactccgttctacaggttacgacgacatgtcaatacttgcccttgacaggcattgatggaatcgtagtctcacgctgatagtctgatcgacaatacaagtgggaccgtggtcccagaccgataatcagaccgacaacacgagtgggatcgtggtcccagactaataatcagaccgacgatacgagtgggaccgtggtcccagactaataatcagaccgacgatacgagtgggaccgtggttccagactaataatcagaccgacgatacgagtgggaccgtggtcccagactaataatcagaccgacgatacgagtgggaccatggtcccagactaataatcagaccgacgatacgagtgggaccgtggtcccagtctgattatcagaccgacgatacgagtgggaccgtggtcccagactaataatcagaccgacgatacgagtgggaccgtggtcccagactaataatcagaccgacgatacgagtgggaccgtggtcccagtctgattatcagaccgacgatacaagtggaacagtgggcccagagagaatattcaggccagttatgctttctggcctgtaacaaaggacattaagtaaagacagataaacgtagactaaaacgtggtcgcatcagggtgctggcttttcaagttccttaagaatggcctcaattttctctatacactcagttggaacacgagacctgtccaggttaagcaccattttatcgcccttatacaatactgtcgctccaggagcaaactgatgtcgtgagcttaaactagttcttgatgcagatgacgttttaagcacagaagttaaaagagtgataacttcttcagcttcaaatatcaccccagcttttttctgctcatgaaggttagatgcctgctgcttaagtaattcctctttatctgtaaaggctttttgaagtgcatcacctgaccgggcagatagttcaccggggtgagaaaaaagagcaacaactgatttaggcaatttggcggtgttgatacagcgggtaataatcttacgtgaaatattttccgcatcagccagcgcagaaatatttccagcaaattcattctgcaatcggcttgcataacgctgaccacgttcataagcacttgttgggcgataatcgttacccaatctggataatgcagccatctgctcatcatccagctcgccaaccagaacacgataatcactttcggtaagtgcagcagctttacgacggcgactcccatcggcaatttctatgacaccagatactcttcgaccgaacgccggtgtctgttgaccagtcagtagaaaagaagggatgagatcatccagtgcgtcctcagtaagcagctcctggtcacgttcattacctgaccatacccgagaggtcttctcaacactatcaccccggagcacttcaagagtaaacttcacatcccgaccacatacaggcaaagtaatggcattaccgcgagccattactcctacgcgcgcaattaacgaatccaccatcggggcagctggtgtcgataacgaagtatcttcaaccggttgagtattgagcgtatgttttggaataacaggcgcacgcttcattatctaatctcccagcgtggtttaatcagacgatcgaaaatttcattgcagacaggttcccaaatagaaagagcatttctccaggcaccagttgaagagcgttgatcaatggcctgttcaaaaacagttctcatccggatctgacctttaccaacttcatccgtttcacgtacaacattttttagaaccatgcttccccaggcatcccgaatttgctcctccatccacggggactgagagccattactattgctgtatttggtaagcaaaatacgtacatcaggctcgaaccctttaagatcaacgttcttgagcagatcacgaagcatatcgaaaaactgcagtgcggaggtgtagtcaaacaactcagcaggcgtgggaacaatcagcacatcagcagcacatacgacattaatcgtgccgatacccaggttaggcgcgctgtcaataactatgacatcatagtcatgagcaacagtttcaatggccagtcggagcatcaggtgtggatcggtgggcagtttaccttcatcaaatttgcccattaactcagtttcaatacggtgcagagccagacaggaaggaataatgtcaagccccggccagcaagtgggctttattgcataagtgacatcgtccttttccccaagatagaaaggcaggagagtgtcttctgcatgaatatgaagatctggtacccatccgtgatacattgaggctgttccctgggggtcgttaccttccacgagcaaaacacgtagccccttcagagccagatcctgagcaagatgaacagaaactgaggttttgtaaacgccacctttatgggcagcaaccccgatcaccggtggaaatacgtcttcagcacgtcgcaatcgcgtaccaaacacatcacgcatatgattaatttgttcaattgtataaccaacacgttgctcaacccgtcctcgaatttccatatccgggtgcggtagtcgccctgctttctcggcatctctgatagcctgagaagaaaccccaactaaatccgctgcttcacctattctccagcgccgggttattttcctcgcttccgggctgtcatcattaaactgtgcaatggcgatagccttcgtcatttcatgaccagcgtttatgcactggttaagtgtttccatgagtttcattctgaacatcctttaatcattgctttgcgtttttttattaaatcttgcaatttactgcaaagcaacaacaaaatcgcaaagtcatcaaaaaaccgcaaagttgtttaaaataagagcaacactacaaaaggagataagaagagcacatacctcagtcacttattatcactagcgctcgccgcagccgtgtaaccgagcatagcgagcgaactggcgaggaagcaaagaagaactgttctgtcagatagctcttacgctcagcgcaagaagaaatatccaccgtgggaaaaactccaggtagaggtacacacgcggatagccaattcagagtaataaactgtgataatcaaccctcatcaatgatgacgaactaacccccgatatcaggtcacatgacgaagggaaagagaaggaaatcaactgtgacaaactgccctcaaatttggcttccttaaaaattacagttcaaaaagtatgagaaaatccatgcaggctgaaggaaacagcaaaactgtgacaaattaccctcagtaggtcagaacaaatgtgacgaaccaccctcaaatctgtgacagataaccctcagactatcctgtcgtcatggaagtgatatcgcggaaggaaaatacgatatgagtcgtctggcggcctttctttttctcaatgtatgagaggcgcattggagttctgctgttgatctcattaacacagacctgcaggaagcggcggcggaagtcaggcatacgctggtaactttgaggcagctggtaacgctctatgatccagtcgattttcagagagacgatgcctgagccatccggcttacgatactgacacagggattcgtataaacgcatggcatacggattggtgatttcttttgtttcactaagccgaaactgcgtaaaccggttctgtaacccgataaagaagggaatgagatatgggttgatatgtacactgtaaagccctctggatggactgtgcgcacgtttgataaaccaaggaaaagattcatagcctttttcatcgccggcatcctcttcagggcgataaaaaaccacttccttccccgcgaaactcttcaatgcctgccgtatatccttactggcttccgcagaggtcaatccgaatatttcagcatatttagcaacatggatctcgcagataccgtcatgttcctgtagggtgccatcagattttctgatctggtcaacgaacagatacagcatacgtttttgatcccgggagagactatatgccgcctcagtgaggtcgtttgactggacgattcgcgggctatttttacgtttcttgtgattgataaccgctgtttccgccatgacagatccatgtgaagtgtgacaagtttttagattgtcacactaaataaaaaagagtcaataagcagggataactttgtgaaaaaacagcttcttctgagggcaatttgtcacagggttaagggcaatttgtcacagACAGGACTGTCATTTGAGGGTGATTTGTCACACTGAAAGGGCAATTTGTCACAACACCTTCTCTAGAACCAGCATGGATAAAGGCCTACAAGGCGCTCTAAAAAAGAAGATCTAAAAACTATAAAAAAAATAATTATAAAAATATCCCCGTGGATAAGTGGATAACCCCAAGGGAAGTTTTTTCAGGCATCGTGTGTAAGCAGAATATATAAGTGCTGTTCCCTGGTGCTTCCTCGCTCACTCGAGGGCTTCGCCCTGTCGCTCAACTGCGGCGAGCACTACTGGCTGTAAaaggacagaccacatcatggttctgtgttcattaggttgttctgtccattgctgacataatccgctccacttcaacgtaacaccgcacgaagatttctattgttcctgaaggcatattcaaatcgttttcgttaccgcttgcaggcatcatgacagaacactacttcctataaacgctacacaggctcctgagattaataatgcggatctctacgataatgggagattttcccgactgtttcgttcgcttctcagtggataacagccagcttctctgtttaacagacaaaaacagcatatccactcagttccacatttccatataaaggccaaggcatttattctcaggataattgtttcagcatcgcaaccgcatcagactccggcatcgcaaactgcacccggtgccgggcagccacatccagcgcaaaaaccttcgtgtagacttccgttgaactgatggacttatgtcccatcaggctttgcagaactttcagcggtataccggcatacagcatgtgcatcgcataggaatggcggaacgtatgtggtgtgaccggaacagagaacgtcacaccgtcagcagcagcggcggcaaccgcctccccaatccaggtcctgaccgttctgtccgtcacttcccagatccgcgctttctctgtccttcctgtgcgacggttacgccgctccatgagcttatcgcgaataaatacctgtgacggaagatcacttcgcagaataaataaatcctggtgtccctgttgataccgggaagccctgggccaacttttggcgaaaatgagacgttgatcggcacgtaagaggttccaactttcaccataatgaaataagatcactaccgggcgtattttttgagttatcgagattttcaggagctaaggaagctaaaatggagaaaaaaatcactggatataccaccgttgatatatcccaatggcatcgtaaagaacattttgaggcatttcagtcagttgctcaatgtacctataaccagaccgttcagctggatattacggcctttttaaagaccgtaaagaaaaataagcacaagttttatccggcctttattcacattcttgcccgcctgatgaatgctcatccggagttccgtatggcaatgaaagacggtgagctggtgatatgggatagtgttcacccttgttacaccgttttccatgagcaaactgaaacgttttcatcgctctggagtgaataccacgacgatttccggcagtttctacacatatattcgcaagatgtggcgtgttacggtgaaaacctggcctatttccctaaagggtttattgagaatatgtttttcgtctcagccaatccctgggtgagtttcaccagttttgatttaaacgtggccaatatggacaacttcttcgcccccgttttcaccatgggcaaatattatacgcaaggcgacaaggtgctgatgccgctggcgattcaggttcatcatgccgtttgtgatggcttccatgtcggcagaatgcttaatgaattacaacagtactgcgatgagtggcagggcggggcgtaatttttttaaggcagttattggtgcccttaaacgcctggttgctacgcctgaataagtgataataagcggatgaatggcagaaattcgatgataagctgtcaaacatgagaattggtcgacggcgcgccaaagcttgcatgcctgcagccgcgtaacctggcaaaatcggttacggttgagtaataaatggatgccctgcgtaagcggggcacatttcattacctctttctccgcacccgacatagataataacttcgtatagtatacattatacgaagttatctagtagacttaattaaggatcgatccggcgcgccaatagtcatgccccgcgcccaccggaaggagctgactgggttgaaggctctcaagggcatcggtcgagcttgacattgtaggactatattgctctaataaatttgcggccgctaatacgactcactatagggagag |
| pRC1835  (Bp3- Hsmar1 Flag) | gatccgcggaattgTGGTTAATAACAGGGGACGTGGTAATCCGTCCCCTTTTTATTTCTGACTGAGTTAATAACAGGCCTGCTTCGGCAGGCCTTTTTATTTCTGACTGAGTTCTTCTCAGGCCTGCTGGTAATCGCAGGCCTTTTTATTTTCTAGaCAATTCCGACGTCTAAGAAACCATTATTATCATGACATTAACCTATAAAAATAGGCGTATCACGAGGCCCTTTCGTCTTCACCTCGAGTCCCTATCAGTGATAGAGATTGACCTCCCTATCAGTGATAGAGATACTGAGCACATCAGCAGGACGCACTGACCACTTTAAGtctcctATATACATATGGAAATGATGCTCGATAAGAAACAGATTCGTGCGATCTTTCTCTTTGAGTTTAAAATGGGTCGCAAAGCGGCGGAGACGACGCGTAATATTAACAACGCGTTCGGTCCTGGCACCGCGAACGAGCGTACCGTGCAATGGTGGTTCAAAAAGTTTCGCAAAGGCGACGAATCTCTGGAGGACGAAGAGCGTTCTGGCCGCCCGTCCGAGGTTGACAACGACCAGCTGCGTGCAATCATCGAAGCTGATCCGCTGACTACCACCCGCGAAGTTGCTGAAGAACTGAATGTGGATCACTCTACTGTGGTTCGCCACCTGAAACAGATCGGTAAAGTAAAAAAACTGGACAAATGGGTTCCTCATGAACTGTCTGAAAACCAGAAAAACCGTCGTTTCGAAGTTAGCTCCTCTCTGATTCTGCGTAACAACAACGAACCGTTCCTGGATCGTATCGTAACCTGTGATGAGAAATGGATTCTGTATGATAACCGTCGCCGCTCTGCTCAGTGGCTGGATCGCGAAGAAGCTCCAAAACACTTCCCGAAACCGAATCTGCACCAGAAGAAAGTCATGGTAACCGTATGGTGGTCTGCCGCAGGTGTTATCCACTATTCCTTCCTGAACCCGGGCGAAACTATCACCAGCGAAAAATACTGCCAGCAGATTGACGAAATGCACCGTAAACTGCAGCGTCTGCAGCCAGCACTGGTGAATCGTAAAGGTCCGATCCTGCTGCATGATAACGCCCGTCCGCACGTTGCCCAACCGACCCTGCAGAAACTGAACGAACTGGGCTATGAAGTTCTGCCACACCCGCCGTACTCCCCGGATCTGTCCCCGACTGACTACCATTTCTTCAAGCATCTGGACAACTTCCTGCAGGGTAAACGTTTTCACAACCAACAGGACGCAGAAAACGCTTTCCAGGAGTTCGTCGAAAGCCGTTCCACTGACTTCTACGCGACCGGTATCAACAAGCTGATCAGCCGTTGGCAGAAATGCGTGGACTGTAACGGCAGCTACTTCGATGGATCCgactacaaggacgacgatgacaagGAAgattataaagatgatgacgataaaAGAgactacaaggacgacgatgacaagTAAAAGCTTGTTCTTCTCAGGCCTGCTGGTAATCGCAGGCCTTTTTATTTGAATTCcgcggatccttctatagtgtcacctaaatgtcgacggccaggcggccgccaggcctacccactagtcaattcgggaggatcgaaacggcagatcgcaaaaaacagtacatacagaaggagacatgaacatgaacatcaaaaaaattgtaaaacaagccacagttctgacttttacgactgcacttctggcaggaggagcgactcaagccttcgcgaaagaaaataaccaaaaagcatacaaagaaacgtacggcgtctctcatattacacgccatgatatgctgcagatccctaaacagcagcaaaacgaaaaataccaagtgcctcaattcgatcaatcaacgattaaaaatattgagtctgcaaaaggacttgatgtgtgggacagctggccgctgcaaaacgctgacggaacagtagctgaatacaacggctatcacgttgtgtttgctcttgcgggaagcccgaaagacgctgatgacacatcaatctacatgttttatcaaaaggtcggcgacaactcaatcgacagctggaaaaacgcgggccgtgtctttaaagacagcgataagttcgacgccaacgatccgatcctgaaagatcagacgcaagaatggtccggttctgcaacctttacatctgacggaaaaatccgtttattctacactgactattccggtaaacattacggcaaacaaagcctgacaacagcgcaggtaaatgtgtcaaaatctgatgacacactcaaaatcaacggagtggaagatcacaaaacgatttttgacggagacggaaaaacatatcagaacgttcagcagtttatcgatgaaggcaattatacatccggcgacaaccatacgctgagagaccctcactacgttgaagacaaaggccataaataccttgtattcgaagccaacacgggaacagaaaacggataccaaggcgaagaatctttatttaacaaagcgtactacggcggcggcacgaacttcttccgtaaagaaagccagaagcttcagcagagcgctaaaaaacgcgatgctgagttagcgaacggcgccctcggtatcatagagttaaataatgattacacattgaaaaaagtaatgaagccgctgatcacttcaaacacggtaactgatgaaatcgagcgcgcgaatgttttcaaaatgaacggcaaatggtacttgttcactgattcacgcggttcaaaaatgacgatcgatggtattaactcaaacgatatttacatgcttggttatgtatcaaactctttaaccggcccttacaagccgctgaacaaaacagggcttgtgctgcaaatgggtcttgatccaaacgatgtgacattcacttactctcacttcgcagtgccgcaagccaaaggcaacaatgtggttatcacaagctacatgacaaacagaggcttcttcgaggataaaaaggcaacatttgcgccaagcttcttaatgaacatcaaaggcaataaaacatccgttgtcaaaaacagcatcctggagcaaggacagctgacagtcaactaataacagcaaaaagaaaatgccgatacttcattggcattttcttttatttctcaacaagatggtgaattgactagtgggtagatccacaggacgggtgtggtcgccatgatcgcgtagtcgatagtggctccaagtagcgaagcgagcaggactgggcggcggccaaagcggtcggacagtgctccgagaacgggtgcgcatagaaattgcatcaacgcatatagcgctagcagcacgccatagtgactggcgatgctgtcggaatggacgatatcccgcaagaggcccggcagtaccggcataaccaagcctatgcctacagcatccagggtgacggtgccgaggatgacgatgagcgcattgttagatttcatacacggtgcctgactgcgttagcaatttaactgtgataaactaccgcattaaagcttatcgatgataagctgtcaaacatgagaattgatccggaacccttaatataacttcgtataatgtatgctatacgaagttattaggtccctcgactatagggtcaccgtcgacagcgacacacttgcatcggatgcagcccggttaacgtgccggcacggcctgggtaaccaggtattttgtccacataaccgtgcgcaaaatgttgtggataagcaggacacagcagcaatccacagcaggcatacaaccgcacaccgaggttactccgttctacaggttacgacgacatgtcaatacttgcccttgacaggcattgatggaatcgtagtctcacgctgatagtctgatcgacaatacaagtgggaccgtggtcccagaccgataatcagaccgacaacacgagtgggatcgtggtcccagactaataatcagaccgacgatacgagtgggaccgtggtcccagactaataatcagaccgacgatacgagtgggaccgtggttccagactaataatcagaccgacgatacgagtgggaccgtggtcccagactaataatcagaccgacgatacgagtgggaccatggtcccagactaataatcagaccgacgatacgagtgggaccgtggtcccagtctgattatcagaccgacgatacgagtgggaccgtggtcccagactaataatcagaccgacgatacgagtgggaccgtggtcccagactaataatcagaccgacgatacgagtgggaccgtggtcccagtctgattatcagaccgacgatacaagtggaacagtgggcccagagagaatattcaggccagttatgctttctggcctgtaacaaaggacattaagtaaagacagataaacgtagactaaaacgtggtcgcatcagggtgctggcttttcaagttccttaagaatggcctcaattttctctatacactcagttggaacacgagacctgtccaggttaagcaccattttatcgcccttatacaatactgtcgctccaggagcaaactgatgtcgtgagcttaaactagttcttgatgcagatgacgttttaagcacagaagttaaaagagtgataacttcttcagcttcaaatatcaccccagcttttttctgctcatgaaggttagatgcctgctgcttaagtaattcctctttatctgtaaaggctttttgaagtgcatcacctgaccgggcagatagttcaccggggtgagaaaaaagagcaacaactgatttaggcaatttggcggtgttgatacagcgggtaataatcttacgtgaaatattttccgcatcagccagcgcagaaatatttccagcaaattcattctgcaatcggcttgcataacgctgaccacgttcataagcacttgttgggcgataatcgttacccaatctggataatgcagccatctgctcatcatccagctcgccaaccagaacacgataatcactttcggtaagtgcagcagctttacgacggcgactcccatcggcaatttctatgacaccagatactcttcgaccgaacgccggtgtctgttgaccagtcagtagaaaagaagggatgagatcatccagtgcgtcctcagtaagcagctcctggtcacgttcattacctgaccatacccgagaggtcttctcaacactatcaccccggagcacttcaagagtaaacttcacatcccgaccacatacaggcaaagtaatggcattaccgcgagccattactcctacgcgcgcaattaacgaatccaccatcggggcagctggtgtcgataacgaagtatcttcaaccggttgagtattgagcgtatgttttggaataacaggcgcacgcttcattatctaatctcccagcgtggtttaatcagacgatcgaaaatttcattgcagacaggttcccaaatagaaagagcatttctccaggcaccagttgaagagcgttgatcaatggcctgttcaaaaacagttctcatccggatctgacctttaccaacttcatccgtttcacgtacaacattttttagaaccatgcttccccaggcatcccgaatttgctcctccatccacggggactgagagccattactattgctgtatttggtaagcaaaatacgtacatcaggctcgaaccctttaagatcaacgttcttgagcagatcacgaagcatatcgaaaaactgcagtgcggaggtgtagtcaaacaactcagcaggcgtgggaacaatcagcacatcagcagcacatacgacattaatcgtgccgatacccaggttaggcgcgctgtcaataactatgacatcatagtcatgagcaacagtttcaatggccagtcggagcatcaggtgtggatcggtgggcagtttaccttcatcaaatttgcccattaactcagtttcaatacggtgcagagccagacaggaaggaataatgtcaagccccggccagcaagtgggctttattgcataagtgacatcgtccttttccccaagatagaaaggcaggagagtgtcttctgcatgaatatgaagatctggtacccatccgtgatacattgaggctgttccctgggggtcgttaccttccacgagcaaaacacgtagccccttcagagccagatcctgagcaagatgaacagaaactgaggttttgtaaacgccacctttatgggcagcaaccccgatcaccggtggaaatacgtcttcagcacgtcgcaatcgcgtaccaaacacatcacgcatatgattaatttgttcaattgtataaccaacacgttgctcaacccgtcctcgaatttccatatccgggtgcggtagtcgccctgctttctcggcatctctgatagcctgagaagaaaccccaactaaatccgctgcttcacctattctccagcgccgggttattttcctcgcttccgggctgtcatcattaaactgtgcaatggcgatagccttcgtcatttcatgaccagcgtttatgcactggttaagtgtttccatgagtttcattctgaacatcctttaatcattgctttgcgtttttttattaaatcttgcaatttactgcaaagcaacaacaaaatcgcaaagtcatcaaaaaaccgcaaagttgtttaaaataagagcaacactacaaaaggagataagaagagcacatacctcagtcacttattatcactagcgctcgccgcagccgtgtaaccgagcatagcgagcgaactggcgaggaagcaaagaagaactgttctgtcagatagctcttacgctcagcgcaagaagaaatatccaccgtgggaaaaactccaggtagaggtacacacgcggatagccaattcagagtaataaactgtgataatcaaccctcatcaatgatgacgaactaacccccgatatcaggtcacatgacgaagggaaagagaaggaaatcaactgtgacaaactgccctcaaatttggcttccttaaaaattacagttcaaaaagtatgagaaaatccatgcaggctgaaggaaacagcaaaactgtgacaaattaccctcagtaggtcagaacaaatgtgacgaaccaccctcaaatctgtgacagataaccctcagactatcctgtcgtcatggaagtgatatcgcggaaggaaaatacgatatgagtcgtctggcggcctttctttttctcaatgtatgagaggcgcattggagttctgctgttgatctcattaacacagacctgcaggaagcggcggcggaagtcaggcatacgctggtaactttgaggcagctggtaacgctctatgatccagtcgattttcagagagacgatgcctgagccatccggcttacgatactgacacagggattcgtataaacgcatggcatacggattggtgatttcttttgtttcactaagccgaaactgcgtaaaccggttctgtaacccgataaagaagggaatgagatatgggttgatatgtacactgtaaagccctctggatggactgtgcgcacgtttgataaaccaaggaaaagattcatagcctttttcatcgccggcatcctcttcagggcgataaaaaaccacttccttccccgcgaaactcttcaatgcctgccgtatatccttactggcttccgcagaggtcaatccgaatatttcagcatatttagcaacatggatctcgcagataccgtcatgttcctgtagggtgccatcagattttctgatctggtcaacgaacagatacagcatacgtttttgatcccgggagagactatatgccgcctcagtgaggtcgtttgactggacgattcgcgggctatttttacgtttcttgtgattgataaccgctgtttccgccatgacagatccatgtgaagtgtgacaagtttttagattgtcacactaaataaaaaagagtcaataagcagggataactttgtgaaaaaacagcttcttctgagggcaatttgtcacagggttaagggcaatttgtcacagACAGGACTGTCATTTGAGGGTGATTTGTCACACTGAAAGGGCAATTTGTCACAACACCTTCTCTAGAACCAGCATGGATAAAGGCCTACAAGGCGCTCTAAAAAAGAAGATCTAAAAACTATAAAAAAAATAATTATAAAAATATCCCCGTGGATAAGTGGATAACCCCAAGGGAAGTTTTTTCAGGCATCGTGTGTAAGCAGAATATATAAGTGCTGTTCCCTGGTGCTTCCTCGCTCACTCGAGGGCTTCGCCCTGTCGCTCAACTGCGGCGAGCACTACTGGCTGTAAaaggacagaccacatcatggttctgtgttcattaggttgttctgtccattgctgacataatccgctccacttcaacgtaacaccgcacgaagatttctattgttcctgaaggcatattcaaatcgttttcgttaccgcttgcaggcatcatgacagaacactacttcctataaacgctacacaggctcctgagattaataatgcggatctctacgataatgggagattttcccgactgtttcgttcgcttctcagtggataacagccagcttctctgtttaacagacaaaaacagcatatccactcagttccacatttccatataaaggccaaggcatttattctcaggataattgtttcagcatcgcaaccgcatcagactccggcatcgcaaactgcacccggtgccgggcagccacatccagcgcaaaaaccttcgtgtagacttccgttgaactgatggacttatgtcccatcaggctttgcagaactttcagcggtataccggcatacagcatgtgcatcgcataggaatggcggaacgtatgtggtgtgaccggaacagagaacgtcacaccgtcagcagcagcggcggcaaccgcctccccaatccaggtcctgaccgttctgtccgtcacttcccagatccgcgctttctctgtccttcctgtgcgacggttacgccgctccatgagcttatcgcgaataaatacctgtgacggaagatcacttcgcagaataaataaatcctggtgtccctgttgataccgggaagccctgggccaacttttggcgaaaatgagacgttgatcggcacgtaagaggttccaactttcaccataatgaaataagatcactaccgggcgtattttttgagttatcgagattttcaggagctaaggaagctaaaatggagaaaaaaatcactggatataccaccgttgatatatcccaatggcatcgtaaagaacattttgaggcatttcagtcagttgctcaatgtacctataaccagaccgttcagctggatattacggcctttttaaagaccgtaaagaaaaataagcacaagttttatccggcctttattcacattcttgcccgcctgatgaatgctcatccggagttccgtatggcaatgaaagacggtgagctggtgatatgggatagtgttcacccttgttacaccgttttccatgagcaaactgaaacgttttcatcgctctggagtgaataccacgacgatttccggcagtttctacacatatattcgcaagatgtggcgtgttacggtgaaaacctggcctatttccctaaagggtttattgagaatatgtttttcgtctcagccaatccctgggtgagtttcaccagttttgatttaaacgtggccaatatggacaacttcttcgcccccgttttcaccatgggcaaatattatacgcaaggcgacaaggtgctgatgccgctggcgattcaggttcatcatgccgtttgtgatggcttccatgtcggcagaatgcttaatgaattacaacagtactgcgatgagtggcagggcggggcgtaatttttttaaggcagttattggtgcccttaaacgcctggttgctacgcctgaataagtgataataagcggatgaatggcagaaattcgatgataagctgtcaaacatgagaattggtcgacggcgcgccaaagcttgcatgcctgcagccgcgtaacctggcaaaatcggttacggttgagtaataaatggatgccctgcgtaagcggggcacatttcattacctctttctccgcacccgacatagataataacttcgtatagtatacattatacgaagttatctagtagacttaattaaggatcgatccggcgcgccaatagtcatgccccgcgcccaccggaaggagctgactgggttgaaggctctcaagggcatcggtcgagcttgacattgtaggactatattgctctaataaatttgcggccgctaatacgactcactatagggagag |
| pRC1836  (Bp4- Hsmar1 Flag) | gatccgcggaattgTGGTTAATAACAGGGGACGTGGTAATCCGTCCCCTTTTTATTTCTGACTGAGTTAATAACAGGCCTGCTTCGGCAGGCCTTTTTATTTCTGACTGAGTTCTTCTCAGGCCTGCTGGTAATCGCAGGCCTTTTTATTTTCTAGgCAATTCCGACGTCTAAGAAACCATTATTATCATGACATTAACCTATAAAAATAGGCGTATCACGAGGCCCTCTCGTCTTCACCTCGAGTCCCTATCAGTGATAGGGATTGACATCCCTATCAGTGATAGAGACACTGGGCACATCAGCAGGACGCACTGACCACTTTAAGtctcctATATACATATGGAAATGATGCTCGATAAGAAACAGATTCGTGCGATCTTTCTCTTTGAGTTTAAAATGGGTCGCAAAGCGGCGGAGACGACGCGTAATATTAACAACGCGTTCGGTCCTGGCACCGCGAACGAGCGTACCGTGCAATGGTGGTTCAAAAAGTTTCGCAAAGGCGACGAATCTCTGGAGGACGAAGAGCGTTCTGGCCGCCCGTCCGAGGTTGACAACGACCAGCTGCGTGCAATCATCGAAGCTGATCCGCTGACTACCACCCGCGAAGTTGCTGAAGAACTGAATGTGGATCACTCTACTGTGGTTCGCCACCTGAAACAGATCGGTAAAGTAAAAAAACTGGACAAATGGGTTCCTCATGAACTGTCTGAAAACCAGAAAAACCGTCGTTTCGAAGTTAGCTCCTCTCTGATTCTGCGTAACAACAACGAACCGTTCCTGGATCGTATCGTAACCTGTGATGAGAAATGGATTCTGTATGATAACCGTCGCCGCTCTGCTCAGTGGCTGGATCGCGAAGAAGCTCCAAAACACTTCCCGAAACCGAATCTGCACCAGAAGAAAGTCATGGTAACCGTATGGTGGTCTGCCGCAGGTGTTATCCACTATTCCTTCCTGAACCCGGGCGAAACTATCACCAGCGAAAAATACTGCCAGCAGATTGACGAAATGCACCGTAAACTGCAGCGTCTGCAGCCAGCACTGGTGAATCGTAAAGGTCCGATCCTGCTGCATGATAACGCCCGTCCGCACGTTGCCCAACCGACCCTGCAGAAACTGAACGAACTGGGCTATGAAGTTCTGCCACACCCGCCGTACTCCCCGGATCTGTCCCCGACTGACTACCATTTCTTCAAGCATCTGGACAACTTCCTGCAGGGTAAACGTTTTCACAACCAACAGGACGCAGAAAACGCTTTCCAGGAGTTCGTCGAAAGCCGTTCCACTGACTTCTACGCGACCGGTATCAACAAGCTGATCAGCCGTTGGCAGAAATGCGTGGACTGTAACGGCAGCTACTTCGATGGATCCgactacaaggacgacgatgacaagGAAgattataaagatgatgacgataaaAGAgactacaaggacgacgatgacaagTAAAAGCTTGTTCTTCTCAGGCCTGCTGGTAATCGCAGGCCTTTTTATTTGAATTCcgcggatccttctatagtgtcacctaaatgtcgacggccaggcggccgccaggcctacccactagtcaattcgggaggatcgaaacggcagatcgcaaaaaacagtacatacagaaggagacatgaacatgaacatcaaaaaaattgtaaaacaagccacagttctgacttttacgactgcacttctggcaggaggagcgactcaagccttcgcgaaagaaaataaccaaaaagcatacaaagaaacgtacggcgtctctcatattacacgccatgatatgctgcagatccctaaacagcagcaaaacgaaaaataccaagtgcctcaattcgatcaatcaacgattaaaaatattgagtctgcaaaaggacttgatgtgtgggacagctggccgctgcaaaacgctgacggaacagtagctgaatacaacggctatcacgttgtgtttgctcttgcgggaagcccgaaagacgctgatgacacatcaatctacatgttttatcaaaaggtcggcgacaactcaatcgacagctggaaaaacgcgggccgtgtctttaaagacagcgataagttcgacgccaacgatccgatcctgaaagatcagacgcaagaatggtccggttctgcaacctttacatctgacggaaaaatccgtttattctacactgactattccggtaaacattacggcaaacaaagcctgacaacagcgcaggtaaatgtgtcaaaatctgatgacacactcaaaatcaacggagtggaagatcacaaaacgatttttgacggagacggaaaaacatatcagaacgttcagcagtttatcgatgaaggcaattatacatccggcgacaaccatacgctgagagaccctcactacgttgaagacaaaggccataaataccttgtattcgaagccaacacgggaacagaaaacggataccaaggcgaagaatctttatttaacaaagcgtactacggcggcggcacgaacttcttccgtaaagaaagccagaagcttcagcagagcgctaaaaaacgcgatgctgagttagcgaacggcgccctcggtatcatagagttaaataatgattacacattgaaaaaagtaatgaagccgctgatcacttcaaacacggtaactgatgaaatcgagcgcgcgaatgttttcaaaatgaacggcaaatggtacttgttcactgattcacgcggttcaaaaatgacgatcgatggtattaactcaaacgatatttacatgcttggttatgtatcaaactctttaaccggcccttacaagccgctgaacaaaacagggcttgtgctgcaaatgggtcttgatccaaacgatgtgacattcacttactctcacttcgcagtgccgcaagccaaaggcaacaatgtggttatcacaagctacatgacaaacagaggcttcttcgaggataaaaaggcaacatttgcgccaagcttcttaatgaacatcaaaggcaataaaacatccgttgtcaaaaacagcatcctggagcaaggacagctgacagtcaactaataacagcaaaaagaaaatgccgatacttcattggcattttcttttatttctcaacaagatggtgaattgactagtgggtagatccacaggacgggtgtggtcgccatgatcgcgtagtcgatagtggctccaagtagcgaagcgagcaggactgggcggcggccaaagcggtcggacagtgctccgagaacgggtgcgcatagaaattgcatcaacgcatatagcgctagcagcacgccatagtgactggcgatgctgtcggaatggacgatatcccgcaagaggcccggcagtaccggcataaccaagcctatgcctacagcatccagggtgacggtgccgaggatgacgatgagcgcattgttagatttcatacacggtgcctgactgcgttagcaatttaactgtgataaactaccgcattaaagcttatcgatgataagctgtcaaacatgagaattgatccggaacccttaatataacttcgtataatgtatgctatacgaagttattaggtccctcgactatagggtcaccgtcgacagcgacacacttgcatcggatgcagcccggttaacgtgccggcacggcctgggtaaccaggtattttgtccacataaccgtgcgcaaaatgttgtggataagcaggacacagcagcaatccacagcaggcatacaaccgcacaccgaggttactccgttctacaggttacgacgacatgtcaatacttgcccttgacaggcattgatggaatcgtagtctcacgctgatagtctgatcgacaatacaagtgggaccgtggtcccagaccgataatcagaccgacaacacgagtgggatcgtggtcccagactaataatcagaccgacgatacgagtgggaccgtggtcccagactaataatcagaccgacgatacgagtgggaccgtggttccagactaataatcagaccgacgatacgagtgggaccgtggtcccagactaataatcagaccgacgatacgagtgggaccatggtcccagactaataatcagaccgacgatacgagtgggaccgtggtcccagtctgattatcagaccgacgatacgagtgggaccgtggtcccagactaataatcagaccgacgatacgagtgggaccgtggtcccagactaataatcagaccgacgatacgagtgggaccgtggtcccagtctgattatcagaccgacgatacaagtggaacagtgggcccagagagaatattcaggccagttatgctttctggcctgtaacaaaggacattaagtaaagacagataaacgtagactaaaacgtggtcgcatcagggtgctggcttttcaagttccttaagaatggcctcaattttctctatacactcagttggaacacgagacctgtccaggttaagcaccattttatcgcccttatacaatactgtcgctccaggagcaaactgatgtcgtgagcttaaactagttcttgatgcagatgacgttttaagcacagaagttaaaagagtgataacttcttcagcttcaaatatcaccccagcttttttctgctcatgaaggttagatgcctgctgcttaagtaattcctctttatctgtaaaggctttttgaagtgcatcacctgaccgggcagatagttcaccggggtgagaaaaaagagcaacaactgatttaggcaatttggcggtgttgatacagcgggtaataatcttacgtgaaatattttccgcatcagccagcgcagaaatatttccagcaaattcattctgcaatcggcttgcataacgctgaccacgttcataagcacttgttgggcgataatcgttacccaatctggataatgcagccatctgctcatcatccagctcgccaaccagaacacgataatcactttcggtaagtgcagcagctttacgacggcgactcccatcggcaatttctatgacaccagatactcttcgaccgaacgccggtgtctgttgaccagtcagtagaaaagaagggatgagatcatccagtgcgtcctcagtaagcagctcctggtcacgttcattacctgaccatacccgagaggtcttctcaacactatcaccccggagcacttcaagagtaaacttcacatcccgaccacatacaggcaaagtaatggcattaccgcgagccattactcctacgcgcgcaattaacgaatccaccatcggggcagctggtgtcgataacgaagtatcttcaaccggttgagtattgagcgtatgttttggaataacaggcgcacgcttcattatctaatctcccagcgtggtttaatcagacgatcgaaaatttcattgcagacaggttcccaaatagaaagagcatttctccaggcaccagttgaagagcgttgatcaatggcctgttcaaaaacagttctcatccggatctgacctttaccaacttcatccgtttcacgtacaacattttttagaaccatgcttccccaggcatcccgaatttgctcctccatccacggggactgagagccattactattgctgtatttggtaagcaaaatacgtacatcaggctcgaaccctttaagatcaacgttcttgagcagatcacgaagcatatcgaaaaactgcagtgcggaggtgtagtcaaacaactcagcaggcgtgggaacaatcagcacatcagcagcacatacgacattaatcgtgccgatacccaggttaggcgcgctgtcaataactatgacatcatagtcatgagcaacagtttcaatggccagtcggagcatcaggtgtggatcggtgggcagtttaccttcatcaaatttgcccattaactcagtttcaatacggtgcagagccagacaggaaggaataatgtcaagccccggccagcaagtgggctttattgcataagtgacatcgtccttttccccaagatagaaaggcaggagagtgtcttctgcatgaatatgaagatctggtacccatccgtgatacattgaggctgttccctgggggtcgttaccttccacgagcaaaacacgtagccccttcagagccagatcctgagcaagatgaacagaaactgaggttttgtaaacgccacctttatgggcagcaaccccgatcaccggtggaaatacgtcttcagcacgtcgcaatcgcgtaccaaacacatcacgcatatgattaatttgttcaattgtataaccaacacgttgctcaacccgtcctcgaatttccatatccgggtgcggtagtcgccctgctttctcggcatctctgatagcctgagaagaaaccccaactaaatccgctgcttcacctattctccagcgccgggttattttcctcgcttccgggctgtcatcattaaactgtgcaatggcgatagccttcgtcatttcatgaccagcgtttatgcactggttaagtgtttccatgagtttcattctgaacatcctttaatcattgctttgcgtttttttattaaatcttgcaatttactgcaaagcaacaacaaaatcgcaaagtcatcaaaaaaccgcaaagttgtttaaaataagagcaacactacaaaaggagataagaagagcacatacctcagtcacttattatcactagcgctcgccgcagccgtgtaaccgagcatagcgagcgaactggcgaggaagcaaagaagaactgttctgtcagatagctcttacgctcagcgcaagaagaaatatccaccgtgggaaaaactccaggtagaggtacacacgcggatagccaattcagagtaataaactgtgataatcaaccctcatcaatgatgacgaactaacccccgatatcaggtcacatgacgaagggaaagagaaggaaatcaactgtgacaaactgccctcaaatttggcttccttaaaaattacagttcaaaaagtatgagaaaatccatgcaggctgaaggaaacagcaaaactgtgacaaattaccctcagtaggtcagaacaaatgtgacgaaccaccctcaaatctgtgacagataaccctcagactatcctgtcgtcatggaagtgatatcgcggaaggaaaatacgatatgagtcgtctggcggcctttctttttctcaatgtatgagaggcgcattggagttctgctgttgatctcattaacacagacctgcaggaagcggcggcggaagtcaggcatacgctggtaactttgaggcagctggtaacgctctatgatccagtcgattttcagagagacgatgcctgagccatccggcttacgatactgacacagggattcgtataaacgcatggcatacggattggtgatttcttttgtttcactaagccgaaactgcgtaaaccggttctgtaacccgataaagaagggaatgagatatgggttgatatgtacactgtaaagccctctggatggactgtgcgcacgtttgataaaccaaggaaaagattcatagcctttttcatcgccggcatcctcttcagggcgataaaaaaccacttccttccccgcgaaactcttcaatgcctgccgtatatccttactggcttccgcagaggtcaatccgaatatttcagcatatttagcaacatggatctcgcagataccgtcatgttcctgtagggtgccatcagattttctgatctggtcaacgaacagatacagcatacgtttttgatcccgggagagactatatgccgcctcagtgaggtcgtttgactggacgattcgcgggctatttttacgtttcttgtgattgataaccgctgtttccgccatgacagatccatgtgaagtgtgacaagtttttagattgtcacactaaataaaaaagagtcaataagcagggataactttgtgaaaaaacagcttcttctgagggcaatttgtcacagggttaagggcaatttgtcacagACAGGACTGTCATTTGAGGGTGATTTGTCACACTGAAAGGGCAATTTGTCACAACACCTTCTCTAGAACCAGCATGGATAAAGGCCTACAAGGCGCTCTAAAAAAGAAGATCTAAAAACTATAAAAAAAATAATTATAAAAATATCCCCGTGGATAAGTGGATAACCCCAAGGGAAGTTTTTTCAGGCATCGTGTGTAAGCAGAATATATAAGTGCTGTTCCCTGGTGCTTCCTCGCTCACTCGAGGGCTTCGCCCTGTCGCTCAACTGCGGCGAGCACTACTGGCTGTAAaaggacagaccacatcatggttctgtgttcattaggttgttctgtccattgctgacataatccgctccacttcaacgtaacaccgcacgaagatttctattgttcctgaaggcatattcaaatcgttttcgttaccgcttgcaggcatcatgacagaacactacttcctataaacgctacacaggctcctgagattaataatgcggatctctacgataatgggagattttcccgactgtttcgttcgcttctcagtggataacagccagcttctctgtttaacagacaaaaacagcatatccactcagttccacatttccatataaaggccaaggcatttattctcaggataattgtttcagcatcgcaaccgcatcagactccggcatcgcaaactgcacccggtgccgggcagccacatccagcgcaaaaaccttcgtgtagacttccgttgaactgatggacttatgtcccatcaggctttgcagaactttcagcggtataccggcatacagcatgtgcatcgcataggaatggcggaacgtatgtggtgtgaccggaacagagaacgtcacaccgtcagcagcagcggcggcaaccgcctccccaatccaggtcctgaccgttctgtccgtcacttcccagatccgcgctttctctgtccttcctgtgcgacggttacgccgctccatgagcttatcgcgaataaatacctgtgacggaagatcacttcgcagaataaataaatcctggtgtccctgttgataccgggaagccctgggccaacttttggcgaaaatgagacgttgatcggcacgtaagaggttccaactttcaccataatgaaataagatcactaccgggcgtattttttgagttatcgagattttcaggagctaaggaagctaaaatggagaaaaaaatcactggatataccaccgttgatatatcccaatggcatcgtaaagaacattttgaggcatttcagtcagttgctcaatgtacctataaccagaccgttcagctggatattacggcctttttaaagaccgtaaagaaaaataagcacaagttttatccggcctttattcacattcttgcccgcctgatgaatgctcatccggagttccgtatggcaatgaaagacggtgagctggtgatatgggatagtgttcacccttgttacaccgttttccatgagcaaactgaaacgttttcatcgctctggagtgaataccacgacgatttccggcagtttctacacatatattcgcaagatgtggcgtgttacggtgaaaacctggcctatttccctaaagggtttattgagaatatgtttttcgtctcagccaatccctgggtgagtttcaccagttttgatttaaacgtggccaatatggacaacttcttcgcccccgttttcaccatgggcaaatattatacgcaaggcgacaaggtgctgatgccgctggcgattcaggttcatcatgccgtttgtgatggcttccatgtcggcagaatgcttaatgaattacaacagtactgcgatgagtggcagggcggggcgtaatttttttaaggcagttattggtgcccttaaacgcctggttgctacgcctgaataagtgataataagcggatgaatggcagaaattcgatgataagctgtcaaacatgagaattggtcgacggcgcgccaaagcttgcatgcctgcagccgcgtaacctggcaaaatcggttacggttgagtaataaatggatgccctgcgtaagcggggcacatttcattacctctttctccgcacccgacatagataataacttcgtatagtatacattatacgaagttatctagtagacttaattaaggatcgatccggcgcgccaatagtcatgccccgcgcccaccggaaggagctgactgggttgaaggctctcaagggcatcggtcgagcttgacattgtaggactatattgctctaataaatttgcggccgctaatacgactcactatagggagag |
| pRC1837  (Bp5- Hsmar1 Flag) | gatccgcggaattgGTTAATAACAGGGGACGTGGTAATCCGTCCCCTTTTTATTTCTGACTGAGTTAATAACAGGCCTGCTTCGGCAGGCCTTTTTATTTCTGACTGAGTTCTTCTCAGGCCTGCTGGTAATCGCAGGCCTTTTTATTTTCTAGcCAATTCCGACGCCTAAGAAACCATTATTATCATGACATTAGCCTATAAAAATAGGCGTACCACGAGGCCCTTTCGTCTTCACCTCGAGTCCCTATCAGTGATAGAGATTGACACCCCTATCAGTGATAGAGATACTGAGCACATCAGCAGGACGCACTGACCACTTTAAGtctcctATATACATATGGAAATGATGCTCGATAAGAAACAGATTCGTGCGATCTTTCTCTTTGAGTTTAAAATGGGTCGCAAAGCGGCGGAGACGACGCGTAATATTAACAACGCGTTCGGTCCTGGCACCGCGAACGAGCGTACCGTGCAATGGTGGTTCAAAAAGTTTCGCAAAGGCGACGAATCTCTGGAGGACGAAGAGCGTTCTGGCCGCCCGTCCGAGGTTGACAACGACCAGCTGCGTGCAATCATCGAAGCTGATCCGCTGACTACCACCCGCGAAGTTGCTGAAGAACTGAATGTGGATCACTCTACTGTGGTTCGCCACCTGAAACAGATCGGTAAAGTAAAAAAACTGGACAAATGGGTTCCTCATGAACTGTCTGAAAACCAGAAAAACCGTCGTTTCGAAGTTAGCTCCTCTCTGATTCTGCGTAACAACAACGAACCGTTCCTGGATCGTATCGTAACCTGTGATGAGAAATGGATTCTGTATGATAACCGTCGCCGCTCTGCTCAGTGGCTGGATCGCGAAGAAGCTCCAAAACACTTCCCGAAACCGAATCTGCACCAGAAGAAAGTCATGGTAACCGTATGGTGGTCTGCCGCAGGTGTTATCCACTATTCCTTCCTGAACCCGGGCGAAACTATCACCAGCGAAAAATACTGCCAGCAGATTGACGAAATGCACCGTAAACTGCAGCGTCTGCAGCCAGCACTGGTGAATCGTAAAGGTCCGATCCTGCTGCATGATAACGCCCGTCCGCACGTTGCCCAACCGACCCTGCAGAAACTGAACGAACTGGGCTATGAAGTTCTGCCACACCCGCCGTACTCCCCGGATCTGTCCCCGACTGACTACCATTTCTTCAAGCATCTGGACAACTTCCTGCAGGGTAAACGTTTTCACAACCAACAGGACGCAGAAAACGCTTTCCAGGAGTTCGTCGAAAGCCGTTCCACTGACTTCTACGCGACCGGTATCAACAAGCTGATCAGCCGTTGGCAGAAATGCGTGGACTGTAACGGCAGCTACTTCGATGGATCCgactacaaggacgacgatgacaagGAAgattataaagatgatgacgataaaAGAgactacaaggacgacgatgacaagTAAAAGCTTGTTCTTCTCAGGCCTGCTGGTAATCGCAGGCCTTTTTATTTGAATTCcgcggatccttctatagtgtcacctaaatgtcgacggccaggcggccgccaggcctacccactagtcaattcgggaggatcgaaacggcagatcgcaaaaaacagtacatacagaaggagacatgaacatgaacatcaaaaaaattgtaaaacaagccacagttctgacttttacgactgcacttctggcaggaggagcgactcaagccttcgcgaaagaaaataaccaaaaagcatacaaagaaacgtacggcgtctctcatattacacgccatgatatgctgcagatccctaaacagcagcaaaacgaaaaataccaagtgcctcaattcgatcaatcaacgattaaaaatattgagtctgcaaaaggacttgatgtgtgggacagctggccgctgcaaaacgctgacggaacagtagctgaatacaacggctatcacgttgtgtttgctcttgcgggaagcccgaaagacgctgatgacacatcaatctacatgttttatcaaaaggtcggcgacaactcaatcgacagctggaaaaacgcgggccgtgtctttaaagacagcgataagttcgacgccaacgatccgatcctgaaagatcagacgcaagaatggtccggttctgcaacctttacatctgacggaaaaatccgtttattctacactgactattccggtaaacattacggcaaacaaagcctgacaacagcgcaggtaaatgtgtcaaaatctgatgacacactcaaaatcaacggagtggaagatcacaaaacgatttttgacggagacggaaaaacatatcagaacgttcagcagtttatcgatgaaggcaattatacatccggcgacaaccatacgctgagagaccctcactacgttgaagacaaaggccataaataccttgtattcgaagccaacacgggaacagaaaacggataccaaggcgaagaatctttatttaacaaagcgtactacggcggcggcacgaacttcttccgtaaagaaagccagaagcttcagcagagcgctaaaaaacgcgatgctgagttagcgaacggcgccctcggtatcatagagttaaataatgattacacattgaaaaaagtaatgaagccgctgatcacttcaaacacggtaactgatgaaatcgagcgcgcgaatgttttcaaaatgaacggcaaatggtacttgttcactgattcacgcggttcaaaaatgacgatcgatggtattaactcaaacgatatttacatgcttggttatgtatcaaactctttaaccggcccttacaagccgctgaacaaaacagggcttgtgctgcaaatgggtcttgatccaaacgatgtgacattcacttactctcacttcgcagtgccgcaagccaaaggcaacaatgtggttatcacaagctacatgacaaacagaggcttcttcgaggataaaaaggcaacatttgcgccaagcttcttaatgaacatcaaaggcaataaaacatccgttgtcaaaaacagcatcctggagcaaggacagctgacagtcaactaataacagcaaaaagaaaatgccgatacttcattggcattttcttttatttctcaacaagatggtgaattgactagtgggtagatccacaggacgggtgtggtcgccatgatcgcgtagtcgatagtggctccaagtagcgaagcgagcaggactgggcggcggccaaagcggtcggacagtgctccgagaacgggtgcgcatagaaattgcatcaacgcatatagcgctagcagcacgccatagtgactggcgatgctgtcggaatggacgatatcccgcaagaggcccggcagtaccggcataaccaagcctatgcctacagcatccagggtgacggtgccgaggatgacgatgagcgcattgttagatttcatacacggtgcctgactgcgttagcaatttaactgtgataaactaccgcattaaagcttatcgatgataagctgtcaaacatgagaattgatccggaacccttaatataacttcgtataatgtatgctatacgaagttattaggtccctcgactatagggtcaccgtcgacagcgacacacttgcatcggatgcagcccggttaacgtgccggcacggcctgggtaaccaggtattttgtccacataaccgtgcgcaaaatgttgtggataagcaggacacagcagcaatccacagcaggcatacaaccgcacaccgaggttactccgttctacaggttacgacgacatgtcaatacttgcccttgacaggcattgatggaatcgtagtctcacgctgatagtctgatcgacaatacaagtgggaccgtggtcccagaccgataatcagaccgacaacacgagtgggatcgtggtcccagactaataatcagaccgacgatacgagtgggaccgtggtcccagactaataatcagaccgacgatacgagtgggaccgtggttccagactaataatcagaccgacgatacgagtgggaccgtggtcccagactaataatcagaccgacgatacgagtgggaccatggtcccagactaataatcagaccgacgatacgagtgggaccgtggtcccagtctgattatcagaccgacgatacgagtgggaccgtggtcccagactaataatcagaccgacgatacgagtgggaccgtggtcccagactaataatcagaccgacgatacgagtgggaccgtggtcccagtctgattatcagaccgacgatacaagtggaacagtgggcccagagagaatattcaggccagttatgctttctggcctgtaacaaaggacattaagtaaagacagataaacgtagactaaaacgtggtcgcatcagggtgctggcttttcaagttccttaagaatggcctcaattttctctatacactcagttggaacacgagacctgtccaggttaagcaccattttatcgcccttatacaatactgtcgctccaggagcaaactgatgtcgtgagcttaaactagttcttgatgcagatgacgttttaagcacagaagttaaaagagtgataacttcttcagcttcaaatatcaccccagcttttttctgctcatgaaggttagatgcctgctgcttaagtaattcctctttatctgtaaaggctttttgaagtgcatcacctgaccgggcagatagttcaccggggtgagaaaaaagagcaacaactgatttaggcaatttggcggtgttgatacagcgggtaataatcttacgtgaaatattttccgcatcagccagcgcagaaatatttccagcaaattcattctgcaatcggcttgcataacgctgaccacgttcataagcacttgttgggcgataatcgttacccaatctggataatgcagccatctgctcatcatccagctcgccaaccagaacacgataatcactttcggtaagtgcagcagctttacgacggcgactcccatcggcaatttctatgacaccagatactcttcgaccgaacgccggtgtctgttgaccagtcagtagaaaagaagggatgagatcatccagtgcgtcctcagtaagcagctcctggtcacgttcattacctgaccatacccgagaggtcttctcaacactatcaccccggagcacttcaagagtaaacttcacatcccgaccacatacaggcaaagtaatggcattaccgcgagccattactcctacgcgcgcaattaacgaatccaccatcggggcagctggtgtcgataacgaagtatcttcaaccggttgagtattgagcgtatgttttggaataacaggcgcacgcttcattatctaatctcccagcgtggtttaatcagacgatcgaaaatttcattgcagacaggttcccaaatagaaagagcatttctccaggcaccagttgaagagcgttgatcaatggcctgttcaaaaacagttctcatccggatctgacctttaccaacttcatccgtttcacgtacaacattttttagaaccatgcttccccaggcatcccgaatttgctcctccatccacggggactgagagccattactattgctgtatttggtaagcaaaatacgtacatcaggctcgaaccctttaagatcaacgttcttgagcagatcacgaagcatatcgaaaaactgcagtgcggaggtgtagtcaaacaactcagcaggcgtgggaacaatcagcacatcagcagcacatacgacattaatcgtgccgatacccaggttaggcgcgctgtcaataactatgacatcatagtcatgagcaacagtttcaatggccagtcggagcatcaggtgtggatcggtgggcagtttaccttcatcaaatttgcccattaactcagtttcaatacggtgcagagccagacaggaaggaataatgtcaagccccggccagcaagtgggctttattgcataagtgacatcgtccttttccccaagatagaaaggcaggagagtgtcttctgcatgaatatgaagatctggtacccatccgtgatacattgaggctgttccctgggggtcgttaccttccacgagcaaaacacgtagccccttcagagccagatcctgagcaagatgaacagaaactgaggttttgtaaacgccacctttatgggcagcaaccccgatcaccggtggaaatacgtcttcagcacgtcgcaatcgcgtaccaaacacatcacgcatatgattaatttgttcaattgtataaccaacacgttgctcaacccgtcctcgaatttccatatccgggtgcggtagtcgccctgctttctcggcatctctgatagcctgagaagaaaccccaactaaatccgctgcttcacctattctccagcgccgggttattttcctcgcttccgggctgtcatcattaaactgtgcaatggcgatagccttcgtcatttcatgaccagcgtttatgcactggttaagtgtttccatgagtttcattctgaacatcctttaatcattgctttgcgtttttttattaaatcttgcaatttactgcaaagcaacaacaaaatcgcaaagtcatcaaaaaaccgcaaagttgtttaaaataagagcaacactacaaaaggagataagaagagcacatacctcagtcacttattatcactagcgctcgccgcagccgtgtaaccgagcatagcgagcgaactggcgaggaagcaaagaagaactgttctgtcagatagctcttacgctcagcgcaagaagaaatatccaccgtgggaaaaactccaggtagaggtacacacgcggatagccaattcagagtaataaactgtgataatcaaccctcatcaatgatgacgaactaacccccgatatcaggtcacatgacgaagggaaagagaaggaaatcaactgtgacaaactgccctcaaatttggcttccttaaaaattacagttcaaaaagtatgagaaaatccatgcaggctgaaggaaacagcaaaactgtgacaaattaccctcagtaggtcagaacaaatgtgacgaaccaccctcaaatctgtgacagataaccctcagactatcctgtcgtcatggaagtgatatcgcggaaggaaaatacgatatgagtcgtctggcggcctttctttttctcaatgtatgagaggcgcattggagttctgctgttgatctcattaacacagacctgcaggaagcggcggcggaagtcaggcatacgctggtaactttgaggcagctggtaacgctctatgatccagtcgattttcagagagacgatgcctgagccatccggcttacgatactgacacagggattcgtataaacgcatggcatacggattggtgatttcttttgtttcactaagccgaaactgcgtaaaccggttctgtaacccgataaagaagggaatgagatatgggttgatatgtacactgtaaagccctctggatggactgtgcgcacgtttgataaaccaaggaaaagattcatagcctttttcatcgccggcatcctcttcagggcgataaaaaaccacttccttccccgcgaaactcttcaatgcctgccgtatatccttactggcttccgcagaggtcaatccgaatatttcagcatatttagcaacatggatctcgcagataccgtcatgttcctgtagggtgccatcagattttctgatctggtcaacgaacagatacagcatacgtttttgatcccgggagagactatatgccgcctcagtgaggtcgtttgactggacgattcgcgggctatttttacgtttcttgtgattgataaccgctgtttccgccatgacagatccatgtgaagtgtgacaagtttttagattgtcacactaaataaaaaagagtcaataagcagggataactttgtgaaaaaacagcttcttctgagggcaatttgtcacagggttaagggcaatttgtcacagACAGGACTGTCATTTGAGGGTGATTTGTCACACTGAAAGGGCAATTTGTCACAACACCTTCTCTAGAACCAGCATGGATAAAGGCCTACAAGGCGCTCTAAAAAAGAAGATCTAAAAACTATAAAAAAAATAATTATAAAAATATCCCCGTGGATAAGTGGATAACCCCAAGGGAAGTTTTTTCAGGCATCGTGTGTAAGCAGAATATATAAGTGCTGTTCCCTGGTGCTTCCTCGCTCACTCGAGGGCTTCGCCCTGTCGCTCAACTGCGGCGAGCACTACTGGCTGTAAaaggacagaccacatcatggttctgtgttcattaggttgttctgtccattgctgacataatccgctccacttcaacgtaacaccgcacgaagatttctattgttcctgaaggcatattcaaatcgttttcgttaccgcttgcaggcatcatgacagaacactacttcctataaacgctacacaggctcctgagattaataatgcggatctctacgataatgggagattttcccgactgtttcgttcgcttctcagtggataacagccagcttctctgtttaacagacaaaaacagcatatccactcagttccacatttccatataaaggccaaggcatttattctcaggataattgtttcagcatcgcaaccgcatcagactccggcatcgcaaactgcacccggtgccgggcagccacatccagcgcaaaaaccttcgtgtagacttccgttgaactgatggacttatgtcccatcaggctttgcagaactttcagcggtataccggcatacagcatgtgcatcgcataggaatggcggaacgtatgtggtgtgaccggaacagagaacgtcacaccgtcagcagcagcggcggcaaccgcctccccaatccaggtcctgaccgttctgtccgtcacttcccagatccgcgctttctctgtccttcctgtgcgacggttacgccgctccatgagcttatcgcgaataaatacctgtgacggaagatcacttcgcagaataaataaatcctggtgtccctgttgataccgggaagccctgggccaacttttggcgaaaatgagacgttgatcggcacgtaagaggttccaactttcaccataatgaaataagatcactaccgggcgtattttttgagttatcgagattttcaggagctaaggaagctaaaatggagaaaaaaatcactggatataccaccgttgatatatcccaatggcatcgtaaagaacattttgaggcatttcagtcagttgctcaatgtacctataaccagaccgttcagctggatattacggcctttttaaagaccgtaaagaaaaataagcacaagttttatccggcctttattcacattcttgcccgcctgatgaatgctcatccggagttccgtatggcaatgaaagacggtgagctggtgatatgggatagtgttcacccttgttacaccgttttccatgagcaaactgaaacgttttcatcgctctggagtgaataccacgacgatttccggcagtttctacacatatattcgcaagatgtggcgtgttacggtgaaaacctggcctatttccctaaagggtttattgagaatatgtttttcgtctcagccaatccctgggtgagtttcaccagttttgatttaaacgtggccaatatggacaacttcttcgcccccgttttcaccatgggcaaatattatacgcaaggcgacaaggtgctgatgccgctggcgattcaggttcatcatgccgtttgtgatggcttccatgtcggcagaatgcttaatgaattacaacagtactgcgatgagtggcagggcggggcgtaatttttttaaggcagttattggtgcccttaaacgcctggttgctacgcctgaataagtgataataagcggatgaatggcagaaattcgatgataagctgtcaaacatgagaattggtcgacggcgcgccaaagcttgcatgcctgcagccgcgtaacctggcaaaatcggttacggttgagtaataaatggatgccctgcgtaagcggggcacatttcattacctctttctccgcacccgacatagataataacttcgtatagtatacattatacgaagttatctagtagacttaattaaggatcgatccggcgcgccaatagtcatgccccgcgcccaccggaaggagctgactgggttgaaggctctcaagggcatcggtcgagcttgacattgtaggactatattgctctaataaatttgcggccgctaatacgactcactatagggagag |
| pRC1838  (Bp6- Hsmar1 Flag) | gatccgcggaattgGTTAATAACAGGGGACGTGGTAATCCGTCCCCTTTTTATTTCTGACTGAGTTAATAACAGGCCTGCTTCGGCAGGCCTTTTTATTTCTGACTGAGTTCTTCTCAGGCCTGCTGGTAATCGCAGGCCTTTTTATTTTCTAGTCAATTCCGACGTCTAAGAAACCATTATTATCATGACATTAACCTATAAAAATAGGCGTATCACGAGGCCCTTTCGTCTTCACCTCGAGTCCCTATCAGTGATAGAGATTGACATCCCTATCAGTGATAGAGATACTGAGCACATCAGCAGGACGCACTGACCACTTTAAGtctcctATATACATATGGAAATGATGCTCGATAAGAAACAGATTCGTGCGATCTTTCTCTTTGAGTTTAAAATGGGTCGCAAAGCGGCGGAGACGACGCGTAATATTAACAACGCGTTCGGTCCTGGCACCGCGAACGAGCGTACCGTGCAATGGTGGTTCAAAAAGTTTCGCAAAGGCGACGAATCTCTGGAGGACGAAGAGCGTTCTGGCCGCCCGTCCGAGGTTGACAACGACCAGCTGCGTGCAATCATCGAAGCTGATCCGCTGACTACCACCCGCGAAGTTGCTGAAGAACTGAATGTGGATCACTCTACTGTGGTTCGCCACCTGAAACAGATCGGTAAAGTAAAAAAACTGGACAAATGGGTTCCTCATGAACTGTCTGAAAACCAGAAAAACCGTCGTTTCGAAGTTAGCTCCTCTCTGATTCTGCGTAACAACAACGAACCGTTCCTGGATCGTATCGTAACCTGTGATGAGAAATGGATTCTGTATGATAACCGTCGCCGCTCTGCTCAGTGGCTGGATCGCGAAGAAGCTCCAAAACACTTCCCGAAACCGAATCTGCACCAGAAGAAAGTCATGGTAACCGTATGGTGGTCTGCCGCAGGTGTTATCCACTATTCCTTCCTGAACCCGGGCGAAACTATCACCAGCGAAAAATACTGCCAGCAGATTGACGAAATGCACCGTAAACTGCAGCGTCTGCAGCCAGCACTGGTGAATCGTAAAGGTCCGATCCTGCTGCATGATAACGCCCGTCCGCACGTTGCCCAACCGACCCTGCAGAAACTGAACGAACTGGGCTATGAAGTTCTGCCACACCCGCCGTACTCCCCGGATCTGTCCCCGACTGACTACCATTTCTTCAAGCATCTGGACAACTTCCTGCAGGGTAAACGTTTTCACAACCAACAGGACGCAGAAAACGCTTTCCAGGAGTTCGTCGAAAGCCGTTCCACTGACTTCTACGCGACCGGTATCAACAAGCTGATCAGCCGTTGGCAGAAATGCGTGGACTGTAACGGCAGCTACTTCGATGGATCCgactacaaggacgacgatgacaagGAAgattataaagatgatgacgataaaAGAgactacaaggacgacgatgacaagTAAAAGCTTGTTCTTCTCAGGCCTGCTGGTAATCGCAGGCCTTTTTATTTGAATTCcgcggatccttctatagtgtcacctaaatgtcgacggccaggcggccgccaggcctacccactagtcaattcgggaggatcgaaacggcagatcgcaaaaaacagtacatacagaaggagacatgaacatgaacatcaaaaaaattgtaaaacaagccacagttctgacttttacgactgcacttctggcaggaggagcgactcaagccttcgcgaaagaaaataaccaaaaagcatacaaagaaacgtacggcgtctctcatattacacgccatgatatgctgcagatccctaaacagcagcaaaacgaaaaataccaagtgcctcaattcgatcaatcaacgattaaaaatattgagtctgcaaaaggacttgatgtgtgggacagctggccgctgcaaaacgctgacggaacagtagctgaatacaacggctatcacgttgtgtttgctcttgcgggaagcccgaaagacgctgatgacacatcaatctacatgttttatcaaaaggtcggcgacaactcaatcgacagctggaaaaacgcgggccgtgtctttaaagacagcgataagttcgacgccaacgatccgatcctgaaagatcagacgcaagaatggtccggttctgcaacctttacatctgacggaaaaatccgtttattctacactgactattccggtaaacattacggcaaacaaagcctgacaacagcgcaggtaaatgtgtcaaaatctgatgacacactcaaaatcaacggagtggaagatcacaaaacgatttttgacggagacggaaaaacatatcagaacgttcagcagtttatcgatgaaggcaattatacatccggcgacaaccatacgctgagagaccctcactacgttgaagacaaaggccataaataccttgtattcgaagccaacacgggaacagaaaacggataccaaggcgaagaatctttatttaacaaagcgtactacggcggcggcacgaacttcttccgtaaagaaagccagaagcttcagcagagcgctaaaaaacgcgatgctgagttagcgaacggcgccctcggtatcatagagttaaataatgattacacattgaaaaaagtaatgaagccgctgatcacttcaaacacggtaactgatgaaatcgagcgcgcgaatgttttcaaaatgaacggcaaatggtacttgttcactgattcacgcggttcaaaaatgacgatcgatggtattaactcaaacgatatttacatgcttggttatgtatcaaactctttaaccggcccttacaagccgctgaacaaaacagggcttgtgctgcaaatgggtcttgatccaaacgatgtgacattcacttactctcacttcgcagtgccgcaagccaaaggcaacaatgtggttatcacaagctacatgacaaacagaggcttcttcgaggataaaaaggcaacatttgcgccaagcttcttaatgaacatcaaaggcaataaaacatccgttgtcaaaaacagcatcctggagcaaggacagctgacagtcaactaataacagcaaaaagaaaatgccgatacttcattggcattttcttttatttctcaacaagatggtgaattgactagtgggtagatccacaggacgggtgtggtcgccatgatcgcgtagtcgatagtggctccaagtagcgaagcgagcaggactgggcggcggccaaagcggtcggacagtgctccgagaacgggtgcgcatagaaattgcatcaacgcatatagcgctagcagcacgccatagtgactggcgatgctgtcggaatggacgatatcccgcaagaggcccggcagtaccggcataaccaagcctatgcctacagcatccagggtgacggtgccgaggatgacgatgagcgcattgttagatttcatacacggtgcctgactgcgttagcaatttaactgtgataaactaccgcattaaagcttatcgatgataagctgtcaaacatgagaattgatccggaacccttaatataacttcgtataatgtatgctatacgaagttattaggtccctcgactatagggtcaccgtcgacagcgacacacttgcatcggatgcagcccggttaacgtgccggcacggcctgggtaaccaggtattttgtccacataaccgtgcgcaaaatgttgtggataagcaggacacagcagcaatccacagcaggcatacaaccgcacaccgaggttactccgttctacaggttacgacgacatgtcaatacttgcccttgacaggcattgatggaatcgtagtctcacgctgatagtctgatcgacaatacaagtgggaccgtggtcccagaccgataatcagaccgacaacacgagtgggatcgtggtcccagactaataatcagaccgacgatacgagtgggaccgtggtcccagactaataatcagaccgacgatacgagtgggaccgtggttccagactaataatcagaccgacgatacgagtgggaccgtggtcccagactaataatcagaccgacgatacgagtgggaccatggtcccagactaataatcagaccgacgatacgagtgggaccgtggtcccagtctgattatcagaccgacgatacgagtgggaccgtggtcccagactaataatcagaccgacgatacgagtgggaccgtggtcccagactaataatcagaccgacgatacgagtgggaccgtggtcccagtctgattatcagaccgacgatacaagtggaacagtgggcccagagagaatattcaggccagttatgctttctggcctgtaacaaaggacattaagtaaagacagataaacgtagactaaaacgtggtcgcatcagggtgctggcttttcaagttccttaagaatggcctcaattttctctatacactcagttggaacacgagacctgtccaggttaagcaccattttatcgcccttatacaatactgtcgctccaggagcaaactgatgtcgtgagcttaaactagttcttgatgcagatgacgttttaagcacagaagttaaaagagtgataacttcttcagcttcaaatatcaccccagcttttttctgctcatgaaggttagatgcctgctgcttaagtaattcctctttatctgtaaaggctttttgaagtgcatcacctgaccgggcagatagttcaccggggtgagaaaaaagagcaacaactgatttaggcaatttggcggtgttgatacagcgggtaataatcttacgtgaaatattttccgcatcagccagcgcagaaatatttccagcaaattcattctgcaatcggcttgcataacgctgaccacgttcataagcacttgttgggcgataatcgttacccaatctggataatgcagccatctgctcatcatccagctcgccaaccagaacacgataatcactttcggtaagtgcagcagctttacgacggcgactcccatcggcaatttctatgacaccagatactcttcgaccgaacgccggtgtctgttgaccagtcagtagaaaagaagggatgagatcatccagtgcgtcctcagtaagcagctcctggtcacgttcattacctgaccatacccgagaggtcttctcaacactatcaccccggagcacttcaagagtaaacttcacatcccgaccacatacaggcaaagtaatggcattaccgcgagccattactcctacgcgcgcaattaacgaatccaccatcggggcagctggtgtcgataacgaagtatcttcaaccggttgagtattgagcgtatgttttggaataacaggcgcacgcttcattatctaatctcccagcgtggtttaatcagacgatcgaaaatttcattgcagacaggttcccaaatagaaagagcatttctccaggcaccagttgaagagcgttgatcaatggcctgttcaaaaacagttctcatccggatctgacctttaccaacttcatccgtttcacgtacaacattttttagaaccatgcttccccaggcatcccgaatttgctcctccatccacggggactgagagccattactattgctgtatttggtaagcaaaatacgtacatcaggctcgaaccctttaagatcaacgttcttgagcagatcacgaagcatatcgaaaaactgcagtgcggaggtgtagtcaaacaactcagcaggcgtgggaacaatcagcacatcagcagcacatacgacattaatcgtgccgatacccaggttaggcgcgctgtcaataactatgacatcatagtcatgagcaacagtttcaatggccagtcggagcatcaggtgtggatcggtgggcagtttaccttcatcaaatttgcccattaactcagtttcaatacggtgcagagccagacaggaaggaataatgtcaagccccggccagcaagtgggctttattgcataagtgacatcgtccttttccccaagatagaaaggcaggagagtgtcttctgcatgaatatgaagatctggtacccatccgtgatacattgaggctgttccctgggggtcgttaccttccacgagcaaaacacgtagccccttcagagccagatcctgagcaagatgaacagaaactgaggttttgtaaacgccacctttatgggcagcaaccccgatcaccggtggaaatacgtcttcagcacgtcgcaatcgcgtaccaaacacatcacgcatatgattaatttgttcaattgtataaccaacacgttgctcaacccgtcctcgaatttccatatccgggtgcggtagtcgccctgctttctcggcatctctgatagcctgagaagaaaccccaactaaatccgctgcttcacctattctccagcgccgggttattttcctcgcttccgggctgtcatcattaaactgtgcaatggcgatagccttcgtcatttcatgaccagcgtttatgcactggttaagtgtttccatgagtttcattctgaacatcctttaatcattgctttgcgtttttttattaaatcttgcaatttactgcaaagcaacaacaaaatcgcaaagtcatcaaaaaaccgcaaagttgtttaaaataagagcaacactacaaaaggagataagaagagcacatacctcagtcacttattatcactagcgctcgccgcagccgtgtaaccgagcatagcgagcgaactggcgaggaagcaaagaagaactgttctgtcagatagctcttacgctcagcgcaagaagaaatatccaccgtgggaaaaactccaggtagaggtacacacgcggatagccaattcagagtaataaactgtgataatcaaccctcatcaatgatgacgaactaacccccgatatcaggtcacatgacgaagggaaagagaaggaaatcaactgtgacaaactgccctcaaatttggcttccttaaaaattacagttcaaaaagtatgagaaaatccatgcaggctgaaggaaacagcaaaactgtgacaaattaccctcagtaggtcagaacaaatgtgacgaaccaccctcaaatctgtgacagataaccctcagactatcctgtcgtcatggaagtgatatcgcggaaggaaaatacgatatgagtcgtctggcggcctttctttttctcaatgtatgagaggcgcattggagttctgctgttgatctcattaacacagacctgcaggaagcggcggcggaagtcaggcatacgctggtaactttgaggcagctggtaacgctctatgatccagtcgattttcagagagacgatgcctgagccatccggcttacgatactgacacagggattcgtataaacgcatggcatacggattggtgatttcttttgtttcactaagccgaaactgcgtaaaccggttctgtaacccgataaagaagggaatgagatatgggttgatatgtacactgtaaagccctctggatggactgtgcgcacgtttgataaaccaaggaaaagattcatagcctttttcatcgccggcatcctcttcagggcgataaaaaaccacttccttccccgcgaaactcttcaatgcctgccgtatatccttactggcttccgcagaggtcaatccgaatatttcagcatatttagcaacatggatctcgcagataccgtcatgttcctgtagggtgccatcagattttctgatctggtcaacgaacagatacagcatacgtttttgatcccgggagagactatatgccgcctcagtgaggtcgtttgactggacgattcgcgggctatttttacgtttcttgtgattgataaccgctgtttccgccatgacagatccatgtgaagtgtgacaagtttttagattgtcacactaaataaaaaagagtcaataagcagggataactttgtgaaaaaacagcttcttctgagggcaatttgtcacagggttaagggcaatttgtcacagACAGGACTGTCATTTGAGGGTGATTTGTCACACTGAAAGGGCAATTTGTCACAACACCTTCTCTAGAACCAGCATGGATAAAGGCCTACAAGGCGCTCTAAAAAAGAAGATCTAAAAACTATAAAAAAAATAATTATAAAAATATCCCCGTGGATAAGTGGATAACCCCAAGGGAAGTTTTTTCAGGCATCGTGTGTAAGCAGAATATATAAGTGCTGTTCCCTGGTGCTTCCTCGCTCACTCGAGGGCTTCGCCCTGTCGCTCAACTGCGGCGAGCACTACTGGCTGTAAaaggacagaccacatcatggttctgtgttcattaggttgttctgtccattgctgacataatccgctccacttcaacgtaacaccgcacgaagatttctattgttcctgaaggcatattcaaatcgttttcgttaccgcttgcaggcatcatgacagaacactacttcctataaacgctacacaggctcctgagattaataatgcggatctctacgataatgggagattttcccgactgtttcgttcgcttctcagtggataacagccagcttctctgtttaacagacaaaaacagcatatccactcagttccacatttccatataaaggccaaggcatttattctcaggataattgtttcagcatcgcaaccgcatcagactccggcatcgcaaactgcacccggtgccgggcagccacatccagcgcaaaaaccttcgtgtagacttccgttgaactgatggacttatgtcccatcaggctttgcagaactttcagcggtataccggcatacagcatgtgcatcgcataggaatggcggaacgtatgtggtgtgaccggaacagagaacgtcacaccgtcagcagcagcggcggcaaccgcctccccaatccaggtcctgaccgttctgtccgtcacttcccagatccgcgctttctctgtccttcctgtgcgacggttacgccgctccatgagcttatcgcgaataaatacctgtgacggaagatcacttcgcagaataaataaatcctggtgtccctgttgataccgggaagccctgggccaacttttggcgaaaatgagacgttgatcggcacgtaagaggttccaactttcaccataatgaaataagatcactaccgggcgtattttttgagttatcgagattttcaggagctaaggaagctaaaatggagaaaaaaatcactggatataccaccgttgatatatcccaatggcatcgtaaagaacattttgaggcatttcagtcagttgctcaatgtacctataaccagaccgttcagctggatattacggcctttttaaagaccgtaaagaaaaataagcacaagttttatccggcctttattcacattcttgcccgcctgatgaatgctcatccggagttccgtatggcaatgaaagacggtgagctggtgatatgggatagtgttcacccttgttacaccgttttccatgagcaaactgaaacgttttcatcgctctggagtgaataccacgacgatttccggcagtttctacacatatattcgcaagatgtggcgtgttacggtgaaaacctggcctatttccctaaagggtttattgagaatatgtttttcgtctcagccaatccctgggtgagtttcaccagttttgatttaaacgtggccaatatggacaacttcttcgcccccgttttcaccatgggcaaatattatacgcaaggcgacaaggtgctgatgccgctggcgattcaggttcatcatgccgtttgtgatggcttccatgtcggcagaatgcttaatgaattacaacagtactgcgatgagtggcagggcggggcgtaatttttttaaggcagttattggtgcccttaaacgcctggttgctacgcctgaataagtgataataagcggatgaatggcagaaattcgatgataagctgtcaaacatgagaattggtcgacggcgcgccaaagcttgcatgcctgcagccgcgtaacctggcaaaatcggttacggttgagtaataaatggatgccctgcgtaagcggggcacatttcattacctctttctccgcacccgacatagataataacttcgtatagtatacattatacgaagttatctagtagacttaattaaggatcgatccggcgcgccaatagtcatgccccgcgcccaccggaaggagctgactgggttgaaggctctcaagggcatcggtcgagcttgacattgtaggactatattgctctaataaatttgcggccgctaatacgactcactatagggagag |
| pRC1845  (Bp-EE+ Hsmar1 Flag) | gatccgcggaattgTTAATAACAGGGGACGTGGTAATCCGTCCCCTTTTTATTTCTGACTGAGTTAATAACAGGCCTGCTTCGGCAGGCCTTTTTATTTCTGACTGAGTTCTTCTCAGGCCTGCTGGTAATCGCAGGCCTTTTTATTTTCTAGgCTGACTGACTGACTGACTGACTGACTGACTGACTGACTGACTGACTGACTGACTGACTGACTGACTGACTGACTGACTGACTGACTGACTGACTGACTGACTGACTGACTGACTGACTGACTGACTGACTGACTGACTGACTGACTGACTGACTGACTGACTGACTGACCATATGGAAATGATGCTCGATAAGAAACAGATTCGTGCGATCTTTCTCTTTGAGTTTAAAATGGGTCGCAAAGCGGCGGAGACGACGCGTAATATTAACAACGCGTTCGGTCCTGGCACCGCGAACGAGCGTACCGTGCAATGGTGGTTCAAAAAGTTTCGCAAAGGCGACGAATCTCTGGAGGACGAAGAGCGTTCTGGCCGCCCGTCCGAGGTTGACAACGACCAGCTGCGTGCAATCATCGAAGCTGATCCGCTGACTACCACCCGCGAAGTTGCTGAAGAACTGAATGTGGATCACTCTACTGTGGTTCGCCACCTGAAACAGATCGGTAAAGTAAAAAAACTGGACAAATGGGTTCCTCATGAACTGTCTGAAAACCAGAAAAACCGTCGTTTCGAAGTTAGCTCCTCTCTGATTCTGCGTAACAACAACGAACCGTTCCTGGATCGTATCGTAACCTGTGATGAGAAATGGATTCTGTATGATAACCGTCGCCGCTCTGCTCAGTGGCTGGATCGCGAAGAAGCTCCAAAACACTTCCCGAAACCGAATCTGCACCAGAAGAAAGTCATGGTAACCGTATGGTGGTCTGCCGCAGGTGTTATCCACTATTCCTTCCTGAACCCGGGCGAAACTATCACCAGCGAAAAATACTGCCAGCAGATTGACGAAATGCACCGTAAACTGCAGCGTCTGCAGCCAGCACTGGTGAATCGTAAAGGTCCGATCCTGCTGCATGATAACGCCCGTCCGCACGTTGCCCAACCGACCCTGCAGAAACTGAACGAACTGGGCTATGAAGTTCTGCCACACCCGCCGTACTCCCCGGATCTGTCCCCGACTGACTACCATTTCTTCAAGCATCTGGACAACTTCCTGCAGGGTAAACGTTTTCACAACCAACAGGACGCAGAAAACGCTTTCCAGGAGTTCGTCGAAAGCCGTTCCACTGACTTCTACGCGACCGGTATCAACAAGCTGATCAGCCGTTGGCAGAAATGCGTGGACTGTAACGGCAGCTACTTCGATGGATCCgactacaaggacgacgatgacaagGAAgattataaagatgatgacgataaaAGAgactacaaggacgacgatgacaagTAAAAGCTTGTTCTTCTCAGGCCTGCTGGTAATCGCAGGCCTTTTTATTTGAATTCcgcggatccttctatagtgtcacctaaatgtcgacggccaggcggccgccaggcctacccactagtcaattcgggaggatcgaaacggcagatcgcaaaaaacagtacatacagaaggagacatgaacatgaacatcaaaaaaattgtaaaacaagccacagttctgacttttacgactgcacttctggcaggaggagcgactcaagccttcgcgaaagaaaataaccaaaaagcatacaaagaaacgtacggcgtctctcatattacacgccatgatatgctgcagatccctaaacagcagcaaaacgaaaaataccaagtgcctcaattcgatcaatcaacgattaaaaatattgagtctgcaaaaggacttgatgtgtgggacagctggccgctgcaaaacgctgacggaacagtagctgaatacaacggctatcacgttgtgtttgctcttgcgggaagcccgaaagacgctgatgacacatcaatctacatgttttatcaaaaggtcggcgacaactcaatcgacagctggaaaaacgcgggccgtgtctttaaagacagcgataagttcgacgccaacgatccgatcctgaaagatcagacgcaagaatggtccggttctgcaacctttacatctgacggaaaaatccgtttattctacactgactattccggtaaacattacggcaaacaaagcctgacaacagcgcaggtaaatgtgtcaaaatctgatgacacactcaaaatcaacggagtggaagatcacaaaacgatttttgacggagacggaaaaacatatcagaacgttcagcagtttatcgatgaaggcaattatacatccggcgacaaccatacgctgagagaccctcactacgttgaagacaaaggccataaataccttgtattcgaagccaacacgggaacagaaaacggataccaaggcgaagaatctttatttaacaaagcgtactacggcggcggcacgaacttcttccgtaaagaaagccagaagcttcagcagagcgctaaaaaacgcgatgctgagttagcgaacggcgccctcggtatcatagagttaaataatgattacacattgaaaaaagtaatgaagccgctgatcacttcaaacacggtaactgatgaaatcgagcgcgcgaatgttttcaaaatgaacggcaaatggtacttgttcactgattcacgcggttcaaaaatgacgatcgatggtattaactcaaacgatatttacatgcttggttatgtatcaaactctttaaccggcccttacaagccgctgaacaaaacagggcttgtgctgcaaatgggtcttgatccaaacgatgtgacattcacttactctcacttcgcagtgccgcaagccaaaggcaacaatgtggttatcacaagctacatgacaaacagaggcttcttcgaggataaaaaggcaacatttgcgccaagcttcttaatgaacatcaaaggcaataaaacatccgttgtcaaaaacagcatcctggagcaaggacagctgacagtcaactaataacagcaaaaagaaaatgccgatacttcattggcattttcttttatttctcaacaagatggtgaattgactagtgggtagatccacaggacgggtgtggtcgccatgatcgcgtagtcgatagtggctccaagtagcgaagcgagcaggactgggcggcggccaaagcggtcggacagtgctccgagaacgggtgcgcatagaaattgcatcaacgcatatagcgctagcagcacgccatagtgactggcgatgctgtcggaatggacgatatcccgcaagaggcccggcagtaccggcataaccaagcctatgcctacagcatccagggtgacggtgccgaggatgacgatgagcgcattgttagatttcatacacggtgcctgactgcgttagcaatttaactgtgataaactaccgcattaaagcttatcgatgataagctgtcaaacatgagaattgatccggaacccttaatataacttcgtataatgtatgctatacgaagttattaggtccctcgactatagggtcaccgtcgacagcgacacacttgcatcggatgcagcccggttaacgtgccggcacggcctgggtaaccaggtattttgtccacataaccgtgcgcaaaatgttgtggataagcaggacacagcagcaatccacagcaggcatacaaccgcacaccgaggttactccgttctacaggttacgacgacatgtcaatacttgcccttgacaggcattgatggaatcgtagtctcacgctgatagtctgatcgacaatacaagtgggaccgtggtcccagaccgataatcagaccgacaacacgagtgggatcgtggtcccagactaataatcagaccgacgatacgagtgggaccgtggtcccagactaataatcagaccgacgatacgagtgggaccgtggttccagactaataatcagaccgacgatacgagtgggaccgtggtcccagactaataatcagaccgacgatacgagtgggaccatggtcccagactaataatcagaccgacgatacgagtgggaccgtggtcccagtctgattatcagaccgacgatacgagtgggaccgtggtcccagactaataatcagaccgacgatacgagtgggaccgtggtcccagactaataatcagaccgacgatacgagtgggaccgtggtcccagtctgattatcagaccgacgatacaagtggaacagtgggcccagagagaatattcaggccagttatgctttctggcctgtaacaaaggacattaagtaaagacagataaacgtagactaaaacgtggtcgcatcagggtgctggcttttcaagttccttaagaatggcctcaattttctctatacactcagttggaacacgagacctgtccaggttaagcaccattttatcgcccttatacaatactgtcgctccaggagcaaactgatgtcgtgagcttaaactagttcttgatgcagatgacgttttaagcacagaagttaaaagagtgataacttcttcagcttcaaatatcaccccagcttttttctgctcatgaaggttagatgcctgctgcttaagtaattcctctttatctgtaaaggctttttgaagtgcatcacctgaccgggcagatagttcaccggggtgagaaaaaagagcaacaactgatttaggcaatttggcggtgttgatacagcgggtaataatcttacgtgaaatattttccgcatcagccagcgcagaaatatttccagcaaattcattctgcaatcggcttgcataacgctgaccacgttcataagcacttgttgggcgataatcgttacccaatctggataatgcagccatctgctcatcatccagctcgccaaccagaacacgataatcactttcggtaagtgcagcagctttacgacggcgactcccatcggcaatttctatgacaccagatactcttcgaccgaacgccggtgtctgttgaccagtcagtagaaaagaagggatgagatcatccagtgcgtcctcagtaagcagctcctggtcacgttcattacctgaccatacccgagaggtcttctcaacactatcaccccggagcacttcaagagtaaacttcacatcccgaccacatacaggcaaagtaatggcattaccgcgagccattactcctacgcgcgcaattaacgaatccaccatcggggcagctggtgtcgataacgaagtatcttcaaccggttgagtattgagcgtatgttttggaataacaggcgcacgcttcattatctaatctcccagcgtggtttaatcagacgatcgaaaatttcattgcagacaggttcccaaatagaaagagcatttctccaggcaccagttgaagagcgttgatcaatggcctgttcaaaaacagttctcatccggatctgacctttaccaacttcatccgtttcacgtacaacattttttagaaccatgcttccccaggcatcccgaatttgctcctccatccacggggactgagagccattactattgctgtatttggtaagcaaaatacgtacatcaggctcgaaccctttaagatcaacgttcttgagcagatcacgaagcatatcgaaaaactgcagtgcggaggtgtagtcaaacaactcagcaggcgtgggaacaatcagcacatcagcagcacatacgacattaatcgtgccgatacccaggttaggcgcgctgtcaataactatgacatcatagtcatgagcaacagtttcaatggccagtcggagcatcaggtgtggatcggtgggcagtttaccttcatcaaatttgcccattaactcagtttcaatacggtgcagagccagacaggaaggaataatgtcaagccccggccagcaagtgggctttattgcataagtgacatcgtccttttccccaagatagaaaggcaggagagtgtcttctgcatgaatatgaagatctggtacccatccgtgatacattgaggctgttccctgggggtcgttaccttccacgagcaaaacacgtagccccttcagagccagatcctgagcaagatgaacagaaactgaggttttgtaaacgccacctttatgggcagcaaccccgatcaccggtggaaatacgtcttcagcacgtcgcaatcgcgtaccaaacacatcacgcatatgattaatttgttcaattgtataaccaacacgttgctcaacccgtcctcgaatttccatatccgggtgcggtagtcgccctgctttctcggcatctctgatagcctgagaagaaaccccaactaaatccgctgcttcacctattctccagcgccgggttattttcctcgcttccgggctgtcatcattaaactgtgcaatggcgatagccttcgtcatttcatgaccagcgtttatgcactggttaagtgtttccatgagtttcattctgaacatcctttaatcattgctttgcgtttttttattaaatcttgcaatttactgcaaagcaacaacaaaatcgcaaagtcatcaaaaaaccgcaaagttgtttaaaataagagcaacactacaaaaggagataagaagagcacatacctcagtcacttattatcactagcgctcgccgcagccgtgtaaccgagcatagcgagcgaactggcgaggaagcaaagaagaactgttctgtcagatagctcttacgctcagcgcaagaagaaatatccaccgtgggaaaaactccaggtagaggtacacacgcggatagccaattcagagtaataaactgtgataatcaaccctcatcaatgatgacgaactaacccccgatatcaggtcacatgacgaagggaaagagaaggaaatcaactgtgacaaactgccctcaaatttggcttccttaaaaattacagttcaaaaagtatgagaaaatccatgcaggctgaaggaaacagcaaaactgtgacaaattaccctcagtaggtcagaacaaatgtgacgaaccaccctcaaatctgtgacagataaccctcagactatcctgtcgtcatggaagtgatatcgcggaaggaaaatacgatatgagtcgtctggcggcctttctttttctcaatgtatgagaggcgcattggagttctgctgttgatctcattaacacagacctgcaggaagcggcggcggaagtcaggcatacgctggtaactttgaggcagctggtaacgctctatgatccagtcgattttcagagagacgatgcctgagccatccggcttacgatactgacacagggattcgtataaacgcatggcatacggattggtgatttcttttgtttcactaagccgaaactgcgtaaaccggttctgtaacccgataaagaagggaatgagatatgggttgatatgtacactgtaaagccctctggatggactgtgcgcacgtttgataaaccaaggaaaagattcatagcctttttcatcgccggcatcctcttcagggcgataaaaaaccacttccttccccgcgaaactcttcaatgcctgccgtatatccttactggcttccgcagaggtcaatccgaatatttcagcatatttagcaacatggatctcgcagataccgtcatgttcctgtagggtgccatcagattttctgatctggtcaacgaacagatacagcatacgtttttgatcccgggagagactatatgccgcctcagtgaggtcgtttgactggacgattcgcgggctatttttacgtttcttgtgattgataaccgctgtttccgccatgacagatccatgtgaagtgtgacaagtttttagattgtcacactaaataaaaaagagtcaataagcagggataactttgtgaaaaaacagcttcttctgagggcaatttgtcacagggttaagggcaatttgtcacagACAGGACTGTCATTTGAGGGTGATTTGTCACACTGAAAGGGCAATTTGTCACAACACCTTCTCTAGAACCAGCATGGATAAAGGCCTACAAGGCGCTCTAAAAAAGAAGATCTAAAAACTATAAAAAAAATAATTATAAAAATATCCCCGTGGATAAGTGGATAACCCCAAGGGAAGTTTTTTCAGGCATCGTGTGTAAGCAGAATATATAAGTGCTGTTCCCTGGTGCTTCCTCGCTCACTCGAGGGCTTCGCCCTGTCGCTCAACTGCGGCGAGCACTACTGGCTGTAAaaggacagaccacatcatggttctgtgttcattaggttgttctgtccattgctgacataatccgctccacttcaacgtaacaccgcacgaagatttctattgttcctgaaggcatattcaaatcgttttcgttaccgcttgcaggcatcatgacagaacactacttcctataaacgctacacaggctcctgagattaataatgcggatctctacgataatgggagattttcccgactgtttcgttcgcttctcagtggataacagccagcttctctgtttaacagacaaaaacagcatatccactcagttccacatttccatataaaggccaaggcatttattctcaggataattgtttcagcatcgcaaccgcatcagactccggcatcgcaaactgcacccggtgccgggcagccacatccagcgcaaaaaccttcgtgtagacttccgttgaactgatggacttatgtcccatcaggctttgcagaactttcagcggtataccggcatacagcatgtgcatcgcataggaatggcggaacgtatgtggtgtgaccggaacagagaacgtcacaccgtcagcagcagcggcggcaaccgcctccccaatccaggtcctgaccgttctgtccgtcacttcccagatccgcgctttctctgtccttcctgtgcgacggttacgccgctccatgagcttatcgcgaataaatacctgtgacggaagatcacttcgcagaataaataaatcctggtgtccctgttgataccgggaagccctgggccaacttttggcgaaaatgagacgttgatcggcacgtaagaggttccaactttcaccataatgaaataagatcactaccgggcgtattttttgagttatcgagattttcaggagctaaggaagctaaaatggagaaaaaaatcactggatataccaccgttgatatatcccaatggcatcgtaaagaacattttgaggcatttcagtcagttgctcaatgtacctataaccagaccgttcagctggatattacggcctttttaaagaccgtaaagaaaaataagcacaagttttatccggcctttattcacattcttgcccgcctgatgaatgctcatccggagttccgtatggcaatgaaagacggtgagctggtgatatgggatagtgttcacccttgttacaccgttttccatgagcaaactgaaacgttttcatcgctctggagtgaataccacgacgatttccggcagtttctacacatatattcgcaagatgtggcgtgttacggtgaaaacctggcctatttccctaaagggtttattgagaatatgtttttcgtctcagccaatccctgggtgagtttcaccagttttgatttaaacgtggccaatatggacaacttcttcgcccccgttttcaccatgggcaaatattatacgcaaggcgacaaggtgctgatgccgctggcgattcaggttcatcatgccgtttgtgatggcttccatgtcggcagaatgcttaatgaattacaacagtactgcgatgagtggcagggcggggcgtaatttttttaaggcagttattggtgcccttaaacgcctggttgctacgcctgaataagtgataataagcggatgaatggcagaaattcgatgataagctgtcaaacatgagaattggtcgacggcgcgccaaagcttgcatgcctgcagccgcgtaacctggcaaaatcggttacggttgagtaataaatggatgccctgcgtaagcggggcacatttcattacctctttctccgcacccgacatagataataacttcgtatagtatacattatacgaagttatctagtagacttaattaaggatcgatccggcgcgccaatagtcatgccccgcgcccaccggaaggagctgactgggttgaaggctctcaagggcatcggtcgagcttgacattgtaggactatattgctctaataaatttgcggccgctaatacgactcactatagggagag |
| pRC1877  (pMAL-c2x, Hsmar1 E2K, no MBP-tag) | CCGACACCATCGAATGGTGCAAAACCTTTCGCGGTATGGCATGATAGCGCCCGGAAGAGAGTCAATTCAGGGTGGTGAATGTGAAACCAGTAACGTTATACGATGTCGCAGAGTATGCCGGTGTCTCTTATCAGACCGTTTCCCGCGTGGTGAACCAGGCCAGCCACGTTTCTGCGAAAACGCGGGAAAAAGTGGAAGCGGCGATGGCGGAGCTGAATTACATTCCCAACCGCGTGGCACAACAACTGGCGGGCAAACAGTCGTTGCTGATTGGCGTTGCCACCTCCAGTCTGGCCCTGCACGCGCCGTCGCAAATTGTCGCGGCGATTAAATCTCGCGCCGATCAACTGGGTGCCAGCGTGGTGGTGTCGATGGTAGAACGAAGCGGCGTCGAAGCCTGTAAAGCGGCGGTGCACAATCTTCTCGCGCAACGCGTCAGTGGGCTGATCATTAACTATCCGCTGGATGACCAGGATGCCATTGCTGTGGAAGCTGCCTGCACTAATGTTCCGGCGTTATTTCTTGATGTCTCTGACCAGACACCCATCAACAGTATTATTTTCTCCCATGAAGACGGTACGCGACTGGGCGTGGAGCATCTGGTCGCATTGGGTCACCAGCAAATCGCGCTGTTAGCGGGCCCATTAAGTTCTGTCTCGGCGCGTCTGCGTCTGGCTGGCTGGCATAAATATCTCACTCGCAATCAAATTCAGCCGATAGCGGAACGGGAAGGCGACTGGAGTGCCATGTCCGGTTTTCAACAAACCATGCAAATGCTGAATGAGGGCATCGTTCCCACTGCGATGCTGGTTGCCAACGATCAGATGGCGCTGGGCGCAATGCGCGCCATTACCGAGTCCGGGCTGCGCGTTGGTGCGGATATCTCGGTAGTGGGATACGACGATACCGAAGACAGCTCATGTTATATCCCGCCGTTAACCACCATCAAACAGGATTTTCGCCTGCTGGGGCAAACCAGCGTGGACCGCTTGCTGCAACTCTCTCAGGGCCAGGCGGTGAAGGGCAATCAGCTGTTGCCCGTCTCACTGGTGAAAAGAAAAACCACCCTGGCGCCCAATACGCAAACCGCCTCTCCCCGCGCGTTGGCCGATTCATTAATGCAGCTGGCACGACAGGTTTCCCGACTGGAAAGCGGGCAGTGAGCGCAACGCAATTAATGTGAGTTAGCTCACTCATTAGGCACAATTCTCATGTTTGACAGCTTATCATCGACTGCACGGTGCACCAATGCTTCTGGCGTCAGGCAGCCATCGGAAGCTGTGGTATGGCTGTGCAGGTCGTAAATCACTGCATAATTCGTGTCGCTCAAGGCGCACTCCCGTTCTGGATAATGTTTTTTGCGCCGACATCATAACGGTTCTGGCAAATATTCTGAAATGAGCTGTTGACAATTAATCATCGGCTCGTATAATGTGTGGAATTGTGAGCGGATAACAATTTCACACAGGAAACAGCCAGTCCGTTTAGGTGTTTTCACGAGCACTTCACCAACAAGGACCATAGCATATGaAAATGATGCTCGATAAGAAACAGATTCGTGCGATCTTTCTCTTTGAGTTTAAAATGGGTCGCAAAGCGGCGGAGACGACGCGTAATATTAACAACGCGTTCGGTCCTGGCACCGCGAACGAGCGTACCGTGCAATGGTGGTTCAAAAAGTTTCGCAAAGGCGACGAATCTCTGGAGGACGAAGAGCGTTCTGGCCGCCCGTCCGAGGTTGACAACGACCAGCTGCGTGCAATCATCGAAGCTGATCCGCTGACTACCACCCGCGAAGTTGCTGAAGAACTGAATGTGGATCACTCTACTGTGGTTCGCCACCTGAAACAGATCGGTAAAGTAAAAAAACTGGACAAATGGGTTCCTCATGAACTGTCTGAAAACCAGAAAAACCGTCGTTTCGAAGTTAGCTCCTCTCTGATTCTGCGTAACAACAACGAACCGTTCCTGGATCGTATCGTAACCTGTGATGAGAAATGGATTCTGTATGATAACCGTCGCCGCTCTGCTCAGTGGCTGGATCGCGAAGAAGCTCCAAAACACTTCCCGAAACCGAATCTGCACCAGAAGAAAGTCATGGTAACCGTATGGTGGTCTGCCGCAGGTGTTATCCACTATTCCTTCCTGAACCCGGGCGAAACTATCACCAGCGAAAAATACTGCCAGCAGATTGACGAAATGCACCGTAAACTGCAGCGTCTGCAGCCAGCACTGGTGAATCGTAAAGGTCCGATCCTGCTGCATGATAACGCCCGTCCGCACGTTGCCCAACCGACCCTGCAGAAACTGAACGAACTGGGCTATGAAGTTCTGCCACACCCGCCGTACTCCCCGGATCTGTCCCCGACTGACTACCATTTCTTCAAGCATCTGGACAACTTCCTGCAGGGTAAACGTTTTCACAACCAACAGGACGCAGAAAACGCTTTCCAGGAGTTCGTCGAAAGCCGTTCCACTGACTTCTACGCGACCGGTATCAACAAGCTGATCAGCCGTTGGCAGAAATGCGTGGACTGTAACGGCAGCTACTTCGATTAAGGATCCTCTAGAGTCGACCTGCAGGCAAGCTTGGCACTGGCCGTCGTTTTACAACGTCGTGACTGGGAAAACCCTGGCGTTACCCAACTTAATCGCCTTGCAGCACATCCCCCTTTCGCCAGCTGGCGTAATAGCGAAGAGGCCCGCACCGATCGCCCTTCCCAACAGTTGCGCAGCCTGAATGGCGAATGGCAGCTTGGCTGTTTTGGCGGATGAGATAAGATTTTCAGCCTGATACAGATTAAATCAGAACGCAGAAGCGGTCTGATAAAACAGAATTTGCCTGGCGGCAGTAGCGCGGTGGTCCCACCTGACCCCATGCCGAACTCAGAAGTGAAACGCCGTAGCGCCGATGGTAGTGTGGGGTCTCCCCATGCGAGAGTAGGGAACTGCCAGGCATCAAATAAAACGAAAGGCTCAGTCGAAAGACTGGGCCTTTCGTTTTATCTGTTGTTTGTCGGTGAACGCTCTCCTGAGTAGGACAAATCCGCCGGGAGCGGATTTGAACGTTGCGAAGCAACGGCCCGGAGGGTGGCGGGCAGGACGCCCGCCATAAACTGCCAGGCATCAAATTAAGCAGAAGGCCATCCTGACGGATGGCCTTTTTGCGTTTCTACAAACTCTTTTTGTTTATTTTTCTAAATACATTCAAATATGTATCCGCTCATGAGACAATAACCCTGATAAATGCTTCAATAATATTGAAAAAGGAAGAGTATGAGTATTCAACATTTCCGTGTCGCCCTTATTCCCTTTTTTGCGGCATTTTGCCTTCCTGTTTTTGCTCACCCAGAAACGCTGGTGAAAGTAAAAGATGCTGAAGATCAGTTGGGTGCACGAGTGGGTTACATCGAACTGGATCTCAACAGCGGTAAGATCCTTGAGAGTTTTCGCCCCGAAGAACGTTCTCCAATGATGAGCACTTTTAAAGTTCTGCTATGTGGCGCGGTATTATCCCGTGTTGACGCCGGGCAAGAGCAACTCGGTCGCCGCATACACTATTCTCAGAATGACTTGGTTGAGTACTCACCAGTCACAGAAAAGCATCTTACGGATGGCATGACAGTAAGAGAATTATGCAGTGCTGCCATAACCATGAGTGATAACACTGCGGCCAACTTACTTCTGACAACGATCGGAGGACCGAAGGAGCTAACCGCTTTTTTGCACAACATGGGGGATCATGTAACTCGCCTTGATCGTTGGGAACCGGAGCTGAATGAAGCCATACCAAACGACGAGCGTGACACCACGATGCCTGTAGCAATGGCAACAACGTTGCGCAAACTATTAACTGGCGAACTACTTACTCTAGCTTCCCGGCAACAATTAATAGACTGGATGGAGGCGGATAAAGTTGCAGGACCACTTCTGCGCTCGGCCCTTCCGGCTGGCTGGTTTATTGCTGATAAATCTGGAGCCGGTGAGCGTGGGTCTCGCGGTATCATTGCAGCACTGGGGCCAGATGGTAAGCCCTCCCGTATCGTAGTTATCTACACGACGGGGAGTCAGGCAACTATGGATGAACGAAATAGACAGATCGCTGAGATAGGTGCCTCACTGATTAAGCATTGGTAACTGTCAGACCAAGTTTACTCATATATACTTTAGATTGATTTACCCCGGTTGATAATCAGAAAAGCCCCAAAAACAGGAAGATTGTATAAGCAAATATTTAAATTGTAAACGTTAATATTTTGTTAAAATTCGCGTTAAATTTTTGTTAAATCAGCTCATTTTTTAACCAATAGGCCGAAATCGGCAAAATCCCTTATAAATCAAAAGAATAGACCGAGATAGGGTTGAGTGTTGTTCCAGTTTGGAACAAGAGTCCACTATTAAAGAACGTGGACTCCAACGTCAAAGGGCGAAAAACCGTCTATCAGGGCGATGGCCCACTACGTGAACCATCACCCAAATCAAGTTTTTTGGGGTCGAGGTGCCGTAAAGCACTAAATCGGAACCCTAAAGGGAGCCCCCGATTTAGAGCTTGACGGGGAAAGCCGGCGAACGTGGCGAGAAAGGAAGGGAAGAAAGCGAAAGGAGCGGGCGCTAGGGCGCTGGCAAGTGTAGCGGTCACGCTGCGCGTAACCACCACACCCGCCGCGCTTAATGCGCCGCTACAGGGCGCGTAAAAGGATCTAGGTGAAGATCCTTTTTGATAATCTCATGACCAAAATCCCTTAACGTGAGTTTTCGTTCCACTGAGCGTCAGACCCCGTAGAAAAGATCAAAGGATCTTCTTGAGATCCTTTTTTTCTGCGCGTAATCTGCTGCTTGCAAACAAAAAAACCACCGCTACCAGCGGTGGTTTGTTTGCCGGATCAAGAGCTACCAACTCTTTTTCCGAAGGTAACTGGCTTCAGCAGAGCGCAGATACCAAATACTGTCCTTCTAGTGTAGCCGTAGTTAGGCCACCACTTCAAGAACTCTGTAGCACCGCCTACATACCTCGCTCTGCTAATCCTGTTACCAGTGGCTGCTGCCAGTGGCGATAAGTCGTGTCTTACCGGGTTGGACTCAAGACGATAGTTACCGGATAAGGCGCAGCGGTCGGGCTGAACGGGGGGTTCGTGCACACAGCCCAGCTTGGAGCGAACGACCTACACCGAACTGAGATACCTACAGCGTGAGCTATGAGAAAGCGCCACGCTTCCCGAAGGGAGAAAGGCGGACAGGTATCCGGTAAGCGGCAGGGTCGGAACAGGAGAGCGCACGAGGGAGCTTCCAGGGGGAAACGCCTGGTATCTTTATAGTCCTGTCGGGTTTCGCCACCTCTGACTTGAGCGTCGATTTTTGTGATGCTCGTCAGGGGGGCGGAGCCTATGGAAAAACGCCAGCAACGCGGCCTTTTTACGGTTCCTGGCCTTTTGCTGGCCTTTTGCTCACATGTTCTTTCCTGCGTTATCCCCTGATTCTGTGGATAACCGTATTACCGCCTTTGAGTGAGCTGATACCGCTCGCCGCAGCCGAACGACCGAGCGCAGCGAGTCAGTGAGCGAGGAAGCGGAAGAGCGCCTGATGCGGTATTTTCTCCTTACGCATCTGTGCGGTATTTCACACCGCATATATGGTGCACTCTCAGTACAATCTGCTCTGATGCCGCATAGTTAAGCCAGTATACACTCCGCTATCGCTACGTGACTGGGTCATGGCTGCGCCCCGACACCCGCCAACACCCGCTGACGCGCCCTGACGGGCTTGTCTGCTCCCGGCATCCGCTTACAGACAAGCTGTGACCGTCTCCGGGAGCTGCATGTGTCAGAGGTTTTCACCGTCATCACCGAAACGCGCGAGGCAGCTGCGGTAAAGCTCATCAGCGTGGTCGTGCAGCGATTCACAGATGTCTGCCTGTTCATCCGCGTCCAGCTCGTTGAGTTTCTCCAGAAGCGTTAATGTCTGGCTTCTGATAAAGCGGGCCATGTTAAGGGCGGTTTTTTCCTGTTTGGTCACTTGATGCCTCCGTGTAAGGGGGAATTTCTGTTCATGGGGGTAATGATACCGATGAAACGAGAGAGGATGCTCACGATACGGGTTACTGATGATGAACATGCCCGGTTACTGGAACGTTGTGAGGGTAAACAACTGGCGGTATGGATGCGGCGGGACCAGAGAAAAATCACTCAGGGTCAATGCCAGCGCTTCGTTAATACAGATGTAGGTGTTCCACAGGGTAGCCAGCAGCATCCTGCGATGCAGATCCGGAACATAATGGTGCAGGGCGCTGACTTCCGCGTTTCCAGACTTTACGAAACACGGAAACCGAAGACCATTCATGTTGTTGCTCAGGTCGCAGACGTTTTGCAGCAGCAGTCGCTTCACGTTCGCTCGCGTATCGGTGATTCATTCTGCTAACCAGTAAGGCAACCCCGCCAGCCTAGCCGGGTCCTCAACGACAGGAGCACGATCATGCGCACCCGTGGCCAGGACCCAACGCTGCCCGAAATT |
| pRC1878  (pMAL-c2x, Hsmar1 R53C, no MBP-tag) | CCGACACCATCGAATGGTGCAAAACCTTTCGCGGTATGGCATGATAGCGCCCGGAAGAGAGTCAATTCAGGGTGGTGAATGTGAAACCAGTAACGTTATACGATGTCGCAGAGTATGCCGGTGTCTCTTATCAGACCGTTTCCCGCGTGGTGAACCAGGCCAGCCACGTTTCTGCGAAAACGCGGGAAAAAGTGGAAGCGGCGATGGCGGAGCTGAATTACATTCCCAACCGCGTGGCACAACAACTGGCGGGCAAACAGTCGTTGCTGATTGGCGTTGCCACCTCCAGTCTGGCCCTGCACGCGCCGTCGCAAATTGTCGCGGCGATTAAATCTCGCGCCGATCAACTGGGTGCCAGCGTGGTGGTGTCGATGGTAGAACGAAGCGGCGTCGAAGCCTGTAAAGCGGCGGTGCACAATCTTCTCGCGCAACGCGTCAGTGGGCTGATCATTAACTATCCGCTGGATGACCAGGATGCCATTGCTGTGGAAGCTGCCTGCACTAATGTTCCGGCGTTATTTCTTGATGTCTCTGACCAGACACCCATCAACAGTATTATTTTCTCCCATGAAGACGGTACGCGACTGGGCGTGGAGCATCTGGTCGCATTGGGTCACCAGCAAATCGCGCTGTTAGCGGGCCCATTAAGTTCTGTCTCGGCGCGTCTGCGTCTGGCTGGCTGGCATAAATATCTCACTCGCAATCAAATTCAGCCGATAGCGGAACGGGAAGGCGACTGGAGTGCCATGTCCGGTTTTCAACAAACCATGCAAATGCTGAATGAGGGCATCGTTCCCACTGCGATGCTGGTTGCCAACGATCAGATGGCGCTGGGCGCAATGCGCGCCATTACCGAGTCCGGGCTGCGCGTTGGTGCGGATATCTCGGTAGTGGGATACGACGATACCGAAGACAGCTCATGTTATATCCCGCCGTTAACCACCATCAAACAGGATTTTCGCCTGCTGGGGCAAACCAGCGTGGACCGCTTGCTGCAACTCTCTCAGGGCCAGGCGGTGAAGGGCAATCAGCTGTTGCCCGTCTCACTGGTGAAAAGAAAAACCACCCTGGCGCCCAATACGCAAACCGCCTCTCCCCGCGCGTTGGCCGATTCATTAATGCAGCTGGCACGACAGGTTTCCCGACTGGAAAGCGGGCAGTGAGCGCAACGCAATTAATGTGAGTTAGCTCACTCATTAGGCACAATTCTCATGTTTGACAGCTTATCATCGACTGCACGGTGCACCAATGCTTCTGGCGTCAGGCAGCCATCGGAAGCTGTGGTATGGCTGTGCAGGTCGTAAATCACTGCATAATTCGTGTCGCTCAAGGCGCACTCCCGTTCTGGATAATGTTTTTTGCGCCGACATCATAACGGTTCTGGCAAATATTCTGAAATGAGCTGTTGACAATTAATCATCGGCTCGTATAATGTGTGGAATTGTGAGCGGATAACAATTTCACACAGGAAACAGCCAGTCCGTTTAGGTGTTTTCACGAGCACTTCACCAACAAGGACCATAGCATATGGAAATGATGCTCGATAAGAAACAGATTCGTGCGATCTTTCTCTTTGAGTTTAAAATGGGTCGCAAAGCGGCGGAGACGACGCGTAATATTAACAACGCGTTCGGTCCTGGCACCGCGAACGAGCGTACCGTGCAATGGTGGTTCAAAAAGTTTtGCAAAGGCGACGAATCTCTGGAGGACGAAGAGCGTTCTGGCCGCCCGTCCGAGGTTGACAACGACCAGCTGCGTGCAATCATCGAAGCTGATCCGCTGACTACCACCCGCGAAGTTGCTGAAGAACTGAATGTGGATCACTCTACTGTGGTTCGCCACCTGAAACAGATCGGTAAAGTAAAAAAACTGGACAAATGGGTTCCTCATGAACTGTCTGAAAACCAGAAAAACCGTCGTTTCGAAGTTAGCTCCTCTCTGATTCTGCGTAACAACAACGAACCGTTCCTGGATCGTATCGTAACCTGTGATGAGAAATGGATTCTGTATGATAACCGTCGCCGCTCTGCTCAGTGGCTGGATCGCGAAGAAGCTCCAAAACACTTCCCGAAACCGAATCTGCACCAGAAGAAAGTCATGGTAACCGTATGGTGGTCTGCCGCAGGTGTTATCCACTATTCCTTCCTGAACCCGGGCGAAACTATCACCAGCGAAAAATACTGCCAGCAGATTGACGAAATGCACCGTAAACTGCAGCGTCTGCAGCCAGCACTGGTGAATCGTAAAGGTCCGATCCTGCTGCATGATAACGCCCGTCCGCACGTTGCCCAACCGACCCTGCAGAAACTGAACGAACTGGGCTATGAAGTTCTGCCACACCCGCCGTACTCCCCGGATCTGTCCCCGACTGACTACCATTTCTTCAAGCATCTGGACAACTTCCTGCAGGGTAAACGTTTTCACAACCAACAGGACGCAGAAAACGCTTTCCAGGAGTTCGTCGAAAGCCGTTCCACTGACTTCTACGCGACCGGTATCAACAAGCTGATCAGCCGTTGGCAGAAATGCGTGGACTGTAACGGCAGCTACTTCGATTAAGGATCCTCTAGAGTCGACCTGCAGGCAAGCTTGGCACTGGCCGTCGTTTTACAACGTCGTGACTGGGAAAACCCTGGCGTTACCCAACTTAATCGCCTTGCAGCACATCCCCCTTTCGCCAGCTGGCGTAATAGCGAAGAGGCCCGCACCGATCGCCCTTCCCAACAGTTGCGCAGCCTGAATGGCGAATGGCAGCTTGGCTGTTTTGGCGGATGAGATAAGATTTTCAGCCTGATACAGATTAAATCAGAACGCAGAAGCGGTCTGATAAAACAGAATTTGCCTGGCGGCAGTAGCGCGGTGGTCCCACCTGACCCCATGCCGAACTCAGAAGTGAAACGCCGTAGCGCCGATGGTAGTGTGGGGTCTCCCCATGCGAGAGTAGGGAACTGCCAGGCATCAAATAAAACGAAAGGCTCAGTCGAAAGACTGGGCCTTTCGTTTTATCTGTTGTTTGTCGGTGAACGCTCTCCTGAGTAGGACAAATCCGCCGGGAGCGGATTTGAACGTTGCGAAGCAACGGCCCGGAGGGTGGCGGGCAGGACGCCCGCCATAAACTGCCAGGCATCAAATTAAGCAGAAGGCCATCCTGACGGATGGCCTTTTTGCGTTTCTACAAACTCTTTTTGTTTATTTTTCTAAATACATTCAAATATGTATCCGCTCATGAGACAATAACCCTGATAAATGCTTCAATAATATTGAAAAAGGAAGAGTATGAGTATTCAACATTTCCGTGTCGCCCTTATTCCCTTTTTTGCGGCATTTTGCCTTCCTGTTTTTGCTCACCCAGAAACGCTGGTGAAAGTAAAAGATGCTGAAGATCAGTTGGGTGCACGAGTGGGTTACATCGAACTGGATCTCAACAGCGGTAAGATCCTTGAGAGTTTTCGCCCCGAAGAACGTTCTCCAATGATGAGCACTTTTAAAGTTCTGCTATGTGGCGCGGTATTATCCCGTGTTGACGCCGGGCAAGAGCAACTCGGTCGCCGCATACACTATTCTCAGAATGACTTGGTTGAGTACTCACCAGTCACAGAAAAGCATCTTACGGATGGCATGACAGTAAGAGAATTATGCAGTGCTGCCATAACCATGAGTGATAACACTGCGGCCAACTTACTTCTGACAACGATCGGAGGACCGAAGGAGCTAACCGCTTTTTTGCACAACATGGGGGATCATGTAACTCGCCTTGATCGTTGGGAACCGGAGCTGAATGAAGCCATACCAAACGACGAGCGTGACACCACGATGCCTGTAGCAATGGCAACAACGTTGCGCAAACTATTAACTGGCGAACTACTTACTCTAGCTTCCCGGCAACAATTAATAGACTGGATGGAGGCGGATAAAGTTGCAGGACCACTTCTGCGCTCGGCCCTTCCGGCTGGCTGGTTTATTGCTGATAAATCTGGAGCCGGTGAGCGTGGGTCTCGCGGTATCATTGCAGCACTGGGGCCAGATGGTAAGCCCTCCCGTATCGTAGTTATCTACACGACGGGGAGTCAGGCAACTATGGATGAACGAAATAGACAGATCGCTGAGATAGGTGCCTCACTGATTAAGCATTGGTAACTGTCAGACCAAGTTTACTCATATATACTTTAGATTGATTTACCCCGGTTGATAATCAGAAAAGCCCCAAAAACAGGAAGATTGTATAAGCAAATATTTAAATTGTAAACGTTAATATTTTGTTAAAATTCGCGTTAAATTTTTGTTAAATCAGCTCATTTTTTAACCAATAGGCCGAAATCGGCAAAATCCCTTATAAATCAAAAGAATAGACCGAGATAGGGTTGAGTGTTGTTCCAGTTTGGAACAAGAGTCCACTATTAAAGAACGTGGACTCCAACGTCAAAGGGCGAAAAACCGTCTATCAGGGCGATGGCCCACTACGTGAACCATCACCCAAATCAAGTTTTTTGGGGTCGAGGTGCCGTAAAGCACTAAATCGGAACCCTAAAGGGAGCCCCCGATTTAGAGCTTGACGGGGAAAGCCGGCGAACGTGGCGAGAAAGGAAGGGAAGAAAGCGAAAGGAGCGGGCGCTAGGGCGCTGGCAAGTGTAGCGGTCACGCTGCGCGTAACCACCACACCCGCCGCGCTTAATGCGCCGCTACAGGGCGCGTAAAAGGATCTAGGTGAAGATCCTTTTTGATAATCTCATGACCAAAATCCCTTAACGTGAGTTTTCGTTCCACTGAGCGTCAGACCCCGTAGAAAAGATCAAAGGATCTTCTTGAGATCCTTTTTTTCTGCGCGTAATCTGCTGCTTGCAAACAAAAAAACCACCGCTACCAGCGGTGGTTTGTTTGCCGGATCAAGAGCTACCAACTCTTTTTCCGAAGGTAACTGGCTTCAGCAGAGCGCAGATACCAAATACTGTCCTTCTAGTGTAGCCGTAGTTAGGCCACCACTTCAAGAACTCTGTAGCACCGCCTACATACCTCGCTCTGCTAATCCTGTTACCAGTGGCTGCTGCCAGTGGCGATAAGTCGTGTCTTACCGGGTTGGACTCAAGACGATAGTTACCGGATAAGGCGCAGCGGTCGGGCTGAACGGGGGGTTCGTGCACACAGCCCAGCTTGGAGCGAACGACCTACACCGAACTGAGATACCTACAGCGTGAGCTATGAGAAAGCGCCACGCTTCCCGAAGGGAGAAAGGCGGACAGGTATCCGGTAAGCGGCAGGGTCGGAACAGGAGAGCGCACGAGGGAGCTTCCAGGGGGAAACGCCTGGTATCTTTATAGTCCTGTCGGGTTTCGCCACCTCTGACTTGAGCGTCGATTTTTGTGATGCTCGTCAGGGGGGCGGAGCCTATGGAAAAACGCCAGCAACGCGGCCTTTTTACGGTTCCTGGCCTTTTGCTGGCCTTTTGCTCACATGTTCTTTCCTGCGTTATCCCCTGATTCTGTGGATAACCGTATTACCGCCTTTGAGTGAGCTGATACCGCTCGCCGCAGCCGAACGACCGAGCGCAGCGAGTCAGTGAGCGAGGAAGCGGAAGAGCGCCTGATGCGGTATTTTCTCCTTACGCATCTGTGCGGTATTTCACACCGCATATATGGTGCACTCTCAGTACAATCTGCTCTGATGCCGCATAGTTAAGCCAGTATACACTCCGCTATCGCTACGTGACTGGGTCATGGCTGCGCCCCGACACCCGCCAACACCCGCTGACGCGCCCTGACGGGCTTGTCTGCTCCCGGCATCCGCTTACAGACAAGCTGTGACCGTCTCCGGGAGCTGCATGTGTCAGAGGTTTTCACCGTCATCACCGAAACGCGCGAGGCAGCTGCGGTAAAGCTCATCAGCGTGGTCGTGCAGCGATTCACAGATGTCTGCCTGTTCATCCGCGTCCAGCTCGTTGAGTTTCTCCAGAAGCGTTAATGTCTGGCTTCTGATAAAGCGGGCCATGTTAAGGGCGGTTTTTTCCTGTTTGGTCACTTGATGCCTCCGTGTAAGGGGGAATTTCTGTTCATGGGGGTAATGATACCGATGAAACGAGAGAGGATGCTCACGATACGGGTTACTGATGATGAACATGCCCGGTTACTGGAACGTTGTGAGGGTAAACAACTGGCGGTATGGATGCGGCGGGACCAGAGAAAAATCACTCAGGGTCAATGCCAGCGCTTCGTTAATACAGATGTAGGTGTTCCACAGGGTAGCCAGCAGCATCCTGCGATGCAGATCCGGAACATAATGGTGCAGGGCGCTGACTTCCGCGTTTCCAGACTTTACGAAACACGGAAACCGAAGACCATTCATGTTGTTGCTCAGGTCGCAGACGTTTTGCAGCAGCAGTCGCTTCACGTTCGCTCGCGTATCGGTGATTCATTCTGCTAACCAGTAAGGCAACCCCGCCAGCCTAGCCGGGTCCTCAACGACAGGAGCACGATCATGCGCACCCGTGGCCAGGACCCAACGCTGCCCGAAATT |
| pRC1879  (pMAL-c2x, Hsmar1 D98N, no MBP-tag) | CCGACACCATCGAATGGTGCAAAACCTTTCGCGGTATGGCATGATAGCGCCCGGAAGAGAGTCAATTCAGGGTGGTGAATGTGAAACCAGTAACGTTATACGATGTCGCAGAGTATGCCGGTGTCTCTTATCAGACCGTTTCCCGCGTGGTGAACCAGGCCAGCCACGTTTCTGCGAAAACGCGGGAAAAAGTGGAAGCGGCGATGGCGGAGCTGAATTACATTCCCAACCGCGTGGCACAACAACTGGCGGGCAAACAGTCGTTGCTGATTGGCGTTGCCACCTCCAGTCTGGCCCTGCACGCGCCGTCGCAAATTGTCGCGGCGATTAAATCTCGCGCCGATCAACTGGGTGCCAGCGTGGTGGTGTCGATGGTAGAACGAAGCGGCGTCGAAGCCTGTAAAGCGGCGGTGCACAATCTTCTCGCGCAACGCGTCAGTGGGCTGATCATTAACTATCCGCTGGATGACCAGGATGCCATTGCTGTGGAAGCTGCCTGCACTAATGTTCCGGCGTTATTTCTTGATGTCTCTGACCAGACACCCATCAACAGTATTATTTTCTCCCATGAAGACGGTACGCGACTGGGCGTGGAGCATCTGGTCGCATTGGGTCACCAGCAAATCGCGCTGTTAGCGGGCCCATTAAGTTCTGTCTCGGCGCGTCTGCGTCTGGCTGGCTGGCATAAATATCTCACTCGCAATCAAATTCAGCCGATAGCGGAACGGGAAGGCGACTGGAGTGCCATGTCCGGTTTTCAACAAACCATGCAAATGCTGAATGAGGGCATCGTTCCCACTGCGATGCTGGTTGCCAACGATCAGATGGCGCTGGGCGCAATGCGCGCCATTACCGAGTCCGGGCTGCGCGTTGGTGCGGATATCTCGGTAGTGGGATACGACGATACCGAAGACAGCTCATGTTATATCCCGCCGTTAACCACCATCAAACAGGATTTTCGCCTGCTGGGGCAAACCAGCGTGGACCGCTTGCTGCAACTCTCTCAGGGCCAGGCGGTGAAGGGCAATCAGCTGTTGCCCGTCTCACTGGTGAAAAGAAAAACCACCCTGGCGCCCAATACGCAAACCGCCTCTCCCCGCGCGTTGGCCGATTCATTAATGCAGCTGGCACGACAGGTTTCCCGACTGGAAAGCGGGCAGTGAGCGCAACGCAATTAATGTGAGTTAGCTCACTCATTAGGCACAATTCTCATGTTTGACAGCTTATCATCGACTGCACGGTGCACCAATGCTTCTGGCGTCAGGCAGCCATCGGAAGCTGTGGTATGGCTGTGCAGGTCGTAAATCACTGCATAATTCGTGTCGCTCAAGGCGCACTCCCGTTCTGGATAATGTTTTTTGCGCCGACATCATAACGGTTCTGGCAAATATTCTGAAATGAGCTGTTGACAATTAATCATCGGCTCGTATAATGTGTGGAATTGTGAGCGGATAACAATTTCACACAGGAAACAGCCAGTCCGTTTAGGTGTTTTCACGAGCACTTCACCAACAAGGACCATAGCATATGGAAATGATGCTCGATAAGAAACAGATTCGTGCGATCTTTCTCTTTGAGTTTAAAATGGGTCGCAAAGCGGCGGAGACGACGCGTAATATTAACAACGCGTTCGGTCCTGGCACCGCGAACGAGCGTACCGTGCAATGGTGGTTCAAAAAGTTTCGCAAAGGCGACGAATCTCTGGAGGACGAAGAGCGTTCTGGCCGCCCGTCCGAGGTTGACAACGACCAGCTGCGTGCAATCATCGAAGCTGATCCGCTGACTACCACCCGCGAAGTTGCTGAAGAACTGAATGTGaacCACTCTACTGTGGTTCGCCACCTGAAACAGATCGGTAAAGTAAAAAAACTGGACAAATGGGTTCCTCATGAACTGTCTGAAAACCAGAAAAACCGTCGTTTCGAAGTTAGCTCCTCTCTGATTCTGCGTAACAACAACGAACCGTTCCTGGATCGTATCGTAACCTGTGATGAGAAATGGATTCTGTATGATAACCGTCGCCGCTCTGCTCAGTGGCTGGATCGCGAAGAAGCTCCAAAACACTTCCCGAAACCGAATCTGCACCAGAAGAAAGTCATGGTAACCGTATGGTGGTCTGCCGCAGGTGTTATCCACTATTCCTTCCTGAACCCGGGCGAAACTATCACCAGCGAAAAATACTGCCAGCAGATTGACGAAATGCACCGTAAACTGCAGCGTCTGCAGCCAGCACTGGTGAATCGTAAAGGTCCGATCCTGCTGCATGATAACGCCCGTCCGCACGTTGCCCAACCGACCCTGCAGAAACTGAACGAACTGGGCTATGAAGTTCTGCCACACCCGCCGTACTCCCCGGATCTGTCCCCGACTGACTACCATTTCTTCAAGCATCTGGACAACTTCCTGCAGGGTAAACGTTTTCACAACCAACAGGACGCAGAAAACGCTTTCCAGGAGTTCGTCGAAAGCCGTTCCACTGACTTCTACGCGACCGGTATCAACAAGCTGATCAGCCGTTGGCAGAAATGCGTGGACTGTAACGGCAGCTACTTCGATTAAGGATCCTCTAGAGTCGACCTGCAGGCAAGCTTGGCACTGGCCGTCGTTTTACAACGTCGTGACTGGGAAAACCCTGGCGTTACCCAACTTAATCGCCTTGCAGCACATCCCCCTTTCGCCAGCTGGCGTAATAGCGAAGAGGCCCGCACCGATCGCCCTTCCCAACAGTTGCGCAGCCTGAATGGCGAATGGCAGCTTGGCTGTTTTGGCGGATGAGATAAGATTTTCAGCCTGATACAGATTAAATCAGAACGCAGAAGCGGTCTGATAAAACAGAATTTGCCTGGCGGCAGTAGCGCGGTGGTCCCACCTGACCCCATGCCGAACTCAGAAGTGAAACGCCGTAGCGCCGATGGTAGTGTGGGGTCTCCCCATGCGAGAGTAGGGAACTGCCAGGCATCAAATAAAACGAAAGGCTCAGTCGAAAGACTGGGCCTTTCGTTTTATCTGTTGTTTGTCGGTGAACGCTCTCCTGAGTAGGACAAATCCGCCGGGAGCGGATTTGAACGTTGCGAAGCAACGGCCCGGAGGGTGGCGGGCAGGACGCCCGCCATAAACTGCCAGGCATCAAATTAAGCAGAAGGCCATCCTGACGGATGGCCTTTTTGCGTTTCTACAAACTCTTTTTGTTTATTTTTCTAAATACATTCAAATATGTATCCGCTCATGAGACAATAACCCTGATAAATGCTTCAATAATATTGAAAAAGGAAGAGTATGAGTATTCAACATTTCCGTGTCGCCCTTATTCCCTTTTTTGCGGCATTTTGCCTTCCTGTTTTTGCTCACCCAGAAACGCTGGTGAAAGTAAAAGATGCTGAAGATCAGTTGGGTGCACGAGTGGGTTACATCGAACTGGATCTCAACAGCGGTAAGATCCTTGAGAGTTTTCGCCCCGAAGAACGTTCTCCAATGATGAGCACTTTTAAAGTTCTGCTATGTGGCGCGGTATTATCCCGTGTTGACGCCGGGCAAGAGCAACTCGGTCGCCGCATACACTATTCTCAGAATGACTTGGTTGAGTACTCACCAGTCACAGAAAAGCATCTTACGGATGGCATGACAGTAAGAGAATTATGCAGTGCTGCCATAACCATGAGTGATAACACTGCGGCCAACTTACTTCTGACAACGATCGGAGGACCGAAGGAGCTAACCGCTTTTTTGCACAACATGGGGGATCATGTAACTCGCCTTGATCGTTGGGAACCGGAGCTGAATGAAGCCATACCAAACGACGAGCGTGACACCACGATGCCTGTAGCAATGGCAACAACGTTGCGCAAACTATTAACTGGCGAACTACTTACTCTAGCTTCCCGGCAACAATTAATAGACTGGATGGAGGCGGATAAAGTTGCAGGACCACTTCTGCGCTCGGCCCTTCCGGCTGGCTGGTTTATTGCTGATAAATCTGGAGCCGGTGAGCGTGGGTCTCGCGGTATCATTGCAGCACTGGGGCCAGATGGTAAGCCCTCCCGTATCGTAGTTATCTACACGACGGGGAGTCAGGCAACTATGGATGAACGAAATAGACAGATCGCTGAGATAGGTGCCTCACTGATTAAGCATTGGTAACTGTCAGACCAAGTTTACTCATATATACTTTAGATTGATTTACCCCGGTTGATAATCAGAAAAGCCCCAAAAACAGGAAGATTGTATAAGCAAATATTTAAATTGTAAACGTTAATATTTTGTTAAAATTCGCGTTAAATTTTTGTTAAATCAGCTCATTTTTTAACCAATAGGCCGAAATCGGCAAAATCCCTTATAAATCAAAAGAATAGACCGAGATAGGGTTGAGTGTTGTTCCAGTTTGGAACAAGAGTCCACTATTAAAGAACGTGGACTCCAACGTCAAAGGGCGAAAAACCGTCTATCAGGGCGATGGCCCACTACGTGAACCATCACCCAAATCAAGTTTTTTGGGGTCGAGGTGCCGTAAAGCACTAAATCGGAACCCTAAAGGGAGCCCCCGATTTAGAGCTTGACGGGGAAAGCCGGCGAACGTGGCGAGAAAGGAAGGGAAGAAAGCGAAAGGAGCGGGCGCTAGGGCGCTGGCAAGTGTAGCGGTCACGCTGCGCGTAACCACCACACCCGCCGCGCTTAATGCGCCGCTACAGGGCGCGTAAAAGGATCTAGGTGAAGATCCTTTTTGATAATCTCATGACCAAAATCCCTTAACGTGAGTTTTCGTTCCACTGAGCGTCAGACCCCGTAGAAAAGATCAAAGGATCTTCTTGAGATCCTTTTTTTCTGCGCGTAATCTGCTGCTTGCAAACAAAAAAACCACCGCTACCAGCGGTGGTTTGTTTGCCGGATCAAGAGCTACCAACTCTTTTTCCGAAGGTAACTGGCTTCAGCAGAGCGCAGATACCAAATACTGTCCTTCTAGTGTAGCCGTAGTTAGGCCACCACTTCAAGAACTCTGTAGCACCGCCTACATACCTCGCTCTGCTAATCCTGTTACCAGTGGCTGCTGCCAGTGGCGATAAGTCGTGTCTTACCGGGTTGGACTCAAGACGATAGTTACCGGATAAGGCGCAGCGGTCGGGCTGAACGGGGGGTTCGTGCACACAGCCCAGCTTGGAGCGAACGACCTACACCGAACTGAGATACCTACAGCGTGAGCTATGAGAAAGCGCCACGCTTCCCGAAGGGAGAAAGGCGGACAGGTATCCGGTAAGCGGCAGGGTCGGAACAGGAGAGCGCACGAGGGAGCTTCCAGGGGGAAACGCCTGGTATCTTTATAGTCCTGTCGGGTTTCGCCACCTCTGACTTGAGCGTCGATTTTTGTGATGCTCGTCAGGGGGGCGGAGCCTATGGAAAAACGCCAGCAACGCGGCCTTTTTACGGTTCCTGGCCTTTTGCTGGCCTTTTGCTCACATGTTCTTTCCTGCGTTATCCCCTGATTCTGTGGATAACCGTATTACCGCCTTTGAGTGAGCTGATACCGCTCGCCGCAGCCGAACGACCGAGCGCAGCGAGTCAGTGAGCGAGGAAGCGGAAGAGCGCCTGATGCGGTATTTTCTCCTTACGCATCTGTGCGGTATTTCACACCGCATATATGGTGCACTCTCAGTACAATCTGCTCTGATGCCGCATAGTTAAGCCAGTATACACTCCGCTATCGCTACGTGACTGGGTCATGGCTGCGCCCCGACACCCGCCAACACCCGCTGACGCGCCCTGACGGGCTTGTCTGCTCCCGGCATCCGCTTACAGACAAGCTGTGACCGTCTCCGGGAGCTGCATGTGTCAGAGGTTTTCACCGTCATCACCGAAACGCGCGAGGCAGCTGCGGTAAAGCTCATCAGCGTGGTCGTGCAGCGATTCACAGATGTCTGCCTGTTCATCCGCGTCCAGCTCGTTGAGTTTCTCCAGAAGCGTTAATGTCTGGCTTCTGATAAAGCGGGCCATGTTAAGGGCGGTTTTTTCCTGTTTGGTCACTTGATGCCTCCGTGTAAGGGGGAATTTCTGTTCATGGGGGTAATGATACCGATGAAACGAGAGAGGATGCTCACGATACGGGTTACTGATGATGAACATGCCCGGTTACTGGAACGTTGTGAGGGTAAACAACTGGCGGTATGGATGCGGCGGGACCAGAGAAAAATCACTCAGGGTCAATGCCAGCGCTTCGTTAATACAGATGTAGGTGTTCCACAGGGTAGCCAGCAGCATCCTGCGATGCAGATCCGGAACATAATGGTGCAGGGCGCTGACTTCCGCGTTTCCAGACTTTACGAAACACGGAAACCGAAGACCATTCATGTTGTTGCTCAGGTCGCAGACGTTTTGCAGCAGCAGTCGCTTCACGTTCGCTCGCGTATCGGTGATTCATTCTGCTAACCAGTAAGGCAACCCCGCCAGCCTAGCCGGGTCCTCAACGACAGGAGCACGATCATGCGCACCCGTGGCCAGGACCCAACGCTGCCCGAAATT |
| pRC1880  (pMAL-c2x, Hsmar1 S124T, no MBP-tag) | CCGACACCATCGAATGGTGCAAAACCTTTCGCGGTATGGCATGATAGCGCCCGGAAGAGAGTCAATTCAGGGTGGTGAATGTGAAACCAGTAACGTTATACGATGTCGCAGAGTATGCCGGTGTCTCTTATCAGACCGTTTCCCGCGTGGTGAACCAGGCCAGCCACGTTTCTGCGAAAACGCGGGAAAAAGTGGAAGCGGCGATGGCGGAGCTGAATTACATTCCCAACCGCGTGGCACAACAACTGGCGGGCAAACAGTCGTTGCTGATTGGCGTTGCCACCTCCAGTCTGGCCCTGCACGCGCCGTCGCAAATTGTCGCGGCGATTAAATCTCGCGCCGATCAACTGGGTGCCAGCGTGGTGGTGTCGATGGTAGAACGAAGCGGCGTCGAAGCCTGTAAAGCGGCGGTGCACAATCTTCTCGCGCAACGCGTCAGTGGGCTGATCATTAACTATCCGCTGGATGACCAGGATGCCATTGCTGTGGAAGCTGCCTGCACTAATGTTCCGGCGTTATTTCTTGATGTCTCTGACCAGACACCCATCAACAGTATTATTTTCTCCCATGAAGACGGTACGCGACTGGGCGTGGAGCATCTGGTCGCATTGGGTCACCAGCAAATCGCGCTGTTAGCGGGCCCATTAAGTTCTGTCTCGGCGCGTCTGCGTCTGGCTGGCTGGCATAAATATCTCACTCGCAATCAAATTCAGCCGATAGCGGAACGGGAAGGCGACTGGAGTGCCATGTCCGGTTTTCAACAAACCATGCAAATGCTGAATGAGGGCATCGTTCCCACTGCGATGCTGGTTGCCAACGATCAGATGGCGCTGGGCGCAATGCGCGCCATTACCGAGTCCGGGCTGCGCGTTGGTGCGGATATCTCGGTAGTGGGATACGACGATACCGAAGACAGCTCATGTTATATCCCGCCGTTAACCACCATCAAACAGGATTTTCGCCTGCTGGGGCAAACCAGCGTGGACCGCTTGCTGCAACTCTCTCAGGGCCAGGCGGTGAAGGGCAATCAGCTGTTGCCCGTCTCACTGGTGAAAAGAAAAACCACCCTGGCGCCCAATACGCAAACCGCCTCTCCCCGCGCGTTGGCCGATTCATTAATGCAGCTGGCACGACAGGTTTCCCGACTGGAAAGCGGGCAGTGAGCGCAACGCAATTAATGTGAGTTAGCTCACTCATTAGGCACAATTCTCATGTTTGACAGCTTATCATCGACTGCACGGTGCACCAATGCTTCTGGCGTCAGGCAGCCATCGGAAGCTGTGGTATGGCTGTGCAGGTCGTAAATCACTGCATAATTCGTGTCGCTCAAGGCGCACTCCCGTTCTGGATAATGTTTTTTGCGCCGACATCATAACGGTTCTGGCAAATATTCTGAAATGAGCTGTTGACAATTAATCATCGGCTCGTATAATGTGTGGAATTGTGAGCGGATAACAATTTCACACAGGAAACAGCCAGTCCGTTTAGGTGTTTTCACGAGCACTTCACCAACAAGGACCATAGCATATGGAAATGATGCTCGATAAGAAACAGATTCGTGCGATCTTTCTCTTTGAGTTTAAAATGGGTCGCAAAGCGGCGGAGACGACGCGTAATATTAACAACGCGTTCGGTCCTGGCACCGCGAACGAGCGTACCGTGCAATGGTGGTTCAAAAAGTTTCGCAAAGGCGACGAATCTCTGGAGGACGAAGAGCGTTCTGGCCGCCCGTCCGAGGTTGACAACGACCAGCTGCGTGCAATCATCGAAGCTGATCCGCTGACTACCACCCGCGAAGTTGCTGAAGAACTGAATGTGGATCACTCTACTGTGGTTCGCCACCTGAAACAGATCGGTAAAGTAAAAAAACTGGACAAATGGGTTCCTCATGAACTGaCTGAAAACCAGAAAAACCGTCGTTTCGAAGTTAGCTCCTCTCTGATTCTGCGTAACAACAACGAACCGTTCCTGGATCGTATCGTAACCTGTGATGAGAAATGGATTCTGTATGATAACCGTCGCCGCTCTGCTCAGTGGCTGGATCGCGAAGAAGCTCCAAAACACTTCCCGAAACCGAATCTGCACCAGAAGAAAGTCATGGTAACCGTATGGTGGTCTGCCGCAGGTGTTATCCACTATTCCTTCCTGAACCCGGGCGAAACTATCACCAGCGAAAAATACTGCCAGCAGATTGACGAAATGCACCGTAAACTGCAGCGTCTGCAGCCAGCACTGGTGAATCGTAAAGGTCCGATCCTGCTGCATGATAACGCCCGTCCGCACGTTGCCCAACCGACCCTGCAGAAACTGAACGAACTGGGCTATGAAGTTCTGCCACACCCGCCGTACTCCCCGGATCTGTCCCCGACTGACTACCATTTCTTCAAGCATCTGGACAACTTCCTGCAGGGTAAACGTTTTCACAACCAACAGGACGCAGAAAACGCTTTCCAGGAGTTCGTCGAAAGCCGTTCCACTGACTTCTACGCGACCGGTATCAACAAGCTGATCAGCCGTTGGCAGAAATGCGTGGACTGTAACGGCAGCTACTTCGATTAAGGATCCTCTAGAGTCGACCTGCAGGCAAGCTTGGCACTGGCCGTCGTTTTACAACGTCGTGACTGGGAAAACCCTGGCGTTACCCAACTTAATCGCCTTGCAGCACATCCCCCTTTCGCCAGCTGGCGTAATAGCGAAGAGGCCCGCACCGATCGCCCTTCCCAACAGTTGCGCAGCCTGAATGGCGAATGGCAGCTTGGCTGTTTTGGCGGATGAGATAAGATTTTCAGCCTGATACAGATTAAATCAGAACGCAGAAGCGGTCTGATAAAACAGAATTTGCCTGGCGGCAGTAGCGCGGTGGTCCCACCTGACCCCATGCCGAACTCAGAAGTGAAACGCCGTAGCGCCGATGGTAGTGTGGGGTCTCCCCATGCGAGAGTAGGGAACTGCCAGGCATCAAATAAAACGAAAGGCTCAGTCGAAAGACTGGGCCTTTCGTTTTATCTGTTGTTTGTCGGTGAACGCTCTCCTGAGTAGGACAAATCCGCCGGGAGCGGATTTGAACGTTGCGAAGCAACGGCCCGGAGGGTGGCGGGCAGGACGCCCGCCATAAACTGCCAGGCATCAAATTAAGCAGAAGGCCATCCTGACGGATGGCCTTTTTGCGTTTCTACAAACTCTTTTTGTTTATTTTTCTAAATACATTCAAATATGTATCCGCTCATGAGACAATAACCCTGATAAATGCTTCAATAATATTGAAAAAGGAAGAGTATGAGTATTCAACATTTCCGTGTCGCCCTTATTCCCTTTTTTGCGGCATTTTGCCTTCCTGTTTTTGCTCACCCAGAAACGCTGGTGAAAGTAAAAGATGCTGAAGATCAGTTGGGTGCACGAGTGGGTTACATCGAACTGGATCTCAACAGCGGTAAGATCCTTGAGAGTTTTCGCCCCGAAGAACGTTCTCCAATGATGAGCACTTTTAAAGTTCTGCTATGTGGCGCGGTATTATCCCGTGTTGACGCCGGGCAAGAGCAACTCGGTCGCCGCATACACTATTCTCAGAATGACTTGGTTGAGTACTCACCAGTCACAGAAAAGCATCTTACGGATGGCATGACAGTAAGAGAATTATGCAGTGCTGCCATAACCATGAGTGATAACACTGCGGCCAACTTACTTCTGACAACGATCGGAGGACCGAAGGAGCTAACCGCTTTTTTGCACAACATGGGGGATCATGTAACTCGCCTTGATCGTTGGGAACCGGAGCTGAATGAAGCCATACCAAACGACGAGCGTGACACCACGATGCCTGTAGCAATGGCAACAACGTTGCGCAAACTATTAACTGGCGAACTACTTACTCTAGCTTCCCGGCAACAATTAATAGACTGGATGGAGGCGGATAAAGTTGCAGGACCACTTCTGCGCTCGGCCCTTCCGGCTGGCTGGTTTATTGCTGATAAATCTGGAGCCGGTGAGCGTGGGTCTCGCGGTATCATTGCAGCACTGGGGCCAGATGGTAAGCCCTCCCGTATCGTAGTTATCTACACGACGGGGAGTCAGGCAACTATGGATGAACGAAATAGACAGATCGCTGAGATAGGTGCCTCACTGATTAAGCATTGGTAACTGTCAGACCAAGTTTACTCATATATACTTTAGATTGATTTACCCCGGTTGATAATCAGAAAAGCCCCAAAAACAGGAAGATTGTATAAGCAAATATTTAAATTGTAAACGTTAATATTTTGTTAAAATTCGCGTTAAATTTTTGTTAAATCAGCTCATTTTTTAACCAATAGGCCGAAATCGGCAAAATCCCTTATAAATCAAAAGAATAGACCGAGATAGGGTTGAGTGTTGTTCCAGTTTGGAACAAGAGTCCACTATTAAAGAACGTGGACTCCAACGTCAAAGGGCGAAAAACCGTCTATCAGGGCGATGGCCCACTACGTGAACCATCACCCAAATCAAGTTTTTTGGGGTCGAGGTGCCGTAAAGCACTAAATCGGAACCCTAAAGGGAGCCCCCGATTTAGAGCTTGACGGGGAAAGCCGGCGAACGTGGCGAGAAAGGAAGGGAAGAAAGCGAAAGGAGCGGGCGCTAGGGCGCTGGCAAGTGTAGCGGTCACGCTGCGCGTAACCACCACACCCGCCGCGCTTAATGCGCCGCTACAGGGCGCGTAAAAGGATCTAGGTGAAGATCCTTTTTGATAATCTCATGACCAAAATCCCTTAACGTGAGTTTTCGTTCCACTGAGCGTCAGACCCCGTAGAAAAGATCAAAGGATCTTCTTGAGATCCTTTTTTTCTGCGCGTAATCTGCTGCTTGCAAACAAAAAAACCACCGCTACCAGCGGTGGTTTGTTTGCCGGATCAAGAGCTACCAACTCTTTTTCCGAAGGTAACTGGCTTCAGCAGAGCGCAGATACCAAATACTGTCCTTCTAGTGTAGCCGTAGTTAGGCCACCACTTCAAGAACTCTGTAGCACCGCCTACATACCTCGCTCTGCTAATCCTGTTACCAGTGGCTGCTGCCAGTGGCGATAAGTCGTGTCTTACCGGGTTGGACTCAAGACGATAGTTACCGGATAAGGCGCAGCGGTCGGGCTGAACGGGGGGTTCGTGCACACAGCCCAGCTTGGAGCGAACGACCTACACCGAACTGAGATACCTACAGCGTGAGCTATGAGAAAGCGCCACGCTTCCCGAAGGGAGAAAGGCGGACAGGTATCCGGTAAGCGGCAGGGTCGGAACAGGAGAGCGCACGAGGGAGCTTCCAGGGGGAAACGCCTGGTATCTTTATAGTCCTGTCGGGTTTCGCCACCTCTGACTTGAGCGTCGATTTTTGTGATGCTCGTCAGGGGGGCGGAGCCTATGGAAAAACGCCAGCAACGCGGCCTTTTTACGGTTCCTGGCCTTTTGCTGGCCTTTTGCTCACATGTTCTTTCCTGCGTTATCCCCTGATTCTGTGGATAACCGTATTACCGCCTTTGAGTGAGCTGATACCGCTCGCCGCAGCCGAACGACCGAGCGCAGCGAGTCAGTGAGCGAGGAAGCGGAAGAGCGCCTGATGCGGTATTTTCTCCTTACGCATCTGTGCGGTATTTCACACCGCATATATGGTGCACTCTCAGTACAATCTGCTCTGATGCCGCATAGTTAAGCCAGTATACACTCCGCTATCGCTACGTGACTGGGTCATGGCTGCGCCCCGACACCCGCCAACACCCGCTGACGCGCCCTGACGGGCTTGTCTGCTCCCGGCATCCGCTTACAGACAAGCTGTGACCGTCTCCGGGAGCTGCATGTGTCAGAGGTTTTCACCGTCATCACCGAAACGCGCGAGGCAGCTGCGGTAAAGCTCATCAGCGTGGTCGTGCAGCGATTCACAGATGTCTGCCTGTTCATCCGCGTCCAGCTCGTTGAGTTTCTCCAGAAGCGTTAATGTCTGGCTTCTGATAAAGCGGGCCATGTTAAGGGCGGTTTTTTCCTGTTTGGTCACTTGATGCCTCCGTGTAAGGGGGAATTTCTGTTCATGGGGGTAATGATACCGATGAAACGAGAGAGGATGCTCACGATACGGGTTACTGATGATGAACATGCCCGGTTACTGGAACGTTGTGAGGGTAAACAACTGGCGGTATGGATGCGGCGGGACCAGAGAAAAATCACTCAGGGTCAATGCCAGCGCTTCGTTAATACAGATGTAGGTGTTCCACAGGGTAGCCAGCAGCATCCTGCGATGCAGATCCGGAACATAATGGTGCAGGGCGCTGACTTCCGCGTTTCCAGACTTTACGAAACACGGAAACCGAAGACCATTCATGTTGTTGCTCAGGTCGCAGACGTTTTGCAGCAGCAGTCGCTTCACGTTCGCTCGCGTATCGGTGATTCATTCTGCTAACCAGTAAGGCAACCCCGCCAGCCTAGCCGGGTCCTCAACGACAGGAGCACGATCATGCGCACCCGTGGCCAGGACCCAACGCTGCCCGAAATT |
| pRC1881  (pMAL-c2x, Hsmar1 N143H, no MBP-tag) | CCGACACCATCGAATGGTGCAAAACCTTTCGCGGTATGGCATGATAGCGCCCGGAAGAGAGTCAATTCAGGGTGGTGAATGTGAAACCAGTAACGTTATACGATGTCGCAGAGTATGCCGGTGTCTCTTATCAGACCGTTTCCCGCGTGGTGAACCAGGCCAGCCACGTTTCTGCGAAAACGCGGGAAAAAGTGGAAGCGGCGATGGCGGAGCTGAATTACATTCCCAACCGCGTGGCACAACAACTGGCGGGCAAACAGTCGTTGCTGATTGGCGTTGCCACCTCCAGTCTGGCCCTGCACGCGCCGTCGCAAATTGTCGCGGCGATTAAATCTCGCGCCGATCAACTGGGTGCCAGCGTGGTGGTGTCGATGGTAGAACGAAGCGGCGTCGAAGCCTGTAAAGCGGCGGTGCACAATCTTCTCGCGCAACGCGTCAGTGGGCTGATCATTAACTATCCGCTGGATGACCAGGATGCCATTGCTGTGGAAGCTGCCTGCACTAATGTTCCGGCGTTATTTCTTGATGTCTCTGACCAGACACCCATCAACAGTATTATTTTCTCCCATGAAGACGGTACGCGACTGGGCGTGGAGCATCTGGTCGCATTGGGTCACCAGCAAATCGCGCTGTTAGCGGGCCCATTAAGTTCTGTCTCGGCGCGTCTGCGTCTGGCTGGCTGGCATAAATATCTCACTCGCAATCAAATTCAGCCGATAGCGGAACGGGAAGGCGACTGGAGTGCCATGTCCGGTTTTCAACAAACCATGCAAATGCTGAATGAGGGCATCGTTCCCACTGCGATGCTGGTTGCCAACGATCAGATGGCGCTGGGCGCAATGCGCGCCATTACCGAGTCCGGGCTGCGCGTTGGTGCGGATATCTCGGTAGTGGGATACGACGATACCGAAGACAGCTCATGTTATATCCCGCCGTTAACCACCATCAAACAGGATTTTCGCCTGCTGGGGCAAACCAGCGTGGACCGCTTGCTGCAACTCTCTCAGGGCCAGGCGGTGAAGGGCAATCAGCTGTTGCCCGTCTCACTGGTGAAAAGAAAAACCACCCTGGCGCCCAATACGCAAACCGCCTCTCCCCGCGCGTTGGCCGATTCATTAATGCAGCTGGCACGACAGGTTTCCCGACTGGAAAGCGGGCAGTGAGCGCAACGCAATTAATGTGAGTTAGCTCACTCATTAGGCACAATTCTCATGTTTGACAGCTTATCATCGACTGCACGGTGCACCAATGCTTCTGGCGTCAGGCAGCCATCGGAAGCTGTGGTATGGCTGTGCAGGTCGTAAATCACTGCATAATTCGTGTCGCTCAAGGCGCACTCCCGTTCTGGATAATGTTTTTTGCGCCGACATCATAACGGTTCTGGCAAATATTCTGAAATGAGCTGTTGACAATTAATCATCGGCTCGTATAATGTGTGGAATTGTGAGCGGATAACAATTTCACACAGGAAACAGCCAGTCCGTTTAGGTGTTTTCACGAGCACTTCACCAACAAGGACCATAGCATATGGAAATGATGCTCGATAAGAAACAGATTCGTGCGATCTTTCTCTTTGAGTTTAAAATGGGTCGCAAAGCGGCGGAGACGACGCGTAATATTAACAACGCGTTCGGTCCTGGCACCGCGAACGAGCGTACCGTGCAATGGTGGTTCAAAAAGTTTCGCAAAGGCGACGAATCTCTGGAGGACGAAGAGCGTTCTGGCCGCCCGTCCGAGGTTGACAACGACCAGCTGCGTGCAATCATCGAAGCTGATCCGCTGACTACCACCCGCGAAGTTGCTGAAGAACTGAATGTGGATCACTCTACTGTGGTTCGCCACCTGAAACAGATCGGTAAAGTAAAAAAACTGGACAAATGGGTTCCTCATGAACTGTCTGAAAACCAGAAAAACCGTCGTTTCGAAGTTAGCTCCTCTCTGATTCTGCGTAACcACAACGAACCGTTCCTGGATCGTATCGTAACCTGTGATGAGAAATGGATTCTGTATGATAACCGTCGCCGCTCTGCTCAGTGGCTGGATCGCGAAGAAGCTCCAAAACACTTCCCGAAACCGAATCTGCACCAGAAGAAAGTCATGGTAACCGTATGGTGGTCTGCCGCAGGTGTTATCCACTATTCCTTCCTGAACCCGGGCGAAACTATCACCAGCGAAAAATACTGCCAGCAGATTGACGAAATGCACCGTAAACTGCAGCGTCTGCAGCCAGCACTGGTGAATCGTAAAGGTCCGATCCTGCTGCATGATAACGCCCGTCCGCACGTTGCCCAACCGACCCTGCAGAAACTGAACGAACTGGGCTATGAAGTTCTGCCACACCCGCCGTACTCCCCGGATCTGTCCCCGACTGACTACCATTTCTTCAAGCATCTGGACAACTTCCTGCAGGGTAAACGTTTTCACAACCAACAGGACGCAGAAAACGCTTTCCAGGAGTTCGTCGAAAGCCGTTCCACTGACTTCTACGCGACCGGTATCAACAAGCTGATCAGCCGTTGGCAGAAATGCGTGGACTGTAACGGCAGCTACTTCGATTAAGGATCCTCTAGAGTCGACCTGCAGGCAAGCTTGGCACTGGCCGTCGTTTTACAACGTCGTGACTGGGAAAACCCTGGCGTTACCCAACTTAATCGCCTTGCAGCACATCCCCCTTTCGCCAGCTGGCGTAATAGCGAAGAGGCCCGCACCGATCGCCCTTCCCAACAGTTGCGCAGCCTGAATGGCGAATGGCAGCTTGGCTGTTTTGGCGGATGAGATAAGATTTTCAGCCTGATACAGATTAAATCAGAACGCAGAAGCGGTCTGATAAAACAGAATTTGCCTGGCGGCAGTAGCGCGGTGGTCCCACCTGACCCCATGCCGAACTCAGAAGTGAAACGCCGTAGCGCCGATGGTAGTGTGGGGTCTCCCCATGCGAGAGTAGGGAACTGCCAGGCATCAAATAAAACGAAAGGCTCAGTCGAAAGACTGGGCCTTTCGTTTTATCTGTTGTTTGTCGGTGAACGCTCTCCTGAGTAGGACAAATCCGCCGGGAGCGGATTTGAACGTTGCGAAGCAACGGCCCGGAGGGTGGCGGGCAGGACGCCCGCCATAAACTGCCAGGCATCAAATTAAGCAGAAGGCCATCCTGACGGATGGCCTTTTTGCGTTTCTACAAACTCTTTTTGTTTATTTTTCTAAATACATTCAAATATGTATCCGCTCATGAGACAATAACCCTGATAAATGCTTCAATAATATTGAAAAAGGAAGAGTATGAGTATTCAACATTTCCGTGTCGCCCTTATTCCCTTTTTTGCGGCATTTTGCCTTCCTGTTTTTGCTCACCCAGAAACGCTGGTGAAAGTAAAAGATGCTGAAGATCAGTTGGGTGCACGAGTGGGTTACATCGAACTGGATCTCAACAGCGGTAAGATCCTTGAGAGTTTTCGCCCCGAAGAACGTTCTCCAATGATGAGCACTTTTAAAGTTCTGCTATGTGGCGCGGTATTATCCCGTGTTGACGCCGGGCAAGAGCAACTCGGTCGCCGCATACACTATTCTCAGAATGACTTGGTTGAGTACTCACCAGTCACAGAAAAGCATCTTACGGATGGCATGACAGTAAGAGAATTATGCAGTGCTGCCATAACCATGAGTGATAACACTGCGGCCAACTTACTTCTGACAACGATCGGAGGACCGAAGGAGCTAACCGCTTTTTTGCACAACATGGGGGATCATGTAACTCGCCTTGATCGTTGGGAACCGGAGCTGAATGAAGCCATACCAAACGACGAGCGTGACACCACGATGCCTGTAGCAATGGCAACAACGTTGCGCAAACTATTAACTGGCGAACTACTTACTCTAGCTTCCCGGCAACAATTAATAGACTGGATGGAGGCGGATAAAGTTGCAGGACCACTTCTGCGCTCGGCCCTTCCGGCTGGCTGGTTTATTGCTGATAAATCTGGAGCCGGTGAGCGTGGGTCTCGCGGTATCATTGCAGCACTGGGGCCAGATGGTAAGCCCTCCCGTATCGTAGTTATCTACACGACGGGGAGTCAGGCAACTATGGATGAACGAAATAGACAGATCGCTGAGATAGGTGCCTCACTGATTAAGCATTGGTAACTGTCAGACCAAGTTTACTCATATATACTTTAGATTGATTTACCCCGGTTGATAATCAGAAAAGCCCCAAAAACAGGAAGATTGTATAAGCAAATATTTAAATTGTAAACGTTAATATTTTGTTAAAATTCGCGTTAAATTTTTGTTAAATCAGCTCATTTTTTAACCAATAGGCCGAAATCGGCAAAATCCCTTATAAATCAAAAGAATAGACCGAGATAGGGTTGAGTGTTGTTCCAGTTTGGAACAAGAGTCCACTATTAAAGAACGTGGACTCCAACGTCAAAGGGCGAAAAACCGTCTATCAGGGCGATGGCCCACTACGTGAACCATCACCCAAATCAAGTTTTTTGGGGTCGAGGTGCCGTAAAGCACTAAATCGGAACCCTAAAGGGAGCCCCCGATTTAGAGCTTGACGGGGAAAGCCGGCGAACGTGGCGAGAAAGGAAGGGAAGAAAGCGAAAGGAGCGGGCGCTAGGGCGCTGGCAAGTGTAGCGGTCACGCTGCGCGTAACCACCACACCCGCCGCGCTTAATGCGCCGCTACAGGGCGCGTAAAAGGATCTAGGTGAAGATCCTTTTTGATAATCTCATGACCAAAATCCCTTAACGTGAGTTTTCGTTCCACTGAGCGTCAGACCCCGTAGAAAAGATCAAAGGATCTTCTTGAGATCCTTTTTTTCTGCGCGTAATCTGCTGCTTGCAAACAAAAAAACCACCGCTACCAGCGGTGGTTTGTTTGCCGGATCAAGAGCTACCAACTCTTTTTCCGAAGGTAACTGGCTTCAGCAGAGCGCAGATACCAAATACTGTCCTTCTAGTGTAGCCGTAGTTAGGCCACCACTTCAAGAACTCTGTAGCACCGCCTACATACCTCGCTCTGCTAATCCTGTTACCAGTGGCTGCTGCCAGTGGCGATAAGTCGTGTCTTACCGGGTTGGACTCAAGACGATAGTTACCGGATAAGGCGCAGCGGTCGGGCTGAACGGGGGGTTCGTGCACACAGCCCAGCTTGGAGCGAACGACCTACACCGAACTGAGATACCTACAGCGTGAGCTATGAGAAAGCGCCACGCTTCCCGAAGGGAGAAAGGCGGACAGGTATCCGGTAAGCGGCAGGGTCGGAACAGGAGAGCGCACGAGGGAGCTTCCAGGGGGAAACGCCTGGTATCTTTATAGTCCTGTCGGGTTTCGCCACCTCTGACTTGAGCGTCGATTTTTGTGATGCTCGTCAGGGGGGCGGAGCCTATGGAAAAACGCCAGCAACGCGGCCTTTTTACGGTTCCTGGCCTTTTGCTGGCCTTTTGCTCACATGTTCTTTCCTGCGTTATCCCCTGATTCTGTGGATAACCGTATTACCGCCTTTGAGTGAGCTGATACCGCTCGCCGCAGCCGAACGACCGAGCGCAGCGAGTCAGTGAGCGAGGAAGCGGAAGAGCGCCTGATGCGGTATTTTCTCCTTACGCATCTGTGCGGTATTTCACACCGCATATATGGTGCACTCTCAGTACAATCTGCTCTGATGCCGCATAGTTAAGCCAGTATACACTCCGCTATCGCTACGTGACTGGGTCATGGCTGCGCCCCGACACCCGCCAACACCCGCTGACGCGCCCTGACGGGCTTGTCTGCTCCCGGCATCCGCTTACAGACAAGCTGTGACCGTCTCCGGGAGCTGCATGTGTCAGAGGTTTTCACCGTCATCACCGAAACGCGCGAGGCAGCTGCGGTAAAGCTCATCAGCGTGGTCGTGCAGCGATTCACAGATGTCTGCCTGTTCATCCGCGTCCAGCTCGTTGAGTTTCTCCAGAAGCGTTAATGTCTGGCTTCTGATAAAGCGGGCCATGTTAAGGGCGGTTTTTTCCTGTTTGGTCACTTGATGCCTCCGTGTAAGGGGGAATTTCTGTTCATGGGGGTAATGATACCGATGAAACGAGAGAGGATGCTCACGATACGGGTTACTGATGATGAACATGCCCGGTTACTGGAACGTTGTGAGGGTAAACAACTGGCGGTATGGATGCGGCGGGACCAGAGAAAAATCACTCAGGGTCAATGCCAGCGCTTCGTTAATACAGATGTAGGTGTTCCACAGGGTAGCCAGCAGCATCCTGCGATGCAGATCCGGAACATAATGGTGCAGGGCGCTGACTTCCGCGTTTCCAGACTTTACGAAACACGGAAACCGAAGACCATTCATGTTGTTGCTCAGGTCGCAGACGTTTTGCAGCAGCAGTCGCTTCACGTTCGCTCGCGTATCGGTGATTCATTCTGCTAACCAGTAAGGCAACCCCGCCAGCCTAGCCGGGTCCTCAACGACAGGAGCACGATCATGCGCACCCGTGGCCAGGACCCAACGCTGCCCGAAATT |
| pRC1882  (pMAL-c2x, Hsmar1 R173Q, no MBP-tag) | CCGACACCATCGAATGGTGCAAAACCTTTCGCGGTATGGCATGATAGCGCCCGGAAGAGAGTCAATTCAGGGTGGTGAATGTGAAACCAGTAACGTTATACGATGTCGCAGAGTATGCCGGTGTCTCTTATCAGACCGTTTCCCGCGTGGTGAACCAGGCCAGCCACGTTTCTGCGAAAACGCGGGAAAAAGTGGAAGCGGCGATGGCGGAGCTGAATTACATTCCCAACCGCGTGGCACAACAACTGGCGGGCAAACAGTCGTTGCTGATTGGCGTTGCCACCTCCAGTCTGGCCCTGCACGCGCCGTCGCAAATTGTCGCGGCGATTAAATCTCGCGCCGATCAACTGGGTGCCAGCGTGGTGGTGTCGATGGTAGAACGAAGCGGCGTCGAAGCCTGTAAAGCGGCGGTGCACAATCTTCTCGCGCAACGCGTCAGTGGGCTGATCATTAACTATCCGCTGGATGACCAGGATGCCATTGCTGTGGAAGCTGCCTGCACTAATGTTCCGGCGTTATTTCTTGATGTCTCTGACCAGACACCCATCAACAGTATTATTTTCTCCCATGAAGACGGTACGCGACTGGGCGTGGAGCATCTGGTCGCATTGGGTCACCAGCAAATCGCGCTGTTAGCGGGCCCATTAAGTTCTGTCTCGGCGCGTCTGCGTCTGGCTGGCTGGCATAAATATCTCACTCGCAATCAAATTCAGCCGATAGCGGAACGGGAAGGCGACTGGAGTGCCATGTCCGGTTTTCAACAAACCATGCAAATGCTGAATGAGGGCATCGTTCCCACTGCGATGCTGGTTGCCAACGATCAGATGGCGCTGGGCGCAATGCGCGCCATTACCGAGTCCGGGCTGCGCGTTGGTGCGGATATCTCGGTAGTGGGATACGACGATACCGAAGACAGCTCATGTTATATCCCGCCGTTAACCACCATCAAACAGGATTTTCGCCTGCTGGGGCAAACCAGCGTGGACCGCTTGCTGCAACTCTCTCAGGGCCAGGCGGTGAAGGGCAATCAGCTGTTGCCCGTCTCACTGGTGAAAAGAAAAACCACCCTGGCGCCCAATACGCAAACCGCCTCTCCCCGCGCGTTGGCCGATTCATTAATGCAGCTGGCACGACAGGTTTCCCGACTGGAAAGCGGGCAGTGAGCGCAACGCAATTAATGTGAGTTAGCTCACTCATTAGGCACAATTCTCATGTTTGACAGCTTATCATCGACTGCACGGTGCACCAATGCTTCTGGCGTCAGGCAGCCATCGGAAGCTGTGGTATGGCTGTGCAGGTCGTAAATCACTGCATAATTCGTGTCGCTCAAGGCGCACTCCCGTTCTGGATAATGTTTTTTGCGCCGACATCATAACGGTTCTGGCAAATATTCTGAAATGAGCTGTTGACAATTAATCATCGGCTCGTATAATGTGTGGAATTGTGAGCGGATAACAATTTCACACAGGAAACAGCCAGTCCGTTTAGGTGTTTTCACGAGCACTTCACCAACAAGGACCATAGCATATGGAAATGATGCTCGATAAGAAACAGATTCGTGCGATCTTTCTCTTTGAGTTTAAAATGGGTCGCAAAGCGGCGGAGACGACGCGTAATATTAACAACGCGTTCGGTCCTGGCACCGCGAACGAGCGTACCGTGCAATGGTGGTTCAAAAAGTTTCGCAAAGGCGACGAATCTCTGGAGGACGAAGAGCGTTCTGGCCGCCCGTCCGAGGTTGACAACGACCAGCTGCGTGCAATCATCGAAGCTGATCCGCTGACTACCACCCGCGAAGTTGCTGAAGAACTGAATGTGGATCACTCTACTGTGGTTCGCCACCTGAAACAGATCGGTAAAGTAAAAAAACTGGACAAATGGGTTCCTCATGAACTGTCTGAAAACCAGAAAAACCGTCGTTTCGAAGTTAGCTCCTCTCTGATTCTGCGTAACAACAACGAACCGTTCCTGGATCGTATCGTAACCTGTGATGAGAAATGGATTCTGTATGATAACCGTCGCCGCTCTGCTCAGTGGCTGGATCagGAAGAAGCTCCAAAACACTTCCCGAAACCGAATCTGCACCAGAAGAAAGTCATGGTAACCGTATGGTGGTCTGCCGCAGGTGTTATCCACTATTCCTTCCTGAACCCGGGCGAAACTATCACCAGCGAAAAATACTGCCAGCAGATTGACGAAATGCACCGTAAACTGCAGCGTCTGCAGCCAGCACTGGTGAATCGTAAAGGTCCGATCCTGCTGCATGATAACGCCCGTCCGCACGTTGCCCAACCGACCCTGCAGAAACTGAACGAACTGGGCTATGAAGTTCTGCCACACCCGCCGTACTCCCCGGATCTGTCCCCGACTGACTACCATTTCTTCAAGCATCTGGACAACTTCCTGCAGGGTAAACGTTTTCACAACCAACAGGACGCAGAAAACGCTTTCCAGGAGTTCGTCGAAAGCCGTTCCACTGACTTCTACGCGACCGGTATCAACAAGCTGATCAGCCGTTGGCAGAAATGCGTGGACTGTAACGGCAGCTACTTCGATTAAGGATCCTCTAGAGTCGACCTGCAGGCAAGCTTGGCACTGGCCGTCGTTTTACAACGTCGTGACTGGGAAAACCCTGGCGTTACCCAACTTAATCGCCTTGCAGCACATCCCCCTTTCGCCAGCTGGCGTAATAGCGAAGAGGCCCGCACCGATCGCCCTTCCCAACAGTTGCGCAGCCTGAATGGCGAATGGCAGCTTGGCTGTTTTGGCGGATGAGATAAGATTTTCAGCCTGATACAGATTAAATCAGAACGCAGAAGCGGTCTGATAAAACAGAATTTGCCTGGCGGCAGTAGCGCGGTGGTCCCACCTGACCCCATGCCGAACTCAGAAGTGAAACGCCGTAGCGCCGATGGTAGTGTGGGGTCTCCCCATGCGAGAGTAGGGAACTGCCAGGCATCAAATAAAACGAAAGGCTCAGTCGAAAGACTGGGCCTTTCGTTTTATCTGTTGTTTGTCGGTGAACGCTCTCCTGAGTAGGACAAATCCGCCGGGAGCGGATTTGAACGTTGCGAAGCAACGGCCCGGAGGGTGGCGGGCAGGACGCCCGCCATAAACTGCCAGGCATCAAATTAAGCAGAAGGCCATCCTGACGGATGGCCTTTTTGCGTTTCTACAAACTCTTTTTGTTTATTTTTCTAAATACATTCAAATATGTATCCGCTCATGAGACAATAACCCTGATAAATGCTTCAATAATATTGAAAAAGGAAGAGTATGAGTATTCAACATTTCCGTGTCGCCCTTATTCCCTTTTTTGCGGCATTTTGCCTTCCTGTTTTTGCTCACCCAGAAACGCTGGTGAAAGTAAAAGATGCTGAAGATCAGTTGGGTGCACGAGTGGGTTACATCGAACTGGATCTCAACAGCGGTAAGATCCTTGAGAGTTTTCGCCCCGAAGAACGTTCTCCAATGATGAGCACTTTTAAAGTTCTGCTATGTGGCGCGGTATTATCCCGTGTTGACGCCGGGCAAGAGCAACTCGGTCGCCGCATACACTATTCTCAGAATGACTTGGTTGAGTACTCACCAGTCACAGAAAAGCATCTTACGGATGGCATGACAGTAAGAGAATTATGCAGTGCTGCCATAACCATGAGTGATAACACTGCGGCCAACTTACTTCTGACAACGATCGGAGGACCGAAGGAGCTAACCGCTTTTTTGCACAACATGGGGGATCATGTAACTCGCCTTGATCGTTGGGAACCGGAGCTGAATGAAGCCATACCAAACGACGAGCGTGACACCACGATGCCTGTAGCAATGGCAACAACGTTGCGCAAACTATTAACTGGCGAACTACTTACTCTAGCTTCCCGGCAACAATTAATAGACTGGATGGAGGCGGATAAAGTTGCAGGACCACTTCTGCGCTCGGCCCTTCCGGCTGGCTGGTTTATTGCTGATAAATCTGGAGCCGGTGAGCGTGGGTCTCGCGGTATCATTGCAGCACTGGGGCCAGATGGTAAGCCCTCCCGTATCGTAGTTATCTACACGACGGGGAGTCAGGCAACTATGGATGAACGAAATAGACAGATCGCTGAGATAGGTGCCTCACTGATTAAGCATTGGTAACTGTCAGACCAAGTTTACTCATATATACTTTAGATTGATTTACCCCGGTTGATAATCAGAAAAGCCCCAAAAACAGGAAGATTGTATAAGCAAATATTTAAATTGTAAACGTTAATATTTTGTTAAAATTCGCGTTAAATTTTTGTTAAATCAGCTCATTTTTTAACCAATAGGCCGAAATCGGCAAAATCCCTTATAAATCAAAAGAATAGACCGAGATAGGGTTGAGTGTTGTTCCAGTTTGGAACAAGAGTCCACTATTAAAGAACGTGGACTCCAACGTCAAAGGGCGAAAAACCGTCTATCAGGGCGATGGCCCACTACGTGAACCATCACCCAAATCAAGTTTTTTGGGGTCGAGGTGCCGTAAAGCACTAAATCGGAACCCTAAAGGGAGCCCCCGATTTAGAGCTTGACGGGGAAAGCCGGCGAACGTGGCGAGAAAGGAAGGGAAGAAAGCGAAAGGAGCGGGCGCTAGGGCGCTGGCAAGTGTAGCGGTCACGCTGCGCGTAACCACCACACCCGCCGCGCTTAATGCGCCGCTACAGGGCGCGTAAAAGGATCTAGGTGAAGATCCTTTTTGATAATCTCATGACCAAAATCCCTTAACGTGAGTTTTCGTTCCACTGAGCGTCAGACCCCGTAGAAAAGATCAAAGGATCTTCTTGAGATCCTTTTTTTCTGCGCGTAATCTGCTGCTTGCAAACAAAAAAACCACCGCTACCAGCGGTGGTTTGTTTGCCGGATCAAGAGCTACCAACTCTTTTTCCGAAGGTAACTGGCTTCAGCAGAGCGCAGATACCAAATACTGTCCTTCTAGTGTAGCCGTAGTTAGGCCACCACTTCAAGAACTCTGTAGCACCGCCTACATACCTCGCTCTGCTAATCCTGTTACCAGTGGCTGCTGCCAGTGGCGATAAGTCGTGTCTTACCGGGTTGGACTCAAGACGATAGTTACCGGATAAGGCGCAGCGGTCGGGCTGAACGGGGGGTTCGTGCACACAGCCCAGCTTGGAGCGAACGACCTACACCGAACTGAGATACCTACAGCGTGAGCTATGAGAAAGCGCCACGCTTCCCGAAGGGAGAAAGGCGGACAGGTATCCGGTAAGCGGCAGGGTCGGAACAGGAGAGCGCACGAGGGAGCTTCCAGGGGGAAACGCCTGGTATCTTTATAGTCCTGTCGGGTTTCGCCACCTCTGACTTGAGCGTCGATTTTTGTGATGCTCGTCAGGGGGGCGGAGCCTATGGAAAAACGCCAGCAACGCGGCCTTTTTACGGTTCCTGGCCTTTTGCTGGCCTTTTGCTCACATGTTCTTTCCTGCGTTATCCCCTGATTCTGTGGATAACCGTATTACCGCCTTTGAGTGAGCTGATACCGCTCGCCGCAGCCGAACGACCGAGCGCAGCGAGTCAGTGAGCGAGGAAGCGGAAGAGCGCCTGATGCGGTATTTTCTCCTTACGCATCTGTGCGGTATTTCACACCGCATATATGGTGCACTCTCAGTACAATCTGCTCTGATGCCGCATAGTTAAGCCAGTATACACTCCGCTATCGCTACGTGACTGGGTCATGGCTGCGCCCCGACACCCGCCAACACCCGCTGACGCGCCCTGACGGGCTTGTCTGCTCCCGGCATCCGCTTACAGACAAGCTGTGACCGTCTCCGGGAGCTGCATGTGTCAGAGGTTTTCACCGTCATCACCGAAACGCGCGAGGCAGCTGCGGTAAAGCTCATCAGCGTGGTCGTGCAGCGATTCACAGATGTCTGCCTGTTCATCCGCGTCCAGCTCGTTGAGTTTCTCCAGAAGCGTTAATGTCTGGCTTCTGATAAAGCGGGCCATGTTAAGGGCGGTTTTTTCCTGTTTGGTCACTTGATGCCTCCGTGTAAGGGGGAATTTCTGTTCATGGGGGTAATGATACCGATGAAACGAGAGAGGATGCTCACGATACGGGTTACTGATGATGAACATGCCCGGTTACTGGAACGTTGTGAGGGTAAACAACTGGCGGTATGGATGCGGCGGGACCAGAGAAAAATCACTCAGGGTCAATGCCAGCGCTTCGTTAATACAGATGTAGGTGTTCCACAGGGTAGCCAGCAGCATCCTGCGATGCAGATCCGGAACATAATGGTGCAGGGCGCTGACTTCCGCGTTTCCAGACTTTACGAAACACGGAAACCGAAGACCATTCATGTTGTTGCTCAGGTCGCAGACGTTTTGCAGCAGCAGTCGCTTCACGTTCGCTCGCGTATCGGTGATTCATTCTGCTAACCAGTAAGGCAACCCCGCCAGCCTAGCCGGGTCCTCAACGACAGGAGCACGATCATGCGCACCCGTGGCCAGGACCCAACGCTGCCCGAAATT |
| pRC1883  (pMAL-c2x, Hsmar1 N184I, no MBP-tag) | CCGACACCATCGAATGGTGCAAAACCTTTCGCGGTATGGCATGATAGCGCCCGGAAGAGAGTCAATTCAGGGTGGTGAATGTGAAACCAGTAACGTTATACGATGTCGCAGAGTATGCCGGTGTCTCTTATCAGACCGTTTCCCGCGTGGTGAACCAGGCCAGCCACGTTTCTGCGAAAACGCGGGAAAAAGTGGAAGCGGCGATGGCGGAGCTGAATTACATTCCCAACCGCGTGGCACAACAACTGGCGGGCAAACAGTCGTTGCTGATTGGCGTTGCCACCTCCAGTCTGGCCCTGCACGCGCCGTCGCAAATTGTCGCGGCGATTAAATCTCGCGCCGATCAACTGGGTGCCAGCGTGGTGGTGTCGATGGTAGAACGAAGCGGCGTCGAAGCCTGTAAAGCGGCGGTGCACAATCTTCTCGCGCAACGCGTCAGTGGGCTGATCATTAACTATCCGCTGGATGACCAGGATGCCATTGCTGTGGAAGCTGCCTGCACTAATGTTCCGGCGTTATTTCTTGATGTCTCTGACCAGACACCCATCAACAGTATTATTTTCTCCCATGAAGACGGTACGCGACTGGGCGTGGAGCATCTGGTCGCATTGGGTCACCAGCAAATCGCGCTGTTAGCGGGCCCATTAAGTTCTGTCTCGGCGCGTCTGCGTCTGGCTGGCTGGCATAAATATCTCACTCGCAATCAAATTCAGCCGATAGCGGAACGGGAAGGCGACTGGAGTGCCATGTCCGGTTTTCAACAAACCATGCAAATGCTGAATGAGGGCATCGTTCCCACTGCGATGCTGGTTGCCAACGATCAGATGGCGCTGGGCGCAATGCGCGCCATTACCGAGTCCGGGCTGCGCGTTGGTGCGGATATCTCGGTAGTGGGATACGACGATACCGAAGACAGCTCATGTTATATCCCGCCGTTAACCACCATCAAACAGGATTTTCGCCTGCTGGGGCAAACCAGCGTGGACCGCTTGCTGCAACTCTCTCAGGGCCAGGCGGTGAAGGGCAATCAGCTGTTGCCCGTCTCACTGGTGAAAAGAAAAACCACCCTGGCGCCCAATACGCAAACCGCCTCTCCCCGCGCGTTGGCCGATTCATTAATGCAGCTGGCACGACAGGTTTCCCGACTGGAAAGCGGGCAGTGAGCGCAACGCAATTAATGTGAGTTAGCTCACTCATTAGGCACAATTCTCATGTTTGACAGCTTATCATCGACTGCACGGTGCACCAATGCTTCTGGCGTCAGGCAGCCATCGGAAGCTGTGGTATGGCTGTGCAGGTCGTAAATCACTGCATAATTCGTGTCGCTCAAGGCGCACTCCCGTTCTGGATAATGTTTTTTGCGCCGACATCATAACGGTTCTGGCAAATATTCTGAAATGAGCTGTTGACAATTAATCATCGGCTCGTATAATGTGTGGAATTGTGAGCGGATAACAATTTCACACAGGAAACAGCCAGTCCGTTTAGGTGTTTTCACGAGCACTTCACCAACAAGGACCATAGCATATGGAAATGATGCTCGATAAGAAACAGATTCGTGCGATCTTTCTCTTTGAGTTTAAAATGGGTCGCAAAGCGGCGGAGACGACGCGTAATATTAACAACGCGTTCGGTCCTGGCACCGCGAACGAGCGTACCGTGCAATGGTGGTTCAAAAAGTTTCGCAAAGGCGACGAATCTCTGGAGGACGAAGAGCGTTCTGGCCGCCCGTCCGAGGTTGACAACGACCAGCTGCGTGCAATCATCGAAGCTGATCCGCTGACTACCACCCGCGAAGTTGCTGAAGAACTGAATGTGGATCACTCTACTGTGGTTCGCCACCTGAAACAGATCGGTAAAGTAAAAAAACTGGACAAATGGGTTCCTCATGAACTGTCTGAAAACCAGAAAAACCGTCGTTTCGAAGTTAGCTCCTCTCTGATTCTGCGTAACAACAACGAACCGTTCCTGGATCGTATCGTAACCTGTGATGAGAAATGGATTCTGTATGATAACCGTCGCCGCTCTGCTCAGTGGCTGGATCGCGAAGAAGCTCCAAAACACTTCCCGAAACCGAtTCTGCACCAGAAGAAAGTCATGGTAACCGTATGGTGGTCTGCCGCAGGTGTTATCCACTATTCCTTCCTGAACCCGGGCGAAACTATCACCAGCGAAAAATACTGCCAGCAGATTGACGAAATGCACCGTAAACTGCAGCGTCTGCAGCCAGCACTGGTGAATCGTAAAGGTCCGATCCTGCTGCATGATAACGCCCGTCCGCACGTTGCCCAACCGACCCTGCAGAAACTGAACGAACTGGGCTATGAAGTTCTGCCACACCCGCCGTACTCCCCGGATCTGTCCCCGACTGACTACCATTTCTTCAAGCATCTGGACAACTTCCTGCAGGGTAAACGTTTTCACAACCAACAGGACGCAGAAAACGCTTTCCAGGAGTTCGTCGAAAGCCGTTCCACTGACTTCTACGCGACCGGTATCAACAAGCTGATCAGCCGTTGGCAGAAATGCGTGGACTGTAACGGCAGCTACTTCGATTAAGGATCCTCTAGAGTCGACCTGCAGGCAAGCTTGGCACTGGCCGTCGTTTTACAACGTCGTGACTGGGAAAACCCTGGCGTTACCCAACTTAATCGCCTTGCAGCACATCCCCCTTTCGCCAGCTGGCGTAATAGCGAAGAGGCCCGCACCGATCGCCCTTCCCAACAGTTGCGCAGCCTGAATGGCGAATGGCAGCTTGGCTGTTTTGGCGGATGAGATAAGATTTTCAGCCTGATACAGATTAAATCAGAACGCAGAAGCGGTCTGATAAAACAGAATTTGCCTGGCGGCAGTAGCGCGGTGGTCCCACCTGACCCCATGCCGAACTCAGAAGTGAAACGCCGTAGCGCCGATGGTAGTGTGGGGTCTCCCCATGCGAGAGTAGGGAACTGCCAGGCATCAAATAAAACGAAAGGCTCAGTCGAAAGACTGGGCCTTTCGTTTTATCTGTTGTTTGTCGGTGAACGCTCTCCTGAGTAGGACAAATCCGCCGGGAGCGGATTTGAACGTTGCGAAGCAACGGCCCGGAGGGTGGCGGGCAGGACGCCCGCCATAAACTGCCAGGCATCAAATTAAGCAGAAGGCCATCCTGACGGATGGCCTTTTTGCGTTTCTACAAACTCTTTTTGTTTATTTTTCTAAATACATTCAAATATGTATCCGCTCATGAGACAATAACCCTGATAAATGCTTCAATAATATTGAAAAAGGAAGAGTATGAGTATTCAACATTTCCGTGTCGCCCTTATTCCCTTTTTTGCGGCATTTTGCCTTCCTGTTTTTGCTCACCCAGAAACGCTGGTGAAAGTAAAAGATGCTGAAGATCAGTTGGGTGCACGAGTGGGTTACATCGAACTGGATCTCAACAGCGGTAAGATCCTTGAGAGTTTTCGCCCCGAAGAACGTTCTCCAATGATGAGCACTTTTAAAGTTCTGCTATGTGGCGCGGTATTATCCCGTGTTGACGCCGGGCAAGAGCAACTCGGTCGCCGCATACACTATTCTCAGAATGACTTGGTTGAGTACTCACCAGTCACAGAAAAGCATCTTACGGATGGCATGACAGTAAGAGAATTATGCAGTGCTGCCATAACCATGAGTGATAACACTGCGGCCAACTTACTTCTGACAACGATCGGAGGACCGAAGGAGCTAACCGCTTTTTTGCACAACATGGGGGATCATGTAACTCGCCTTGATCGTTGGGAACCGGAGCTGAATGAAGCCATACCAAACGACGAGCGTGACACCACGATGCCTGTAGCAATGGCAACAACGTTGCGCAAACTATTAACTGGCGAACTACTTACTCTAGCTTCCCGGCAACAATTAATAGACTGGATGGAGGCGGATAAAGTTGCAGGACCACTTCTGCGCTCGGCCCTTCCGGCTGGCTGGTTTATTGCTGATAAATCTGGAGCCGGTGAGCGTGGGTCTCGCGGTATCATTGCAGCACTGGGGCCAGATGGTAAGCCCTCCCGTATCGTAGTTATCTACACGACGGGGAGTCAGGCAACTATGGATGAACGAAATAGACAGATCGCTGAGATAGGTGCCTCACTGATTAAGCATTGGTAACTGTCAGACCAAGTTTACTCATATATACTTTAGATTGATTTACCCCGGTTGATAATCAGAAAAGCCCCAAAAACAGGAAGATTGTATAAGCAAATATTTAAATTGTAAACGTTAATATTTTGTTAAAATTCGCGTTAAATTTTTGTTAAATCAGCTCATTTTTTAACCAATAGGCCGAAATCGGCAAAATCCCTTATAAATCAAAAGAATAGACCGAGATAGGGTTGAGTGTTGTTCCAGTTTGGAACAAGAGTCCACTATTAAAGAACGTGGACTCCAACGTCAAAGGGCGAAAAACCGTCTATCAGGGCGATGGCCCACTACGTGAACCATCACCCAAATCAAGTTTTTTGGGGTCGAGGTGCCGTAAAGCACTAAATCGGAACCCTAAAGGGAGCCCCCGATTTAGAGCTTGACGGGGAAAGCCGGCGAACGTGGCGAGAAAGGAAGGGAAGAAAGCGAAAGGAGCGGGCGCTAGGGCGCTGGCAAGTGTAGCGGTCACGCTGCGCGTAACCACCACACCCGCCGCGCTTAATGCGCCGCTACAGGGCGCGTAAAAGGATCTAGGTGAAGATCCTTTTTGATAATCTCATGACCAAAATCCCTTAACGTGAGTTTTCGTTCCACTGAGCGTCAGACCCCGTAGAAAAGATCAAAGGATCTTCTTGAGATCCTTTTTTTCTGCGCGTAATCTGCTGCTTGCAAACAAAAAAACCACCGCTACCAGCGGTGGTTTGTTTGCCGGATCAAGAGCTACCAACTCTTTTTCCGAAGGTAACTGGCTTCAGCAGAGCGCAGATACCAAATACTGTCCTTCTAGTGTAGCCGTAGTTAGGCCACCACTTCAAGAACTCTGTAGCACCGCCTACATACCTCGCTCTGCTAATCCTGTTACCAGTGGCTGCTGCCAGTGGCGATAAGTCGTGTCTTACCGGGTTGGACTCAAGACGATAGTTACCGGATAAGGCGCAGCGGTCGGGCTGAACGGGGGGTTCGTGCACACAGCCCAGCTTGGAGCGAACGACCTACACCGAACTGAGATACCTACAGCGTGAGCTATGAGAAAGCGCCACGCTTCCCGAAGGGAGAAAGGCGGACAGGTATCCGGTAAGCGGCAGGGTCGGAACAGGAGAGCGCACGAGGGAGCTTCCAGGGGGAAACGCCTGGTATCTTTATAGTCCTGTCGGGTTTCGCCACCTCTGACTTGAGCGTCGATTTTTGTGATGCTCGTCAGGGGGGCGGAGCCTATGGAAAAACGCCAGCAACGCGGCCTTTTTACGGTTCCTGGCCTTTTGCTGGCCTTTTGCTCACATGTTCTTTCCTGCGTTATCCCCTGATTCTGTGGATAACCGTATTACCGCCTTTGAGTGAGCTGATACCGCTCGCCGCAGCCGAACGACCGAGCGCAGCGAGTCAGTGAGCGAGGAAGCGGAAGAGCGCCTGATGCGGTATTTTCTCCTTACGCATCTGTGCGGTATTTCACACCGCATATATGGTGCACTCTCAGTACAATCTGCTCTGATGCCGCATAGTTAAGCCAGTATACACTCCGCTATCGCTACGTGACTGGGTCATGGCTGCGCCCCGACACCCGCCAACACCCGCTGACGCGCCCTGACGGGCTTGTCTGCTCCCGGCATCCGCTTACAGACAAGCTGTGACCGTCTCCGGGAGCTGCATGTGTCAGAGGTTTTCACCGTCATCACCGAAACGCGCGAGGCAGCTGCGGTAAAGCTCATCAGCGTGGTCGTGCAGCGATTCACAGATGTCTGCCTGTTCATCCGCGTCCAGCTCGTTGAGTTTCTCCAGAAGCGTTAATGTCTGGCTTCTGATAAAGCGGGCCATGTTAAGGGCGGTTTTTTCCTGTTTGGTCACTTGATGCCTCCGTGTAAGGGGGAATTTCTGTTCATGGGGGTAATGATACCGATGAAACGAGAGAGGATGCTCACGATACGGGTTACTGATGATGAACATGCCCGGTTACTGGAACGTTGTGAGGGTAAACAACTGGCGGTATGGATGCGGCGGGACCAGAGAAAAATCACTCAGGGTCAATGCCAGCGCTTCGTTAATACAGATGTAGGTGTTCCACAGGGTAGCCAGCAGCATCCTGCGATGCAGATCCGGAACATAATGGTGCAGGGCGCTGACTTCCGCGTTTCCAGACTTTACGAAACACGGAAACCGAAGACCATTCATGTTGTTGCTCAGGTCGCAGACGTTTTGCAGCAGCAGTCGCTTCACGTTCGCTCGCGTATCGGTGATTCATTCTGCTAACCAGTAAGGCAACCCCGCCAGCCTAGCCGGGTCCTCAACGACAGGAGCACGATCATGCGCACCCGTGGCCAGGACCCAACGCTGCCCGAAATT |
| pRC1884  (pMAL-c2x, Hsmar1 Q187P, no MBP-tag) | CCGACACCATCGAATGGTGCAAAACCTTTCGCGGTATGGCATGATAGCGCCCGGAAGAGAGTCAATTCAGGGTGGTGAATGTGAAACCAGTAACGTTATACGATGTCGCAGAGTATGCCGGTGTCTCTTATCAGACCGTTTCCCGCGTGGTGAACCAGGCCAGCCACGTTTCTGCGAAAACGCGGGAAAAAGTGGAAGCGGCGATGGCGGAGCTGAATTACATTCCCAACCGCGTGGCACAACAACTGGCGGGCAAACAGTCGTTGCTGATTGGCGTTGCCACCTCCAGTCTGGCCCTGCACGCGCCGTCGCAAATTGTCGCGGCGATTAAATCTCGCGCCGATCAACTGGGTGCCAGCGTGGTGGTGTCGATGGTAGAACGAAGCGGCGTCGAAGCCTGTAAAGCGGCGGTGCACAATCTTCTCGCGCAACGCGTCAGTGGGCTGATCATTAACTATCCGCTGGATGACCAGGATGCCATTGCTGTGGAAGCTGCCTGCACTAATGTTCCGGCGTTATTTCTTGATGTCTCTGACCAGACACCCATCAACAGTATTATTTTCTCCCATGAAGACGGTACGCGACTGGGCGTGGAGCATCTGGTCGCATTGGGTCACCAGCAAATCGCGCTGTTAGCGGGCCCATTAAGTTCTGTCTCGGCGCGTCTGCGTCTGGCTGGCTGGCATAAATATCTCACTCGCAATCAAATTCAGCCGATAGCGGAACGGGAAGGCGACTGGAGTGCCATGTCCGGTTTTCAACAAACCATGCAAATGCTGAATGAGGGCATCGTTCCCACTGCGATGCTGGTTGCCAACGATCAGATGGCGCTGGGCGCAATGCGCGCCATTACCGAGTCCGGGCTGCGCGTTGGTGCGGATATCTCGGTAGTGGGATACGACGATACCGAAGACAGCTCATGTTATATCCCGCCGTTAACCACCATCAAACAGGATTTTCGCCTGCTGGGGCAAACCAGCGTGGACCGCTTGCTGCAACTCTCTCAGGGCCAGGCGGTGAAGGGCAATCAGCTGTTGCCCGTCTCACTGGTGAAAAGAAAAACCACCCTGGCGCCCAATACGCAAACCGCCTCTCCCCGCGCGTTGGCCGATTCATTAATGCAGCTGGCACGACAGGTTTCCCGACTGGAAAGCGGGCAGTGAGCGCAACGCAATTAATGTGAGTTAGCTCACTCATTAGGCACAATTCTCATGTTTGACAGCTTATCATCGACTGCACGGTGCACCAATGCTTCTGGCGTCAGGCAGCCATCGGAAGCTGTGGTATGGCTGTGCAGGTCGTAAATCACTGCATAATTCGTGTCGCTCAAGGCGCACTCCCGTTCTGGATAATGTTTTTTGCGCCGACATCATAACGGTTCTGGCAAATATTCTGAAATGAGCTGTTGACAATTAATCATCGGCTCGTATAATGTGTGGAATTGTGAGCGGATAACAATTTCACACAGGAAACAGCCAGTCCGTTTAGGTGTTTTCACGAGCACTTCACCAACAAGGACCATAGCATATGGAAATGATGCTCGATAAGAAACAGATTCGTGCGATCTTTCTCTTTGAGTTTAAAATGGGTCGCAAAGCGGCGGAGACGACGCGTAATATTAACAACGCGTTCGGTCCTGGCACCGCGAACGAGCGTACCGTGCAATGGTGGTTCAAAAAGTTTCGCAAAGGCGACGAATCTCTGGAGGACGAAGAGCGTTCTGGCCGCCCGTCCGAGGTTGACAACGACCAGCTGCGTGCAATCATCGAAGCTGATCCGCTGACTACCACCCGCGAAGTTGCTGAAGAACTGAATGTGGATCACTCTACTGTGGTTCGCCACCTGAAACAGATCGGTAAAGTAAAAAAACTGGACAAATGGGTTCCTCATGAACTGTCTGAAAACCAGAAAAACCGTCGTTTCGAAGTTAGCTCCTCTCTGATTCTGCGTAACAACAACGAACCGTTCCTGGATCGTATCGTAACCTGTGATGAGAAATGGATTCTGTATGATAACCGTCGCCGCTCTGCTCAGTGGCTGGATCGCGAAGAAGCTCCAAAACACTTCCCGAAACCGAATCTGCACCcGAAGAAAGTCATGGTAACCGTATGGTGGTCTGCCGCAGGTGTTATCCACTATTCCTTCCTGAACCCGGGCGAAACTATCACCAGCGAAAAATACTGCCAGCAGATTGACGAAATGCACCGTAAACTGCAGCGTCTGCAGCCAGCACTGGTGAATCGTAAAGGTCCGATCCTGCTGCATGATAACGCCCGTCCGCACGTTGCCCAACCGACCCTGCAGAAACTGAACGAACTGGGCTATGAAGTTCTGCCACACCCGCCGTACTCCCCGGATCTGTCCCCGACTGACTACCATTTCTTCAAGCATCTGGACAACTTCCTGCAGGGTAAACGTTTTCACAACCAACAGGACGCAGAAAACGCTTTCCAGGAGTTCGTCGAAAGCCGTTCCACTGACTTCTACGCGACCGGTATCAACAAGCTGATCAGCCGTTGGCAGAAATGCGTGGACTGTAACGGCAGCTACTTCGATTAAGGATCCTCTAGAGTCGACCTGCAGGCAAGCTTGGCACTGGCCGTCGTTTTACAACGTCGTGACTGGGAAAACCCTGGCGTTACCCAACTTAATCGCCTTGCAGCACATCCCCCTTTCGCCAGCTGGCGTAATAGCGAAGAGGCCCGCACCGATCGCCCTTCCCAACAGTTGCGCAGCCTGAATGGCGAATGGCAGCTTGGCTGTTTTGGCGGATGAGATAAGATTTTCAGCCTGATACAGATTAAATCAGAACGCAGAAGCGGTCTGATAAAACAGAATTTGCCTGGCGGCAGTAGCGCGGTGGTCCCACCTGACCCCATGCCGAACTCAGAAGTGAAACGCCGTAGCGCCGATGGTAGTGTGGGGTCTCCCCATGCGAGAGTAGGGAACTGCCAGGCATCAAATAAAACGAAAGGCTCAGTCGAAAGACTGGGCCTTTCGTTTTATCTGTTGTTTGTCGGTGAACGCTCTCCTGAGTAGGACAAATCCGCCGGGAGCGGATTTGAACGTTGCGAAGCAACGGCCCGGAGGGTGGCGGGCAGGACGCCCGCCATAAACTGCCAGGCATCAAATTAAGCAGAAGGCCATCCTGACGGATGGCCTTTTTGCGTTTCTACAAACTCTTTTTGTTTATTTTTCTAAATACATTCAAATATGTATCCGCTCATGAGACAATAACCCTGATAAATGCTTCAATAATATTGAAAAAGGAAGAGTATGAGTATTCAACATTTCCGTGTCGCCCTTATTCCCTTTTTTGCGGCATTTTGCCTTCCTGTTTTTGCTCACCCAGAAACGCTGGTGAAAGTAAAAGATGCTGAAGATCAGTTGGGTGCACGAGTGGGTTACATCGAACTGGATCTCAACAGCGGTAAGATCCTTGAGAGTTTTCGCCCCGAAGAACGTTCTCCAATGATGAGCACTTTTAAAGTTCTGCTATGTGGCGCGGTATTATCCCGTGTTGACGCCGGGCAAGAGCAACTCGGTCGCCGCATACACTATTCTCAGAATGACTTGGTTGAGTACTCACCAGTCACAGAAAAGCATCTTACGGATGGCATGACAGTAAGAGAATTATGCAGTGCTGCCATAACCATGAGTGATAACACTGCGGCCAACTTACTTCTGACAACGATCGGAGGACCGAAGGAGCTAACCGCTTTTTTGCACAACATGGGGGATCATGTAACTCGCCTTGATCGTTGGGAACCGGAGCTGAATGAAGCCATACCAAACGACGAGCGTGACACCACGATGCCTGTAGCAATGGCAACAACGTTGCGCAAACTATTAACTGGCGAACTACTTACTCTAGCTTCCCGGCAACAATTAATAGACTGGATGGAGGCGGATAAAGTTGCAGGACCACTTCTGCGCTCGGCCCTTCCGGCTGGCTGGTTTATTGCTGATAAATCTGGAGCCGGTGAGCGTGGGTCTCGCGGTATCATTGCAGCACTGGGGCCAGATGGTAAGCCCTCCCGTATCGTAGTTATCTACACGACGGGGAGTCAGGCAACTATGGATGAACGAAATAGACAGATCGCTGAGATAGGTGCCTCACTGATTAAGCATTGGTAACTGTCAGACCAAGTTTACTCATATATACTTTAGATTGATTTACCCCGGTTGATAATCAGAAAAGCCCCAAAAACAGGAAGATTGTATAAGCAAATATTTAAATTGTAAACGTTAATATTTTGTTAAAATTCGCGTTAAATTTTTGTTAAATCAGCTCATTTTTTAACCAATAGGCCGAAATCGGCAAAATCCCTTATAAATCAAAAGAATAGACCGAGATAGGGTTGAGTGTTGTTCCAGTTTGGAACAAGAGTCCACTATTAAAGAACGTGGACTCCAACGTCAAAGGGCGAAAAACCGTCTATCAGGGCGATGGCCCACTACGTGAACCATCACCCAAATCAAGTTTTTTGGGGTCGAGGTGCCGTAAAGCACTAAATCGGAACCCTAAAGGGAGCCCCCGATTTAGAGCTTGACGGGGAAAGCCGGCGAACGTGGCGAGAAAGGAAGGGAAGAAAGCGAAAGGAGCGGGCGCTAGGGCGCTGGCAAGTGTAGCGGTCACGCTGCGCGTAACCACCACACCCGCCGCGCTTAATGCGCCGCTACAGGGCGCGTAAAAGGATCTAGGTGAAGATCCTTTTTGATAATCTCATGACCAAAATCCCTTAACGTGAGTTTTCGTTCCACTGAGCGTCAGACCCCGTAGAAAAGATCAAAGGATCTTCTTGAGATCCTTTTTTTCTGCGCGTAATCTGCTGCTTGCAAACAAAAAAACCACCGCTACCAGCGGTGGTTTGTTTGCCGGATCAAGAGCTACCAACTCTTTTTCCGAAGGTAACTGGCTTCAGCAGAGCGCAGATACCAAATACTGTCCTTCTAGTGTAGCCGTAGTTAGGCCACCACTTCAAGAACTCTGTAGCACCGCCTACATACCTCGCTCTGCTAATCCTGTTACCAGTGGCTGCTGCCAGTGGCGATAAGTCGTGTCTTACCGGGTTGGACTCAAGACGATAGTTACCGGATAAGGCGCAGCGGTCGGGCTGAACGGGGGGTTCGTGCACACAGCCCAGCTTGGAGCGAACGACCTACACCGAACTGAGATACCTACAGCGTGAGCTATGAGAAAGCGCCACGCTTCCCGAAGGGAGAAAGGCGGACAGGTATCCGGTAAGCGGCAGGGTCGGAACAGGAGAGCGCACGAGGGAGCTTCCAGGGGGAAACGCCTGGTATCTTTATAGTCCTGTCGGGTTTCGCCACCTCTGACTTGAGCGTCGATTTTTGTGATGCTCGTCAGGGGGGCGGAGCCTATGGAAAAACGCCAGCAACGCGGCCTTTTTACGGTTCCTGGCCTTTTGCTGGCCTTTTGCTCACATGTTCTTTCCTGCGTTATCCCCTGATTCTGTGGATAACCGTATTACCGCCTTTGAGTGAGCTGATACCGCTCGCCGCAGCCGAACGACCGAGCGCAGCGAGTCAGTGAGCGAGGAAGCGGAAGAGCGCCTGATGCGGTATTTTCTCCTTACGCATCTGTGCGGTATTTCACACCGCATATATGGTGCACTCTCAGTACAATCTGCTCTGATGCCGCATAGTTAAGCCAGTATACACTCCGCTATCGCTACGTGACTGGGTCATGGCTGCGCCCCGACACCCGCCAACACCCGCTGACGCGCCCTGACGGGCTTGTCTGCTCCCGGCATCCGCTTACAGACAAGCTGTGACCGTCTCCGGGAGCTGCATGTGTCAGAGGTTTTCACCGTCATCACCGAAACGCGCGAGGCAGCTGCGGTAAAGCTCATCAGCGTGGTCGTGCAGCGATTCACAGATGTCTGCCTGTTCATCCGCGTCCAGCTCGTTGAGTTTCTCCAGAAGCGTTAATGTCTGGCTTCTGATAAAGCGGGCCATGTTAAGGGCGGTTTTTTCCTGTTTGGTCACTTGATGCCTCCGTGTAAGGGGGAATTTCTGTTCATGGGGGTAATGATACCGATGAAACGAGAGAGGATGCTCACGATACGGGTTACTGATGATGAACATGCCCGGTTACTGGAACGTTGTGAGGGTAAACAACTGGCGGTATGGATGCGGCGGGACCAGAGAAAAATCACTCAGGGTCAATGCCAGCGCTTCGTTAATACAGATGTAGGTGTTCCACAGGGTAGCCAGCAGCATCCTGCGATGCAGATCCGGAACATAATGGTGCAGGGCGCTGACTTCCGCGTTTCCAGACTTTACGAAACACGGAAACCGAAGACCATTCATGTTGTTGCTCAGGTCGCAGACGTTTTGCAGCAGCAGTCGCTTCACGTTCGCTCGCGTATCGGTGATTCATTCTGCTAACCAGTAAGGCAACCCCGCCAGCCTAGCCGGGTCCTCAACGACAGGAGCACGATCATGCGCACCCGTGGCCAGGACCCAACGCTGCCCGAAATT |
| pRC1885  (pMAL-c2x, Hsmar1 V194I, no MBP-tag) | CCGACACCATCGAATGGTGCAAAACCTTTCGCGGTATGGCATGATAGCGCCCGGAAGAGAGTCAATTCAGGGTGGTGAATGTGAAACCAGTAACGTTATACGATGTCGCAGAGTATGCCGGTGTCTCTTATCAGACCGTTTCCCGCGTGGTGAACCAGGCCAGCCACGTTTCTGCGAAAACGCGGGAAAAAGTGGAAGCGGCGATGGCGGAGCTGAATTACATTCCCAACCGCGTGGCACAACAACTGGCGGGCAAACAGTCGTTGCTGATTGGCGTTGCCACCTCCAGTCTGGCCCTGCACGCGCCGTCGCAAATTGTCGCGGCGATTAAATCTCGCGCCGATCAACTGGGTGCCAGCGTGGTGGTGTCGATGGTAGAACGAAGCGGCGTCGAAGCCTGTAAAGCGGCGGTGCACAATCTTCTCGCGCAACGCGTCAGTGGGCTGATCATTAACTATCCGCTGGATGACCAGGATGCCATTGCTGTGGAAGCTGCCTGCACTAATGTTCCGGCGTTATTTCTTGATGTCTCTGACCAGACACCCATCAACAGTATTATTTTCTCCCATGAAGACGGTACGCGACTGGGCGTGGAGCATCTGGTCGCATTGGGTCACCAGCAAATCGCGCTGTTAGCGGGCCCATTAAGTTCTGTCTCGGCGCGTCTGCGTCTGGCTGGCTGGCATAAATATCTCACTCGCAATCAAATTCAGCCGATAGCGGAACGGGAAGGCGACTGGAGTGCCATGTCCGGTTTTCAACAAACCATGCAAATGCTGAATGAGGGCATCGTTCCCACTGCGATGCTGGTTGCCAACGATCAGATGGCGCTGGGCGCAATGCGCGCCATTACCGAGTCCGGGCTGCGCGTTGGTGCGGATATCTCGGTAGTGGGATACGACGATACCGAAGACAGCTCATGTTATATCCCGCCGTTAACCACCATCAAACAGGATTTTCGCCTGCTGGGGCAAACCAGCGTGGACCGCTTGCTGCAACTCTCTCAGGGCCAGGCGGTGAAGGGCAATCAGCTGTTGCCCGTCTCACTGGTGAAAAGAAAAACCACCCTGGCGCCCAATACGCAAACCGCCTCTCCCCGCGCGTTGGCCGATTCATTAATGCAGCTGGCACGACAGGTTTCCCGACTGGAAAGCGGGCAGTGAGCGCAACGCAATTAATGTGAGTTAGCTCACTCATTAGGCACAATTCTCATGTTTGACAGCTTATCATCGACTGCACGGTGCACCAATGCTTCTGGCGTCAGGCAGCCATCGGAAGCTGTGGTATGGCTGTGCAGGTCGTAAATCACTGCATAATTCGTGTCGCTCAAGGCGCACTCCCGTTCTGGATAATGTTTTTTGCGCCGACATCATAACGGTTCTGGCAAATATTCTGAAATGAGCTGTTGACAATTAATCATCGGCTCGTATAATGTGTGGAATTGTGAGCGGATAACAATTTCACACAGGAAACAGCCAGTCCGTTTAGGTGTTTTCACGAGCACTTCACCAACAAGGACCATAGCATATGGAAATGATGCTCGATAAGAAACAGATTCGTGCGATCTTTCTCTTTGAGTTTAAAATGGGTCGCAAAGCGGCGGAGACGACGCGTAATATTAACAACGCGTTCGGTCCTGGCACCGCGAACGAGCGTACCGTGCAATGGTGGTTCAAAAAGTTTCGCAAAGGCGACGAATCTCTGGAGGACGAAGAGCGTTCTGGCCGCCCGTCCGAGGTTGACAACGACCAGCTGCGTGCAATCATCGAAGCTGATCCGCTGACTACCACCCGCGAAGTTGCTGAAGAACTGAATGTGGATCACTCTACTGTGGTTCGCCACCTGAAACAGATCGGTAAAGTAAAAAAACTGGACAAATGGGTTCCTCATGAACTGTCTGAAAACCAGAAAAACCGTCGTTTCGAAGTTAGCTCCTCTCTGATTCTGCGTAACAACAACGAACCGTTCCTGGATCGTATCGTAACCTGTGATGAGAAATGGATTCTGTATGATAACCGTCGCCGCTCTGCTCAGTGGCTGGATCGCGAAGAAGCTCCAAAACACTTCCCGAAACCGAATCTGCACCAGAAGAAAGTCATGGTAACCaTtTGGTGGTCTGCCGCAGGTGTTATCCACTATTCCTTCCTGAACCCGGGCGAAACTATCACCAGCGAAAAATACTGCCAGCAGATTGACGAAATGCACCGTAAACTGCAGCGTCTGCAGCCAGCACTGGTGAATCGTAAAGGTCCGATCCTGCTGCATGATAACGCCCGTCCGCACGTTGCCCAACCGACCCTGCAGAAACTGAACGAACTGGGCTATGAAGTTCTGCCACACCCGCCGTACTCCCCGGATCTGTCCCCGACTGACTACCATTTCTTCAAGCATCTGGACAACTTCCTGCAGGGTAAACGTTTTCACAACCAACAGGACGCAGAAAACGCTTTCCAGGAGTTCGTCGAAAGCCGTTCCACTGACTTCTACGCGACCGGTATCAACAAGCTGATCAGCCGTTGGCAGAAATGCGTGGACTGTAACGGCAGCTACTTCGATTAAGGATCCTCTAGAGTCGACCTGCAGGCAAGCTTGGCACTGGCCGTCGTTTTACAACGTCGTGACTGGGAAAACCCTGGCGTTACCCAACTTAATCGCCTTGCAGCACATCCCCCTTTCGCCAGCTGGCGTAATAGCGAAGAGGCCCGCACCGATCGCCCTTCCCAACAGTTGCGCAGCCTGAATGGCGAATGGCAGCTTGGCTGTTTTGGCGGATGAGATAAGATTTTCAGCCTGATACAGATTAAATCAGAACGCAGAAGCGGTCTGATAAAACAGAATTTGCCTGGCGGCAGTAGCGCGGTGGTCCCACCTGACCCCATGCCGAACTCAGAAGTGAAACGCCGTAGCGCCGATGGTAGTGTGGGGTCTCCCCATGCGAGAGTAGGGAACTGCCAGGCATCAAATAAAACGAAAGGCTCAGTCGAAAGACTGGGCCTTTCGTTTTATCTGTTGTTTGTCGGTGAACGCTCTCCTGAGTAGGACAAATCCGCCGGGAGCGGATTTGAACGTTGCGAAGCAACGGCCCGGAGGGTGGCGGGCAGGACGCCCGCCATAAACTGCCAGGCATCAAATTAAGCAGAAGGCCATCCTGACGGATGGCCTTTTTGCGTTTCTACAAACTCTTTTTGTTTATTTTTCTAAATACATTCAAATATGTATCCGCTCATGAGACAATAACCCTGATAAATGCTTCAATAATATTGAAAAAGGAAGAGTATGAGTATTCAACATTTCCGTGTCGCCCTTATTCCCTTTTTTGCGGCATTTTGCCTTCCTGTTTTTGCTCACCCAGAAACGCTGGTGAAAGTAAAAGATGCTGAAGATCAGTTGGGTGCACGAGTGGGTTACATCGAACTGGATCTCAACAGCGGTAAGATCCTTGAGAGTTTTCGCCCCGAAGAACGTTCTCCAATGATGAGCACTTTTAAAGTTCTGCTATGTGGCGCGGTATTATCCCGTGTTGACGCCGGGCAAGAGCAACTCGGTCGCCGCATACACTATTCTCAGAATGACTTGGTTGAGTACTCACCAGTCACAGAAAAGCATCTTACGGATGGCATGACAGTAAGAGAATTATGCAGTGCTGCCATAACCATGAGTGATAACACTGCGGCCAACTTACTTCTGACAACGATCGGAGGACCGAAGGAGCTAACCGCTTTTTTGCACAACATGGGGGATCATGTAACTCGCCTTGATCGTTGGGAACCGGAGCTGAATGAAGCCATACCAAACGACGAGCGTGACACCACGATGCCTGTAGCAATGGCAACAACGTTGCGCAAACTATTAACTGGCGAACTACTTACTCTAGCTTCCCGGCAACAATTAATAGACTGGATGGAGGCGGATAAAGTTGCAGGACCACTTCTGCGCTCGGCCCTTCCGGCTGGCTGGTTTATTGCTGATAAATCTGGAGCCGGTGAGCGTGGGTCTCGCGGTATCATTGCAGCACTGGGGCCAGATGGTAAGCCCTCCCGTATCGTAGTTATCTACACGACGGGGAGTCAGGCAACTATGGATGAACGAAATAGACAGATCGCTGAGATAGGTGCCTCACTGATTAAGCATTGGTAACTGTCAGACCAAGTTTACTCATATATACTTTAGATTGATTTACCCCGGTTGATAATCAGAAAAGCCCCAAAAACAGGAAGATTGTATAAGCAAATATTTAAATTGTAAACGTTAATATTTTGTTAAAATTCGCGTTAAATTTTTGTTAAATCAGCTCATTTTTTAACCAATAGGCCGAAATCGGCAAAATCCCTTATAAATCAAAAGAATAGACCGAGATAGGGTTGAGTGTTGTTCCAGTTTGGAACAAGAGTCCACTATTAAAGAACGTGGACTCCAACGTCAAAGGGCGAAAAACCGTCTATCAGGGCGATGGCCCACTACGTGAACCATCACCCAAATCAAGTTTTTTGGGGTCGAGGTGCCGTAAAGCACTAAATCGGAACCCTAAAGGGAGCCCCCGATTTAGAGCTTGACGGGGAAAGCCGGCGAACGTGGCGAGAAAGGAAGGGAAGAAAGCGAAAGGAGCGGGCGCTAGGGCGCTGGCAAGTGTAGCGGTCACGCTGCGCGTAACCACCACACCCGCCGCGCTTAATGCGCCGCTACAGGGCGCGTAAAAGGATCTAGGTGAAGATCCTTTTTGATAATCTCATGACCAAAATCCCTTAACGTGAGTTTTCGTTCCACTGAGCGTCAGACCCCGTAGAAAAGATCAAAGGATCTTCTTGAGATCCTTTTTTTCTGCGCGTAATCTGCTGCTTGCAAACAAAAAAACCACCGCTACCAGCGGTGGTTTGTTTGCCGGATCAAGAGCTACCAACTCTTTTTCCGAAGGTAACTGGCTTCAGCAGAGCGCAGATACCAAATACTGTCCTTCTAGTGTAGCCGTAGTTAGGCCACCACTTCAAGAACTCTGTAGCACCGCCTACATACCTCGCTCTGCTAATCCTGTTACCAGTGGCTGCTGCCAGTGGCGATAAGTCGTGTCTTACCGGGTTGGACTCAAGACGATAGTTACCGGATAAGGCGCAGCGGTCGGGCTGAACGGGGGGTTCGTGCACACAGCCCAGCTTGGAGCGAACGACCTACACCGAACTGAGATACCTACAGCGTGAGCTATGAGAAAGCGCCACGCTTCCCGAAGGGAGAAAGGCGGACAGGTATCCGGTAAGCGGCAGGGTCGGAACAGGAGAGCGCACGAGGGAGCTTCCAGGGGGAAACGCCTGGTATCTTTATAGTCCTGTCGGGTTTCGCCACCTCTGACTTGAGCGTCGATTTTTGTGATGCTCGTCAGGGGGGCGGAGCCTATGGAAAAACGCCAGCAACGCGGCCTTTTTACGGTTCCTGGCCTTTTGCTGGCCTTTTGCTCACATGTTCTTTCCTGCGTTATCCCCTGATTCTGTGGATAACCGTATTACCGCCTTTGAGTGAGCTGATACCGCTCGCCGCAGCCGAACGACCGAGCGCAGCGAGTCAGTGAGCGAGGAAGCGGAAGAGCGCCTGATGCGGTATTTTCTCCTTACGCATCTGTGCGGTATTTCACACCGCATATATGGTGCACTCTCAGTACAATCTGCTCTGATGCCGCATAGTTAAGCCAGTATACACTCCGCTATCGCTACGTGACTGGGTCATGGCTGCGCCCCGACACCCGCCAACACCCGCTGACGCGCCCTGACGGGCTTGTCTGCTCCCGGCATCCGCTTACAGACAAGCTGTGACCGTCTCCGGGAGCTGCATGTGTCAGAGGTTTTCACCGTCATCACCGAAACGCGCGAGGCAGCTGCGGTAAAGCTCATCAGCGTGGTCGTGCAGCGATTCACAGATGTCTGCCTGTTCATCCGCGTCCAGCTCGTTGAGTTTCTCCAGAAGCGTTAATGTCTGGCTTCTGATAAAGCGGGCCATGTTAAGGGCGGTTTTTTCCTGTTTGGTCACTTGATGCCTCCGTGTAAGGGGGAATTTCTGTTCATGGGGGTAATGATACCGATGAAACGAGAGAGGATGCTCACGATACGGGTTACTGATGATGAACATGCCCGGTTACTGGAACGTTGTGAGGGTAAACAACTGGCGGTATGGATGCGGCGGGACCAGAGAAAAATCACTCAGGGTCAATGCCAGCGCTTCGTTAATACAGATGTAGGTGTTCCACAGGGTAGCCAGCAGCATCCTGCGATGCAGATCCGGAACATAATGGTGCAGGGCGCTGACTTCCGCGTTTCCAGACTTTACGAAACACGGAAACCGAAGACCATTCATGTTGTTGCTCAGGTCGCAGACGTTTTGCAGCAGCAGTCGCTTCACGTTCGCTCGCGTATCGGTGATTCATTCTGCTAACCAGTAAGGCAACCCCGCCAGCCTAGCCGGGTCCTCAACGACAGGAGCACGATCATGCGCACCCGTGGCCAGGACCCAACGCTGCCCGAAATT |
[truncated: 207,946 more chars]
